# Moths (Insecta: Lepidoptera) of Delhi, India: An illustrated checklist based on museum specimens and surveys

**DOI:** 10.3897/BDJ.9.e73997

**Published:** 2021-10-06

**Authors:** J. Komal, P. R. Shashank, Sanjay Sondhi, Sohail Madan, Yash Sondhi, Naresh M. Meshram, S. S. Anooj

**Affiliations:** 1 National Pusa Collection, Division of Entomology, ICAR-Indian Agricultural Research Institute, New Delhi, India National Pusa Collection, Division of Entomology, ICAR-Indian Agricultural Research Institute New Delhi India; 2 Titli Trust, 49 Rajpur Road Enclave, Dhoran Khas, near IT Park, P.O. Gujrada, Dehradun, Uttarakhand, India Titli Trust, 49 Rajpur Road Enclave, Dhoran Khas, near IT Park, P.O. Gujrada Dehradun, Uttarakhand India; 3 Conservation Education Centre - ABWLS, Delhi Asola Bhatti Wildlife Sanctuary, Near Karni Singh Shooting Range, New Delhi, India Conservation Education Centre - ABWLS, Delhi Asola Bhatti Wildlife Sanctuary, Near Karni Singh Shooting Range New Delhi India; 4 Department of Biology, Florida International University, Miami, Florida, United States of America Department of Biology, Florida International University Miami, Florida United States of America; 5 ICAR- Central Citrus Research Institute, Nagpur, India ICAR- Central Citrus Research Institute Nagpur India

**Keywords:** species checklist, biodiversity inventory, Pusa, Heterocera, India

## Abstract

**Background:**

There have been several recent checklists, books and publications about Indian moths; however, much of this work has focused on biodiversity hotspots such as North-east India, Western Ghats and Western Himalayas. There is a lack of published literature on urban centres in India, despite the increased need to monitor insects at sites with high levels of human disturbance. In this study, we examine the moths of Delhi, the national capital region of India, one of the fastest growing mega-metropolitan cities. We present a comprehensive checklist of 338 moths species using 8 years of light trapping data (2012-2020) and examining about 2000 specimens from historical collections at the National Pusa Collection of ICAR-Indian Agricultural Research Institute, New Delhi (NPC-IARI) spanning over 100 years (1907-2020). The checklist comprises moths from 32 families spanning 14 superfamilies with Noctuoidea (48.5%) and Pyraloidea (20.4%) being the the two most dominant superfamilies. We provide links to images of live individuals and pinned specimens for all moths and provide detailed distribution records and an updated taxonomic treatment.

**New information:**

This is the first comprehensive annotated checklist of the moths of Delhi. The present study adds 234 species to the biodiversity of moths from Delhi that were not reported previously, along with illustrations for 195 species.

## Introduction

Lepidoptera Linnaeus, 1758 which includes butterflies and moths, is one of the largest insect orders consisting of 45 super families and having 157,424 species described (Van Nieukerken et al. 2011). It constitutes 10% of the total described species of living organisms (Mallet 2007). Of these, moths form roughly 85% of all known Lepidoptera, with over 12000 known species of moths from the Indian subcontinent (Chandra 2007). Moths are ecologically and economically significant as a primary food source for vertebrate insectivores, as pests of crop plants (Common 1990), pollinators (MacGregor et al. 2015), food for humans (Zagrobelny et al. 2009) and model organisms in scientific research (Roe and Just 2009). Nevertheless, the recent reports on insect decline are alarming and it is also evident in decline in moth diversity and abundance around the world (Hallmann et al. 2020). Numerous factors contribute to the decline of moths, such as rapid urbanisation, habitat loss, artificial light, intensive agriculture, pesticide pollution and lack of conservation policies (Dennis et al. 2019). These reports on global insect declines highlight the need for better conservation and management; however, they need to occur in tandem with ongoing monitoring and cataloguing of insects. Much of the work on Indian moth fauna was done pre-independence, including Hampson (1891), Hampson (1892), Hampson (1894), Hampson (1895), Hampson (1896), Fletcher (1920), Fletcher (1932), Fletcher (1933), Moore (1880), Moore (1882), Moore (1884), Bell and Scott (1937) and while they are extensive contributions to Indian moth fauna, these works are in need of a systematic update with additional modern surveys and current taxonomy. The more recent studies on moth fauna by Indian authors have been growing in number and include surveys, based checklists on the moth fauna of specific regions, viz. Mathew and Rahmathulla (1995) (Silent Valley National Park, Kerala, 318 species), Rose (2002) (Jatinga, Assam, 81 species), Shubhalaxmi et al. (2011) (northern Western Ghats, 418 species), Gurule and Nikam (2013) (northern Maharashtra, 245 species), Sondhi and Sondhi (2016) (Garhwal, Uttarakhand, 248 species), Singh et al. (2017) (North East Jharkhand, 81 species), Dey et al. (2018) (Wildlife Institute of India, Dehradun, 291 species), Sondhi et al. (2018) (Agastyamalai Biosphere Reserve, Kerala, 282 species), Dar et al. (2020) (Jammu and Kashimir, 461 species) and Bhagat (2020) (Jammu and Kashmir, and Ladakh, 55 species). There has been a tendencey to focus on studying regions of higher biodiversity, including the Western and Eastern Himalayas and the Western Ghats; however, there has been much less work done from regions with higher levels of human disturbance or metropolitian cities in India.

In this study, we focus on the moth fauna of Delhi, the National capital territory of India, one of the largest growing metropolitan centres in the world with an estimated population of 23 million (MPD 2021). Delhi, with a geographical coverage of 783 km^2^ , extends on the western bank of River Yamuna between 28º12' and 28º53' N latitude and 76º50' and 77º23' E longitude and is bound on the northeast by the Indo-Gangetic plain and on the southeast by the Thar Desert (Dakshini 1968). The prominent component of the natural vegetation is the Delhi Ridge forest which is an outcrop of the Aravali Hills, one of the oldest chains of hills in the world. Open scrub forest, classified under the Tropical Dry Thorn Forest type (Champion and Seth 1968), covers a large extent of the ridge. Such vegetation type is widely distributed in the arid and semi-arid zones of the Earth where the total annual rainfall ranges from 50-100 mm. Tree species commonly found in Delhi, in such vegetation include, *Acacia leucoplachia, Prosopis cineraria, Ziziphus nummularia, Anogeissus pendula* etc. (Maheshwari 1953, Maheshwari 1963). Additionally, *Prosopisjuliflora*, an exotic species introduced, as part of the afforestation drive, also dominates this thorny vegetation (Sinha 2014). Another prominent feature of the natural vegetation in Delhi is the Dichanthium-Cenchrus-Lasiurus grasslands (Dabadghao et al. 1973). The fertile alluvial plains of the State support agricultural crops which also influence the moth diversity of the region by favouring many heteroceran agricultural pests. Donahue (1966), in his study of Butterflies of Delhi, identified two distinct habitats in Delhi: the arid xerophytic Aravalli Ridge (Delhi Ridge) and mesophytic urban nursery area. Though the city is a highly urbanised landscape with a human population of about 16.75 million (as per 2011 census), it holds a forest cover of about 13.18% (Forest Survey of India 2019), one of the largest percentages of forest cover when compared to other Indian cities. This, along with factors like the presence of the Yamuna River and its nearness to the Himalayas, adds to factors that augment biodiversity of the area. Much of the rainfall in Delhi is received during the months of July to August during which the otherwise dry vegetation shows luxuriant growth and supports the insect diversity.

In general, the insect fauna of Delhi has received less attention with only very few groups like butterflies (Lepidoptera) (Biswas et al. 2017) and Odonata (Nazneen 2019) being well documented. The studies on moth fauna of Delhi were always insufficient, the State fauna series of Delhi, published by the Zoological Survey of India in the year 1997, included only 11 species of moths (Ghosh and Varshney 1997). Later, after two decades, Paul et al. (2016) added 36 species of moths to the biodiversity of Delhi which included mostly agricultural pests. The recent checklist of moths of Delhi consists of only 74 species (Paul et al. 2017, Paul 2021).

There are limited studies in India that have utilised moth collections preserved in museums and none that have integrated this with primary survey data and secondary data from literature and citizen science projects. In the present work, we have studied the moth collections at National Pusa Collection, Division of Entomology, ICAR-Indian Agricultural Research Institute, New Delhi (NPC-IARI) which is one of the four important Lepidoptera collections in India (Smetacek 2011). NPC houses over 0.4 million specimens, comprising 56000 specimens of Lepidoptera representing 3300 species. NPC has an illustrious history in agriculturally important insect pest collection. Famous lepidopterists such as T.B. Fletcher and Edward Meyrick, worked on these collections prior to India’s independence. However, after independence, there are only a few experts on moths who could visit and work on these collections. In the current study, we studied all the moths that are collected from Delhi housed in NPC-IARI, including our own observations from 2012-2020 and data from different citizen science portals. An illustrated checklist of the 338 moths found in Delhi, along with up-to-date taxonomic treatment, are presented.

## Materials and methods

### Museum specimens

In the present study, the biodiversity of moths of the region was studied by an exhaustive exploration of the museum holdings of the National Pusa Collection, Department of Entomology at ICAR-Indian Agricultural Research Institute, Delhi (NPC-IARI) which is one of the largest insect repositories in Asia for agricultural pests since the 1900s. The specimens of moths belonging to Delhi were sorted separately for the present study. A database has been created from individual specimens, based on label data including the name of the collector, date of collection, method of collection, associated host plants and sex. This includes more than 1500 specimens since 1907 up to 2020 which can be accessed at *Moths of Delhi, India dataset*. Furthermore, identification and reconfirmation of all the specimens was done and were updated to their current taxonomic positions. All the representative species were photographed with a Cannon 70D with a 100 mm macro lens. The micromoths were photographed with a digitalised camera Leica DFC 425C on the Leica 19205FA Stereozoom Automountage microscope.

### Field surveys

Field surveys were conducted from 2012 to 2020 by setting up light traps at different locations, viz. the Indian Agricultural Research Institute (ICAR- IARI), Pusa (28.04°N, 77.12°E), Rashtrapathi Bhawan (28.61°N, 77.19°E) and Asola Bhatti Wildlife Sanctuary (28.4762°N, 77.23°E). This accounted for a total of 73 survey nights. The light traps were set after sunset during the evening hours generally for 5 hours from 6 to 11 PM using a Mercury vapour bulb of 160 W. Most of the time, electrical mains were available for surveys, but a portable diesel-based generator was used to set the light traps at the locations without a source of electricity. All the moths were photographed in the field using a Cannon 70D with a 100 mm macro lens.

### Identification and preparation of checklist

The available literature was used to identify the moths, including Barlow (1982), Holloway (1999), Holloway (1998), Holloway (1996), Holloway (1993), Holloway (1989), Holloway (1986), Holloway (1987), Holloway (1983), Holloway (1985), Holloway (2003), Holloway (2011), Holloway (1997), Holloway (1988), Bell and Scott (1937), Kononenko and Pinratana (2005), Kononenko and Pinratana (2013), Moore (1880), Moore (1882), Moore (1884), Schintlmeister and Pinratana (2007), Zolotuhin and Pinratana (2005), Kirti and Singh (2015), Kirti and Singh (2016), Kendrick (2002), Hampson (1891), Hampson (1895), Robinson et al. (1994) and Inoue et al. (1996).

Along with the above museum collection data and surveys, additionally, data from citizen science internet portals, such as the Moths of India (http://www.mothsofindia.org/; Sondhi et al. 2021), iNaturalist (https://www.inaturalist.org) and India Biodiversity (http://indiabiodiversity.org/), were also used to prepare the checklist. For a few morphospecies, we could not identify up to species and we have mentioned only genera name and numbers.

Finally, a comprehensive checklist has been prepared by including all the data from museum specimens, field surveys, available literature and citizen science portals. The classification system used by Van Nieukerken et al. (2011) was followed. Systematic arrangement was made alphabetically, the checklist being presented below with notes mentioning previous reports. Additional data related to materials studied can be accessed here: http://ipt.pensoft.net/resource?r=moths_of_delhi&amp;v=1.8. Representative species photographs of museum specimens and also those captured in the field are arranged into plates alphabetically.

## Checklists

### Checklist

#### Lepidoptera

Linnaeus, 1758

E671E34E-877B-513C-A364-7F55184B50F8

#### Bombyx mori

(Linnaeus, 1758)

5D105D90-4B6E-5DE0-A17D-9D3DA5D731FD

##### Notes

Present study; Fig. 1a

#### Trilocha varians

(Walker, 1855)

940FF476-F0D3-578B-BA5F-BBBDACF91B55

##### Notes

Present study; Fig. 1b

#### Eupterote fabia

(Cramer, 1780)

051665F2-49A4-5E52-9BF7-177825E4EA01

##### Notes

Paul et al. 2017

#### Eupterote undata

Blanchard, [1844]

2A4B1F14-F7D2-5DCE-91E0-14D11B6811EA

##### Notes

Present study; Fig. 1c

#### Cephonodes hylas

(Linnaeus, 1771)

EEA51E5F-B2DA-5C72-AC74-9A6E34F7A735

##### Notes

Inaturalist, Present study; Fig. 1d

#### Daphnis nerii

(Linnaeus, 1758)

590E1054-84A4-5ADA-AC58-0B8CEE084338

##### Notes

Present study

#### Hippotion boerhaviae

(Fabricius, 1775)

A6A3FB1B-9362-50FE-8C0F-D824425A9481

##### Notes

Present study; Fig. 1e

#### Hippotion celerio

(Linnaeus, 1758)

6FFE461A-90E2-5AAF-8585-E8D6761EF9BF

##### Notes

Paul et al. 2017, Present study; Fig. 1f

#### Hyles lineata

(Fabricius, 1775)

CAF64F9C-1B20-522C-AEB9-B415F4A05D16

##### Notes

Present study; Fig. 2a

#### Macroglossum neotroglodytus

Kitching & Cadiou, 2000

D695DF4A-43C8-5158-BB6B-91551E603F60

##### Notes

Present study; Fig. 2b

#### Macroglossum stellatarum

(Linnaeus, 1758)

E5E3959B-1F52-5C93-9335-B443FFA527A2

##### Notes

Present study

#### Nephele hespera

(Fabricius, 1775)

1ABF43F2-7AD7-5A47-9D13-3B22DE2F0F11

##### Notes

Present study; Fig. 2c

#### Theretra alecto

(Linnaeus, 1758)

73F2C989-5689-561A-AFF8-01524CB566F8

##### Notes

Present study; Fig. 2d

#### Theretra nessus

(Drury, 1773)

85545900-C243-53C3-B0D4-D57002CCE605

##### Notes

Present study; Fig. 2e

#### Theretra oldenlandiae

(Fabricius, 1775)

9DDDC540-308B-55CF-9CB0-40B856BAAEDE

##### Notes

Paul et al. 2017

#### Theretra silhetensis

(Walker, 1856)

898199BE-6987-50A1-A43C-BC6CC6887577

##### Notes

Paul et al. 2017

#### Clanis phalaris

(Cramer, 1777)

257CA43C-65D4-5D49-964E-3C34E2BA9B99

##### Notes

Paul et al. 2017

#### Clanis sp.

E9DA1853-BF72-5078-BAEE-8C4F8E2151D3

##### Notes

Present study; Fig. 2f

#### Leucophlebia lineata

Westwood, 1847

0CFC0894-8462-5D6E-923A-017E50EB15A8

##### Notes

Present study

#### Acherontia lachesis

(Fabricius, 1798)

17ADD229-DE3D-59B3-9BD0-52AA229DB87E

##### Notes

Present study

#### Acherontia styx

(Westwood, 1847)

B1AABE30-9348-573B-8FD7-F8ECF63E5492

##### Notes

Paul et al. 2017, Present study; Fig. 3a

#### Agrius convolvuli

(Linnaeus, 1758)

10B98C85-8DA4-5BB2-83FF-7EDD606833A0

##### Notes

Kumar et al. 2012, Paul et al. 2017, Present study; Fig. 3b

#### Psilogramma increta

(Walker 1865)

6A30D89C-7687-559C-B16F-E832B31D3EEE

##### Notes

Paul et al. 2017, Present study; Fig. 3c

#### Psilogramma sp.

83504E58-2010-5997-84BE-A7AC5D4334F7

##### Notes

Paul et al. 2017

#### Phycodes minor

Moore, 1881

455E6C08-0764-5B65-98C8-CD46421EE5FE

##### Notes

Fig. 3d

#### Exinotis catachlora

Meyrick, 1916

14E25F82-306B-576E-8861-20372864BE5E

##### Notes

Present study; Fig. 3e

#### Coleophora sp.

0A0C1B39-6C0B-57DD-A859-AFAB8D8E104A

##### Notes

Present study; Fig. 3f

#### Ascalenia crypsiloga

(Meyrick, 1915)

340DABF4-A3A5-52DB-A4BA-BFDA00CAB54D

##### Notes

Present study; Fig. 4a

#### Anatrachyntis sp.

86304A3A-D119-5724-B106-EC0D8EC5C8DF

##### Notes

Present study; Fig. 4b

#### Ethmia acontias

Meyrick, 1906

CFDA71FE-736F-5C5C-9B24-7EE5C438B0E7

##### Notes

Present study; Fig. 4c

#### Ethmia sp.

752386DA-D1D9-5D5C-A252-C5AD4E7FE83F

##### Notes

Present study

#### Psorosticha zizyphi

(Stainton, 1859)

E966C606-8952-5CB0-8587-3C28BAA4BDD9

##### Notes

Present study; Fig. 4d

#### Anarsia ephippias

Meyrick, 1908

6DAFC95F-E4DD-558F-8676-4E5A570FAD65

##### Notes

Present study; Fig. 4e

#### Anarsia lineatella

Zeller, 1839

C1A4649F-990F-51ED-B7E1-AD5741D749FF

##### Notes

Present study

#### Helcystogramma engraptum

(Meyrick, 1918)

9B0B62B2-F2D2-50C8-BC8E-1C79EE4EC46A

##### Notes

Present study; Fig. 4f

#### Helcystogramma hibisci

(Stainton, 1859)

E2E93F31-94B5-55D1-8C4C-1D1865214763

##### Notes

Present study; Fig. 5a

#### Phthorimaea operculella

(Zeller, 1873)

8A993B6F-849A-50DC-8702-2AD74AE93564

##### Notes

Kumar et al. 2012, Present study; Fig. 5b

#### Pseudodoxia albinea

Meyrick 1914

B1B373F6-EE9B-5E78-99BB-86E4425CD371

##### Notes

Present study; Fig. 5c

#### Eretmocera impactella

(Walker, 1864)

757A486F-1894-5B99-B828-824F9EC49466

##### Notes

Present study; Fig. 5d

#### Stathmopoda sp.

5DFEAD83-176E-5ECE-8A34-F2FEDDAD353B

##### Notes

Present study; Fig. 5e

#### Chiasmia fidoniata

(Guenee, 1858)

41062A58-068D-5C2E-BC72-AD06C3572E0A

##### Notes

Present study

#### Chiasmia frugaliata

(Guenee, 1858)

8F5BC556-6CBF-5A1E-8DB7-EA43782197C9

##### Notes

Paul et al. 2017

#### Chiasmia nora

(Walker, 1861)

5246F8FD-820C-5967-86A1-B8B96866BEC4

##### Notes

Present study; Fig. 5f

#### Chiasmia sp. 1

E6CA1B50-F53D-5D80-B099-DE7CE79F55C1

##### Notes

Paul et al. 2017

#### Chiasmia sp. 2

7B5BED2A-81B3-5724-90EE-9E4EB3C8C150

##### Notes

Present study

#### Cleora acaciaria

(Boisduval, 1833)

34DE94BF-D6C1-5C4D-84F1-30C5FCD8F30D

##### Notes

Paul et al. 2017

#### Cleora cornaria

(Guenée, 1857)

1B2F6DF5-D469-5E8D-A588-405FD0675A8A

##### Notes

Paul et al. 2017

#### Cleora sp.

90713B70-038A-5C03-A178-8B04DEC279B5

##### Notes

Present study; Fig. 6a

#### Hyperythra lutea

(Stoll, [1781])

8CF70F17-7AD3-5EF9-86AA-A4EA535FF446

##### Notes

Present study

#### Hyperythra swinhoei

Butler, 1880

000FD01D-E293-5172-A432-3101F420BC45

##### Notes

Present study; Fig. 6b

#### Hyposidra talaca

(Walker, 1860)

33AC87F7-2217-5765-9BD9-1CFD00CE1011

##### Notes

Present study

#### Isturgia arenacearia

(Denis & Schiffermüller, 1775)

4F8A33B2-B870-5F30-A400-DF19936F57DC

##### Notes

Present study

#### Isturgia disputaria

(Guenée, [1858])

E74A30D7-E133-5591-B8FD-02B90CED9DC9

##### Notes

Paul et al. 2017

#### Petelia medardaria

Herrich-Schäffer, [1856]

D792BDA7-E28B-5983-B306-AC5CBDC81A06

##### Notes

Present study

#### Petelia sp.

0C7585A2-2126-5743-908F-748CBD936534

##### Notes

Present study; Fig. 6c

#### Rhodometra sacraria

(Linnaeus, 1767)

CAE5F177-B096-5041-AC16-48F65C165C60

##### Notes

Paul et al. 2017, Present study; Fig. 6d

#### Scardamia metallaria

Guenée, 1858

B11F7AD3-8EEB-5DBB-82DE-E95A6A6F068B

##### Notes

Present study; Fig. 6e

#### Hemithea aestivaria

(Hübner, 1799)

B7A38E5E-135B-5EBD-A46C-2CAF7F7FE1E5

##### Notes

Present study

#### Ornithospila avicularia

(Guenée, 1857)

2E9F0EE3-C673-5630-8505-9A858B766011

##### Notes

Present study; Fig. 6f

#### Pelagodes veraria

(Guenée, 1857)

87F88EBA-B179-50D4-8554-471F134F838C

##### Notes

Paul et al. 2017

#### Pelagodes / Thalassodes sp.

AB9646F8-2608-5BDD-8D5A-5C5DFF7EEDA1

##### Notes

Present study

#### Pingasa dispensata

(Walker, 1860)

AD7A7ABC-0AB5-590B-987B-ADA19A0F3795

##### Notes

Present study

#### Thalassodes quadraria

(Guenee, 1857)

54FDE17C-3698-5064-80F8-EB553BA85082

##### Notes

Paul et al. 2017

#### Thalassodes sp. 1

885972EF-E7EA-5C00-85B2-4A00FB1F912C

##### Notes

Present study

#### Thalassodes sp. 2

1AD3895D-5DDA-55FE-8AD7-E2272319B374

##### Notes

Present study

#### Eupithecia ultimaria

Boisduval, 1840

B599464E-D4F1-5D15-87AD-19C857634664

##### Notes

Present study; Fig. 7a

#### Pasiphila rectangulata

(Linnaeus, 1758)

F5F7DDD0-4CD6-5C2A-9FE8-B46F7A30F692

##### Notes

Present study

#### Craspediopsis sp.

38429F97-FBD9-5A22-81CA-26BB69B16978

##### Notes

Present study; Fig. 7b

#### Haematopis grataria

(Fabricius, 1798)

1C1EFF93-BC73-5B17-8758-265A85594167

##### Notes

Inaturalist

#### Problepsis vulgaris

Butler, 1889

A65FF609-E71F-518C-88D0-2636C81C1D8B

##### Notes

Present study

#### Scopula nesciaria

(Walker, 1861)

4C72CCB7-3AD2-5AEA-87B3-0A4AEF7BE682

##### Notes

Present study; Fig. 7c

#### Scopula relictata

(Walker, 1866)

CD2E5BD7-FA6D-525D-9248-51F486C3E5CB

##### Notes

Present study; Fig. 7d

#### Scopula subpunctaria

(Herrich-Schäffer, 1847)

C4223D70-39A9-50B6-A32A-3E4EC62E434C

##### Notes

Present study

#### Scopula sp. 1

19DEBE4C-7867-5321-8F9B-C0A911E05AE7

##### Notes

Paul et al. 2017

#### Scopula sp.

3EC45BD3-2CDB-5827-9409-9518ADFE88D5

##### Notes

Present study; Fig. 7e

#### Traminda mundissima

(Walker, 1861)

1091BDD2-A475-568F-AC08-83E492D1414F

##### Notes

Paul et al. 2017; Fig. 7f

#### Phazaca theclata

(Guenée, 1858)

8F722A77-8008-5448-AB27-566A36592D6B

##### Notes

Present study; Fig. 8a

#### Acrocercops phaeomorpha

Meyrick, 1919

3C30BE90-33C9-5B43-9749-03740B3B54E5

##### Notes

Present study; Fig. 8b

#### Acrocercops trissoptila

Meyrick, 1921

6652296E-D087-5207-A773-C4B331EF984C

##### Notes

Present study; Fig. 8c

#### Caloptilia soyella

(van Deventer, 1904)

94516A48-99A2-5EF7-8E63-5CEC9519C5F1

##### Notes

Present study; Fig. 8d

#### Euspilapteryx sp.

7DDD8884-4078-5AC3-A217-4C5F6F3ABAA5

##### Notes

Present study; Fig. 8e

#### Phyllocnistis citrella

Stainton, 1856

34D4CB9B-4FEE-5835-9D94-4F38AE6E75EA

##### Notes

Present study; Fig. 8f

#### Chilena similis

Walker, 1855

590B49E7-548D-59D7-B8DB-562D6CB58FD4

##### Notes

Present study

#### Chilena strigula

Walker, 1865

60023587-3223-543E-BD53-3856C9EC7763

##### Notes

Present study

#### Kunugia latipennis

(Walker, 1855)

BCA773C2-E86F-5BCF-9414-665E967F9699

##### Notes

Present study; Fig. 9a

#### Streblote dorsalis

(Walker, 1866)

5CAFC541-F3E3-57B1-A2DB-0C05D30893D5

##### Notes

Present study

#### Streblote siva

(Lefèbvre, 1827)

AC41065E-E68B-5714-A02A-2DAF7858AF24

##### Notes

Present study

#### Streblote sp.

FC85B354-AB44-58FB-BBAD-8AAB85531CD8

##### Notes

Present study; Fig. 9b

#### Trabala vishnou

(Lefebvre, 1827)

EADF00C2-B240-5026-9202-FA8CB87EB2E4

##### Notes

Paul et al. 2017

#### Hyblaea puera

(Cramer, 1777)

7EBFB117-3168-53D5-BAD7-FAE812975D9D

##### Notes

Present study; Fig. 9c

#### Asota caricae

(Fabricius, 1775)

9F846E53-9ACB-5E44-8396-5A1C05BDEF26

##### Notes

Present study; Fig. 9d

#### Asota ficus

(Fabricius, 1775)

C786B8CA-0758-504F-8FBA-2E44A7187B88

##### Notes

Paul et al. 2017, Present study; Fig. 9e

#### Digama hearseyana

Moore ,1859

9913CC13-1DB9-5205-BB81-D2CE67A99573

##### Notes

Paul et al. 2017, Present study; Fig. 9f

#### Plecoptera reflexa

Guenée, 1852

9EA0DE89-47D1-5F5B-8466-393BE56FBF50

##### Notes

Present study; Fig. 10a

#### Agylla pallens

(Hampson, 1894)

2B83C427-4797-5F07-A63D-DCFF8DB0BEB0

##### Notes

Present study; Fig. 10b

#### Aloa lactinea

(Cramer, 1777)

AAE06A60-40D3-5C5C-A426-E1EAA081613B

##### Notes

Present study

#### Aloa lineola

(Fabricius, 1793)

58C6DA28-55A8-5B55-ACF2-38C5470860A2

##### Notes

Present study

#### Aloa moorei

(Butler, 1875)

C38CF058-07DC-5A11-BE71-DC4A1950FB4D

##### Notes

Rajesh et al. 2012, Present study; Fig. 10c

#### Amata cyssea

Stoll, 1782

F87F752D-40F0-55D7-9973-9E96164780F2

##### Notes

Paul et al. 2017, Present study; Fig. 10d

#### Amata sperbius

(Fabricius, 1787)

065D8666-B7EA-5AC5-B1D8-8BB91DEDC467

##### Notes

Present study; Fig. 10e

#### Argina astrea

(Drury, 1773)

4AF44590-3BA9-5359-8F1B-02224A038130

##### Notes

Paul et al. 2017

#### Creatonotos gangis

(Linnaeus, 1763)

2733B22B-D6FC-5E6C-8F81-AF3850558918

##### Notes

Paul et al. 2017, Present study; Fig. 10f

#### Eressa confinis

(Walker, 1854)

3102A7B6-1D77-5754-BB4D-166FD4CABF95

##### Notes

Present study; Fig. 11a

#### Mangina syringa

(Cramer, 1775)

21BE20A0-02D8-5E66-AC46-1083B4442824

##### Notes

Present study; Fig. 11b

#### Olepa ricini

(Fabricius, 1775)

74D7B405-FC15-5A36-961A-58814F924B75

##### Notes

Kumar et al. 2012, Present study

#### Psichotoe duvaucelii

Boisduval, 1829

6E15E1C4-4CEB-54BE-92BB-39F53AA1C4B5

##### Notes

Present study; Fig. 11c

#### Spilosoma obliqua

(Walker, 1855)

8C4FD71D-082D-5257-B5D0-2BC39872F389

##### Notes

Kumar et al. 2012, Present study;Fig. 11d

#### Utethesia pulchella

(Linnaeus, 1758)

33DA876D-11CC-57C6-A18A-93FF15A70796

##### Notes

Paul et al. 2017, Present study; Fig. 11e

#### Autoba olivacea

Walker, [1858]

C81EE91E-B8B3-5AA3-A806-D820EF69DF9B

##### Notes

Present study; Fig. 11f

#### Autoba silicula

Swinhoe, 1897

0080B09C-A876-5BE6-B245-2608E490026B

##### Notes

Present study; Fig. 12a

#### Hiccoda nigripalpis

(Walker, 1866)

1505903A-31EB-5FF8-8B2A-826F4A23BDF0

##### Notes

Present study; Fig. 12b

#### Metachrostis badia

Swinhoe, 1886

48C8FDA9-8835-54BE-96C5-09D483CD998D

##### Notes

Paul et al. 2017

#### Eudocima materna

(Linnaeus, 1767)

9FEEAF1F-B2B0-5B3A-8CB7-331E8D51910C

##### Notes

Present study

#### Eudocima phalonia

(Linnaeus, 1763)

AA321002-3E6A-5134-9692-CDFAA74DBD77

##### Notes

Present study; Fig. 12c

#### Lyncestis amphix

(Cramer, 1777)

3AA879C0-59A5-51A7-BDFA-63ED52D4CDC5

##### Notes

Present study; Fig. 12d

#### Oraesia emarginata

(Fabricius, 1794)

2937E017-7A7A-544C-90F6-B628200282AF

##### Notes

Paul et al. 2017

#### Oraesia cf. emarginata

(Fabricius, 1794)

C95397BB-BFA2-5714-BC8F-01CD45F69FD6

##### Notes

Present study; Fig. 12e

#### Polydesma umbricola

Boisduval, 1833

BC7BD69B-57A1-5F07-8F80-D9653017D447

##### Notes

Present study; Fig. 12f

#### Acantholipes circumdata

Walker, 1858

7DA8C5CE-450D-5400-B285-D6BD516C49DD

##### Notes

Present study

#### Achaea janata

(Linnaeus, 1758)

D31A897E-0803-5F8B-9FE7-A0106AEB374F

##### Notes

Paul et al. 2017, Present study; Fig. 13a

#### Antarchaea cucullata

Moore, 1885

E60DD811-B27B-5E3D-AA00-494550CAD39F

##### Notes

Present study

#### Attatha ino

(Drury, 1782)

3BB9F65A-6A95-5F19-9C5C-F1EE255E77E8

##### Notes

Paul et al. 2017; Fig. 13b

#### Attatha regalis

(Moore, 1872)

49E5C08C-70EA-575B-BF4E-60C9B0E60191

##### Notes

Present study

#### Bastilla crameri

(Moore, 1885)

94ABB5FE-F926-5347-BBD8-98125E87848E

##### Notes

Present study

#### Bastilla joviania

(Stoll, 1782)

9CCE5EBE-D79F-50B4-95FE-6A8E2131B542

##### Notes

Present study; Fig. 13c

#### Buzara onelia

(Guenée, 1852)

ADFD18B9-A67E-59B7-BAD0-E6F0EF8AC2A7

##### Notes

Present study; Fig. 13d

#### Dysgonia crameri

(Moore, 1885)

0D2CE769-5A94-5502-B489-23E2E26C3C8F

##### Notes

Paul et al. 2017

#### Dysgonia nr. torrida

(Guenee, 1852)

2FE80C93-3E38-57A4-B385-FB529FEAACB6

##### Notes

Paul et al. 2017; Fig. 13e

#### Entomogramma torsa

Guenée, 1852

F8C8F3A3-60A9-5A1E-8061-357B7DA5D4FB

##### Notes

Present study; Fig. 13f

#### Erebus macrops

(Linnaeus, 1768)

DA5F790B-F8DF-538A-8487-9E897E225D20

##### Notes

Present study; Fig. 14a

#### Ericeia inangulata

(Guenée, 1852)

78BC73D7-1EE3-5534-B240-93A9EBC8CE29

##### Notes

Present study; Fig. 14b

#### Grammodes geometrica

(Fabricius, 1775)

5F211242-4C4A-5CF9-8906-2F22EAB25DCB

##### Notes

Present study; Fig. 14c

#### Grammodes stolida

(Fabricius, 1775)

0E3F17AE-D706-599F-BAEF-4D3DFE447AD6

##### Notes

Present study; Fig. 14d

#### Mocis frugalis

(Fabricius, 1775)

9177DF6B-6619-519E-8F33-ACA14A5404CC

##### Notes

Present study; Fig. 14e

#### Mocis undata

(Fabricius, 1775)

A00D8AB4-744D-5363-A8F9-62B6E1DEFC53

##### Notes

Present study

#### Ophiusa triphaenoides

(Walker, 1858)

C280FA8F-7BD4-57E8-B51E-D1C848806FED

##### Notes

Paul et al. 2017

#### Ophisma gravata

Guenée, 1852

592239F0-5A3A-5F4D-9B97-DB2FDCE631FA

##### Notes

Present study; Fig. 14f

#### Pandesma anysa

Guenée, 1852

40579DED-6089-5EBF-B329-011D00BB79D8

##### Notes

Present study; Fig. 15a

#### Pandesma quenavadi

Guenée, 1852

009E036A-4553-5F2C-86E0-219685BF33C4

##### Notes

Present study; Fig. 15b

#### Pandesma sp.

AB9C3BD8-D231-5CFF-B820-9300BBAFD1E8

##### Notes

Paul et al. 2017

#### Pericyma albidens

(Walker, 1865)

D9745E62-F413-5A86-A6B4-F4987B5A783D

##### Notes

Present study; Fig. 15c

#### Pericyma glaucinans

(Guenée, 1852)

B97602F1-3DC1-5F6D-B035-DCD66968255C

##### Notes

Present study; Fig. 15d

#### Pericyma umbrina

(Guenée, 1852)

144E317D-A59B-5E2D-A013-32697CF7A2AC

##### Notes

Present study; Fig. 15e

#### Sphingomorpha chlorea

(Cramer, 1777)

2993D2D2-2660-5700-951F-F0436B79D6AB

##### Notes

Present study

#### Spirama helicina

(Hubner, 1831)

1A085AC5-B818-5404-A74D-E04465D6B580

##### Notes

Paul et al. 2017, Present study

#### Spirama retorta

(Clerk,1764)

81E3B5BF-6C5F-5964-806C-4209D7AF4683

##### Notes

Paul et al. 2017

#### Spirama sp.

686B5590-8EBD-575E-9C82-8E44FC18E890

##### Notes

Present study; Fig. 15f

#### Tathorhynchus exsiccata

(Lederer, 1855)

85E7384E-4731-5A82-BBCA-A9D609A732F3

##### Notes

Present study; Fig. 16a

#### Thyas coronata

(Fabricius, 1775)

7ED66BF6-90A7-5448-95E5-F89BD1635D78

##### Notes

Present study

#### Trigonodes hyppasia

(Cramer, 1779)

959CA6A4-08C7-54E2-B4D3-AA48C8912BC8

##### Notes

Paul et al. 2017; Fig. 16b

#### Eublemma anachoresis

(Wallengren, 1863)

01F6746E-CA9D-525F-B37B-4461B2426A57

##### Notes

Paul et al. 2017; Fig. 16c

#### Eublemma bifasciata

(Moore, 1881)

EC79E0C7-3EE0-530C-ABF5-E119D22909DE

##### Notes

Present study; Fig. 16d

#### Eublemma cochylioides

(Guenée, 1852)

6BDAAADB-2D67-581B-BE1B-A6F51DF129D9

##### Notes

Present study; Fig. 16e

#### Eublemma parva

(Hübner, 1808)

EE49309F-0F0E-54E2-B7A3-AFA531D19291

##### Notes

Present study; Fig. 16f

#### Eublemma roseana

(Moore, 1881)

09B93434-8B9B-5814-B2F7-448FFB9DF00A

##### Notes

Present study; Fig. 17a

#### Eublemma scitula

(Rambur, 1833)

962AAA0B-A2F3-54E1-B2AE-4B9947C31BEE

##### Notes

Present study; Fig. 17b

#### Anticarsia irrorata

(Fabricius, 1781)

7E616910-7C38-5EC7-A780-E17F2AEB15B2

##### Notes

Present study; Fig. 17c

#### Hydrillodes lentalis

(Guenee, 1854)

0C76134F-02F7-5DE2-8FBC-80CA827E9925

##### Notes

Present study; Fig. 17d

#### Nodaria cingala

Moore, [1885]

50529B23-E7BD-50CF-9F37-417A10794737

##### Notes

Present study; Fig. 17e

#### Hypena laceratalis

Walker, 1859

2E4CE5E5-2723-5C53-80AB-21957A109720

##### Notes

Present study; Fig. 17f

#### Hypena peruvialis

Schaus, 1904

BB070E63-B22B-5196-A15C-BD5C1DF848E4

##### Notes

Present study

#### Hypena sp.

EBFAD9FE-0B79-5160-9A84-82689E8A7E86

##### Notes

Paul et al., 2017

#### Rhynchina obliqualis

(Kollar, 1844)

55946889-E675-578A-A653-88B97FC5BF4E

##### Notes

Present study

#### Rhynchina xylina

Swinhoe, 1886

DE3AF907-76EF-5CEE-BA92-EE76CA314595

##### Notes

Present study

#### Euproctis cervina

(Moore, 1877)

48974901-24D8-563B-AD2D-010B2E10C85A

##### Notes

Present study;Fig. 18a

#### Euproctis fraterna

(Moore, [1883])

E3C122B7-D745-5B4B-B853-BECCAB7A2B1A

##### Notes

Present study; Fig. 18b

#### Euproctis lunata

Walker, 1855

90597B05-70FA-5B80-93A5-D23190DB4C4C

##### Notes

Paul et al. 2017

#### Euproctis lutea

(Fabricius, 1775)

D3C53F1D-8685-53B8-91BE-B463F23B31D9

##### Notes

Present study

#### Euproctis similis

(Füssli, 1775)

AA91867B-E679-5FF3-BA6E-CBF81C2ED013

##### Notes

Present study

#### Euproctis varians

(Walker, 1855)

63FC5652-DD6D-5B12-B4B0-4F87CEA59DFC

##### Notes

Present study

#### Euproctis virguncula

Walker, 1855

725775B5-7A6C-53AD-A58D-9F730BA7C277

##### Notes

Present study

#### Euproctis xanthorrhoea

(Kollar, 1848)

EEBD7A46-00BC-5A9B-844D-F69122B78AC9

##### Notes

Present study; Fig. 18c

#### Euproctis sp. 1

E22B01A5-8C53-596F-87D8-EDD52A7F3DD2

##### Notes

Present study

#### Euproctis sp. 2

9AFF0707-A289-521D-BA5E-4587727C4600

##### Notes

Present study

#### Laelia testacea

(Walker, 1855)

B65AC02C-FFAD-56F8-9AC5-DA25CC11E6BA

##### Notes

Present study; Fig. 18d

#### Lymantria sp.

630E0EAE-BA49-515D-A84C-841330BF2C7F

##### Notes

Paul et al. 2017

#### Olene mendosa

(Hübner, 1823)

6B6BE7FE-0E55-572E-9723-9A0B997932DC

##### Notes

Present study; Fig. 18e

#### Orvasca subnotata

Walker, 1865

0B0AAA30-BE57-55AC-9F03-2CFC27942FFC

##### Notes

Present study

#### Psalis pennatula

(Fabricius, 1793)

419358E0-D1DA-5972-B7C1-F1217E0BA215

##### Notes

Present study

#### Somena scintillans

(Walker, 1856)

9230472D-29DA-57D1-AEBC-7E11C565A0B4

##### Notes

Kumar et al. 2012, Present study

#### Anomis flava

(Fabricius, 1775)

71B6C052-E099-5A5E-8C62-8ABC73999C28

##### Notes

Paul et al. 2017; Fig. 18f

#### Anomis involuta

(Walker, [1858])

055309D9-5EF7-5C5F-AF1F-B0D5CAC4F67A

##### Notes

Present study; Fig. 19a

#### Syntomoides imaon

(Cramer, 1780)

0B928708-E2AD-51C5-B0A6-2E25DB21FBDC

##### Notes

Present study; Fig. 19b

#### Calesia haemorrhoa

Guenée, 1852

C3052EDF-9C10-5EB1-BAE8-99C30C2B3D1D

##### Notes

Present study; Fig. 19c

#### Anumeta atrosignata

Walker, 1858

1C0D52A5-74C2-5DD5-89CC-6EED6495BE26

##### Notes

Present study; Fig. 19d

#### Chlumetia transversa

(Walker, 1863)

F326998E-9468-5713-8DC4-EDDADADE91FF

##### Notes

Present study

#### Acontia basifera

Walker, [1858]

71D1E8F7-6CED-532E-A46A-AC203D805323

##### Notes

Present study

#### Acontia catenula

(Walker, 1865)

94C1284E-BB9C-5602-9172-17313F60F073

##### Notes

Present study; Fig. 19e

#### Acontia lucida

(Hufnagel, 1766)

64D6C931-76DB-5022-9030-F561EA4AA95F

##### Notes

Paul et al. 2017; Fig. 19f

#### Acontia marmoralis

(Fabricius, 1794)

3A24B3AE-235A-59EA-964E-283F715141FA

##### Notes

Present study; Fig. 20a

#### Acontia notabilis

(Walker, 1857)

E6588E7C-AA7B-5D40-965B-8C426BAE7A25

##### Notes

Present study; Fig. 20b

#### Acontia opalinoides

Guenée, 1852

0853EE8C-58BF-5812-B388-5335AE2F5340

##### Notes

Present study

#### Acontia sexpunctata

(Fabricius, 1794)

EA4070AD-BA95-594F-ABB4-9777A7C63B3C

##### Notes

Present study; Fig. 20c

#### Emmelia lunana

(Fabricius, 1794)

F52B88B3-40F2-5A50-A920-B7343DC0DE82

##### Notes

Present study

#### Emmelia semipallida

(Warren, 1913)

4415DAD6-9B2B-5BD1-A877-BCD71E7F03C1

##### Notes

Present study; Fig. 20d

#### Athetis bremusa

(Swinhoe, 1885)

C5948D54-CA16-5715-8B0F-12FF8D65A662

##### Notes

Present study

#### Athetis obtusa

(Hampson, 1891)

8F58DBFA-3746-5CD1-B2BE-4F28B4A03905

##### Notes

Present study

#### Athetis placida

(Moore, [1884])

1B3A1296-2931-531A-9C59-08501096192B

##### Notes

Present study

#### Aedia leucomelas

(Linnaeus, 1758)

B6AAB46A-40A5-5E66-876D-174853D0ACD0

##### Notes

Present study

#### Aegocera venulia

(Cramer, [1777])

537A1270-5766-5EE6-BC31-E0C5994D67BB

##### Notes

Present study; Fig. 20e

#### Matopo selecta

(Walker, 1865)

58A047E7-DC9C-527E-91C8-D292422E054E

##### Notes

Present study; Fig. 20f

#### Amyna axis

(Guenée, 1852)

88BD48DE-7D57-5052-898A-86B72956EC32

##### Notes

Present study; Fig. 21a

#### Perigea galaxia

Butler, 1883

5F09C105-55C4-55BF-8F25-D7C15B7E7132

##### Notes

Present study; Fig. 21b

#### Cretonia vegetus

(Swinhoe, 1885)

6B98D522-0584-53D9-919C-579D76BDD551

##### Notes

Present study

#### Deltote marginata

(Walker, 1866)

CAC17DC8-8F5F-5529-96EC-A8C4CDA306F8

##### Notes

Present study; Fig. 21c

#### Maliattha signifera

(Walker, [1858])

95E5110F-7067-56A0-838F-5039C3B63F22

##### Notes

Inaturalist; Fig. 21d

#### Ozarba mallarba

Swinhoe, 1885

C4C381AD-6BDE-571B-8835-ABD81B786093

##### Notes

Present study; Fig. 21e

#### Ozarba punctigera

Walker, 1865

A1DEC3D2-5F16-5158-98A3-DD27CE327D22

##### Notes

Present study; Fig. 21f

#### Ozarba rufula

Hampson, 1910

21FB5E92-B764-562B-8C5C-1940B6DF5F9D

##### Notes

Present study; Fig. 22a

#### Ozarba venata

Butler, 1889

DDCC78A1-2E63-5DE1-AAE8-C5AB922E4648

##### Notes

Present study

#### Adisura atkinsoni

Moore, 1881

B944E0D4-193F-5091-8188-BCCE9B453E09

##### Notes

Kumar et al. 2012, Present study

#### Helicoverpa armigera

(Hubner, 1809)

70068DC2-2731-595A-9351-CA2C727FCBD8

##### Notes

Kumar et al. 2012, Paul et al. 2017; Fig. 22b

#### Helicoverpa assulta

(Guenée, 1852)

A545365C-A96A-5D2C-A567-A20B9B4FF1A6

##### Notes

Paul et al. 2017; Fig. 22c

#### Heliothis peltigera

(Denis & Schiffermüller, 1775)

6E51A493-61F0-5935-BDB8-18679DCC963D

##### Notes

Paul et al. 2017, Present study; Fig. 22d

#### Agrotis ipsilon

(Hufnagel, 1766)

A52D2159-4955-5C22-92E9-27277569586D

##### Notes

Kumar et al. 2012, Paul et al. 2017, Present study; Fig. 22e

#### Agrotis segetum

(Denis & Schiffermüller, 1775)

DAFE1D2B-B916-5E31-99BD-3DA1199F1B8F

##### Notes

Kumar et al. 2012, Present study; Fig. 22f

#### Agrotis spinifera

(Hübner, 1808)

DCAA05B7-30EB-5913-94AF-D32633597C52

##### Notes

Present study; Fig. 23a

#### Dichagyris flammatra

(Schiffermuller, 1775)

EC0146E9-292D-5D64-B91F-84A78EA32F6F

##### Notes

Paul et al. 2017; Fig. 23b

#### Leucania comma

(Linnaeus, 1761)

94910D7C-22E5-5940-A784-1B7E72C3EF22

##### Notes

Present study; Fig. 23c

#### Leucania irregularis

(Walker, 1857)

86EBFBFF-865A-5D63-AD51-2E678A0AE1DF

##### Notes

Present study

#### Leucania loreyi

(Duponchel, 1827)

D25FF037-F37E-5682-9959-8A21AC42AC9E

##### Notes

Paul et al. 2017

#### Mudaria cornifrons

Moore, 1893

075DE5BE-B8AF-56D8-AB9F-D01FBE142FB5

##### Notes

Present study; Fig. 23d

#### Mythimna separata

(Walker, 1865)

95D35280-80DC-5224-8DD6-7081136A0831

##### Notes

Paul et al. 2017; Fig. 23e

#### Polytela cliens

(Felder & Rogenhofer, 1874)

73D7D097-10AE-54A2-A419-5F3CF69D2617

##### Notes

Present study; Fig. 23f

#### Polytela gloriosae

(Fabricius, 1781)

8A83640C-31CF-5EDC-9D3C-087F224508CE

##### Notes

Present study

#### Sasunaga tenebrosa

(Moore, 1867)

EF603C32-6BA0-5954-B5E7-03C912876BA4

##### Notes

Present study; Fig. 24a

#### Sesamia uniformis

(Dudgeon, 1905)

32D82D2C-9491-52EA-8FFE-2098CF1DF120

##### Notes

Present study; Fig. 24b

#### Spodoptera exigua

(Hubner, 1808)

8E318A41-A3A7-543A-AB74-DE1098192356

##### Notes

Kumar et al. 2012, Paul et al. 2017, Present study; Fig. 24c

#### Spodoptera litura

(Fabricius, 1775)

400FE2C9-390B-5CFA-BF76-8FCEC0EEDF44

##### Notes

Kumar et al. 2012, Paul et al. 2017 Present study; Fig. 24d

#### Spodoptera pecten

Guenee, 1852

01609FC7-4F92-50ED-8D83-9ADC40E3F94E

##### Notes

Present study

#### Xestia sp.

(Hübner, 1790)

29823B7E-4C7C-5694-A231-74EAC531B637

##### Notes

Paul et al. 2017

#### Autographa nigrisigna

(Walker, 1857)

4556D4C4-B7ED-576E-82F2-F25FD00DD055

##### Notes

Kumar et al. 2012, Paul et al. 2017; Fig. 24e

#### Chrysodeixis acuta

(Doubleday, 1843)

149819C8-9607-5EF6-BA79-FABA7CC7929F

##### Notes

Kumar et al. 2012; Paul et al. 2017

#### Chrysodeixis chalcites

(Esper, 1789)

E4B82900-2170-5403-B016-7B3A89791F8F

##### Notes

Kumar et al. 2012; Paul et al. 2017; Fig. 24f

#### Chrysodeixis eriosoma

(Doubleday, 1843)

3993318E-23FA-5C28-83B1-89FE61228567

##### Notes

Paul et al. 2017, Present study; Fig. 25a

#### Ctenoplusia albostriata

(Bremer & Grey, 1853)

94452FF8-169E-5EA4-9DB2-DA85D429F5FF

##### Notes

Paul et al. 2017, Present study

#### Erythroplusia pyropia

(Butler, 1879)

AA2CC9AF-C5FD-54E6-9925-68480848AACF

##### Notes

Paul et al. 2017

#### Thysanoplusia daubei

(Boisduval, 1840)

34AE02E4-FFF3-5BF5-A1D4-2B5557CF3E6F

##### Notes

Paul et al. 2017

#### Thysanoplusia orichalcea

(Fabricius, 1775)

66D5CE69-1D98-5023-8721-252B43D457EB

##### Notes

Paul et al. 2017; Present study; Fig. 25b

#### Trichoplusia ni

(Hubner, 1803)

AA4210AB-FE6C-5DF5-A18D-4A019E41A79D

##### Notes

Kumar et al. 2012, Present study; Fig. 25c

#### Arcyophora dentula

(Lederer, 1869)

18B16718-132E-5654-B2A4-B8039E4DF172

##### Notes

Present study; Fig. 25d

#### Aquis orbicularis

(Walker, 1858)

EAB05AA3-C4F6-5CD1-8AB6-186DDFC94591

##### Notes

Present study

#### Carea angulata

(Fabricius, 1793)

630DB011-FA49-5056-8882-97030439D39C

##### Notes

Present study; Fig. 25e

#### Earias cupreoviridis

(Walker, 1862)

8D246DF2-326D-5C10-8DC0-AE2ED4D08B6F

##### Notes

Present study; Fig. 25f

#### Earias insulana

(Boisduval, 1833)

57039E33-0BB6-52F7-93BC-7AB2690AF914

##### Notes

Kumar et al. 2012, Paul et al. 2017, Fig. 26a

#### Earias vittella

(Fabricius, 1794)

D8BB28FF-5184-5102-8C0F-F0DFF85D9B4F

##### Notes

Kumar et al. 2012, Present study; Fig. 26b

#### Giaura sceptica

(Swinhoe, 1885)

2144F880-EAAA-5DDD-B07A-13AD1B444A2C

##### Notes

Present study; Fig. 26c

#### Labanda semipars

(Walker, 1858)

91E09C98-1C45-58CC-8FFE-3876AFA0F095

##### Notes

Present study

#### Maurilia iconica

(Walker, 1857)

1D6E8DE0-D000-5984-BD75-72515A0961C7

##### Notes

Present study; Fig. 26d

#### Selepa celtis

Moore, [1858]

826E456A-D43A-5CB3-BFB3-A4D448538F83

##### Notes

Present study; Fig. 26e

#### Selepa docilis

Butler, 1881

43A39821-5B8E-526C-8AD7-A683E651609A

##### Notes

Present study; Fig. 26f

#### Evonima plagiola

(Hampson, 1898)

C22D34D4-9552-5274-B94D-502DC4E856B4

##### Notes

Present study; Fig. 27a

#### Meganola sp.

B1B10EBE-CB54-5F4B-BC30-E26A74DD18A9

##### Notes

Present study

#### Nola internella

(Walker, 1865)

3165B2DC-4B11-5DA8-B9F6-977A4A581121

##### Notes

Present study

#### Exelastis atomosa

(Walsingham, 1885)

078FEB07-4A3B-53AD-B781-A4BBDAE8BAB9

##### Notes

Kumar et al. 2012; Present study

#### Sphenarches caffer

(Zeller, 1852)

4FE28362-1273-5B4A-9DAB-32013961284C

##### Notes

Kumar et al. 2012; Present study; Fig. 27b

#### Sphenarches sp.

9E01FF6D-EF21-5C83-819B-D8452D2FEA0F

##### Notes

Present study

#### Elophila sp.

4E963DFA-1C52-593E-8ACF-A46272F35CD7

##### Notes

Present study

#### Parapoynx diminutalis

(Snellen, 1880)

3AC6B5EB-D1BD-5E48-9AAA-EC8930DF1ED3

##### Notes

Present study

#### Paraponyx fluctuosalis

(Meyrick, 1899)

93BE277E-CAB1-5DE9-B6B2-37D018DA991D

##### Notes

Present study

#### Chilo partellus

(Swinhoe, 1885)

34AD9109-AED5-5B63-B366-1413364609FC

##### Notes

Fig. 27c

#### Chilo suppressalis

(Walker, 1863)

2F3E4715-55AF-5D26-AAAC-68ECF2AFC40D

##### Notes

Present study

#### Hendecasis duplifascialis

(Hampson, 1891)

00EB3B6F-3667-52E5-8FC5-BED329B6D691

##### Notes

Present study

#### Ptychopseustis sp.

(Hampson, 1896)

0F2B20FF-34CE-534E-AA59-1271D1E499FA

##### Notes

Present study; Fig. 27d

#### Crocidolomia pavonana

(Fabricius, 1794)

458B0BBB-3F19-57CF-9698-FA7396B3DD2B

##### Notes

Kumar et al. 2012, Present study; Fig. 27e

#### Crocidolomia suffusalis

(Hampson, 1891)

4D67A97D-FC92-51C0-9002-8565E46D0ACF

##### Notes

Arora 2000

#### Hellula undalis

(Fabricius, 1794)

C4D40C6A-BD1A-5607-917B-E06E73837A7C

##### Notes

Kumar et al. 2012, Present study

#### Aporodes floralis

(Hübner, 1809)

F8EF8788-4B38-5FD3-A22F-47C4DEF894E1

##### Notes

Arora 2000, Present study; Fig. 27f

#### Autocharis fessalis

(Swinhoe, 1886)

C597FF7C-413B-51A2-A5E7-C93984600C3B

##### Notes

Present study; Fig. 28a

#### Tegostoma baphialis

(Staudinger, 1871)

CB469662-247B-5104-AB3B-6B77A6D47DB7

##### Notes

Present study

#### Tegostoma comparalis

(Hübner, 1796)

E5602AAA-0595-5539-A1C9-C4E6BC32407E

##### Notes

Present study

#### Hydriris ornatalis

(Duponchel, 1832)

06268BE2-1672-5FF6-BE4C-6C93B2BDADBF

##### Notes

Present study; Fig. 28b

#### Isocentris filalis

(Guenée, 1854)

4E7459EF-A475-52D3-8D09-61898C60A6F3

##### Notes

Present study

#### Pyrausta indistans

Moore, 1888

3B3FE776-688C-5F0E-9810-7E05D4D9C3E5

##### Notes

Gupta 1994, Present study; Fig. 28c

#### Pyrausta phoenicealis

(Hübner, 1818)

4D421788-9840-5801-BFCA-13A434F850D4

##### Notes

Present study

#### Spoladea recurvalis

(Fabricius, 1775)

BA930765-DA6C-585C-8CC0-526705E2EDE6

##### Notes

Kumar et al. 2012, Paul et al. 2017, Present study; Fig. 28d

#### Scirpophaga incertulas

(Walker, 1863)

7217A8DD-0A74-5FCF-8BC0-9AE16424A9DF

##### Notes

Fig. 28e

#### Scirpophaga nivella

(Fabricius, 1794)

2D8C3AE5-4827-5F87-BC9D-9CE3646314BA

##### Notes

Fig. 28f

#### Scirpophaga sp.

372B81A5-118A-5BAB-BE7C-4C37D258E811

##### Notes

Present study

#### Aethaloessa calidalis

(Guenée, 1854)

889884F9-12CF-51E4-8180-4D3728099B53

##### Notes

Present study

#### Antigastra catalaunalis

(Duponchel, 1833)

6EFFEA19-5656-5315-9584-AAAD9FB0BF74

##### Notes

Present study; Fig. 29a

#### Botyodes asialis

Guenée, 1854

BB3106F0-E720-557F-B00A-EF45BC7F4666

##### Notes

Present study

#### Botyodes diniasalis

(Walker, 1859)

AE337A42-BE66-5AED-8CEA-0A5FDB4C9D19

##### Notes

Paul et al. 2017; Fig. 29b

#### Botyodes sp.

CEDD204C-4ACB-5A5A-9B60-7694AA572458

##### Notes

Present study

#### Chabula acamasalis

(Walker, 1859)

BE4D7301-A8ED-53D6-9916-47D96DC58AF5

##### Notes

Present study

#### Cirrhochrista brizoalis

(Walker, 1859)

2324D32D-D6E5-56F8-93ED-9E6FEC3DE357

##### Notes

Present study; Fig. 29c

#### Cnaphalocrocis exigua

(Butler, 1879)

27658894-A014-5AD4-A655-04C777ACFA4C

##### Notes

Present study

#### Cnaphalocrocis medinalis

(Guenee, 1854)

522BB981-7DF4-5201-B95C-774DE969542B

##### Notes

Paul et al. 2017; Fig. 29d

#### Cnaphalocrocis trapezalis

(Guenée, 1854)

BF760AC3-81F6-53CA-909A-CFCF4700B11B

##### Notes

Inaturalist; Present study

#### Cnaphalocrocis sp.

Lederer, 1863

D07523B9-9BC8-512E-93EF-CE737804B4B2

##### Notes

Paul et al. 2017

#### Conogethes punctiferalis

(Guenée, 1854)

0970E2D2-4196-5B90-B181-59DAF33E6A46

##### Notes

Present study; Fig. 29eFig. 29f

#### Diaphania indica

(Saunders, 1851)

59A4B0A5-6621-5CEC-BC0C-C42B2E436EC4

##### Notes

Paul et al. 2017, Present study; Fig. 29f

#### Eurrhyparodes bracteolalis

(Zeller, 1852)

305412C8-8935-51F2-BC68-C4BB83D24D65

##### Notes

Present study; Fig. 30a

#### Eurrhyparodes tricoloralis

(Zeller, 1852)

10BBE712-DCEC-5CBA-85C2-1252C49A8315

##### Notes

Arora 2000, Present study

#### Gadessa nilusalis

(Walker, 1859)

E1CB21E3-C926-5635-A55F-F6DAB76A5C0E

##### Notes

Paul et al. 2017

#### Glyphodes onychinalis

Guenée, 1854

46B41D70-3B65-5EFC-887F-88BEA8EDFD86

##### Notes

Present study; Fig. 30b

#### Haritalodes derogata

(Fabricius, 1775)

7E2FCC95-C37A-54A2-B475-971BFB0E6A79

##### Notes

Kumar et al. 2012, Present study; Fig. 30c

#### Herpetogramma bipunctalis

(Fabricius, 1794)

BDC05AEB-7312-54AC-9618-5C32C634C6EA

##### Notes

Present study

#### Herpetogramma licarsisalis

(Walker, 1859)

E0058E62-2BF0-55E3-B53A-48D1F0E729E1

##### Notes

Inaturalist; Fig. 30d

#### Herpetogramma phaeopteralis

(Guenee, 1854)

DF89C720-4EAC-56FA-9223-D9BE1F4CF4E3

##### Notes

Present study

#### Herpetogramma stultalis

(Walker, 1859)

FA9F6022-B60F-5F6F-8DBD-0254E3AFB4B5

##### Notes

Present study

#### Leucinodes orbonalis

Guenée, 1854

8E7CB30D-D79E-5528-A0E7-29EA3967501D

##### Notes

Kumar et al. 2012, Inaturalist, Present study; Fig. 30e

#### Maruca vitrata

(Fabricius, 1787)

4056AE90-5384-5B96-96FB-F649AD10D8E0

##### Notes

Kumar et al. 2012, Paul et al. 2017, Present study; Fig. 30f

#### Nausinoe geometralis

(Guenée, 1854)

F2FDBDE5-8C6B-5025-B366-5E3452BE459D

##### Notes

Present study

#### Nausinoe perspectata

(Fabricius, 1775)

B45962A6-3D54-5941-9588-E974F09A4C86

##### Notes

Present study; Fig. 31a

#### Nomophila nearctica

Munroe, 1973

7B4F114C-7492-5BB9-A85C-E3D46D546B36

##### Notes

Present study

#### Nomophila noctuella

(Denis & Schiffermüller, 1775)

3F5C638C-A817-5F63-A9F2-32AC203387C1

##### Notes

Present study; Fig. 31b

#### Noorda blitealis

Walker, 1859

08083EFC-033F-588B-BE2D-A7B0C889107C

##### Notes

Present study

#### Notarcha aurolinealis

(Walker, 1859)

ADE53D47-DF65-5A66-8F72-75483AB6B8C0

##### Notes

Present study

#### Omiodes indicata

(Fabricius, 1775)

D65AD284-981F-5475-9558-B4E209C41DD9

##### Notes

Present study

#### Prorodes mimica

Swinhoe, 1894

47937437-78B4-5C12-8A9A-55B225025B78

##### Notes

Gupta 1994

#### Pygospila tyres

(Cramer, 1780)

3B83AF10-C627-5B43-A2F2-84954E8C998D

##### Notes

Present study; Fig. 31c

#### Sameodes cancellalis

(Zeller, 1852)

6DE640BA-EC3B-5365-8C1A-B63833DD80ED

##### Notes

Paul et al. 2017; Fig. 31d

#### Synclera traducalis

(Zeller, 1852)

1A7D7A08-424A-58F7-AC08-B49E49EF57A0

##### Notes

Mandal and Bhattacharya 1979

#### Achroia grisella

(Fabricius, 1794)

D6A0826B-7B28-524B-BA9F-2B904B2341EB

##### Notes

Present study

#### Corcyra cephalonica

(Stainton, 1866)

A69F195E-9B50-5AE4-B1F6-C443E22634AE

##### Notes

Arora 2000

#### Galleria mellonella

(Linnaeus, 1758)

4AAF5FAB-27ED-587D-8386-E016334C0244

##### Notes

Present study

#### Trachylepidia fructicassiella

Ragonot, 1887

D7D27179-BB38-5AF3-B8A1-356A4F41B072

##### Notes

Present study; Fig. 31e

#### Copamyntis infusella

(Meyrick, 1879)

28220B72-C44B-52D7-813B-64A20C974235

##### Notes

Arora 2000

#### Etiella zinckenella

(Treitschke, 1832)

2800FF0D-698B-59D7-B7E6-ACB007965A8B

##### Notes

Kumar et al. 2012, Present study; Fig. 31f

#### Euzophera perticella

Ragonot, 1888

2C5DAF0D-A3F4-5F06-B6FF-F068F7949807

##### Notes

Kumar et al. 2012, Present study

#### Nephopterix eugraphella

Ragonot, 1888

80D4CF50-C32D-5EF5-BF15-61944631BC17

##### Notes

Arora 2000

#### Phycita clientella

Zeller, 1867

86A2C04D-E0B7-554B-A7A4-E4DB98C59A6D

##### Notes

Arora 2000, Present study

#### Polyocha depressellus

(Swinhoe, 1885)

1C09F0FD-E00B-5EFF-9932-464AE3CE9C96

##### Notes

Arora 2000

#### Pristarthria akbarella

Ragonot, 1888

9C04E162-7F09-52C4-8CE5-9E6DDD1B2B74

##### Notes

Paul et al. 2017

#### Hypsopygia mauritialis

(Boisduval, 1833)

95EBEB0A-299D-55F0-9C97-8B03AAB74439

##### Notes

Present study; Fig. 32a

#### Acanthoclita balanoptycha

(Meyrick, 1910)

86F52545-5C7A-5EA7-A63B-F56890F67133

##### Notes

Present study; Fig. 32b

#### Dudua aprobola

(Meyrick, 1886)

A27FBD3A-77E0-5A6F-A879-9923D5C02F5F

##### Notes

Present study; Fig. 32c

#### Loboschiza koenigiana

(Fabricius, 1775)

CE343C4E-2F03-5E1F-8A43-5D6C3D9923BF

##### Notes

Present study; Fig. 32d

#### Syntozyga ephippias

(Meyrick, 1907)

CC357AB8-AF97-5A17-8F3D-5954041621A8

##### Notes

Present study; Fig. 32e

#### Typhonia autochthonia

(Meyrick, 1931)

93954CC1-9463-5C34-92AD-471A6CA83995

##### Notes

Present study

#### Leucoptera sphenograpta

Meyrick, 1911

D920391B-E874-5843-87ED-28D7056D837C

##### Notes

Present study

#### Plutella xylostella

(Linnaeus, 1758)

B0D48D0F-F840-5236-99D3-2491F84B8BA4

##### Notes

Kumar et al. 2012, Present study; Fig. 32f

#### Yponomeuta sp.

507BA2B8-E767-5D81-942A-AD60B2B6701B

##### Notes

Present study

#### Fulgoraecia melanoleuca

(Fletcher, 1939)

4102D411-30D8-5807-8B0E-A58FD9B1607B

##### Notes

Fig. 33a

#### Aergina hilaris

Meyrick, 1913

CF12A815-E161-5164-818F-BDCD03C7CC31

##### Notes

Present study; Fig. 33b

#### Altha nivea

Walker, 1862

B3580EA4-5F2B-5543-A281-D6B5F11E8019

##### Notes

Present study; Fig. 33c

#### Campylotes histrionicus

Westwood, 1839

1B89BDA3-B377-55DF-AE4F-DA19D399801D

##### Notes

Paul et al. 2017

## Analysis

The present study encompasses 338 moth species belonging to 32 different families pertaining to 14 superfamilies. Two hundred and thirty four species were added to the existing moth fauna of Delhi. Amongst the different superfamilies, the highest number of species were recorded in the superfamily Noctuoidea with 164 species accounting for about 48.5% of all the moths, followed by the superfamily Pyraloidea which constitutes about 20.4% of the moths and includes 69 species. The least number of species were observed in the superfamilies Cossoidea, Tineoidea and Hyblaeoidea comprising only one species each as shown in Table 1. Of the superfamilies, more familial diversity was exhibited by the superfamily Gelichioidea having species belonging to eight different families, followed by Noctuoidea representing four families. Amongst the different 32 families, the highest number of species (95) were recorded in Erebidae (Table 1). Noctuoidea is the most speciose superfamily recorded in current study with 164 species belonging to four families. In these four families, Erebidae was observed to be the largest family comprising 95 species which constitutes about more than half of the superfamily making up to 56.7%. Pyraloidea is the second largest superfamily in the current study with 69 species belonging to two families. Of the two families, Crambidae dominates with 57 species accounting for about 82.6% of Pyraloidea. All the data regarding the total number of species in different superfamilies and families are presented in Table 1.

## Discussion

In most parts of the world, the nocturnal Lepidoptera (such as Noctuoidea, Tortricoidea, Bombycoidea, Geometroidea, Pyraloidea, Yponomeutoidea and Gelechioidea) have received less attention than their more charismatic diurnal cousins, butterflies. However, as herbivores and a food supply as a prey for other insects, birds and bats (Vaughan 1997), these insects play an important ecological role and some of them are important pollinators of specific plant species (e.g. some Orchids and many members of Caryophyllaceae). Moth numbers have been declining dramatically in recent decades (Dennis et al. 2019). For example, macro-moth abundance decreased by 28% in the United Kingdom between 1968 and 2007 (Fox et al. 2013) and similar negative patterns have been observed in Sweden (Franzén and Johannesson 2007) and The Netherlands (Groenendijk and Ellis 2011). Due to the keystone importance of moths in many habitats, such losses are predicted to have cascading consequences at both higher (bats, birds) and lower (plants) trophic levels (Wickramasinghe et al. 2004). The present checklist is an accumulation of surveys in three locations in Delhi which include the Indian Agricultural Research Institute (IARI) campus, Rashtrapathi Bhawan and Asola Bhatti Wildlife Sanctuary and the study of moth specimens housed in NPC-IARI. In the current study, we have added 234 species to the checklist of moths of Delhi. This accounts for 338 species of moths belonging to 32 families of 14 different superfamilies. Many of the specimens studied from the museum collections were previously unidentified and those identified were not published. Interestingly, the identification in these collections was done by Mr. E. Meyrick (Microlepidoptera), Mr. T.B. Fletcher and Dr. S.L. Gupta (Macrolepidotpera and microlepidoptera). Post-independence, there were very few collection events and there is a discontinuity in moth monitoring and collections in Delhi, except for few agriculturally-important pests (Paul et al. 2016) and a few common moths (Paul et al. 2017). This may be because of: 1. Absence of continuous moth monitoring projects in Delhi; 2. Lack of training amongst researchers to collect and deposit moths in museums and 3. Most importantly, photographic identification of common moths has gained momentum during the last decade (Sondhi et al. 2021). The identification of many moth species is difficult with photographs alone and there is a need to collect and study them morphologically. In our study, we found that most of the moths from Delhi in the collections belong to macro-moth families. The under-representation of micro-moths is likely due to a lack of systematic collections and a lack of experts who study micro-moths. Additionally, our study documents more moth species associated with agricultural and horticultural habitat (e.g. *Helicoverpaarmigera*, *Spodopteralitura* etc.) likely due to extensive human-led landscaping in Delhi and also due to the greater survey effort in the IARI campus, which contains many agricultrual and horticultural research farms.

The paucity of baseline data, both in terms of abundance and diversity of moths, poses a significant hurdle in assessing the impact of various threats like land-use changes, rapid urbanisation, pollution, insecticides and global warming (Dennis et al. 2019) to insect diversity. According to a recent analysis (Sharma et al. 2020), unplanned urbanisation in Delhi that occurred between 1998 and 2018 led to Delhi's forest cover shrinking by half between 1998 and 2018, suggesting the need for development of conservation zones inside and adjacent to the capital, as well as increased interaction with urban citizens to create a better understanding of urban biodiversity. There are examples of the forested land converted into conserved sites, such as the Sanjay Van, Aravalli Biodiversity Parks and Asola Wildlife Sanctuary has assisted in sustaining the biodiversity to an appreciable extent. However, there have been no systematic studies on moth diversity till now in these locations and, given the rapid urban growth, more such sites are needed to prioritise the conservation efforts documenting available biodiversity and continuous monitoring is very important. We strongly recommend the setting up of a study site/sites for long term monitoring of insect populations and their diversity in the State of Delhi. The monitoring programme could be undertaken by public participation in biodiversity documentation involving citizens and it has been proven successful in certain nations (Miller-Rushing et al. 2012, Pocock et al. 2015).

In conclusion, we believe that there will still be many more species that can be added to the present list as moths are sampled more extensively and studied more intensively using modern techniques, such as DNA barcoding. However, our study helps to establish the first comprehensive preliminary dataset on moths of the region, which can be a spring-board for future well-planned moth recording in Delhi. The areas for future investigation include concentrating on developing comprehensive species inventory, studying larval host associations and evaluation and prioritising moth species for conservation.

## Figures and Tables

**Figure 1a.**
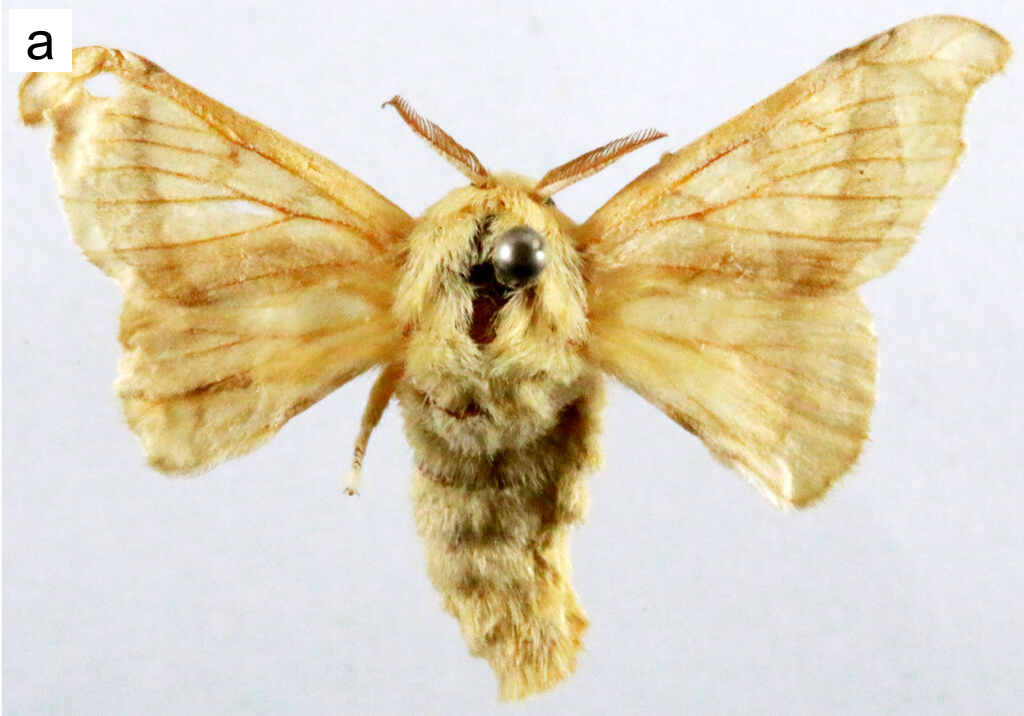
Bombyxmori

**Figure 1b.**
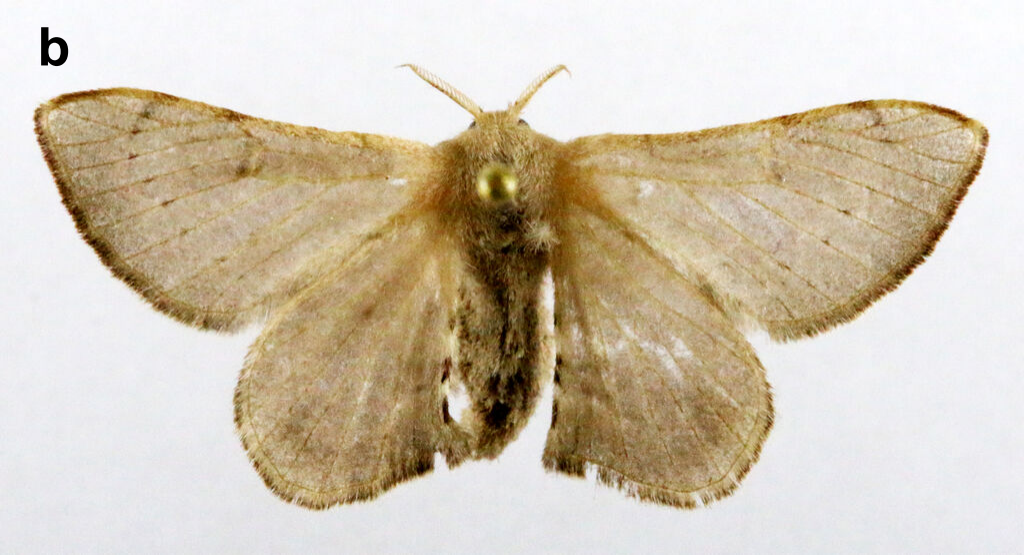
Trilochavarians

**Figure 1c.**
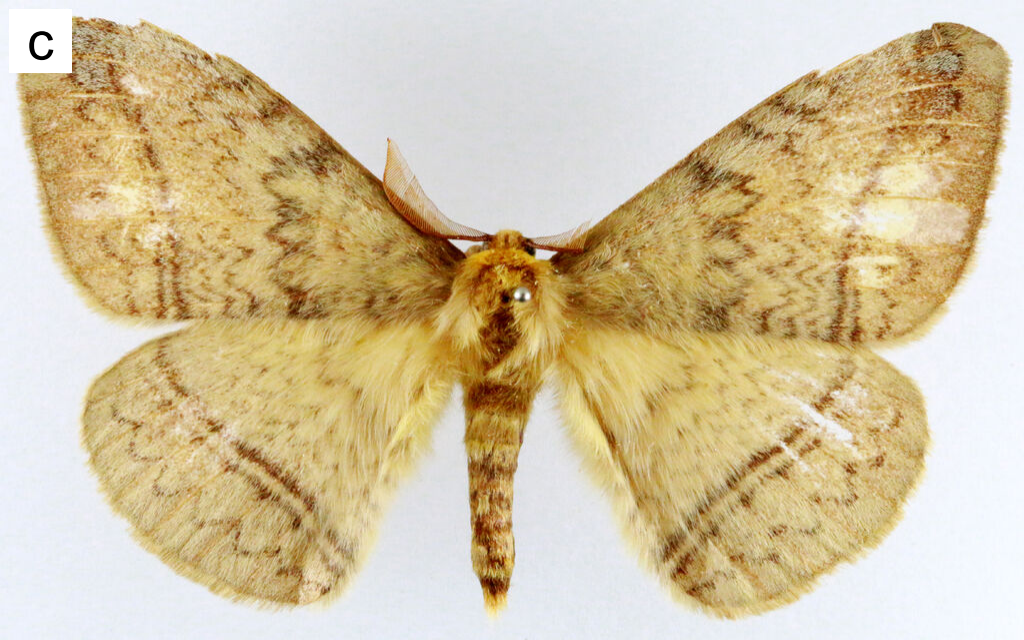
Eupteroteundata

**Figure 1d.**
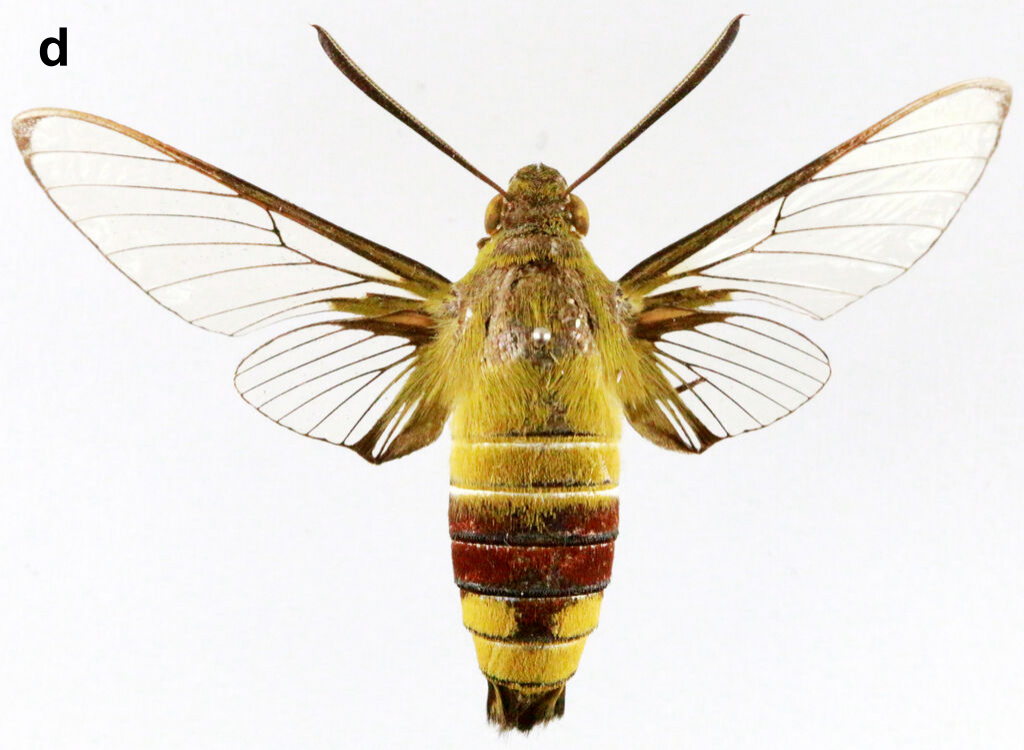
Cephonodeshylas

**Figure 1e.**
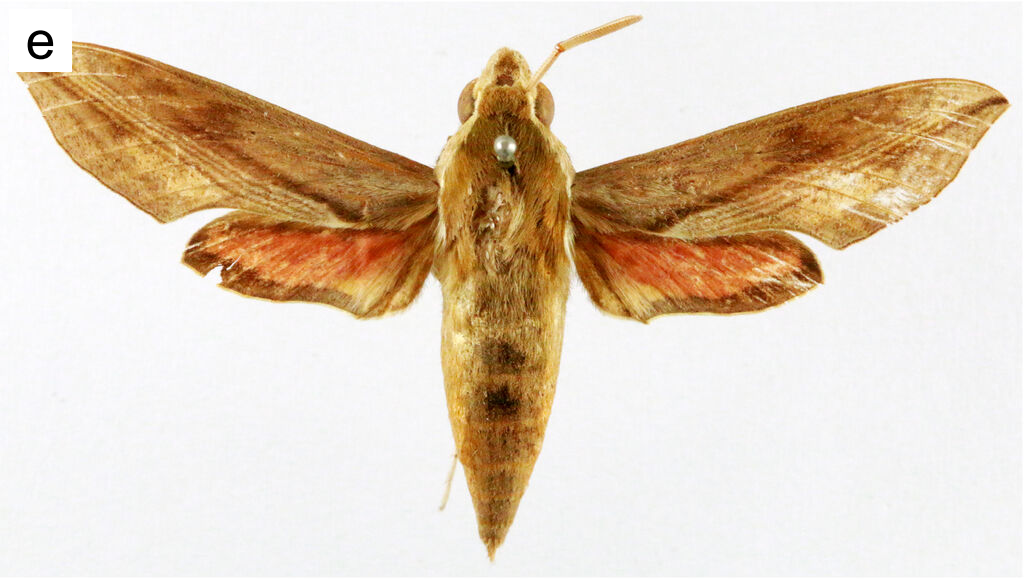
Hippotionboerhaviae

**Figure 1f.**
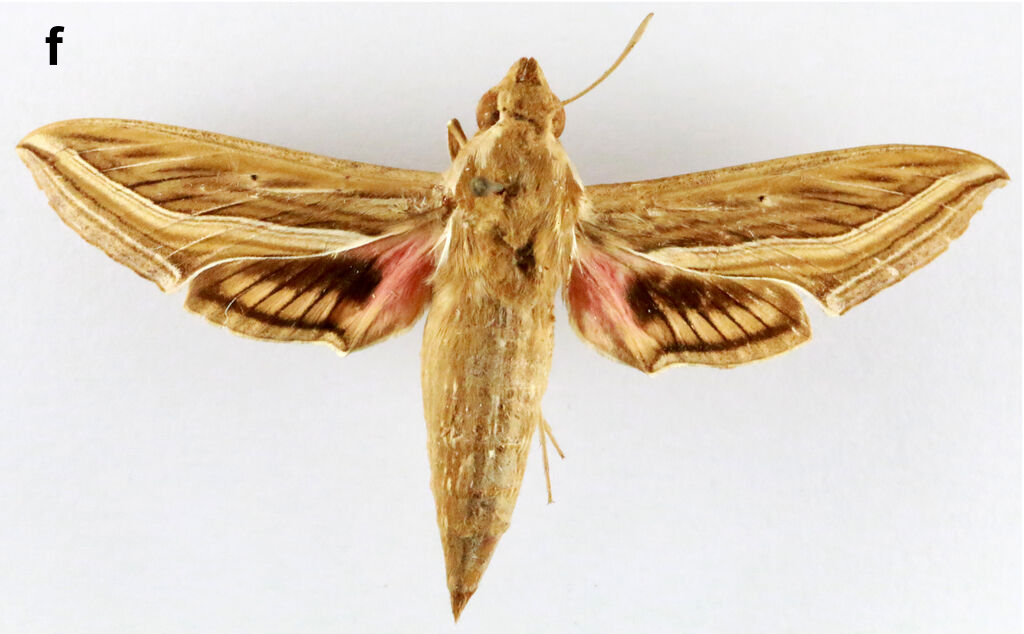
*Hippotioncelerio*.

**Figure 2a.**
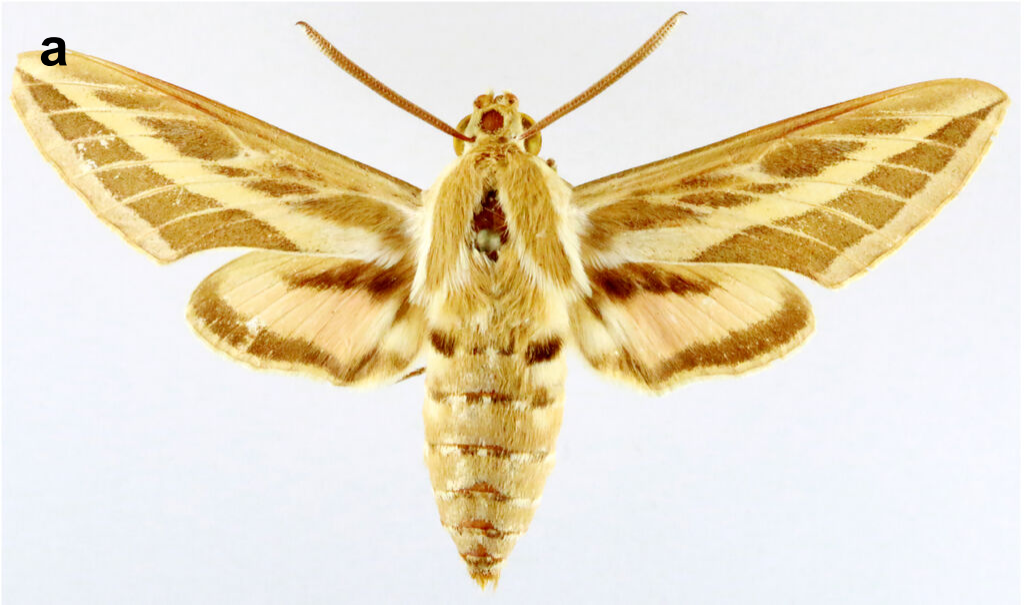
Hyleslineata

**Figure 2b.**
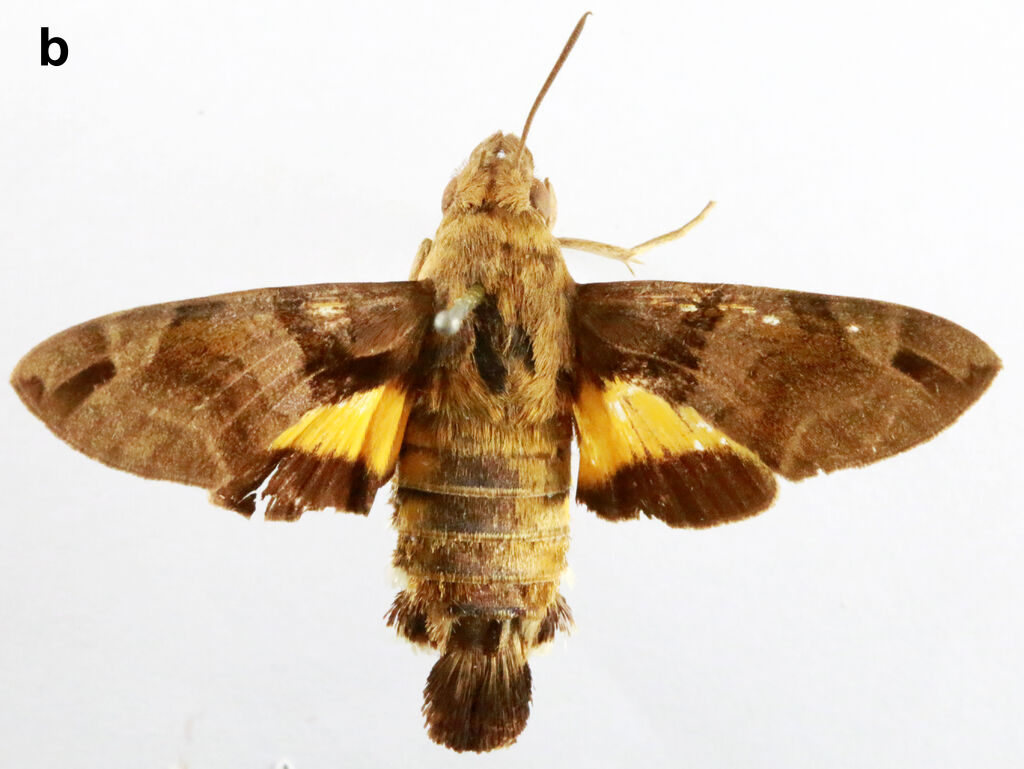
Macroglossumneotroglodytus

**Figure 2c.**
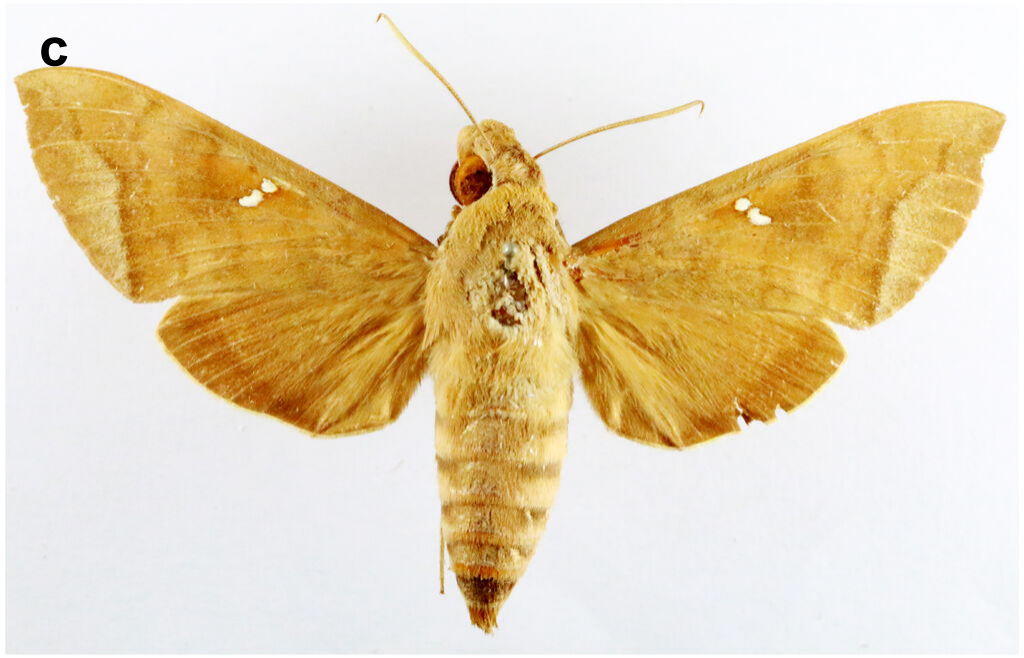
Nephelehespera

**Figure 2d.**
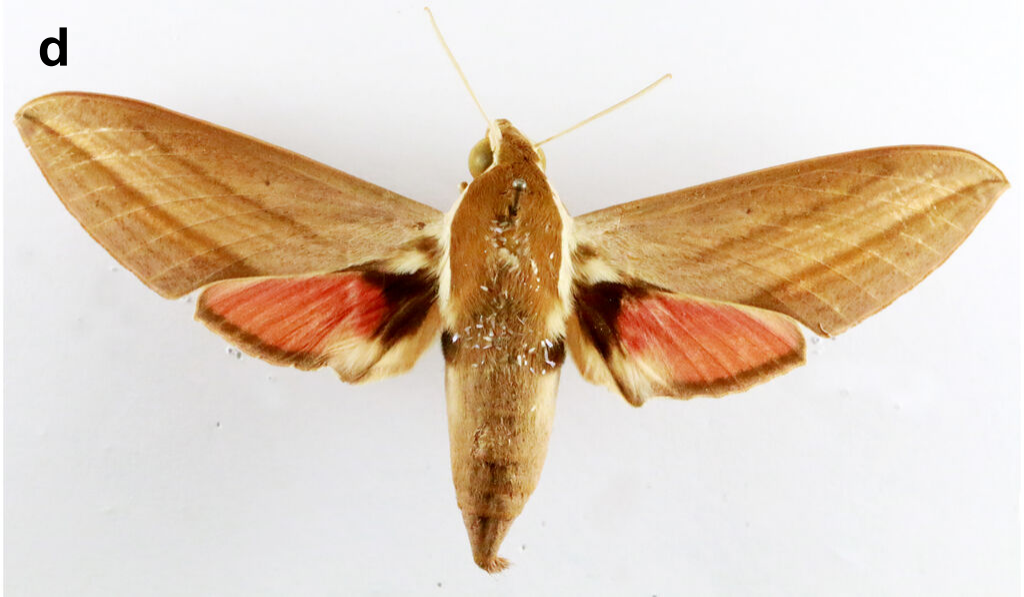
Theretraalecto

**Figure 2e.**
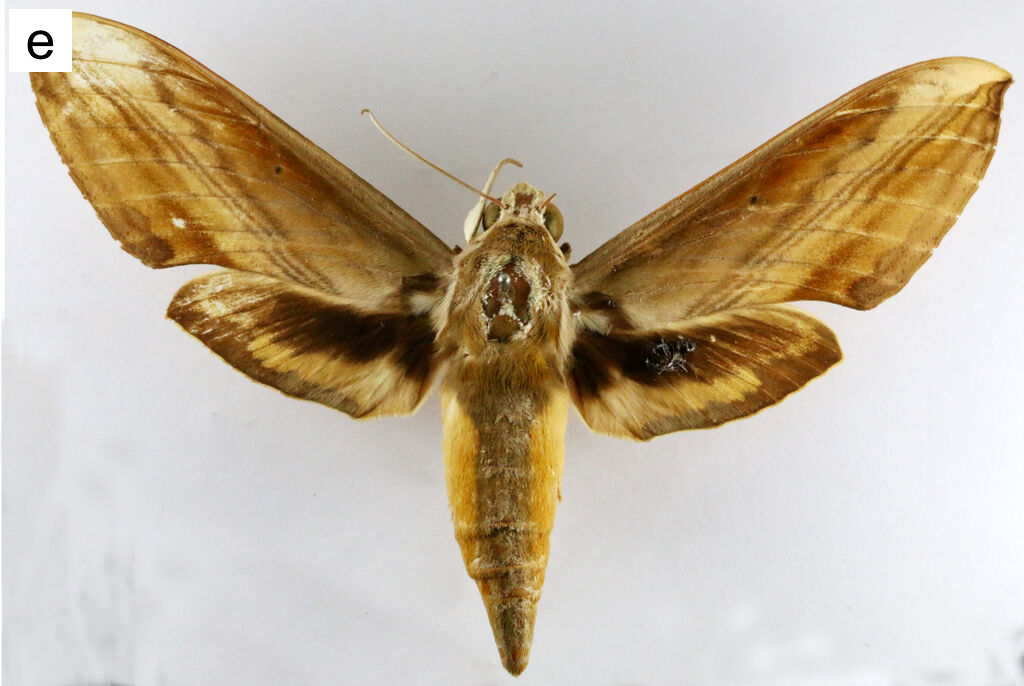
Theretranessus

**Figure 2f.**
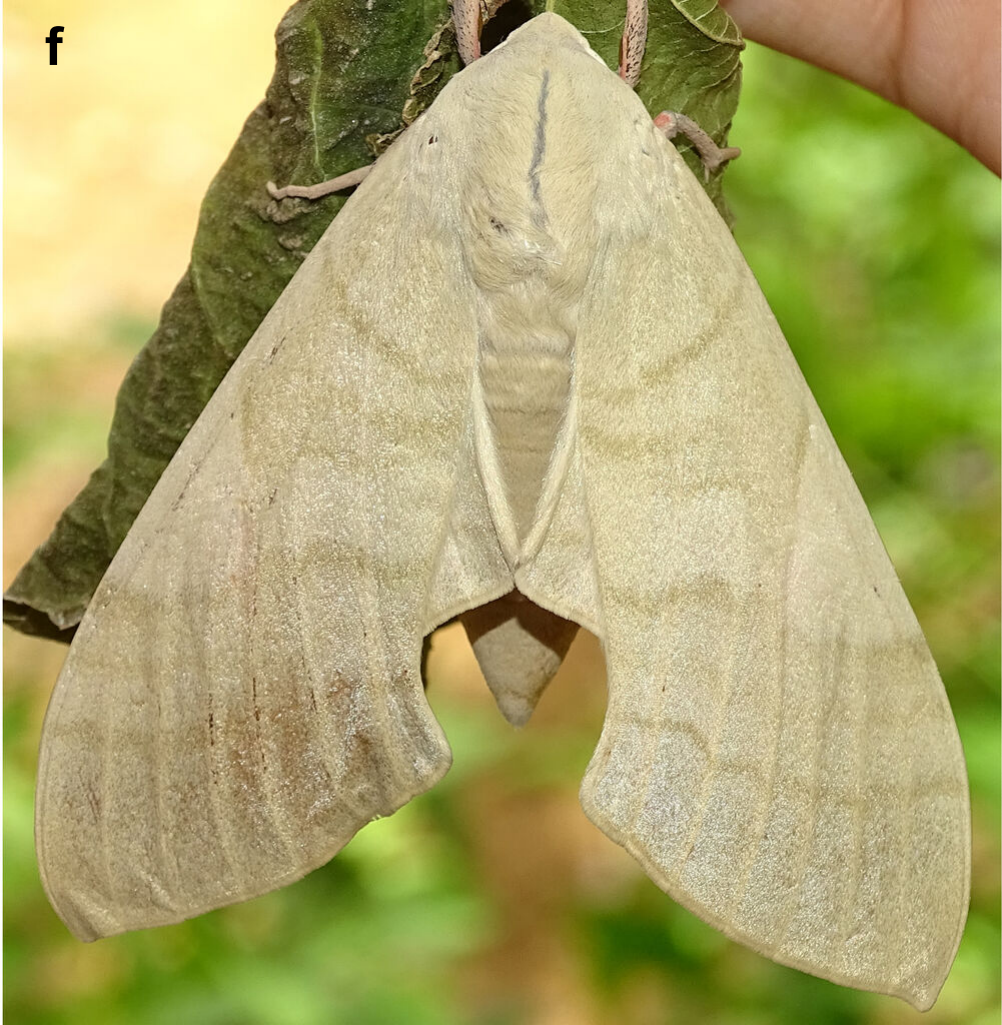
*Clanis* sp.

**Figure 3a.**
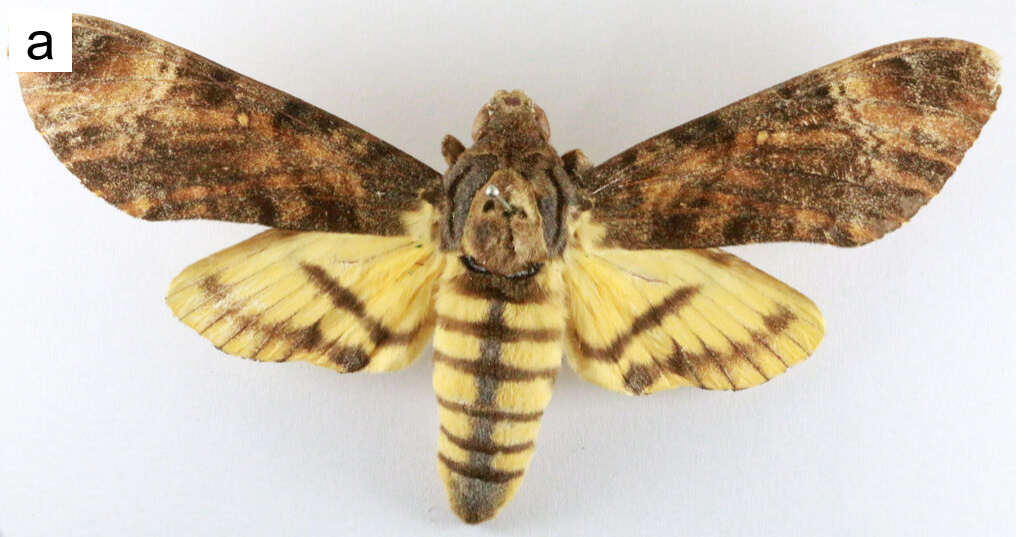
Acherontiastyx

**Figure 3b.**
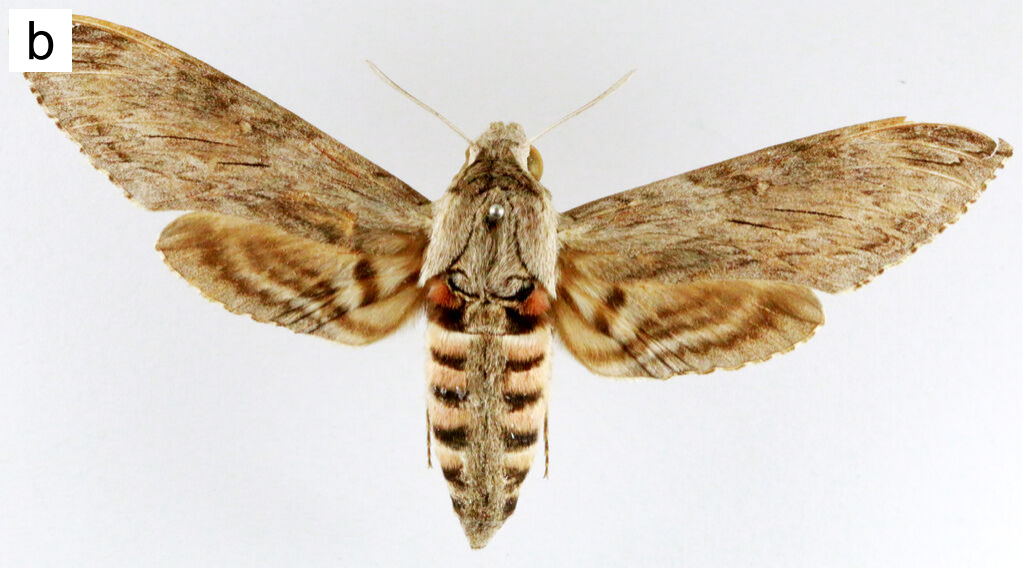
Agriusconvolvuli

**Figure 3c.**
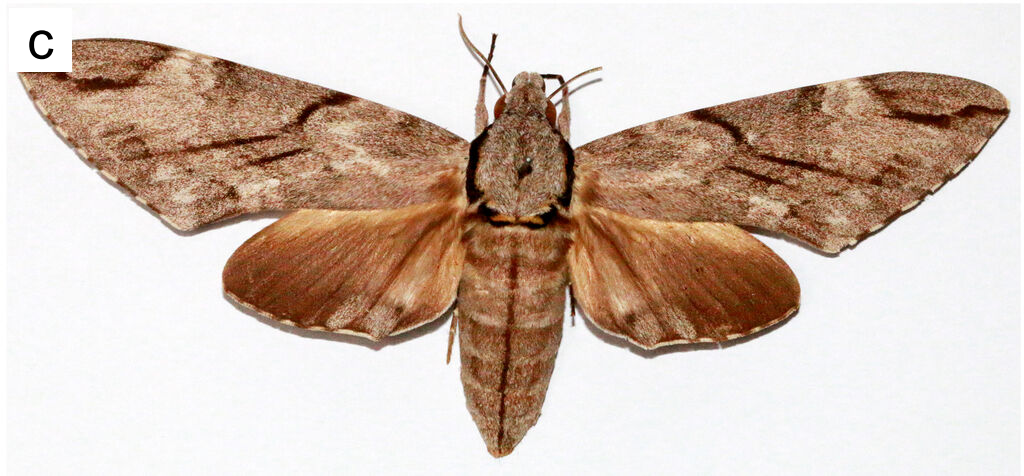
Psilogrammaincreta

**Figure 3d.**
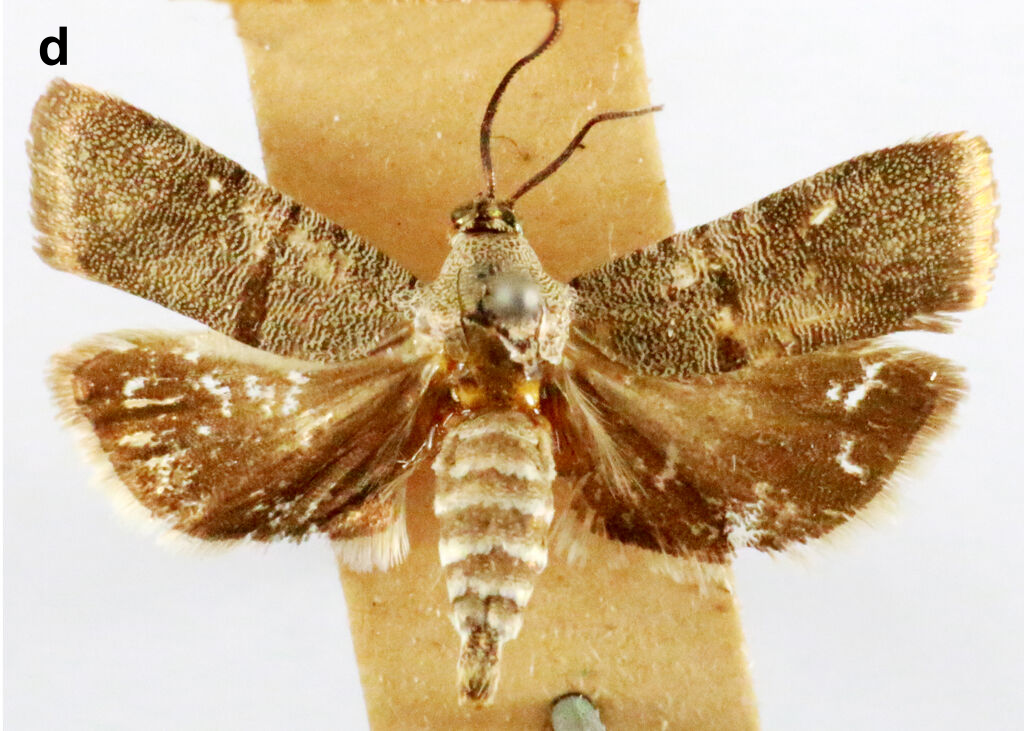
Phycodesminor

**Figure 3e.**
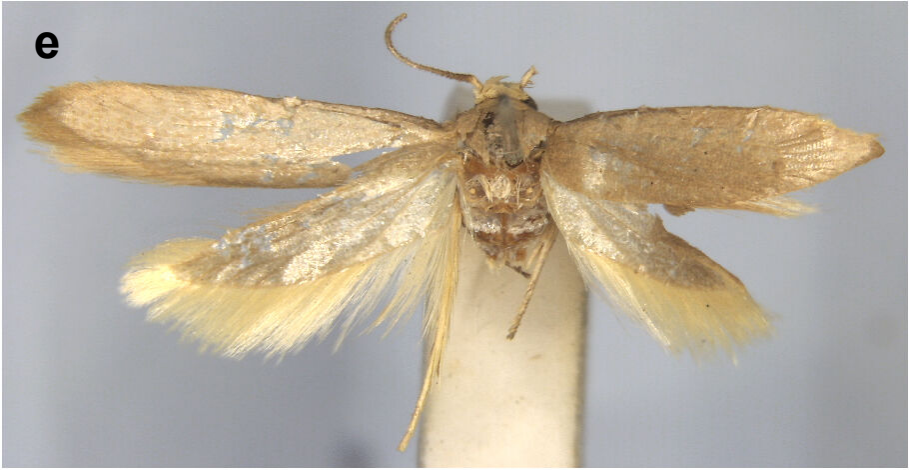
Exinotiscatachlora

**Figure 3f.**
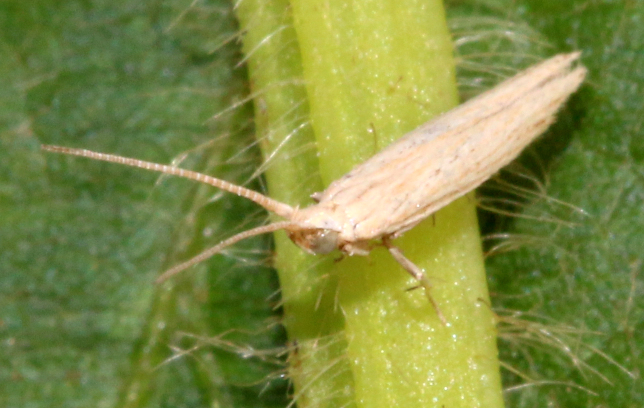
*Coleophora* sp.

**Figure 4a.**
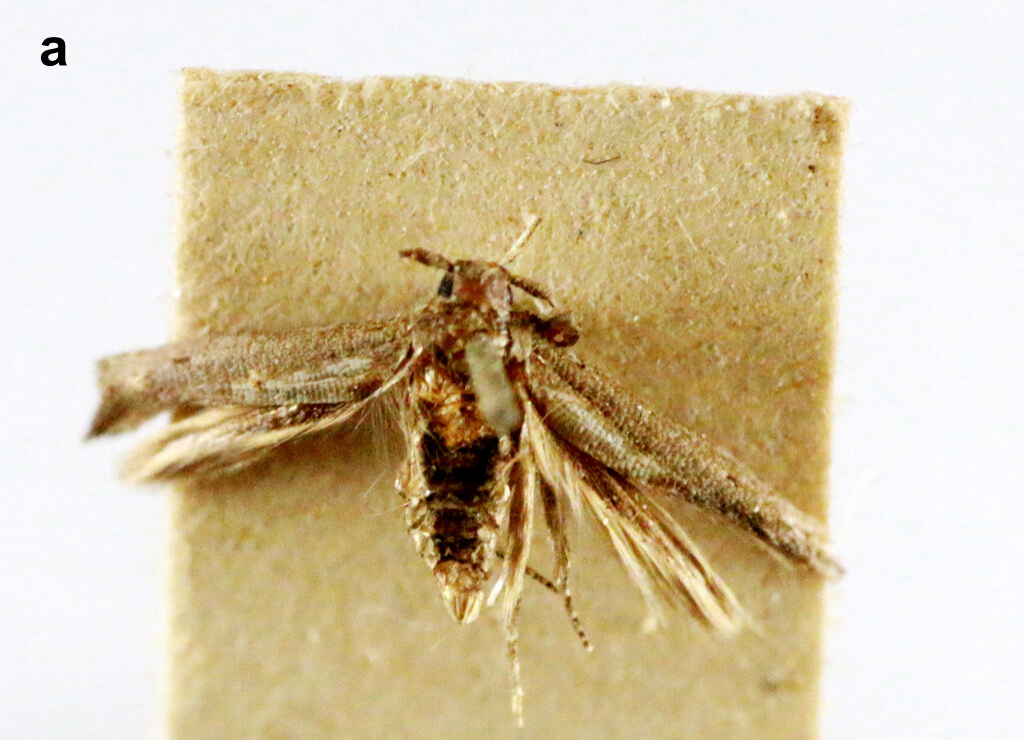
Ascaleniacrypsiloga

**Figure 4b.**
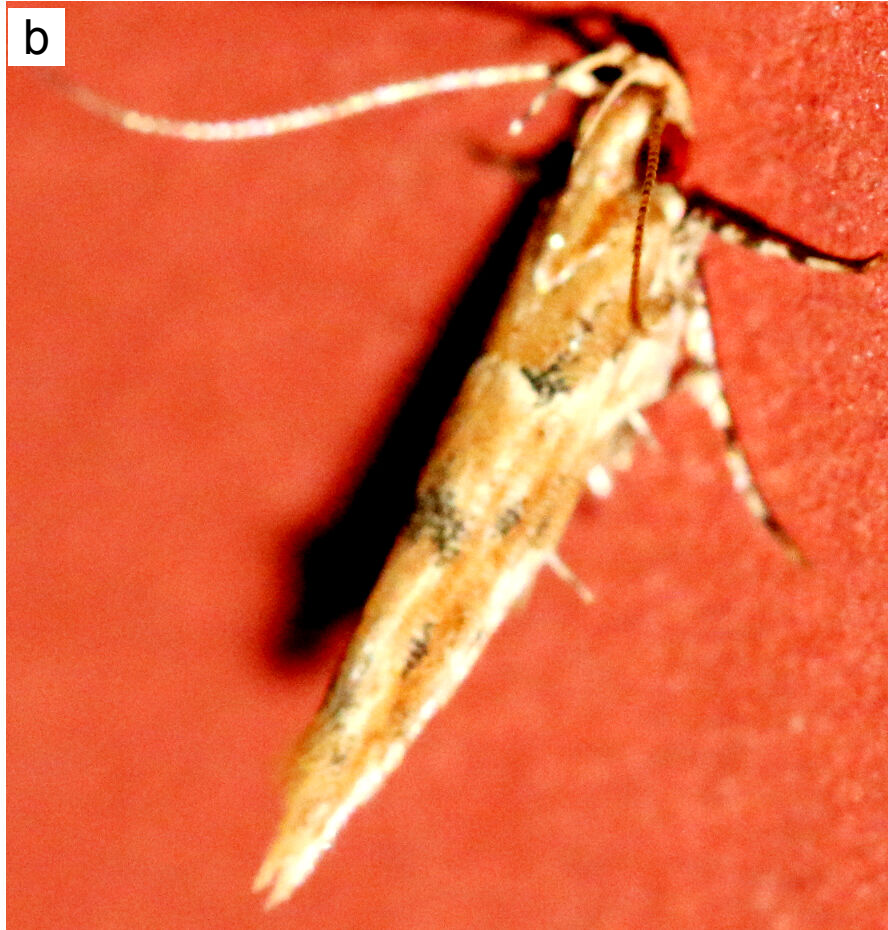
*Anatrachyntis* sp.

**Figure 4c.**
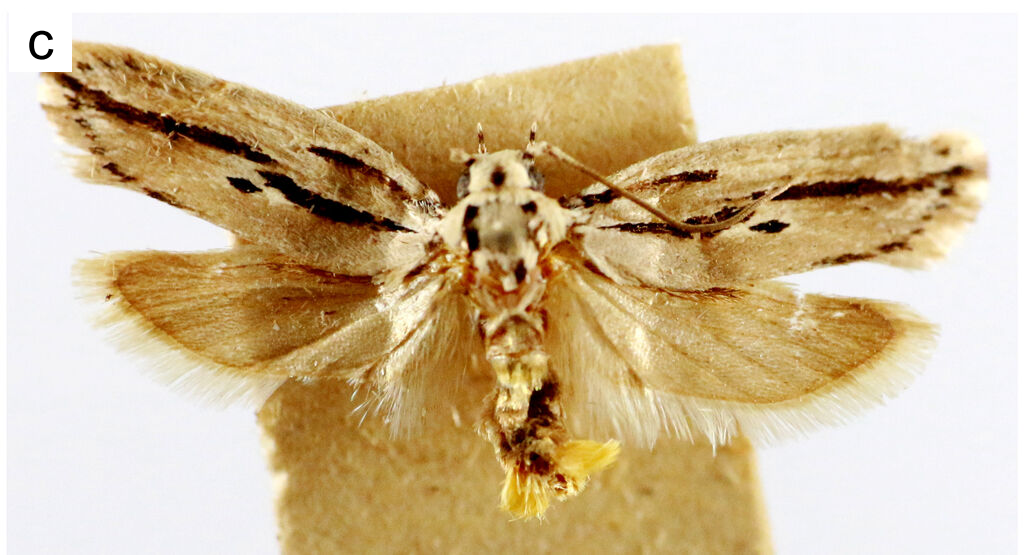
Ethmiaacontias

**Figure 4d.**
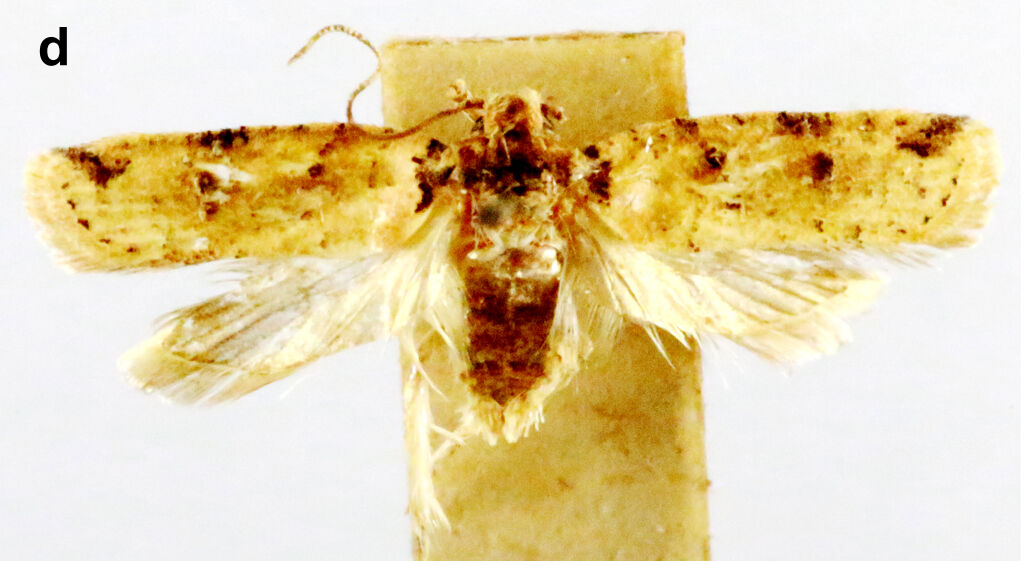
Psorostichazizyphi

**Figure 4e.**
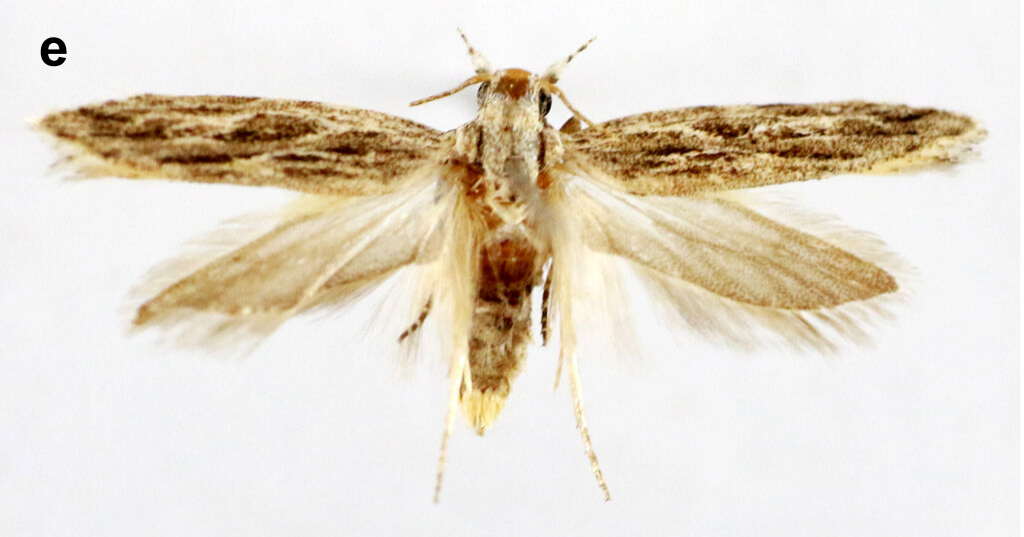
Anarsiaephippias

**Figure 4f.**
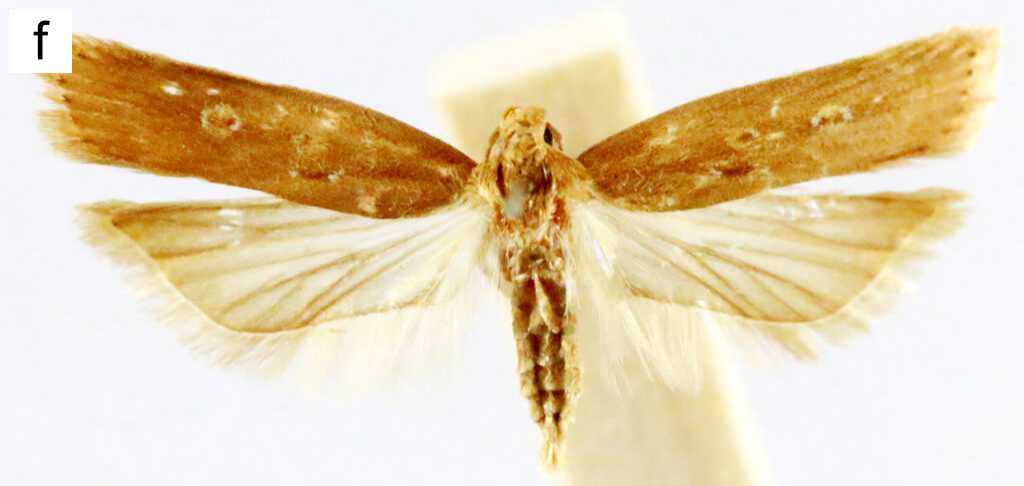
Helcystogrammaengraptum

**Figure 5a.**
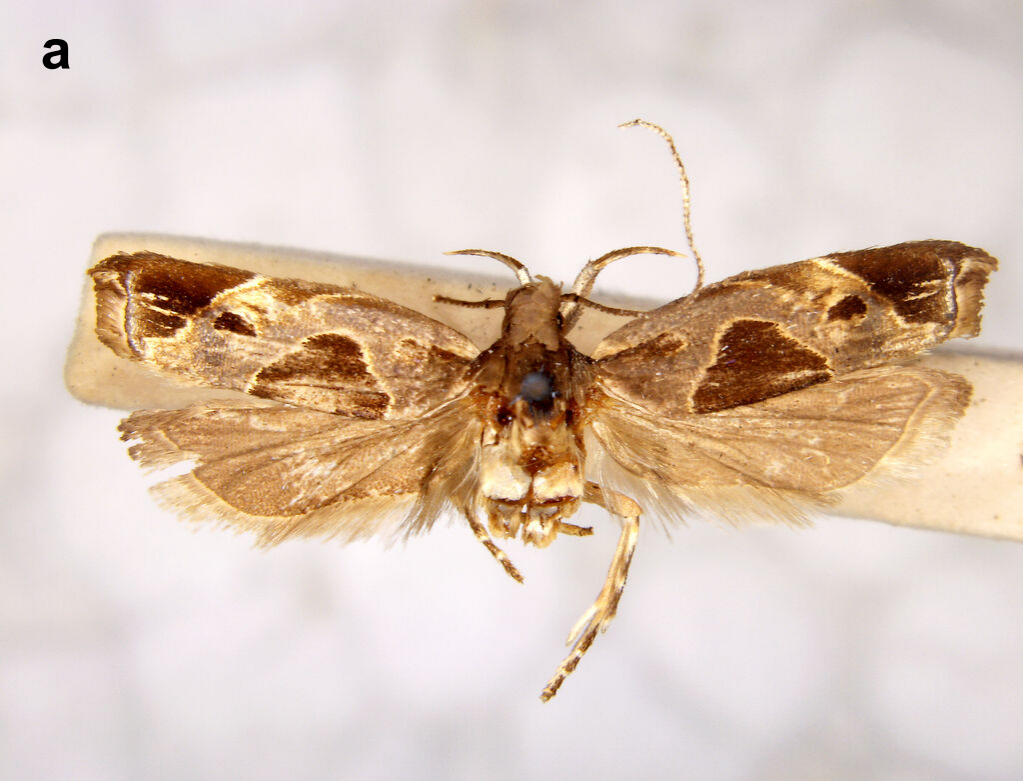
Helcystogrammahibisci

**Figure 5b.**
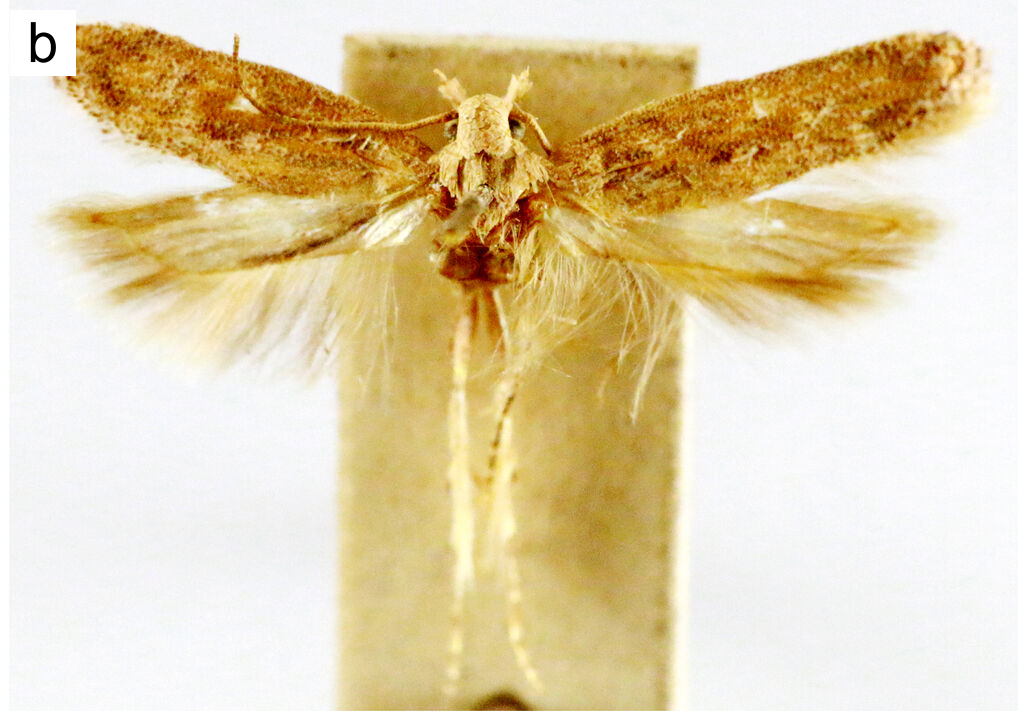
Phthorimaeaoperculella

**Figure 5c.**
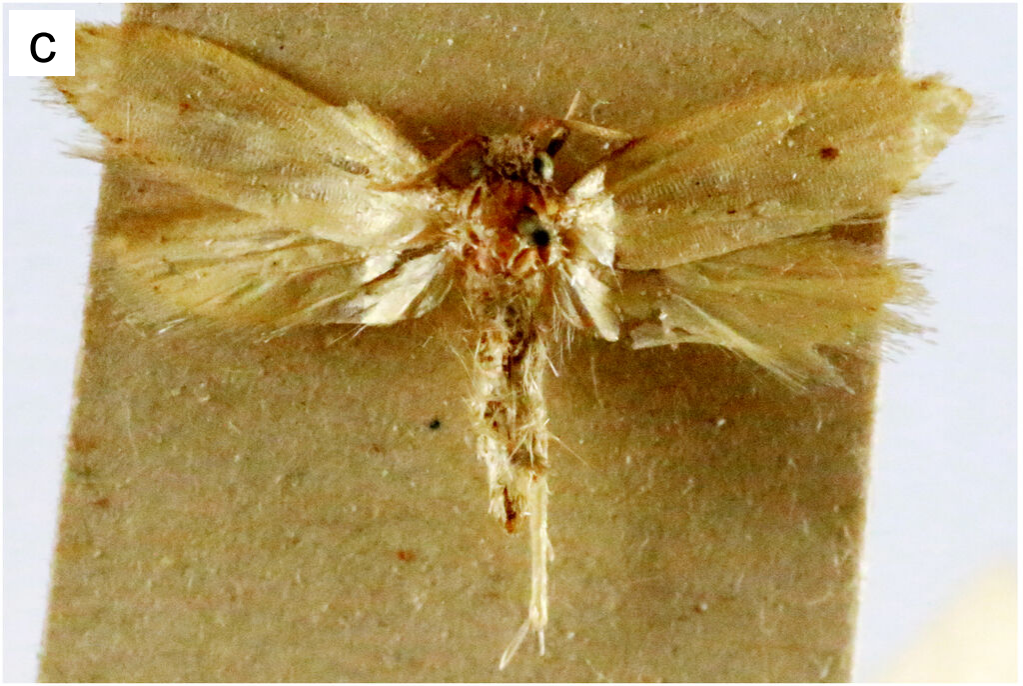
Pseudodoxiaalbinea

**Figure 5d.**
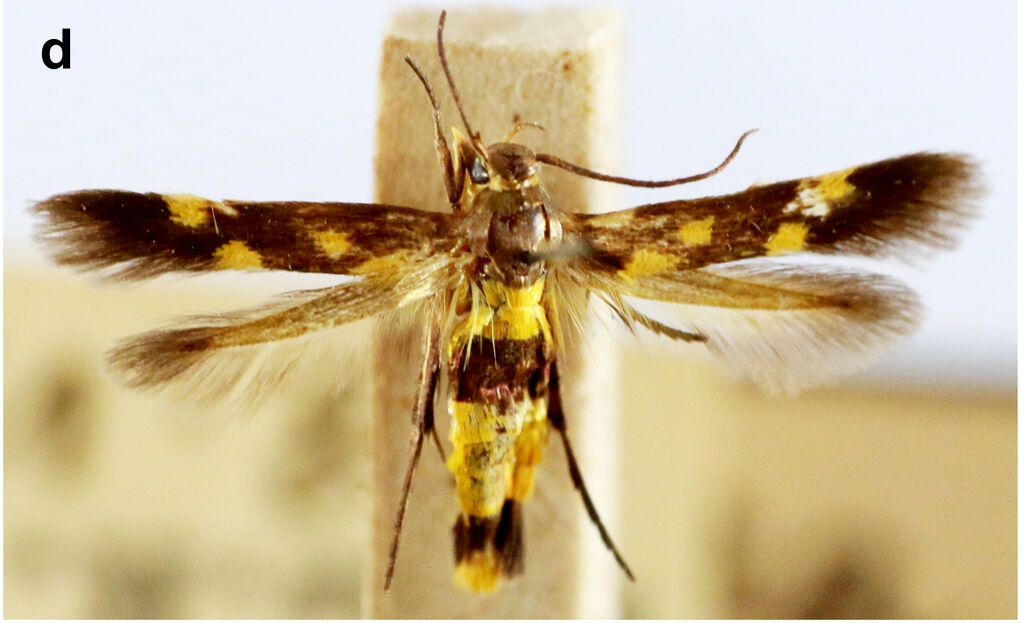
Eretmoceraimpactella

**Figure 5e.**
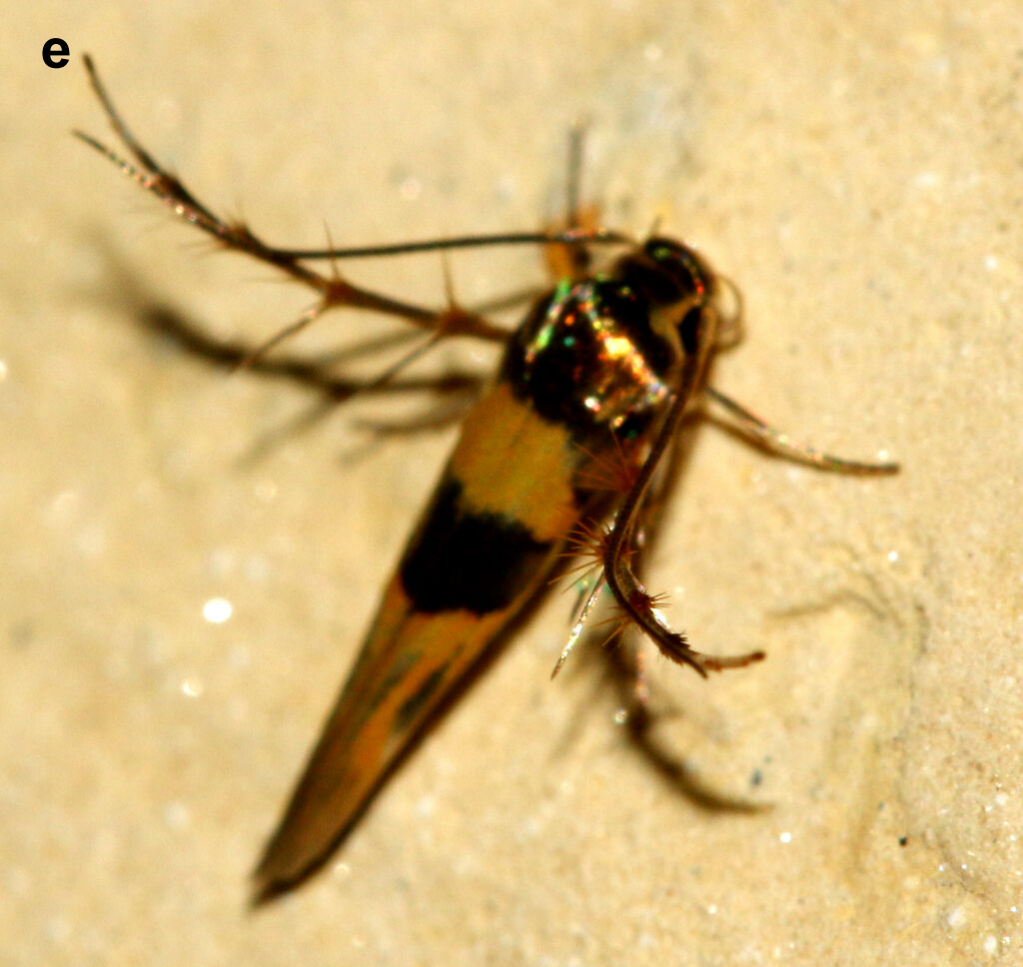
*Stathmopoda* sp.

**Figure 5f.**
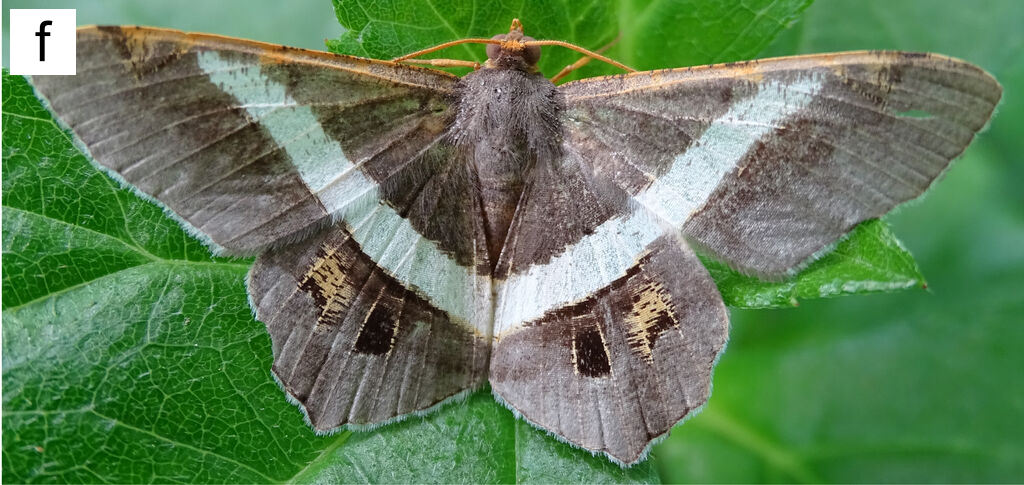
Chiasmianora

**Figure 6a.**
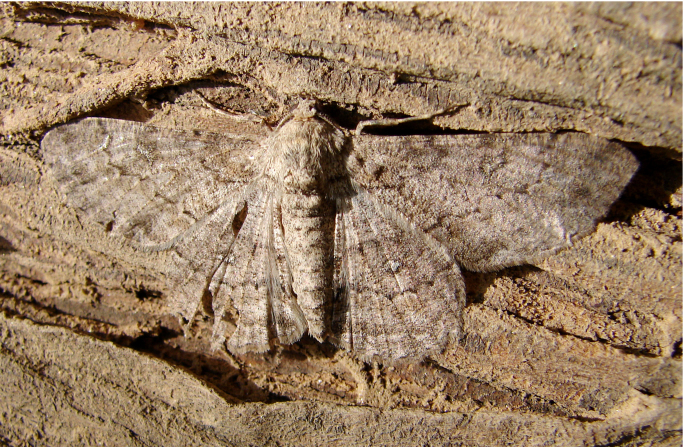
*Cleora* sp.

**Figure 6b.**
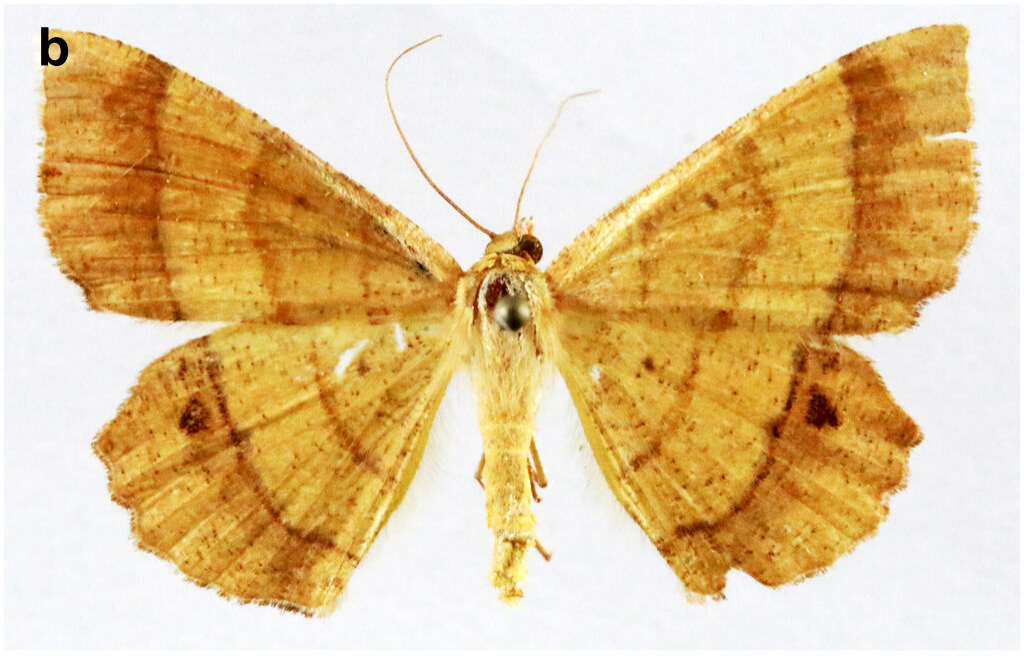
Hyperythraswinhoei

**Figure 6c.**
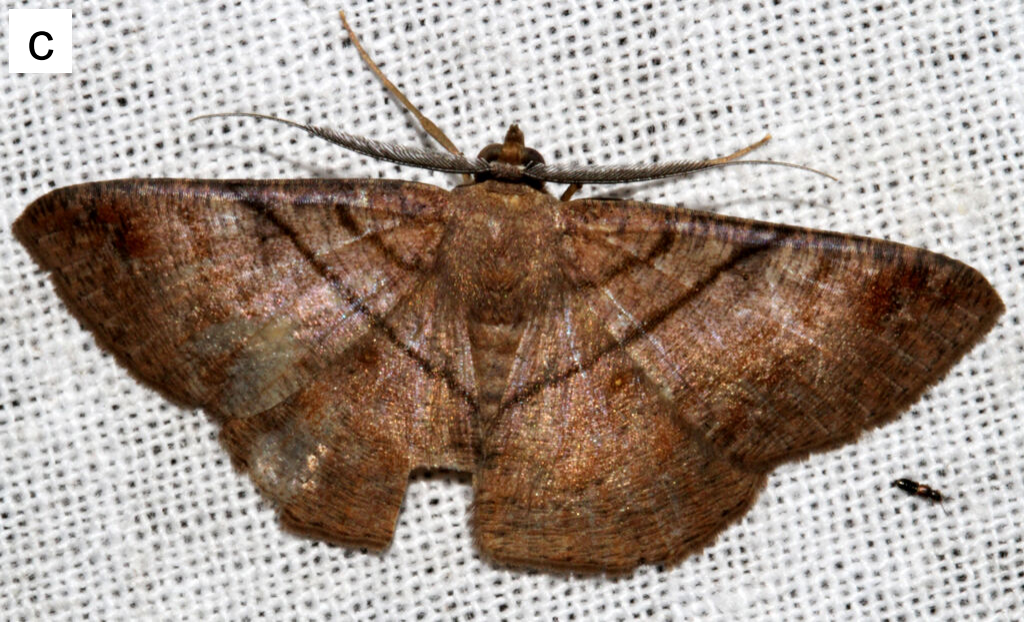
*Petelia* sp.

**Figure 6d.**
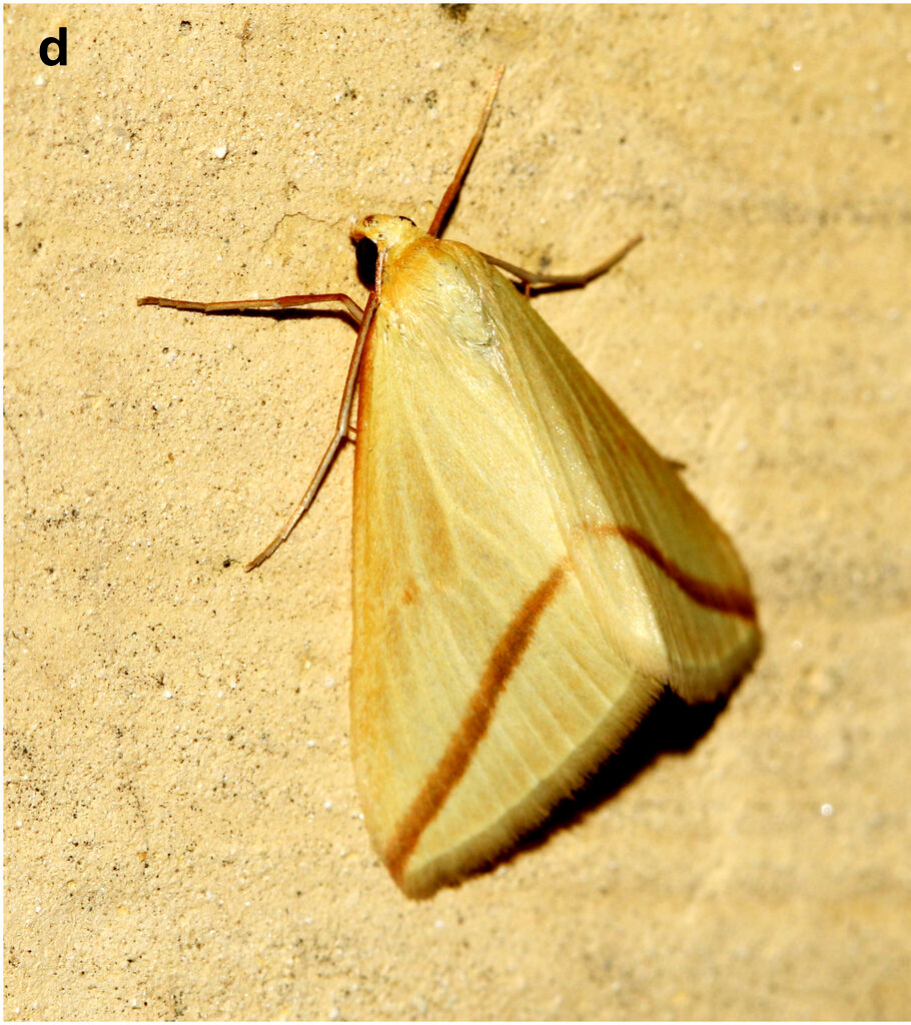
Rhodometrasacraria

**Figure 6e.**
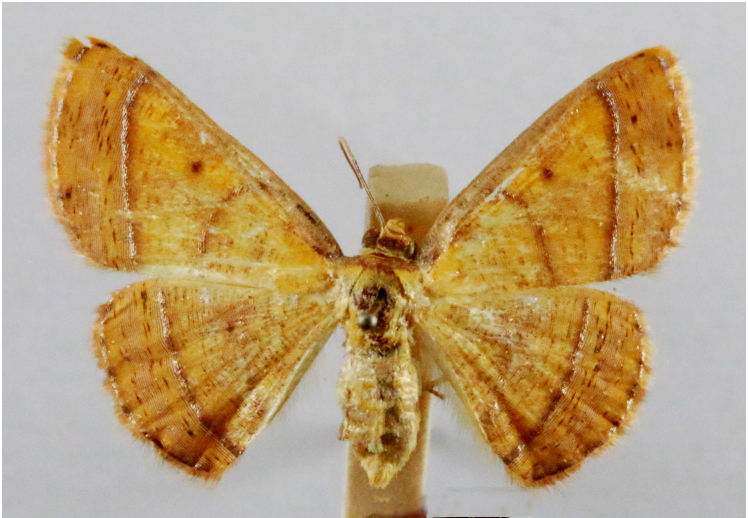
Scardamiametallaria

**Figure 6f.**
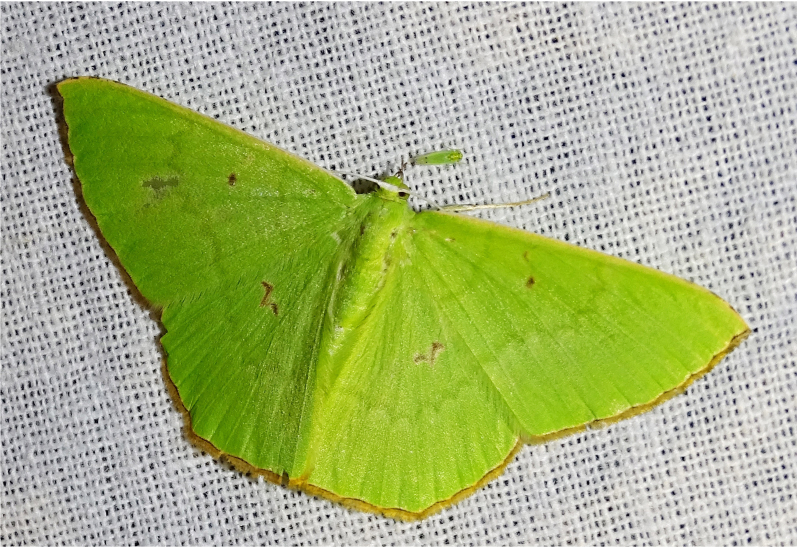
Ornithospilaavicularia

**Figure 7a.**
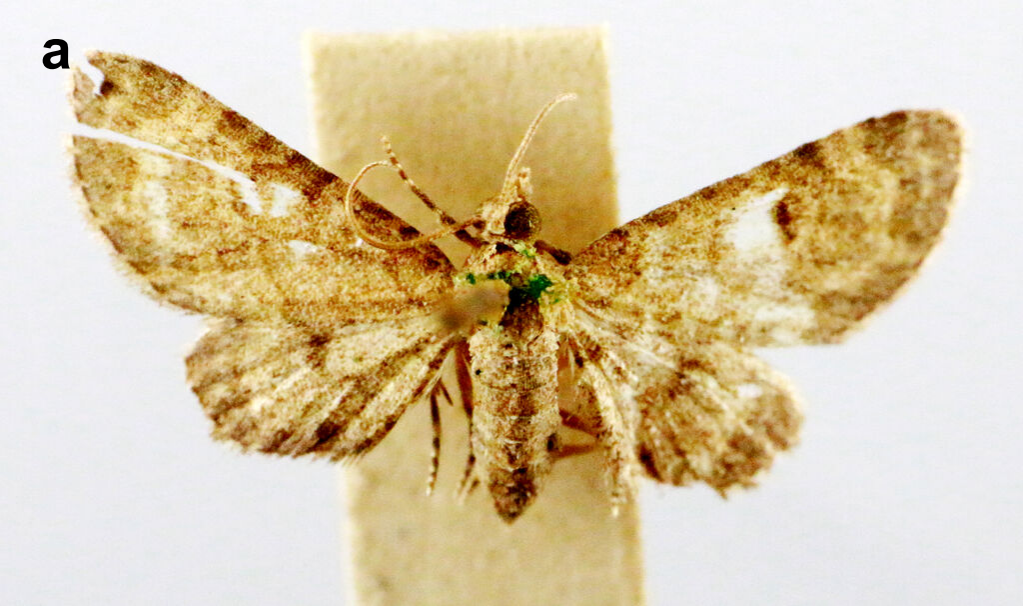
Eupitheciaultimaria

**Figure 7b.**
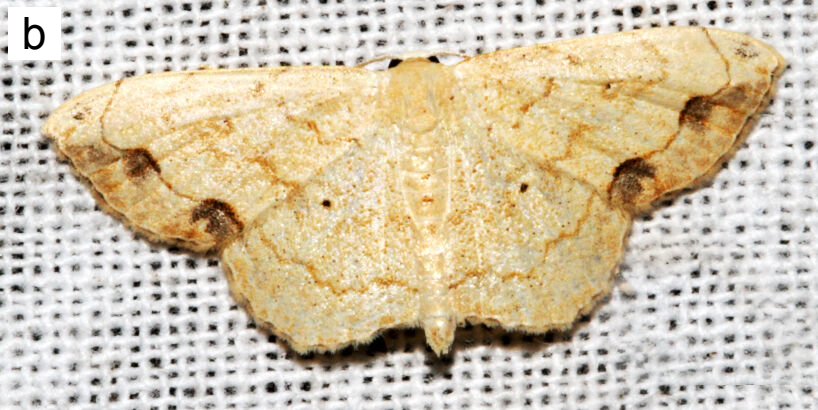
*Craspediopsis* sp.

**Figure 7c.**
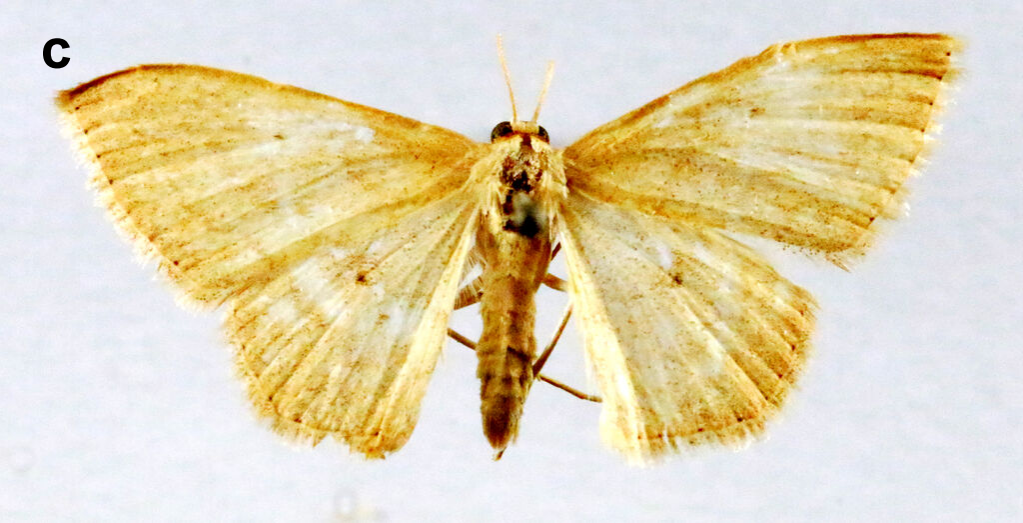
Scopulanesciaria

**Figure 7d.**
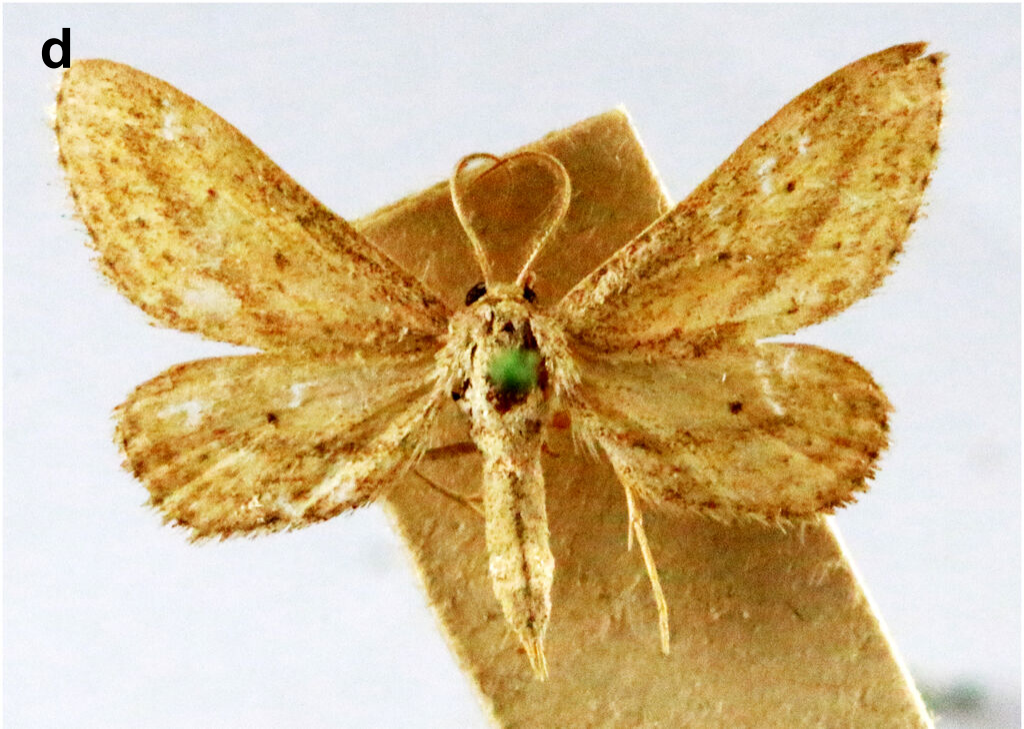
Scopularelictata

**Figure 7e.**
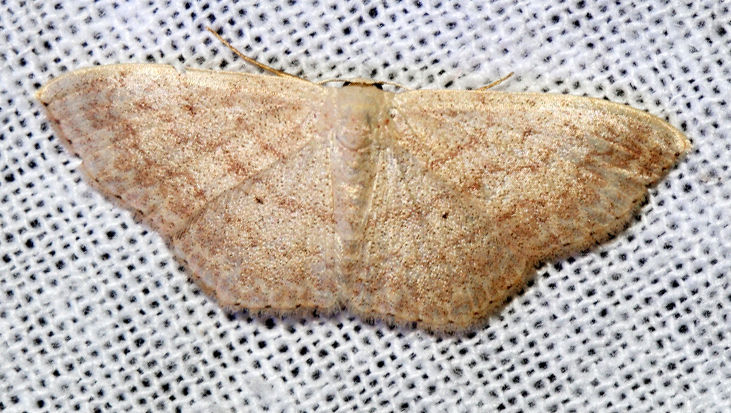
*Scopula* sp.

**Figure 7f.**
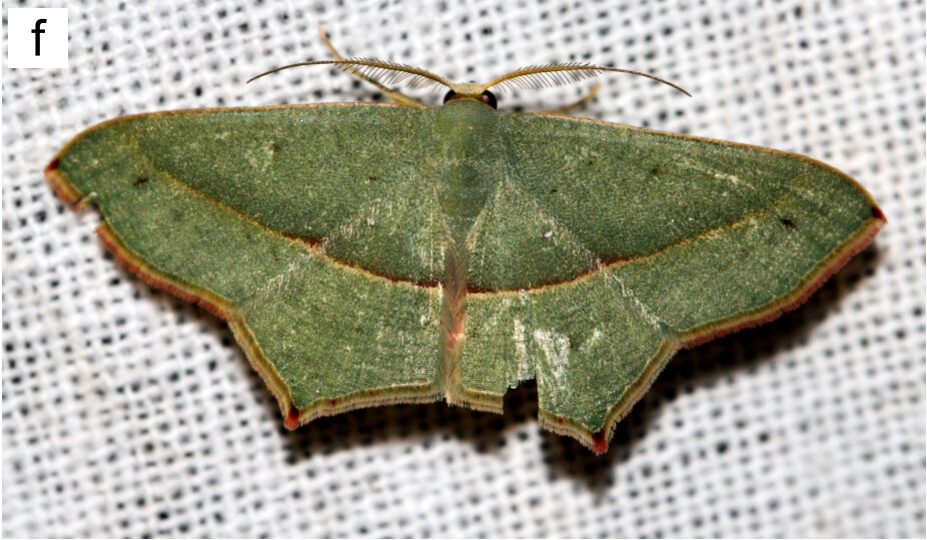
Tramindamundissima

**Figure 8a.**
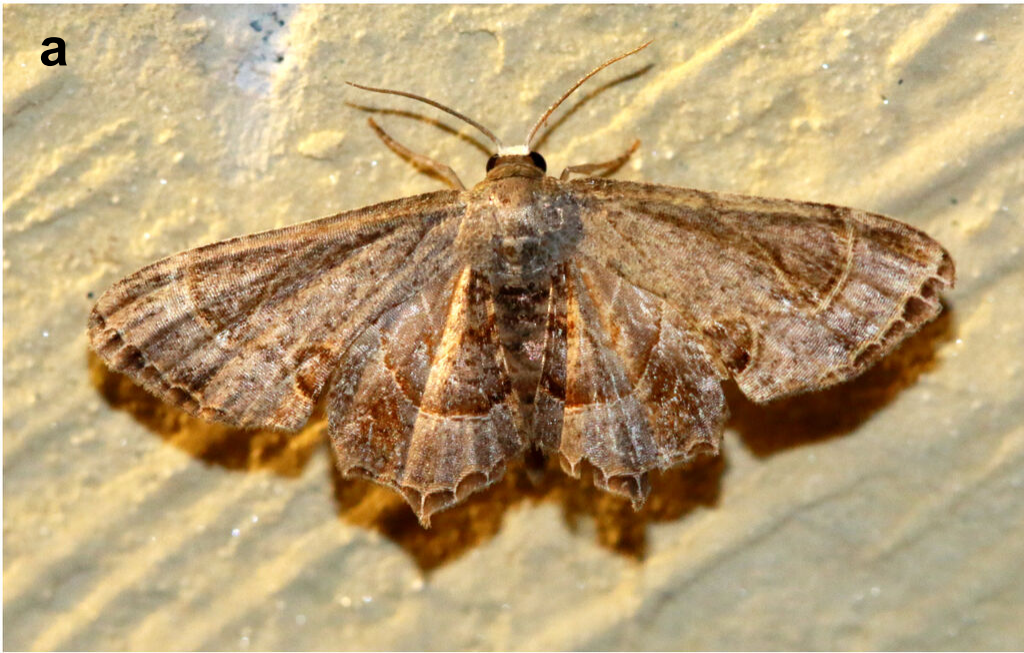
Phazacatheclata

**Figure 8b.**
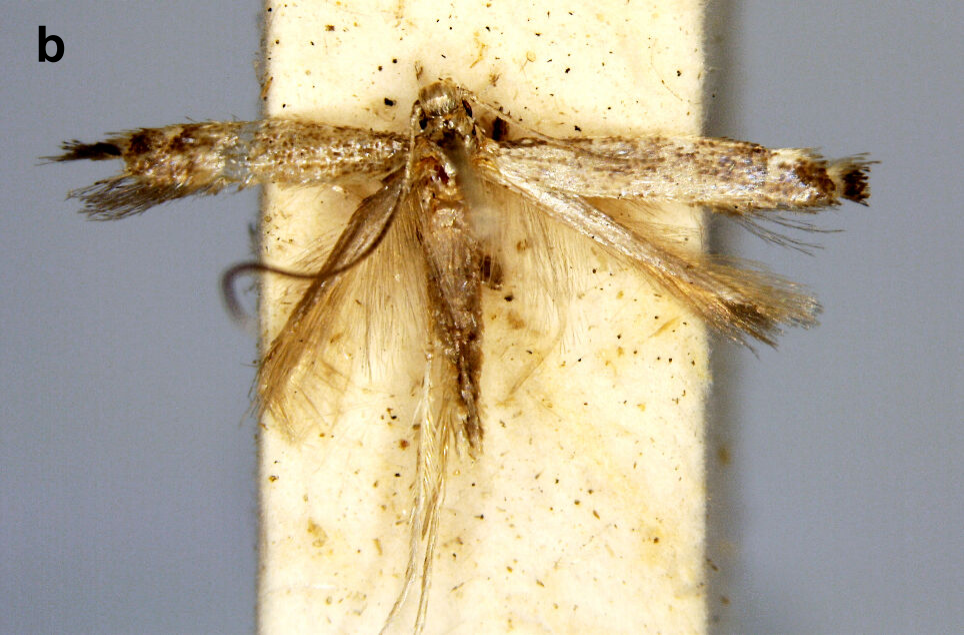
Acrocercopsphaeomorpha

**Figure 8c.**
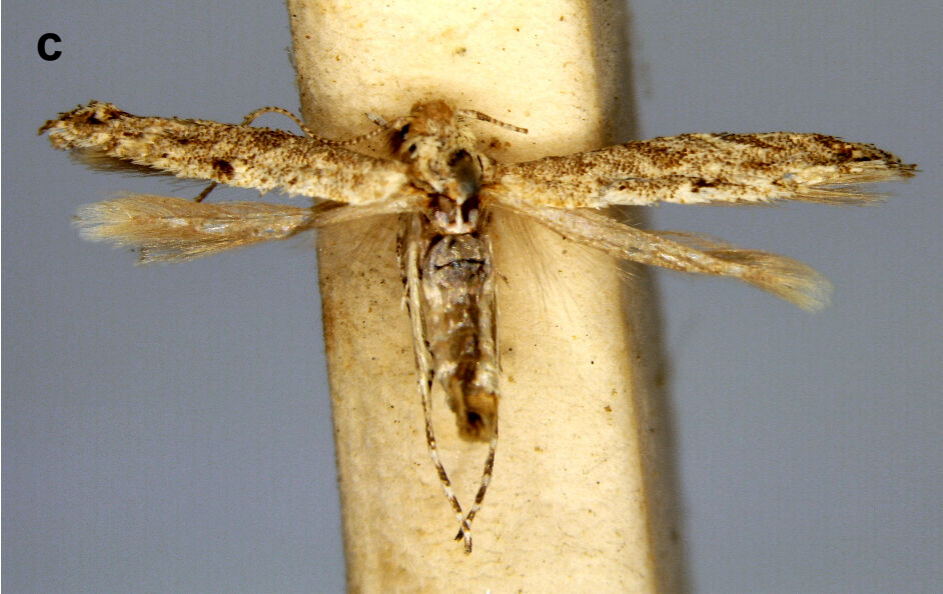
Acrocercopstrissoptila

**Figure 8d.**
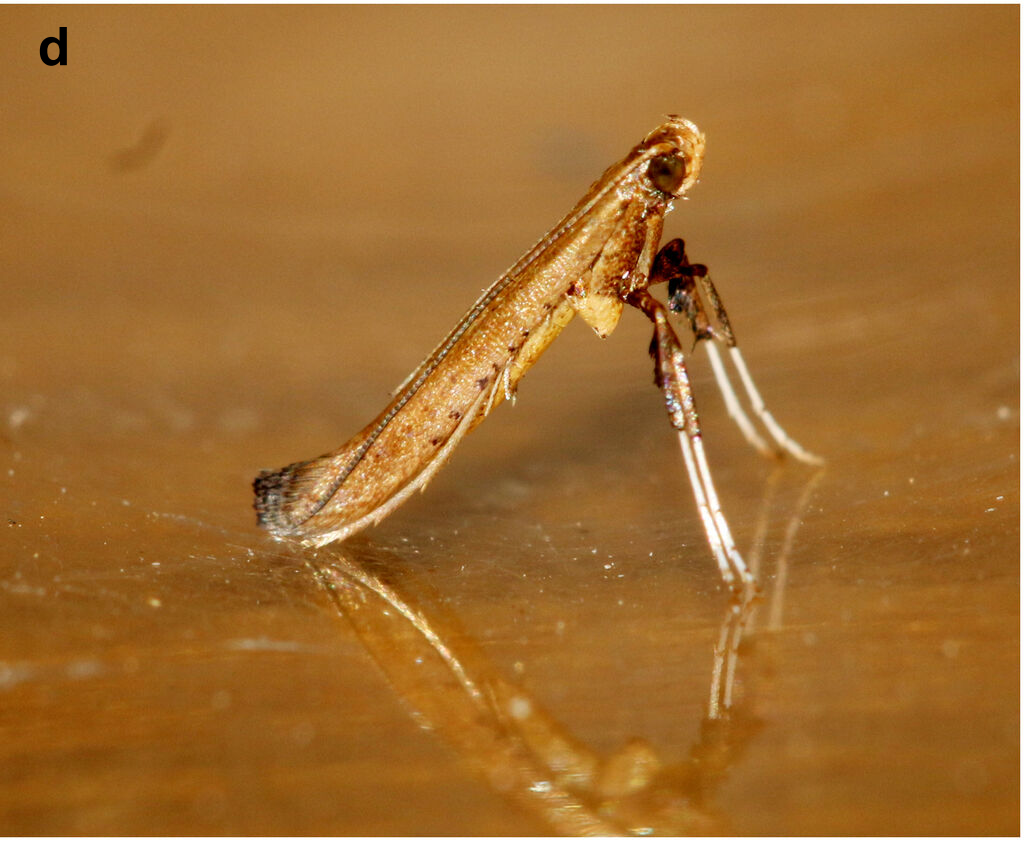
Caloptiliasoyella

**Figure 8e.**
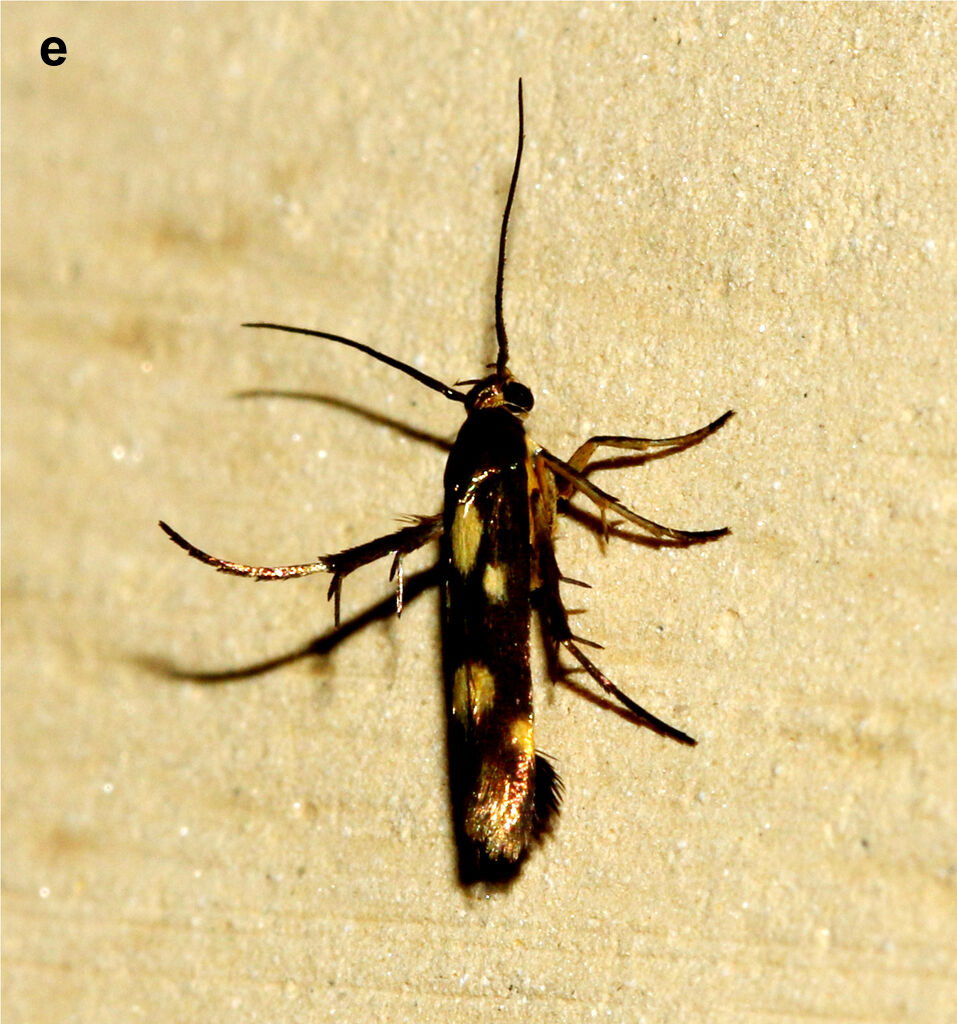
*Euspilapteryx* sp.

**Figure 8f.**
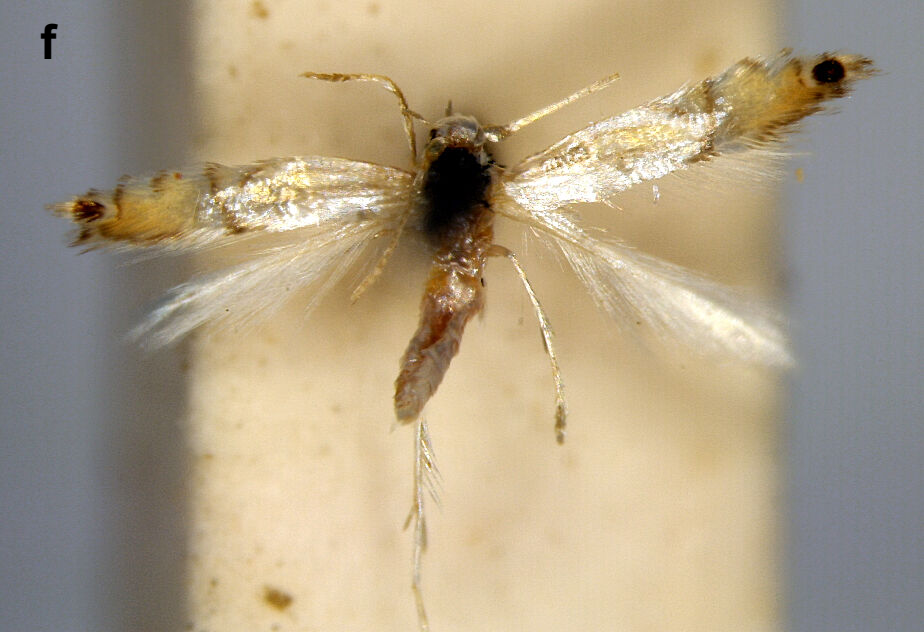
Phyllocnistiscitrella

**Figure 9a.**
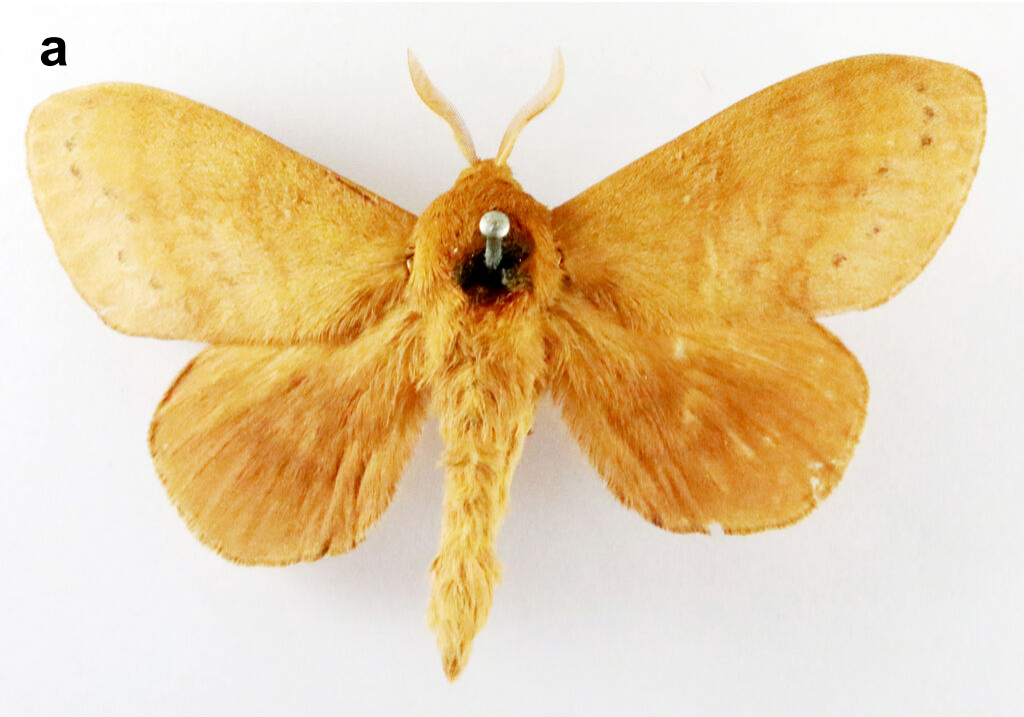
Kunugialatipennis

**Figure 9b.**
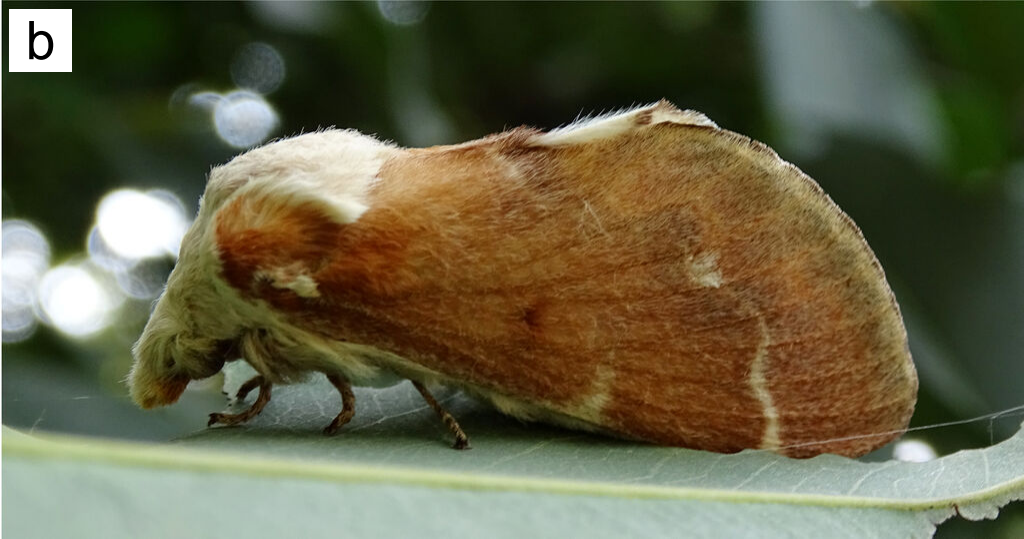
*Streblote* sp.

**Figure 9c.**
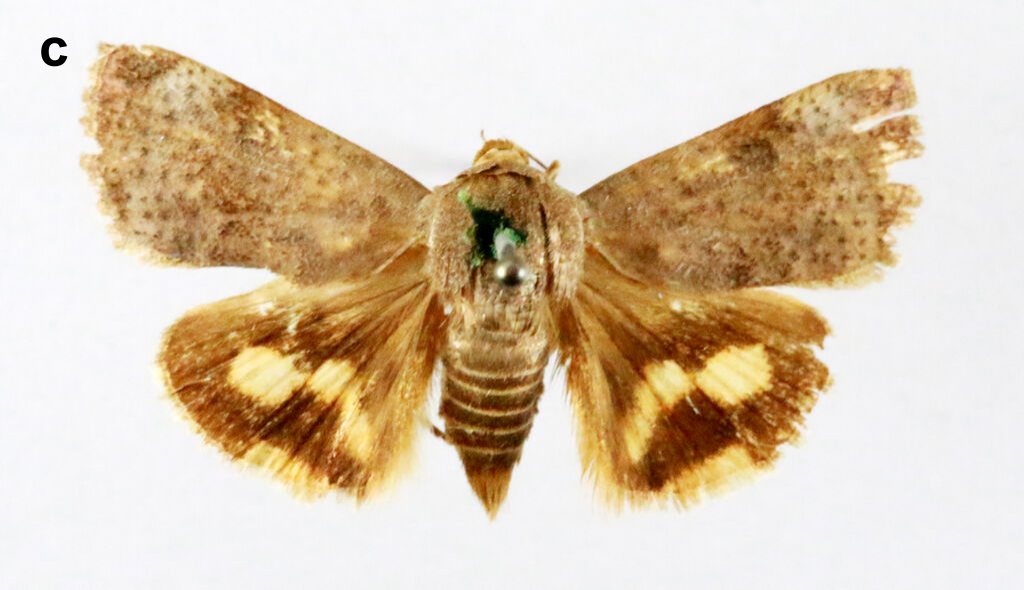
Hyblaeapuera

**Figure 9d.**
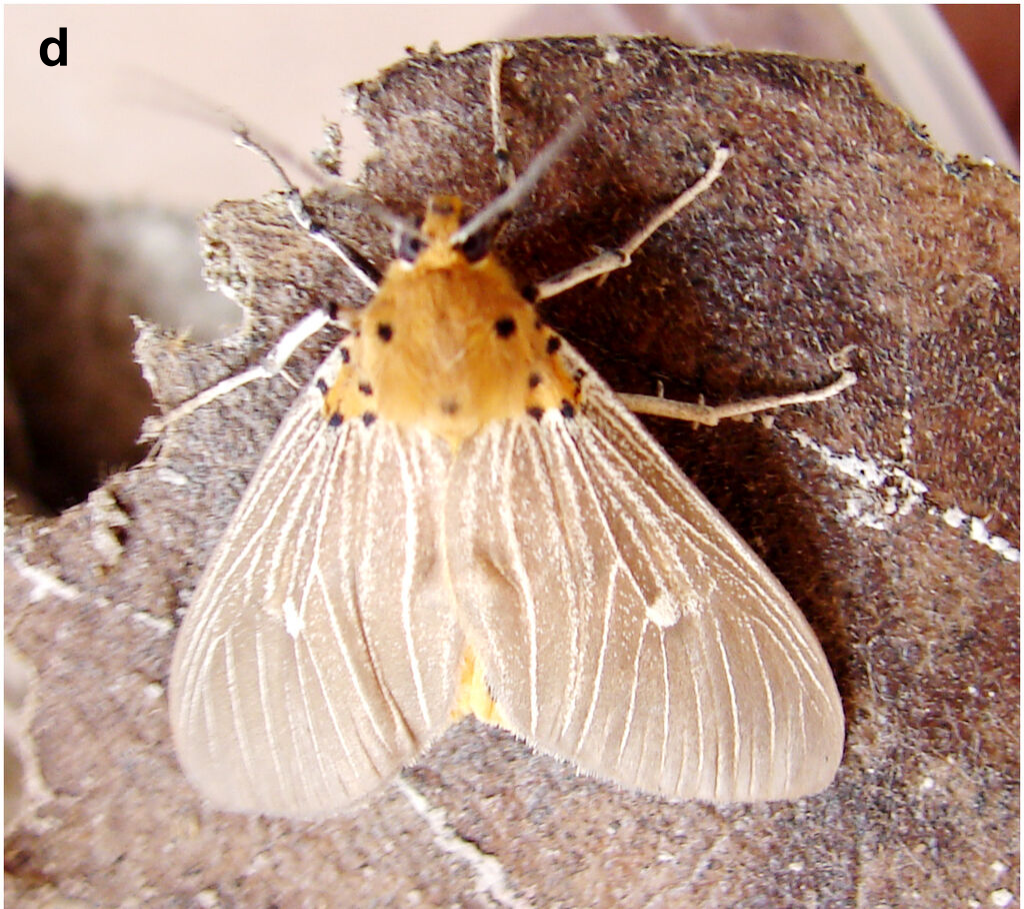
Asotacaricae

**Figure 9e.**
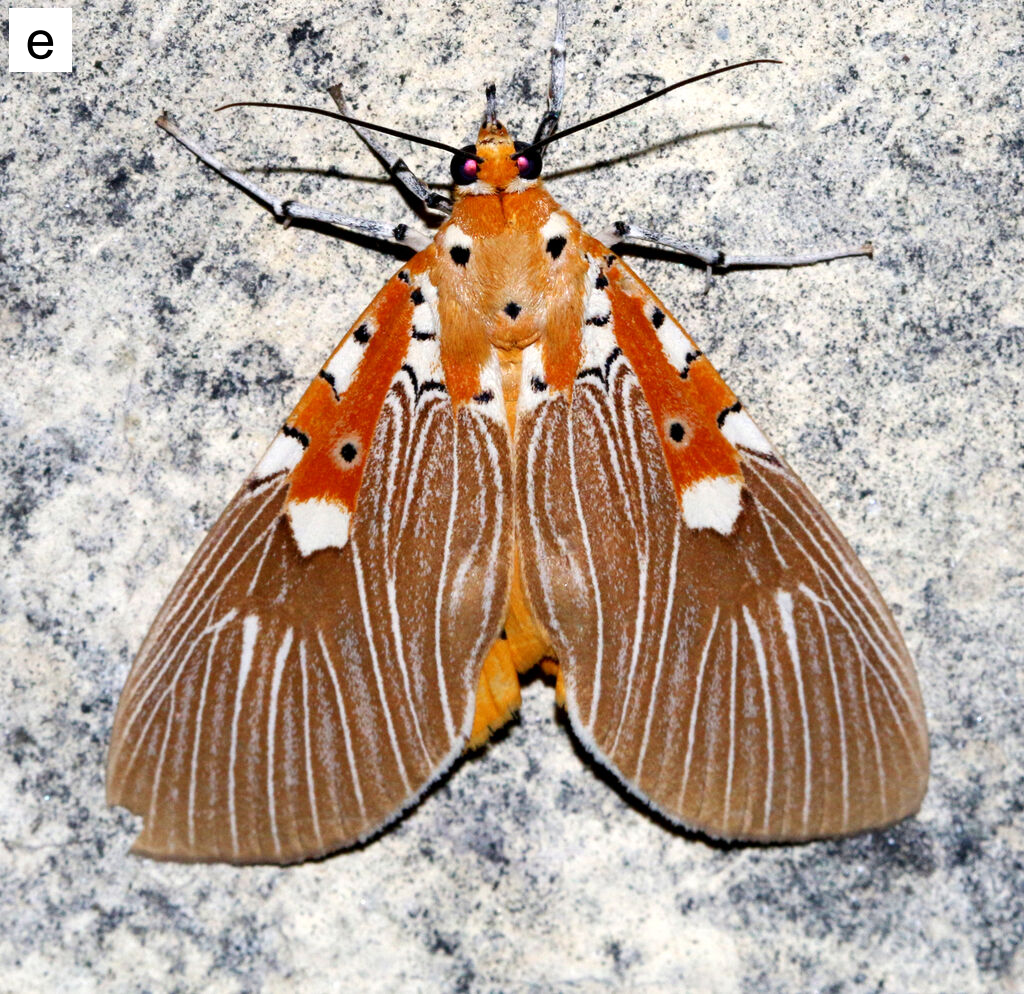
Asotaficus

**Figure 9f.**
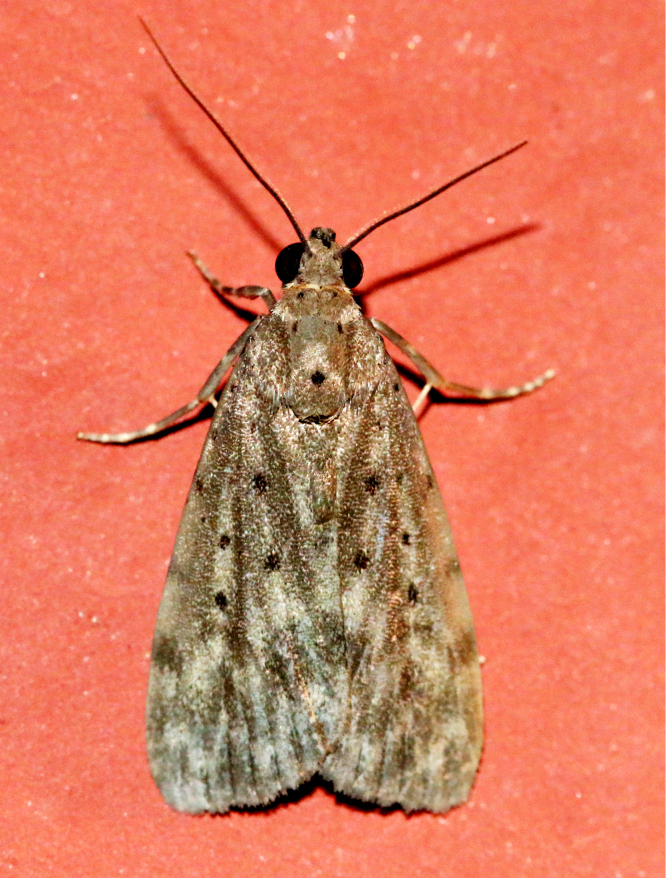
Digamahearseyana

**Figure 10a.**
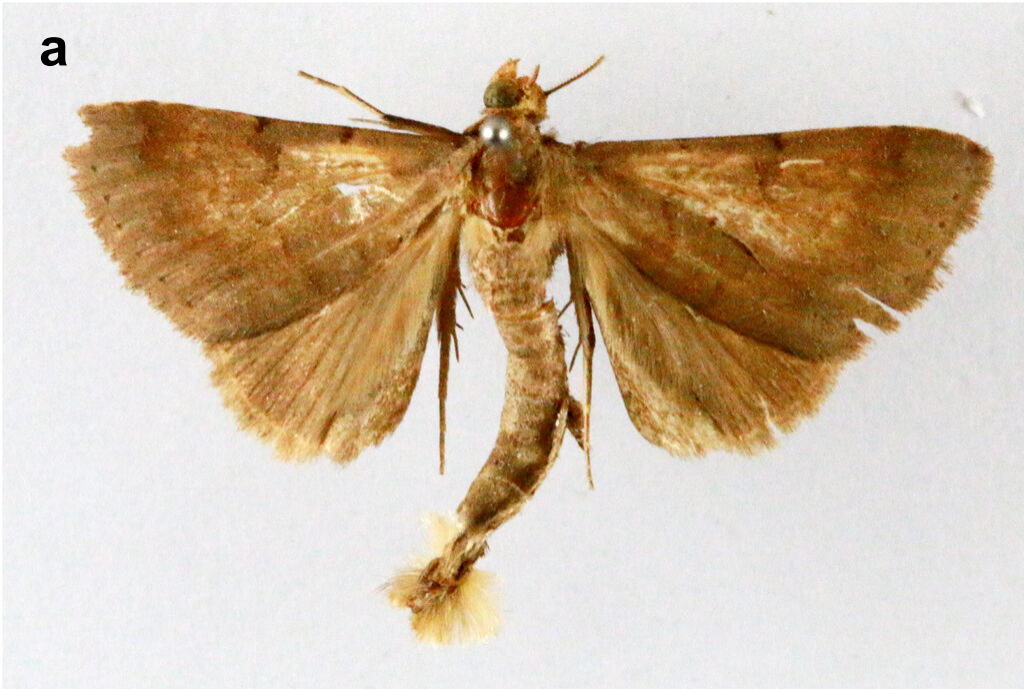
Plecopterareflexa

**Figure 10b.**
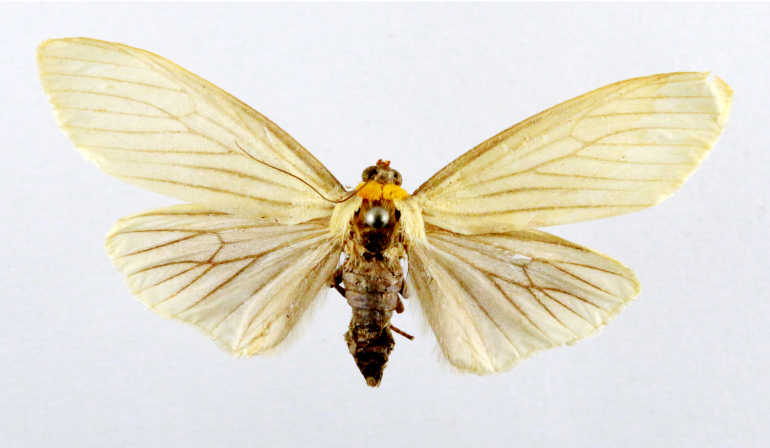
Agyllapallens

**Figure 10c.**
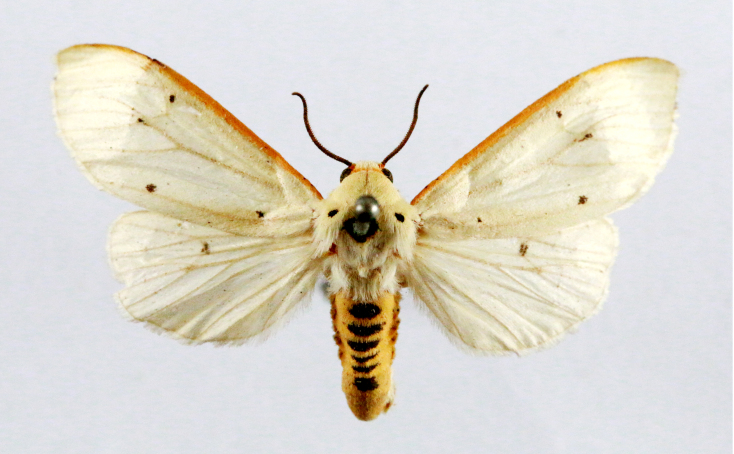
Aloamoorei

**Figure 10d.**
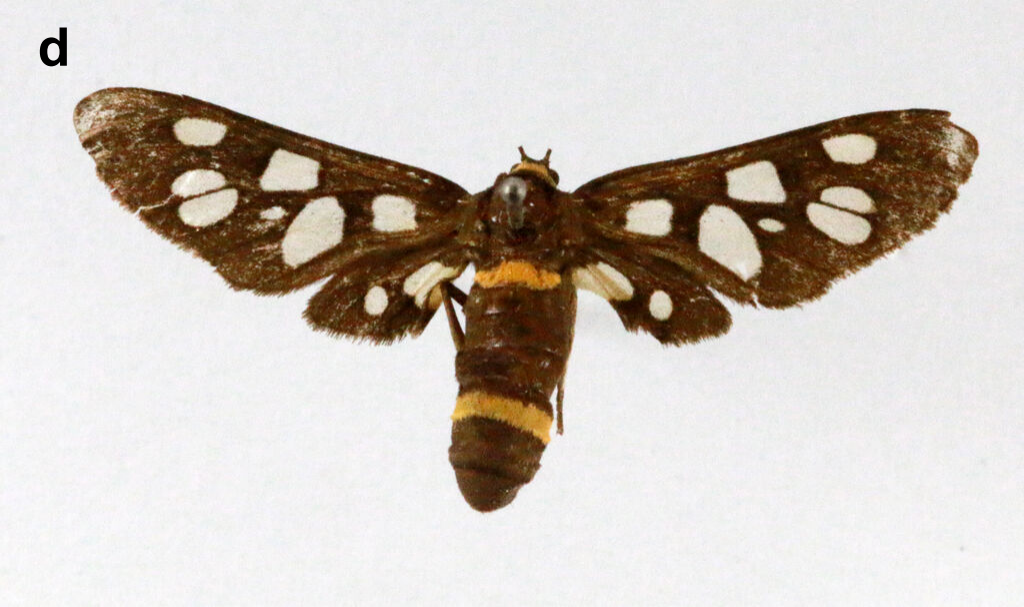
Amatacyssea

**Figure 10e.**
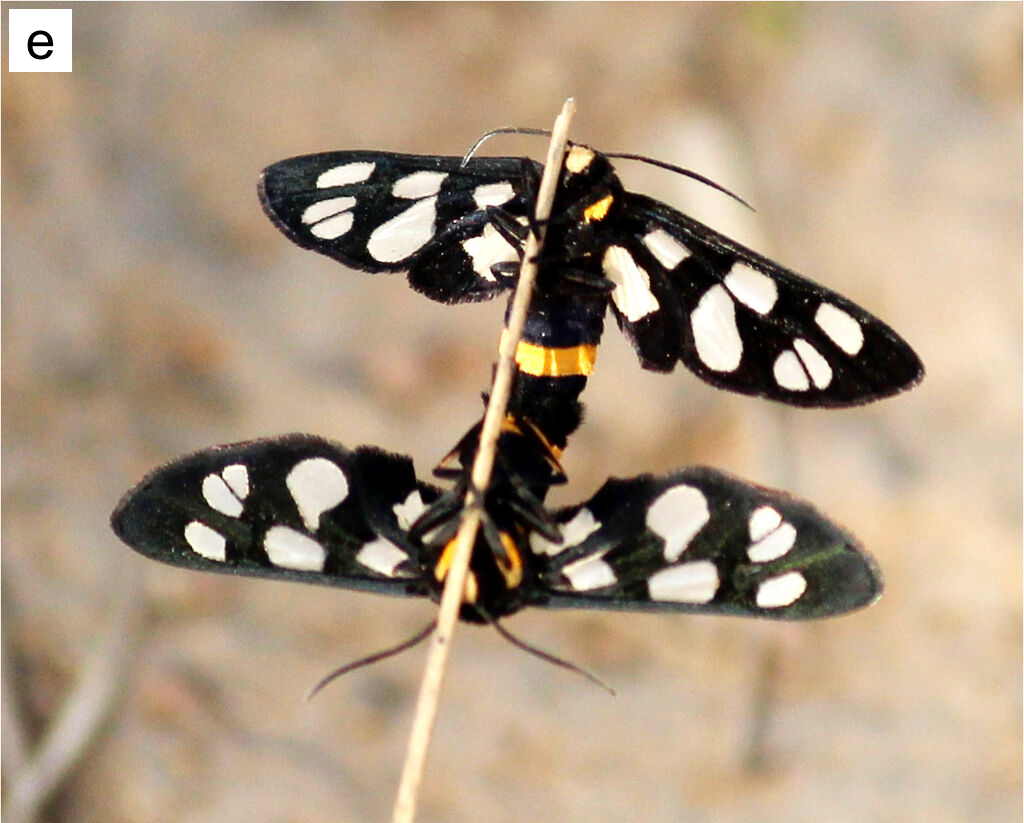
Amatasperbius

**Figure 10f.**
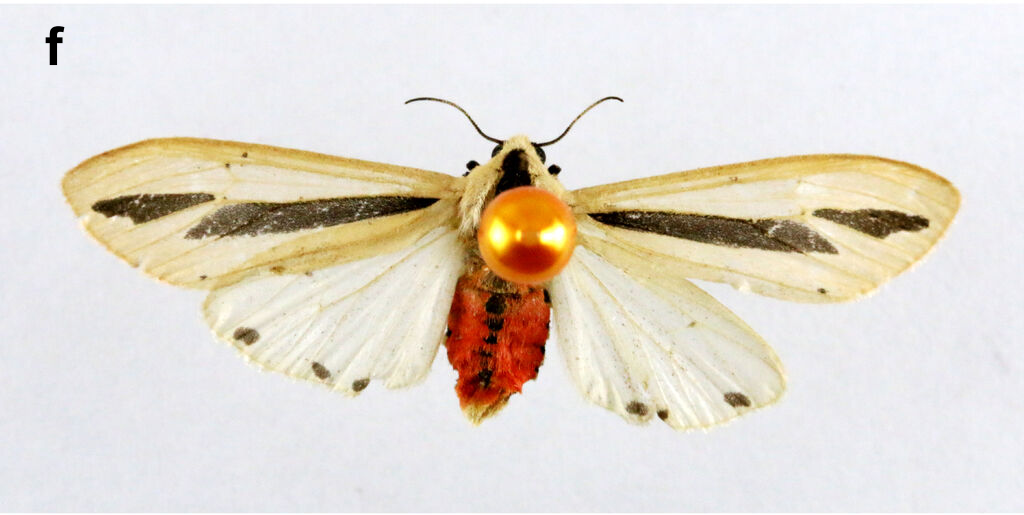
Creatonotosgangis

**Figure 11a.**
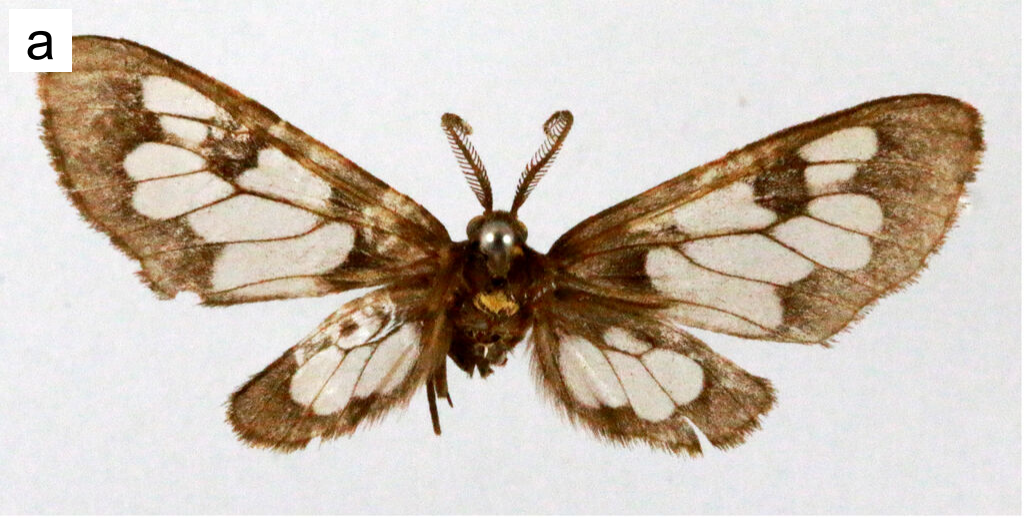
Eressaconfinis

**Figure 11b.**
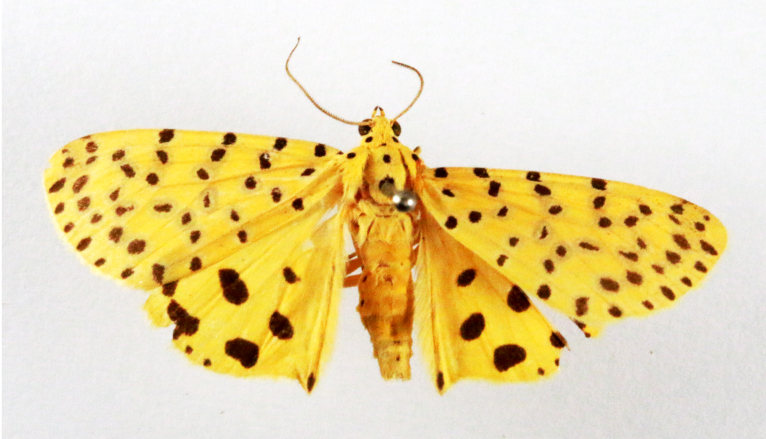
Manginasyringa

**Figure 11c.**
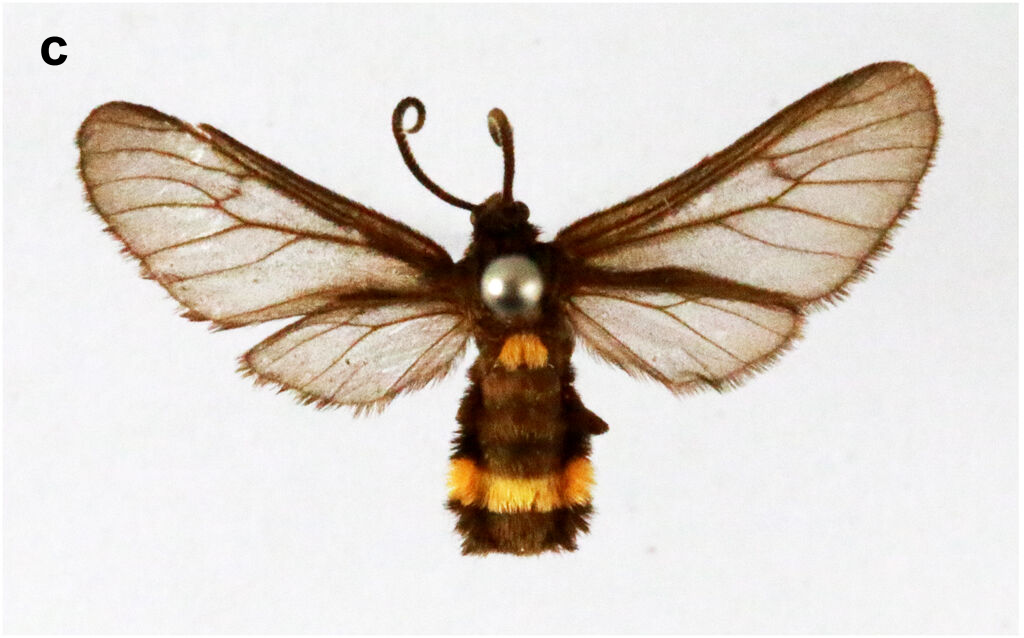
Psichotoeduvaucelii

**Figure 11d.**
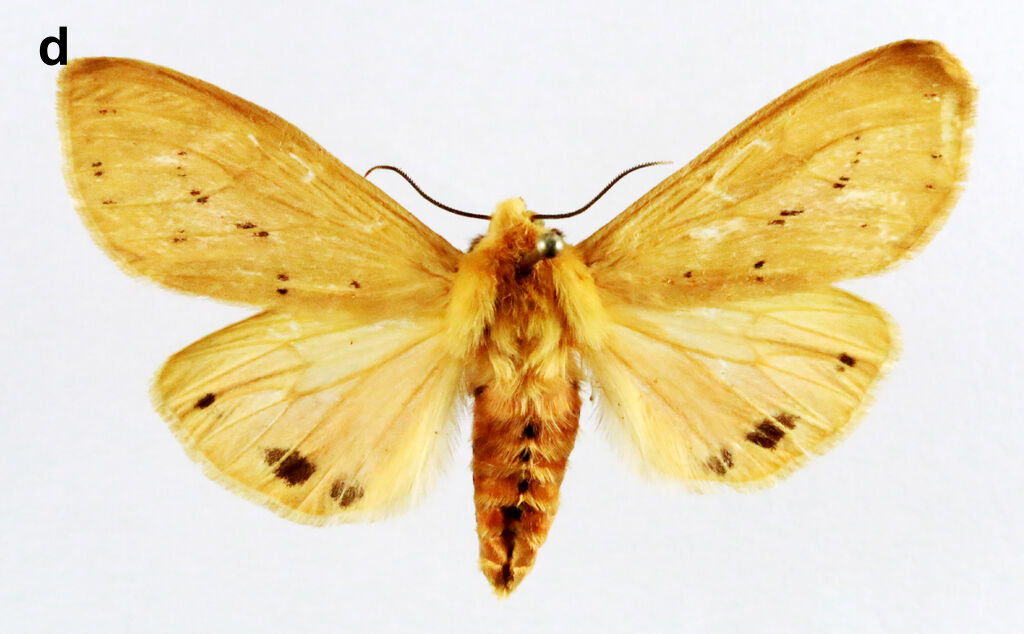
Spilosomaobliqua

**Figure 11e.**
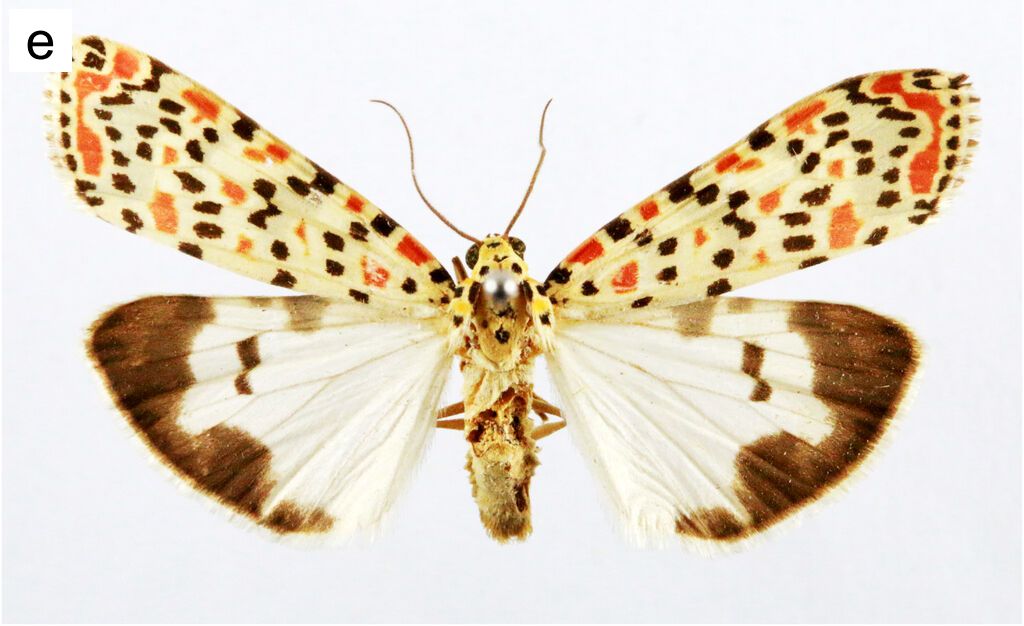
Utethesiapulchella

**Figure 11f.**
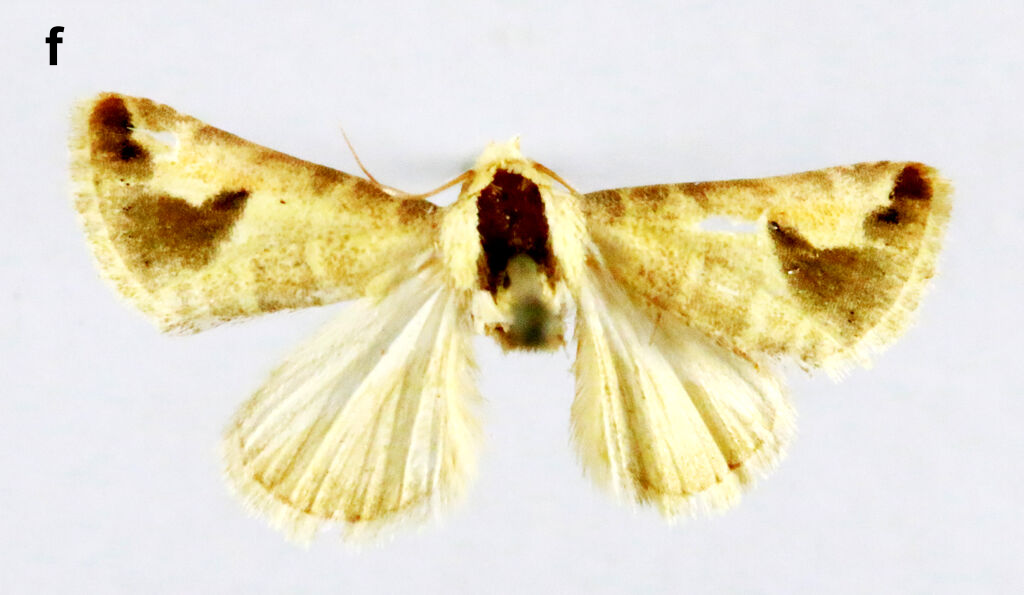
Autobaolivacea

**Figure 12a.**
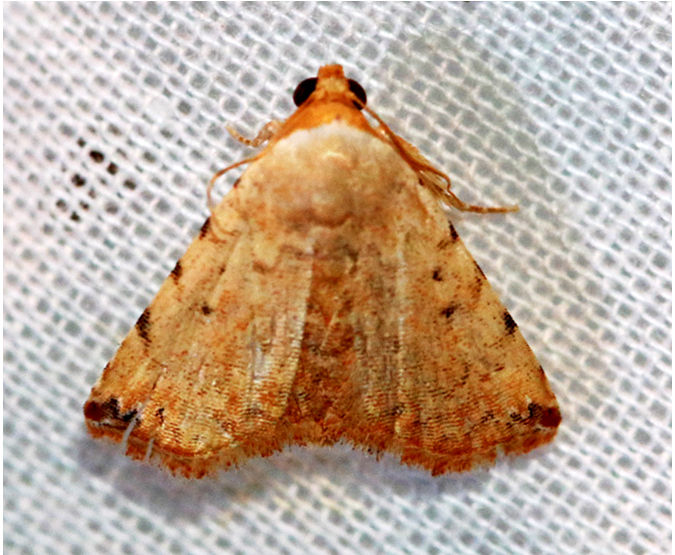
Autobasilicula

**Figure 12b.**
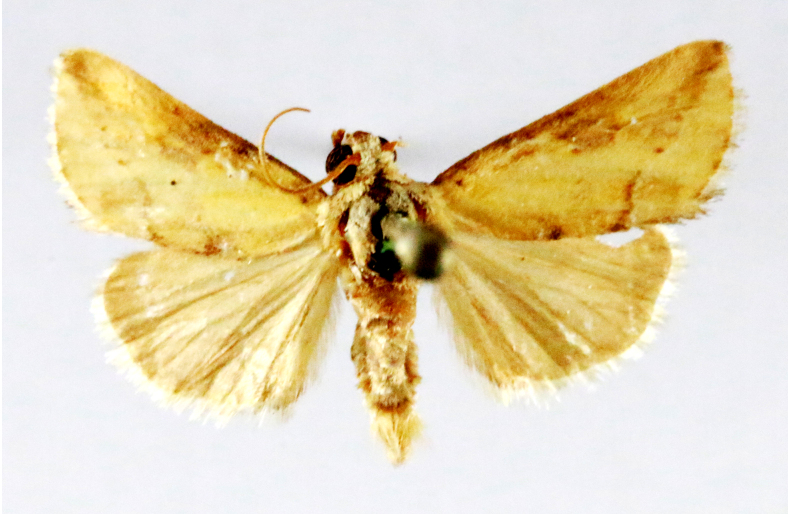
Hiccodanigripalpis

**Figure 12c.**
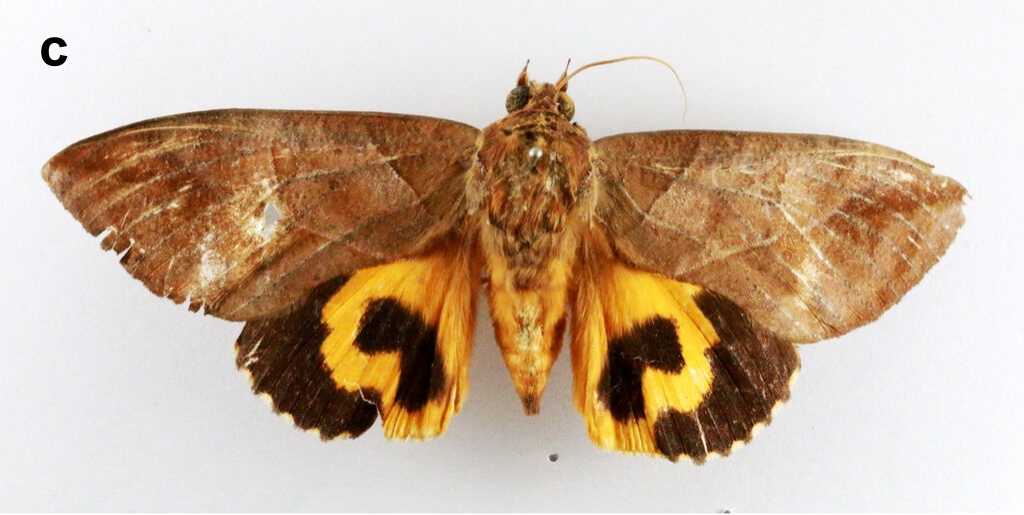
Eudocimaphalonia

**Figure 12d.**
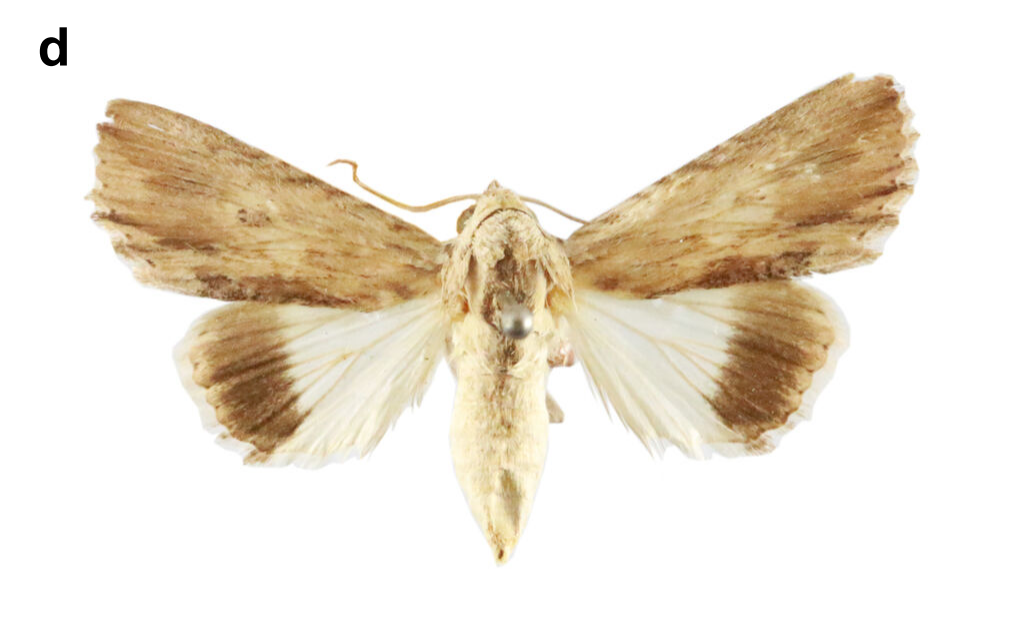
Lyncestisamphix

**Figure 12e.**
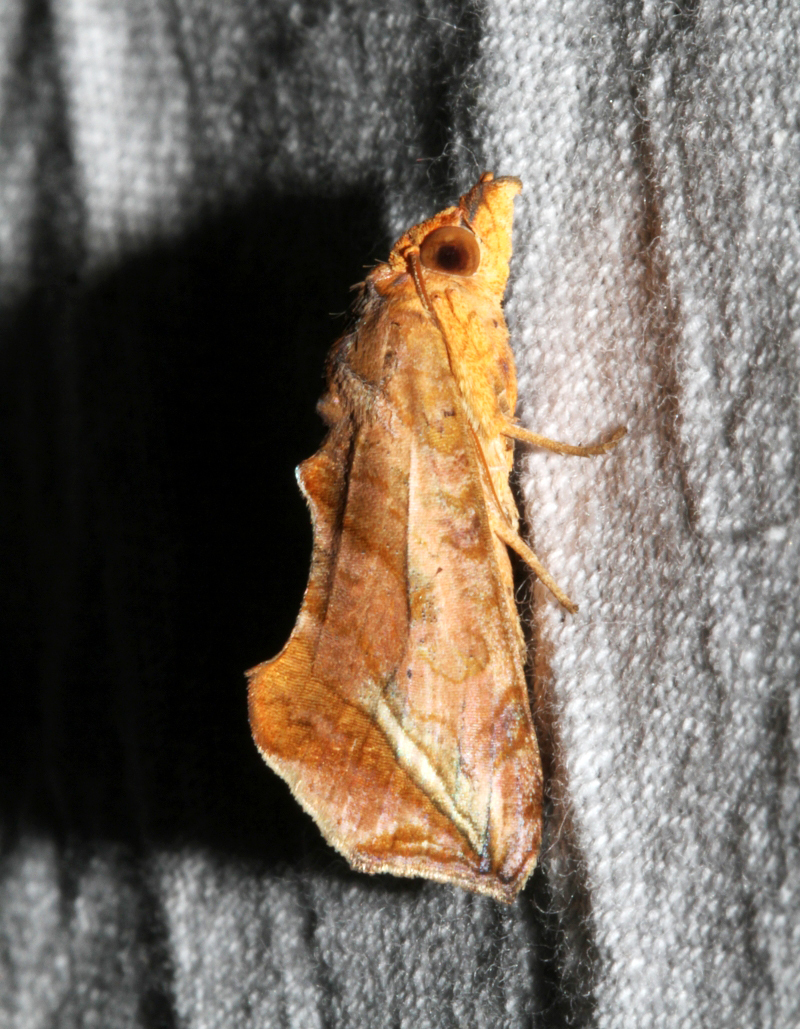
Oraesiacf.emarginata

**Figure 12f.**
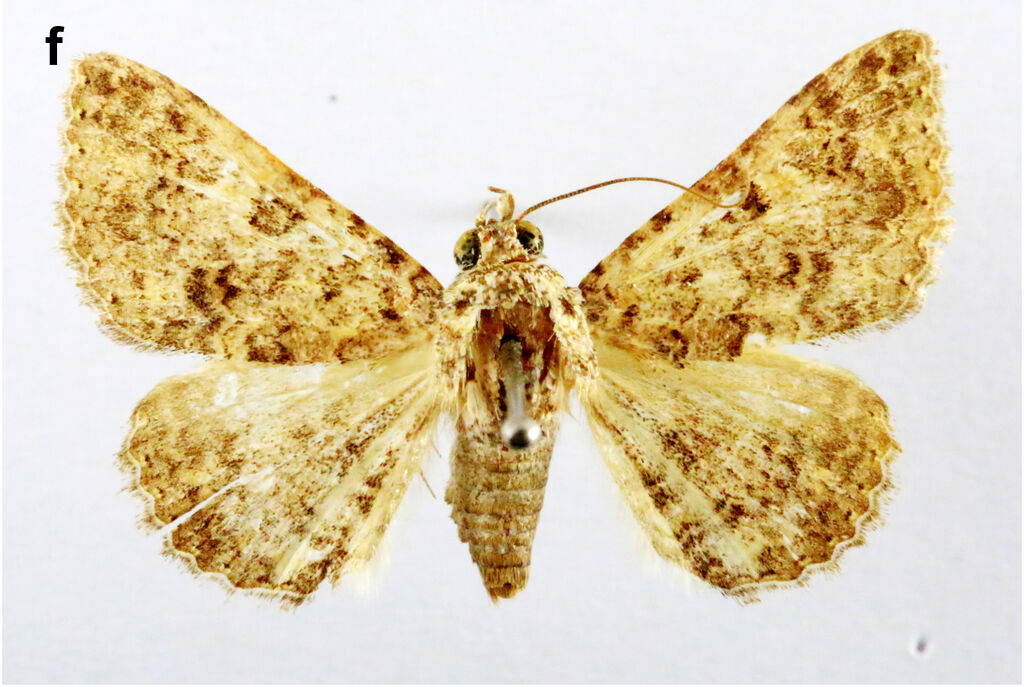
Polydesmaumbricola

**Figure 13a.**
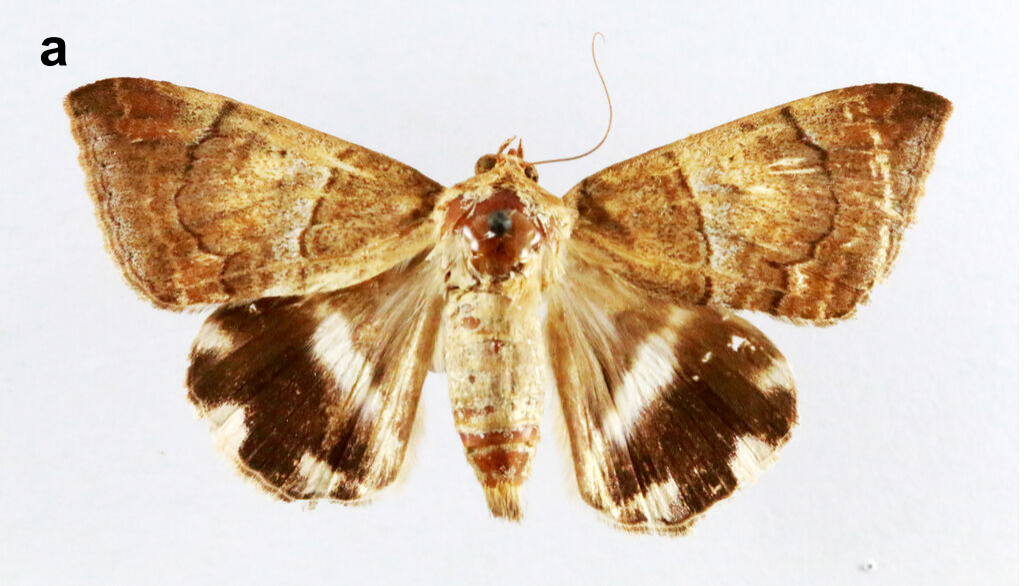
Achaeajanata

**Figure 13b.**
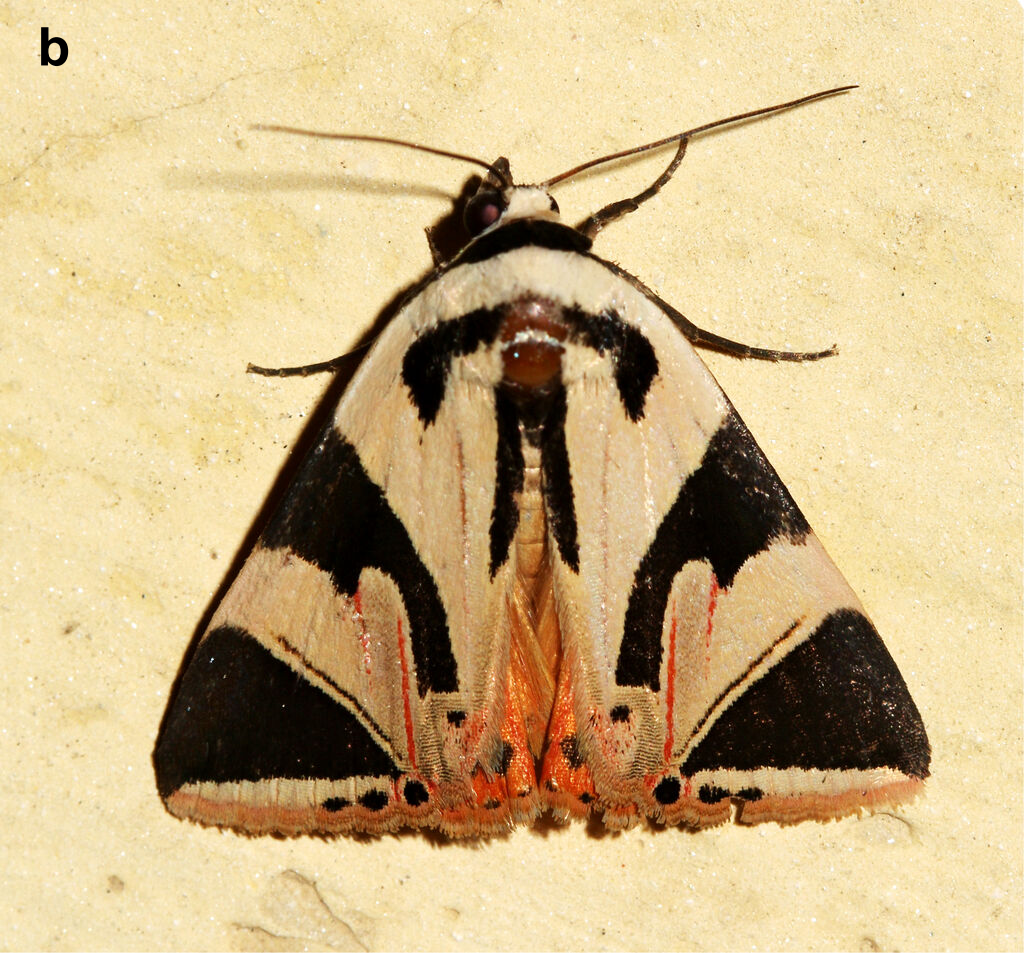
Attathaino

**Figure 13c.**
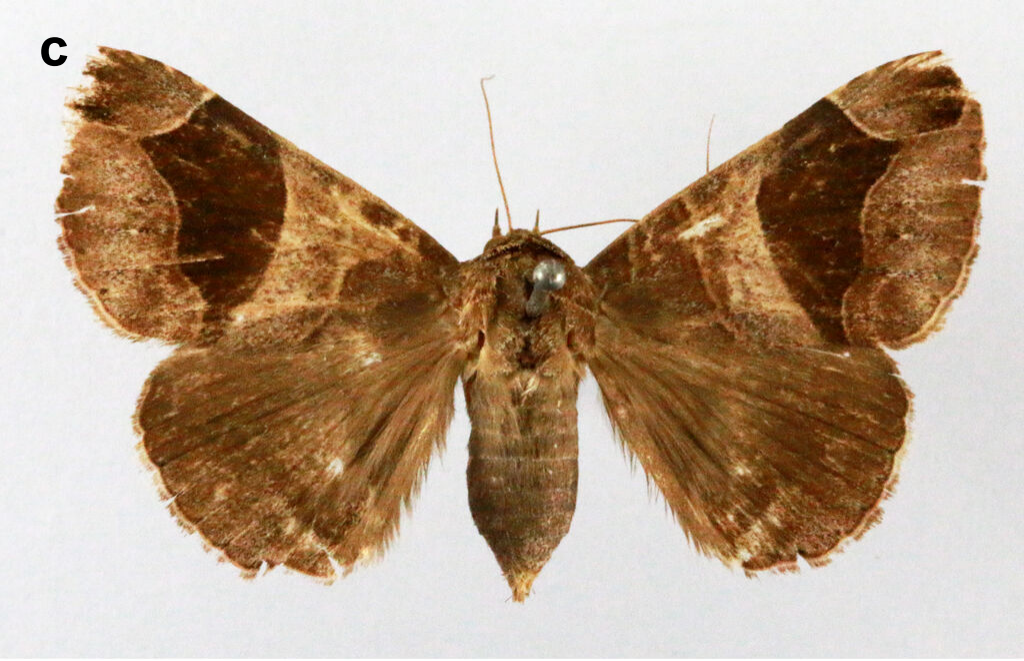
Bastillajoviania

**Figure 13d.**
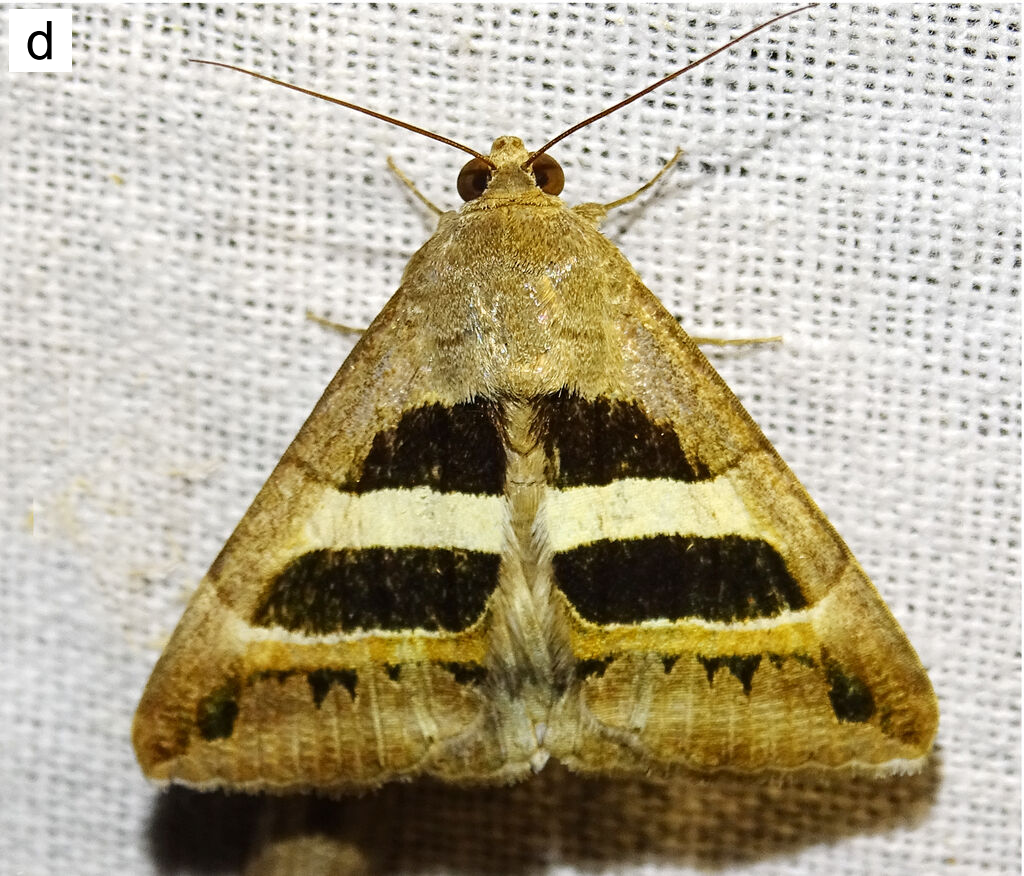
Buzaraonelia

**Figure 13e.**
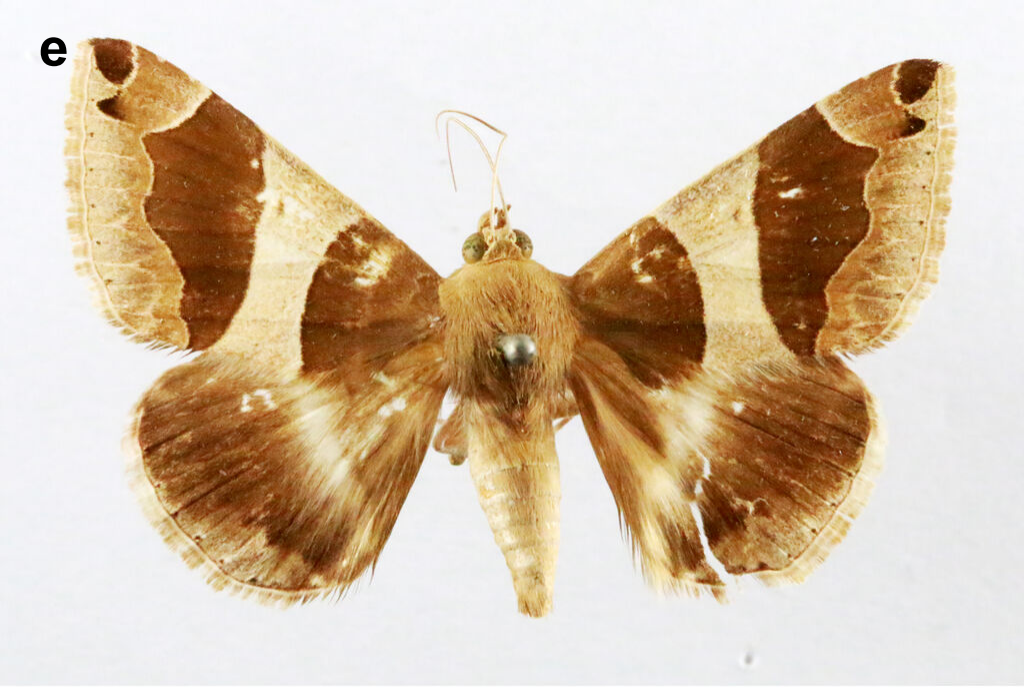
Dysgonianr.torrida

**Figure 13f.**
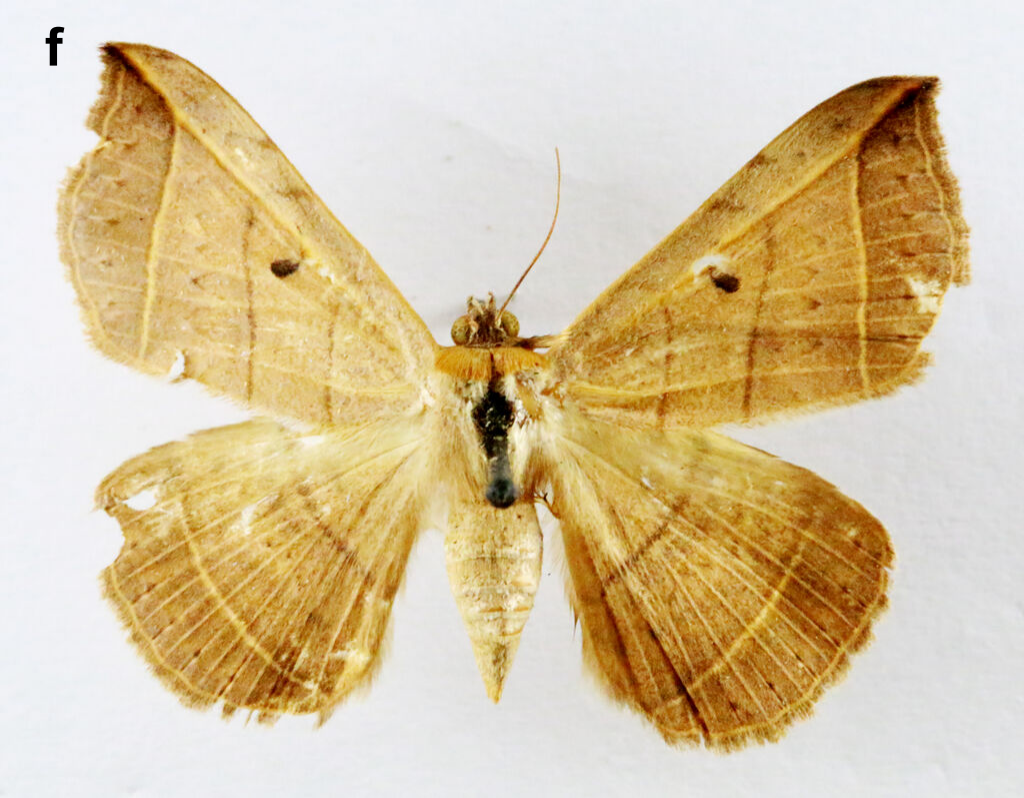
Entomogrammatorsa

**Figure 14a.**
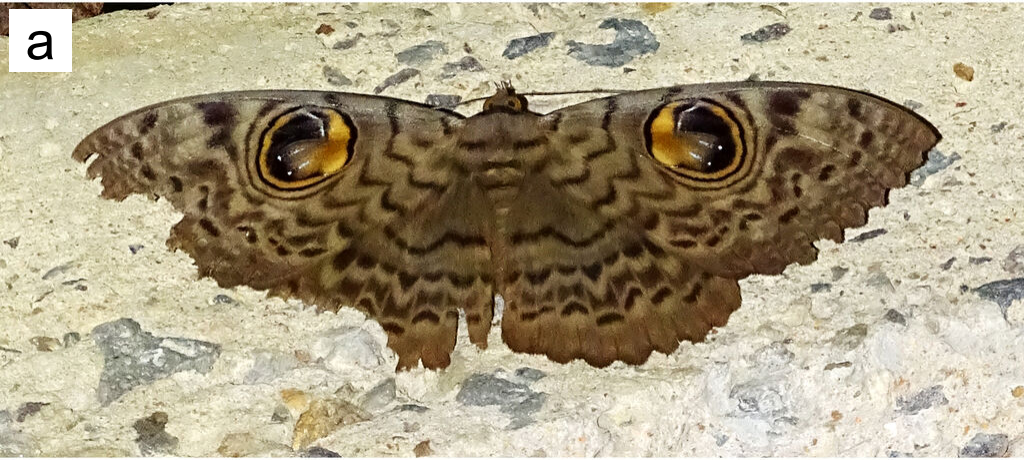
Erebusmacrops

**Figure 14b.**
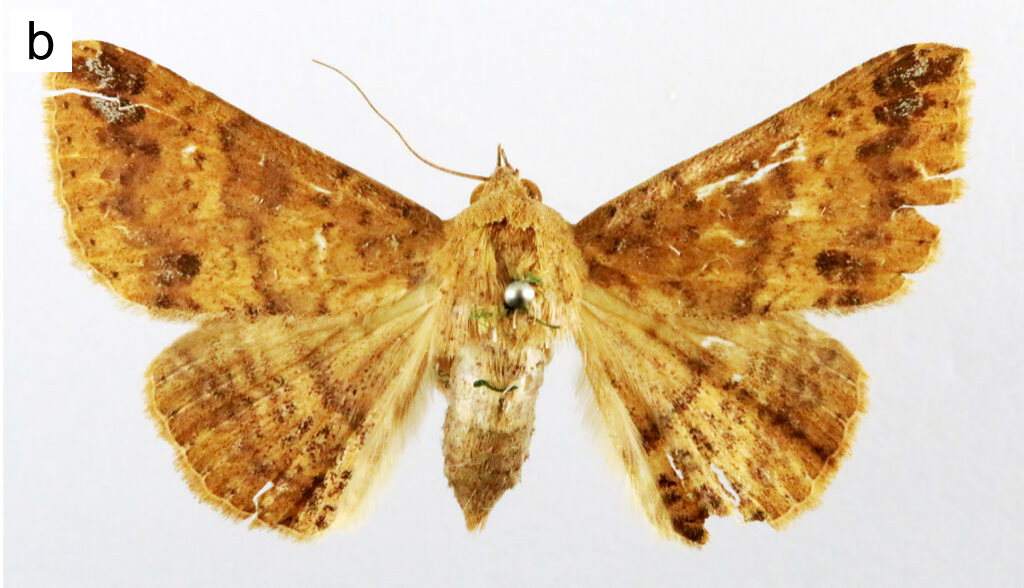
Ericeiainangulata

**Figure 14c.**
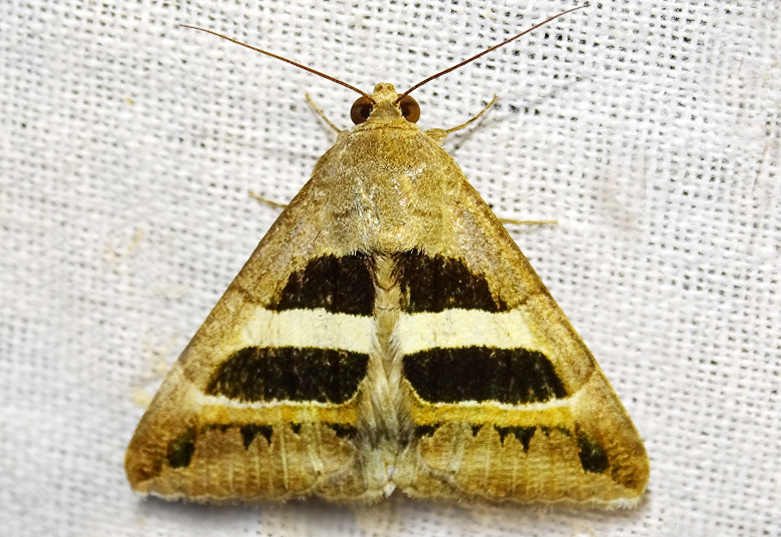
Grammodesgeometrica

**Figure 14d.**
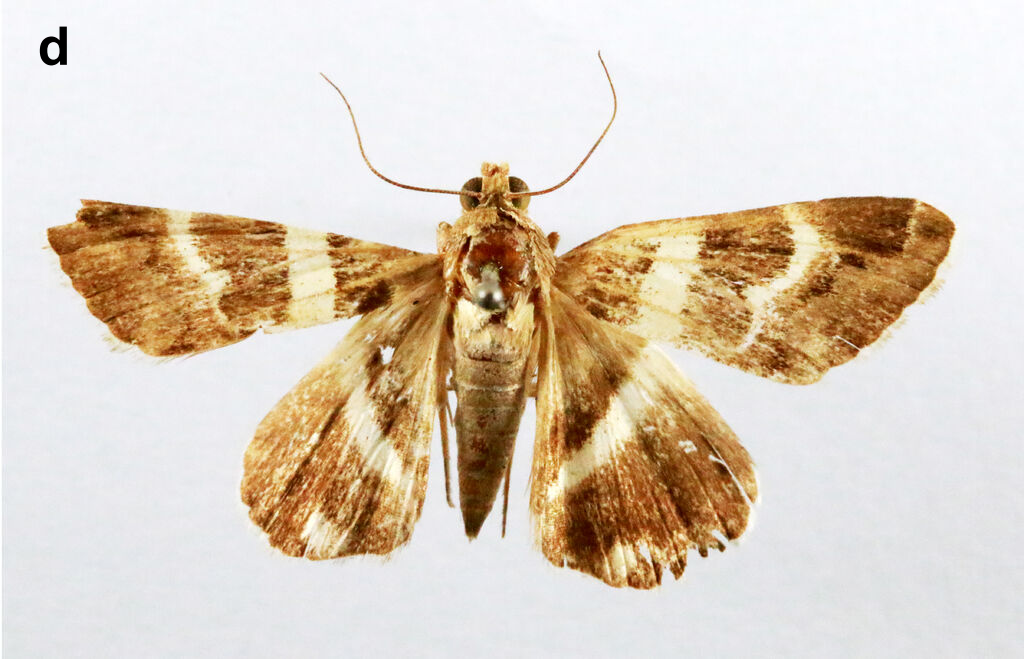
Grammodesstolida

**Figure 14e.**
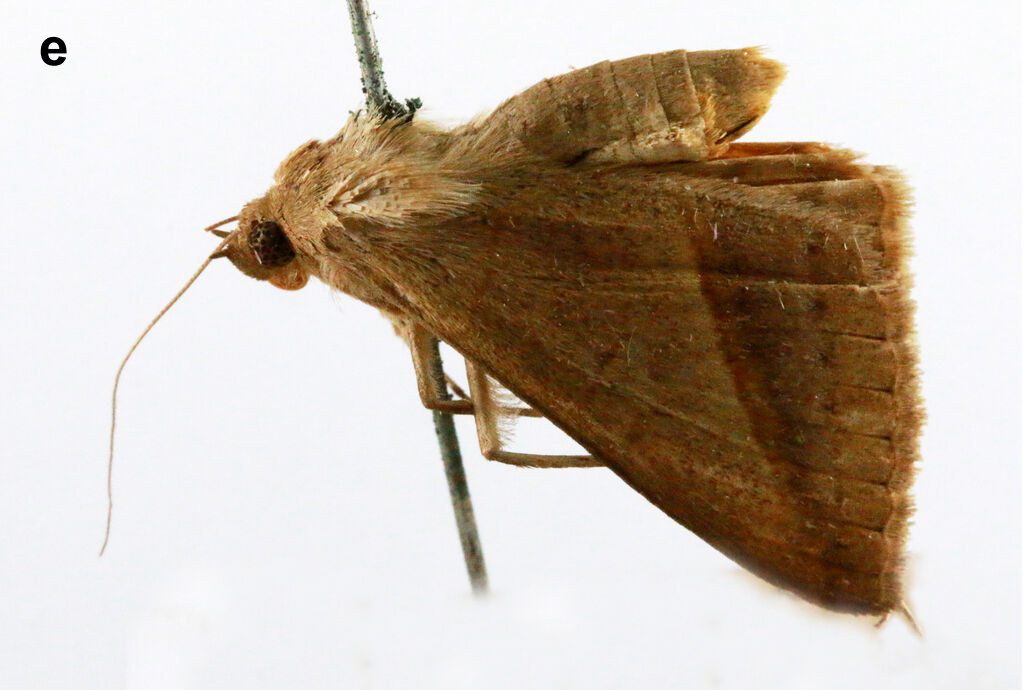
Mocisfrugalis

**Figure 14f.**
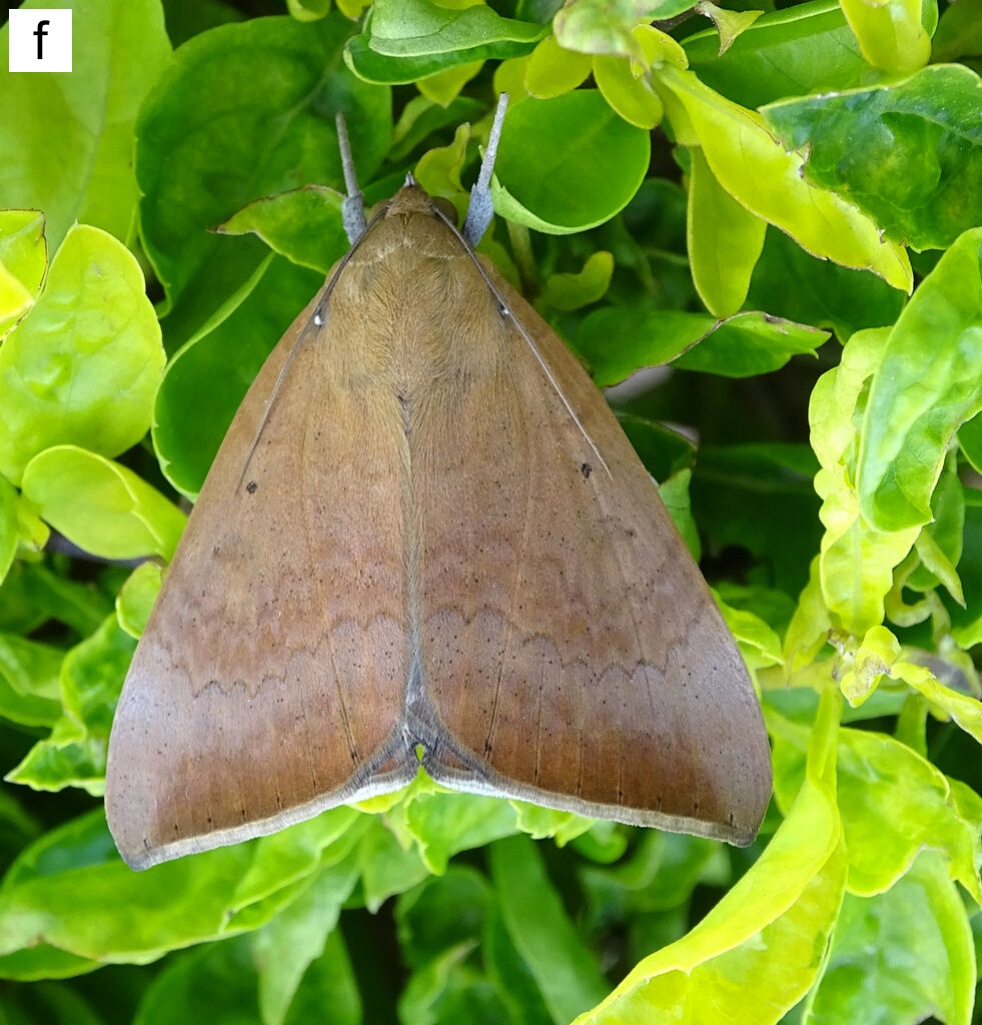
Ophismagravata

**Figure 15a.**
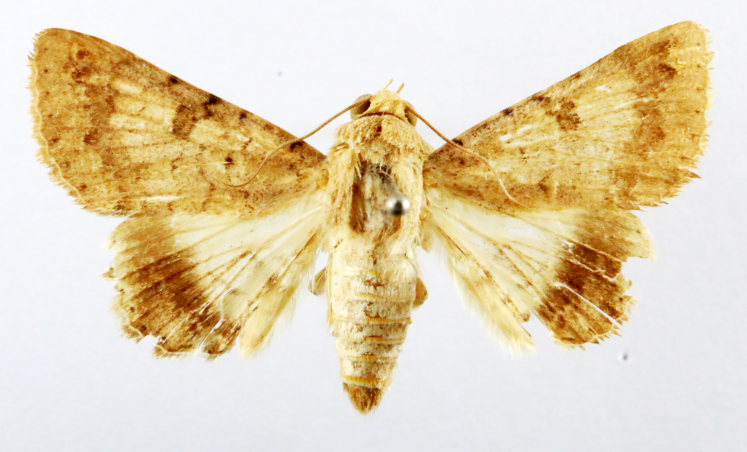
Pandesmaanysa

**Figure 15b.**
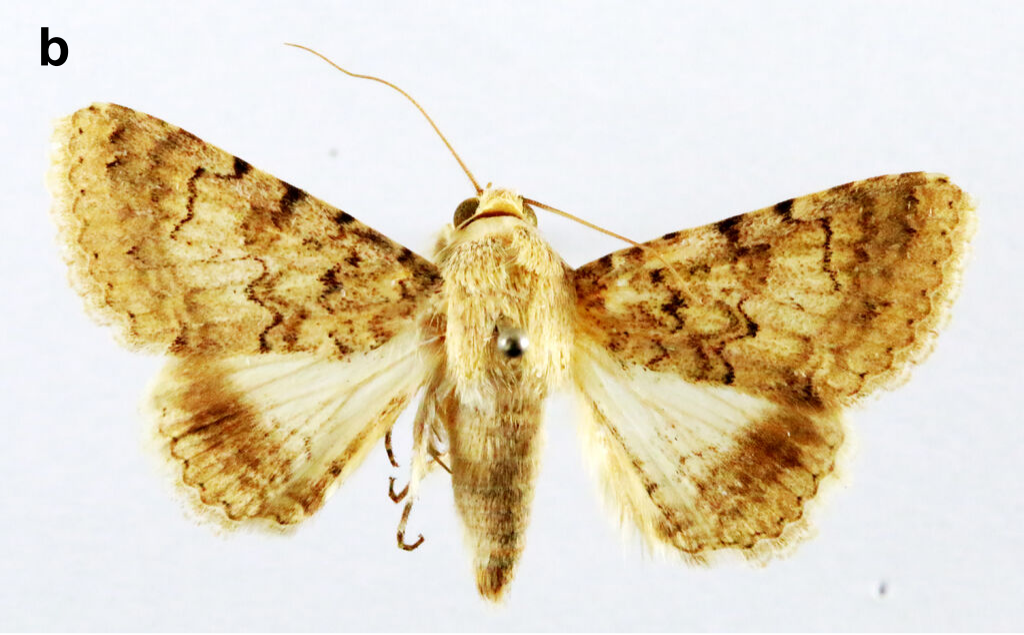
Pandesmaquenavadi

**Figure 15c.**
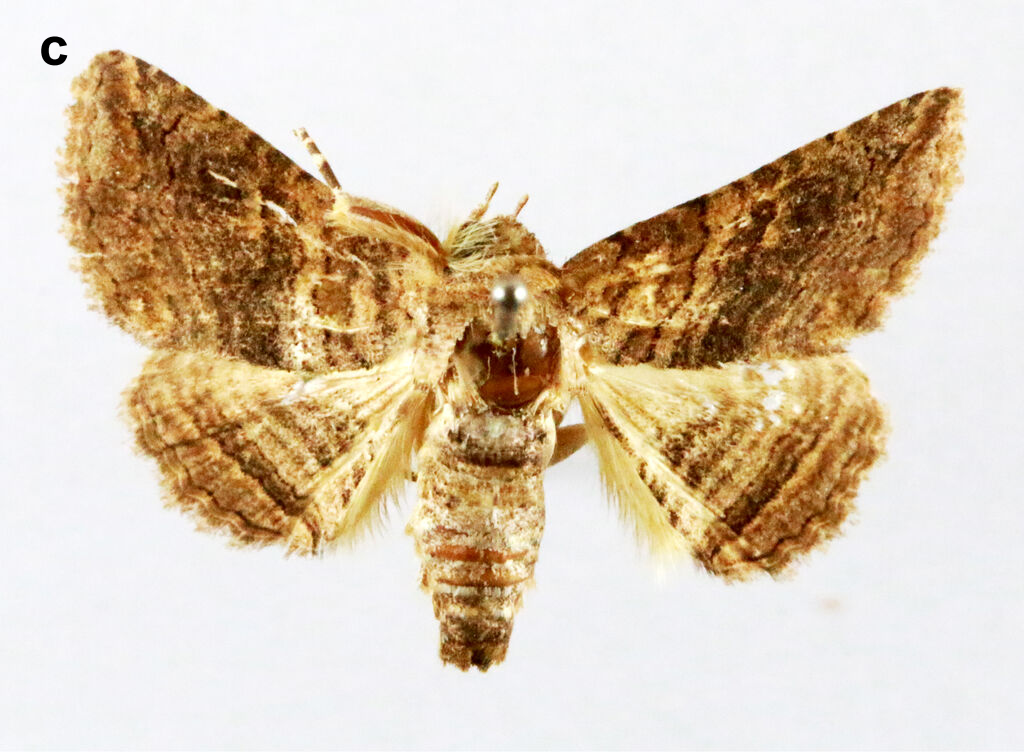
Pericymaalbidens

**Figure 15d.**
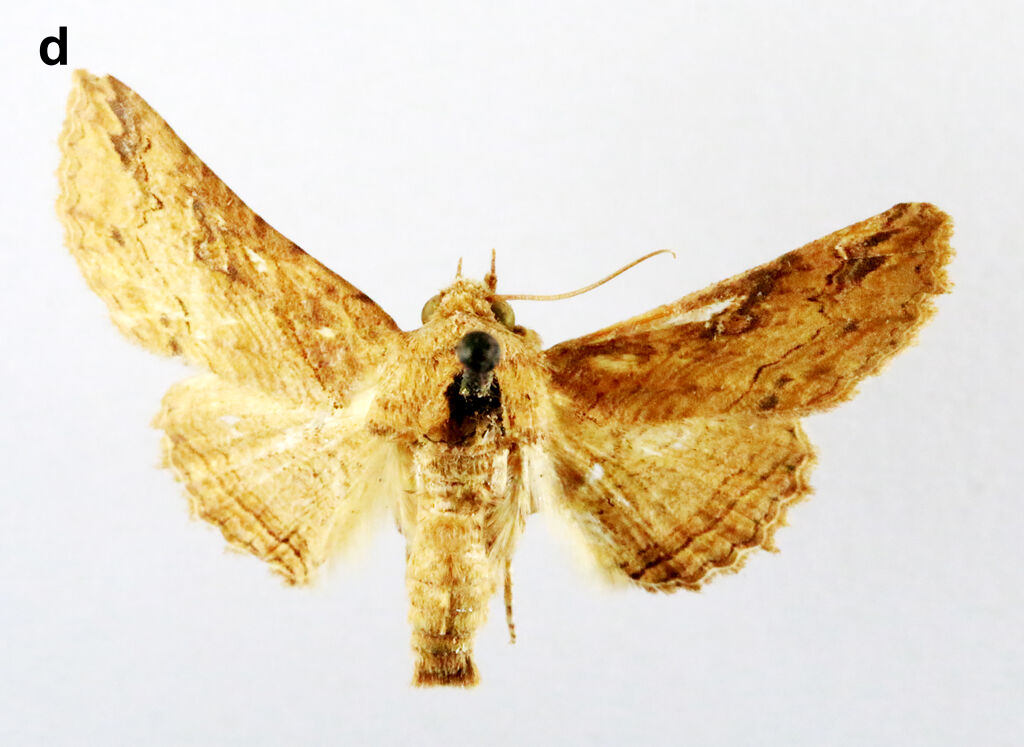
Pericymaglaucinans

**Figure 15e.**
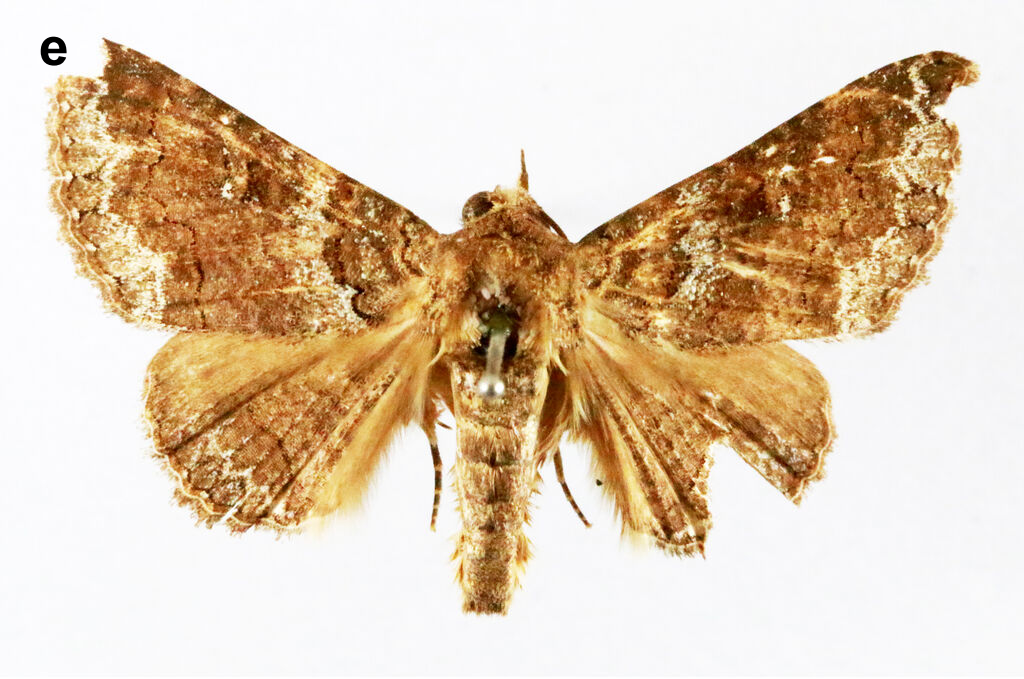
Pericymaumbrina

**Figure 15f.**
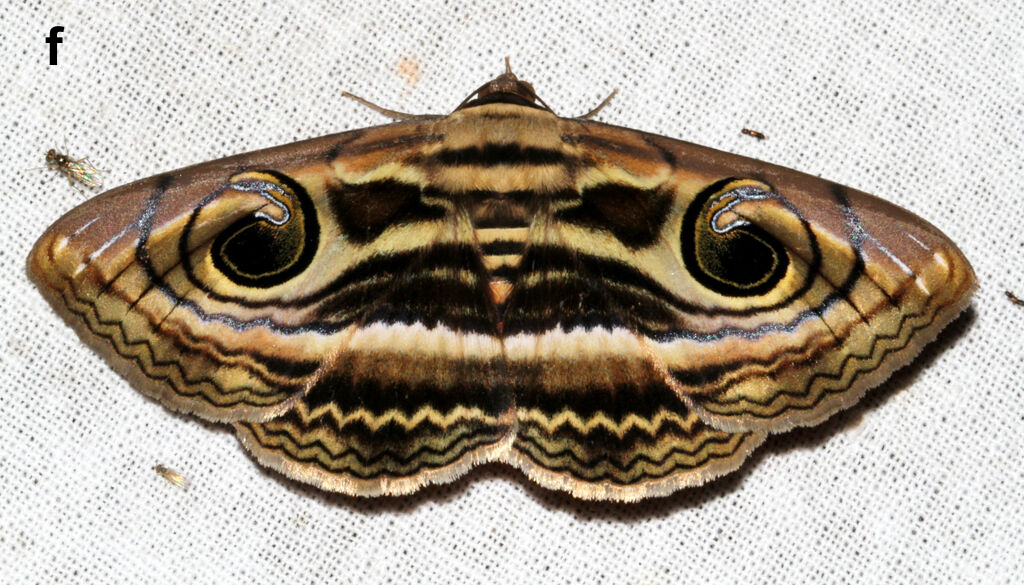
*Spirama* sp.

**Figure 16a.**
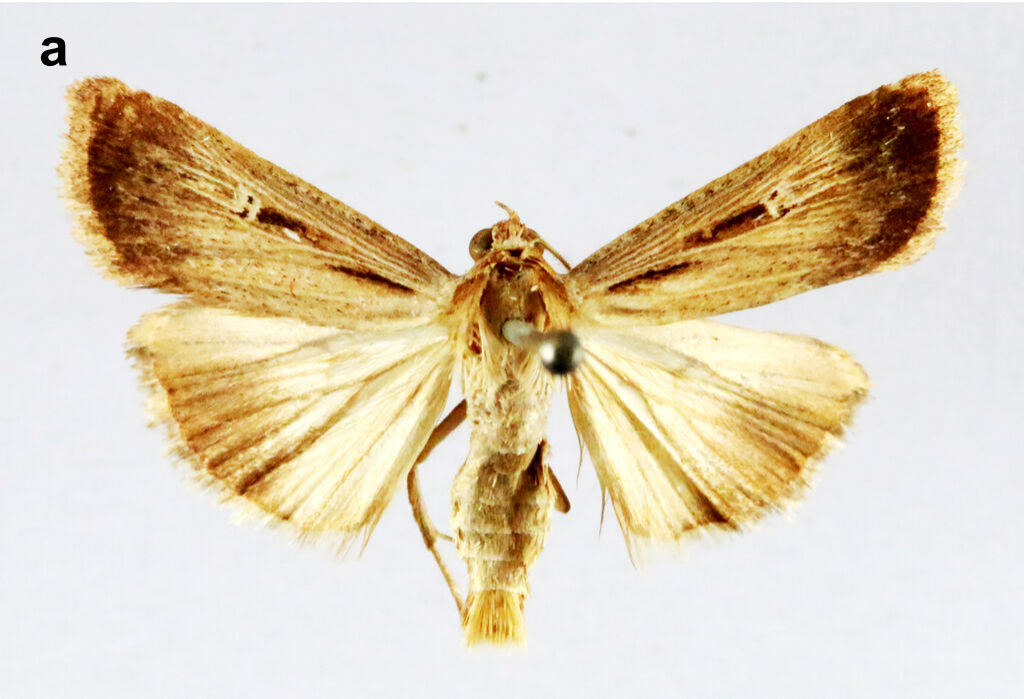
Tathorhynchusexsiccate

**Figure 16b.**
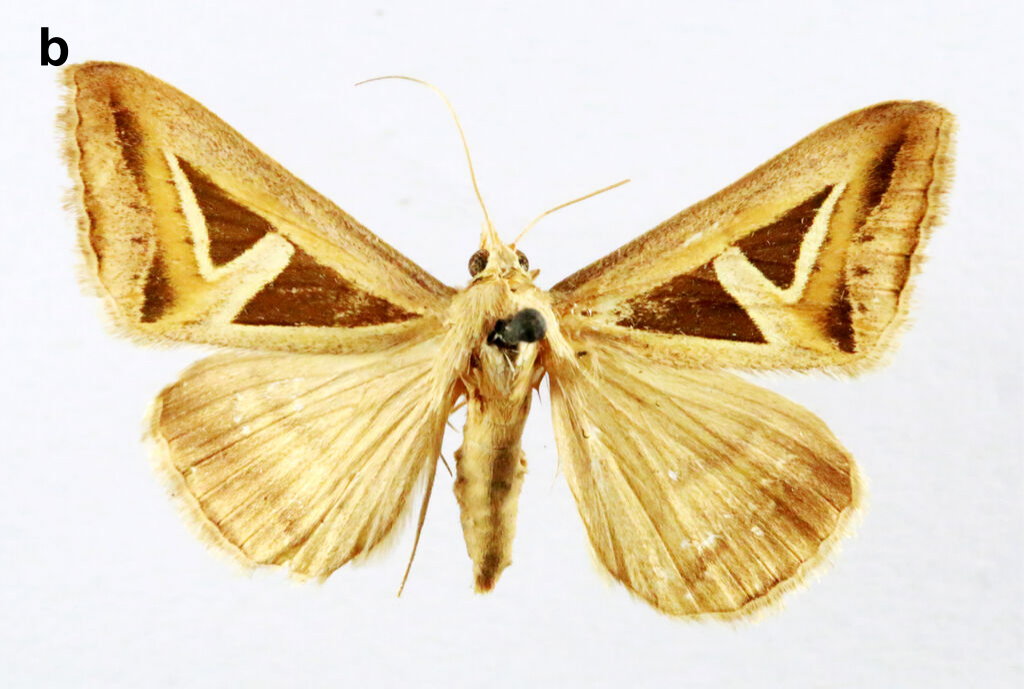
Trigonodeshyppasia

**Figure 16c.**
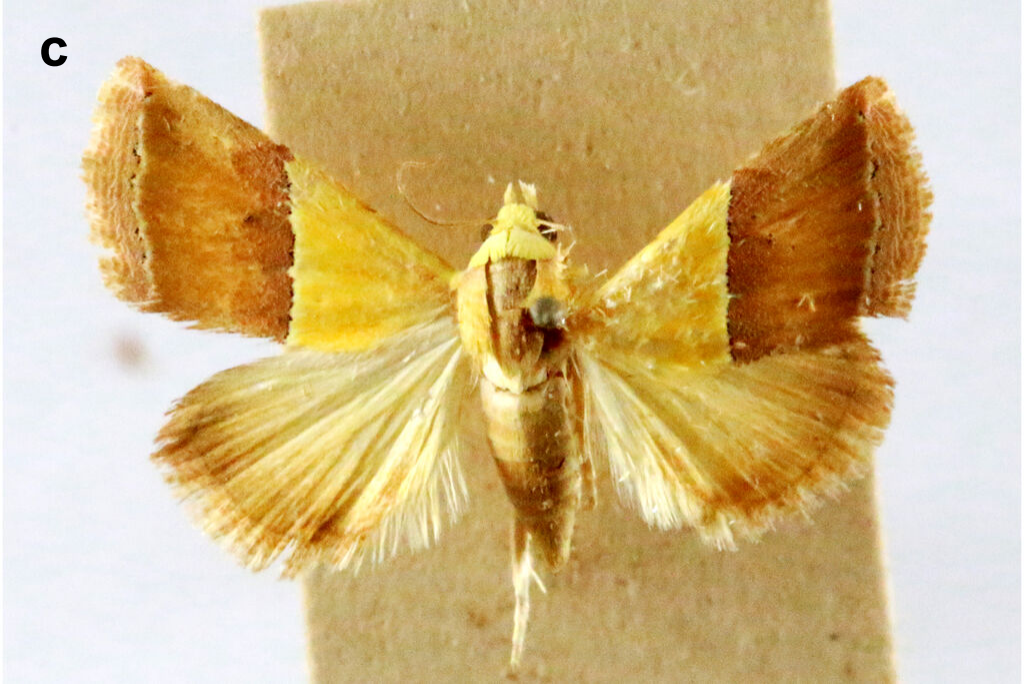
Eublemmaanachoresis

**Figure 16d.**
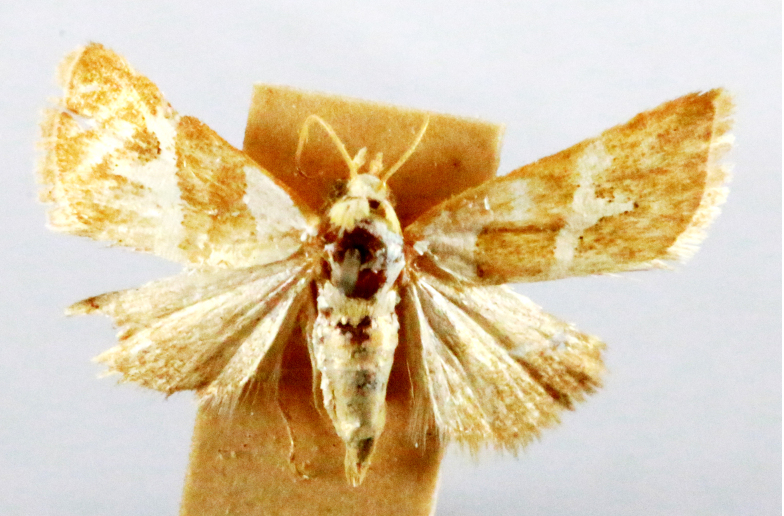
Eublemmabifasciata

**Figure 16e.**
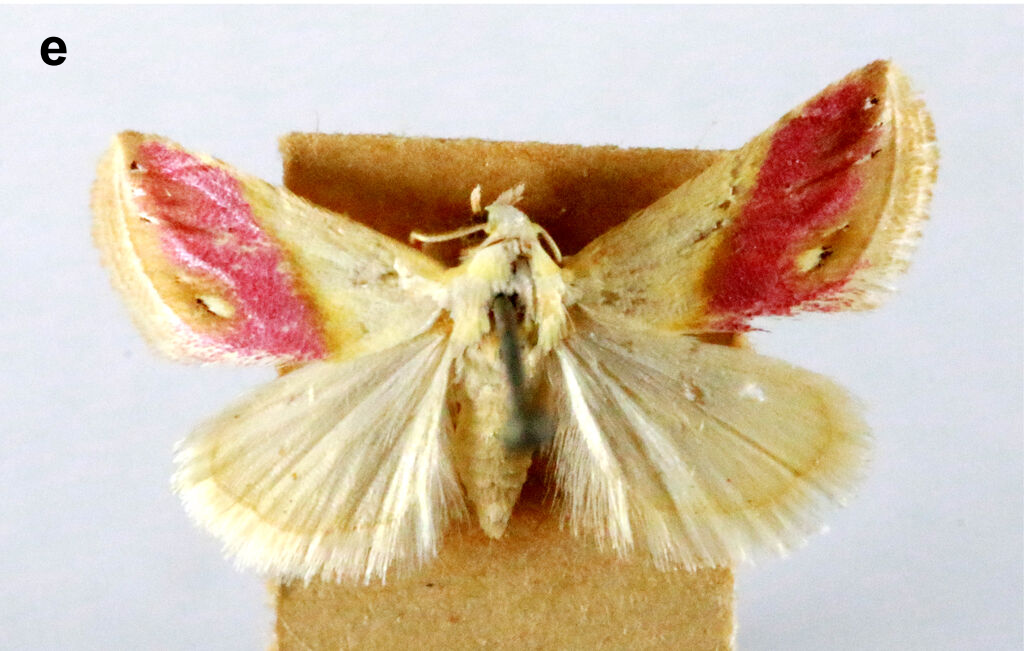
Eublemmacochylioides

**Figure 16f.**
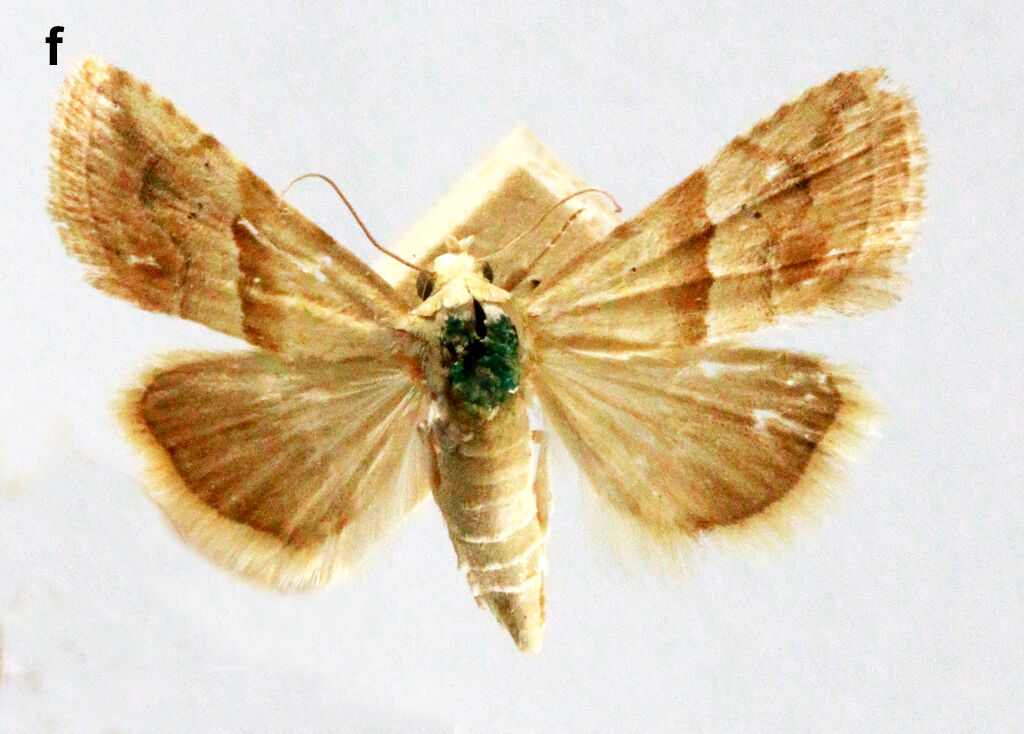
Eublemmaparva

**Figure 17a.**
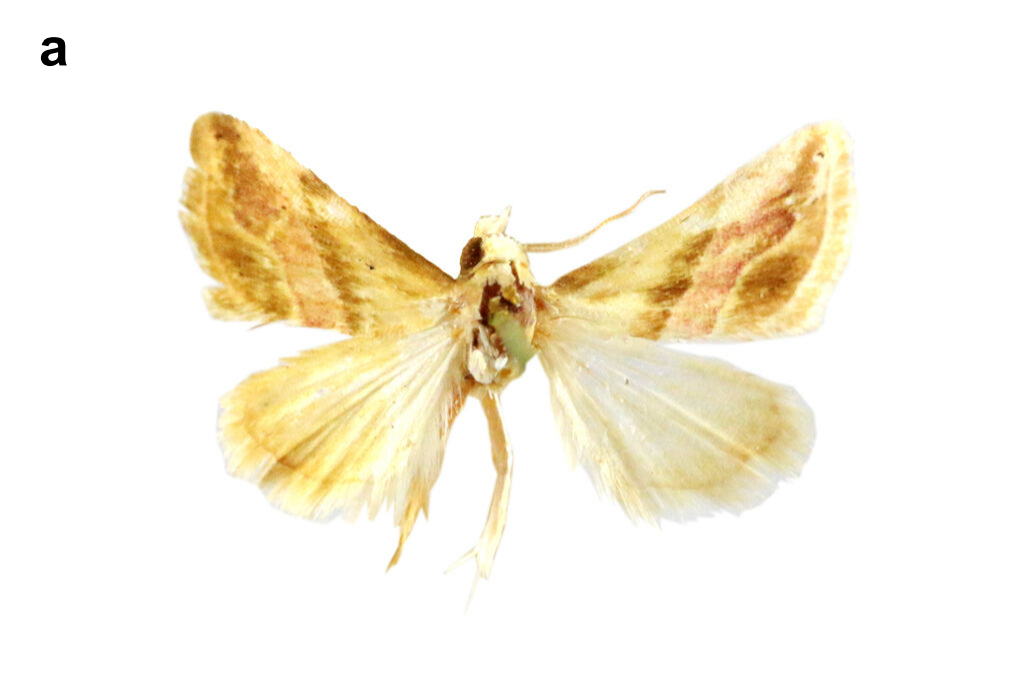
Eublemmaroseana

**Figure 17b.**
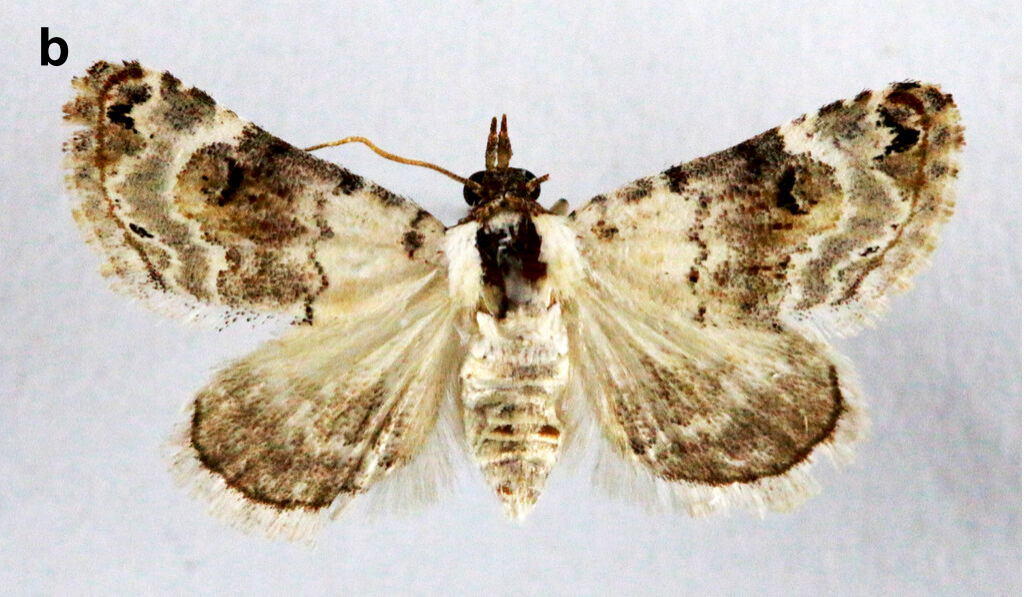
Eublemmascitula

**Figure 17c.**
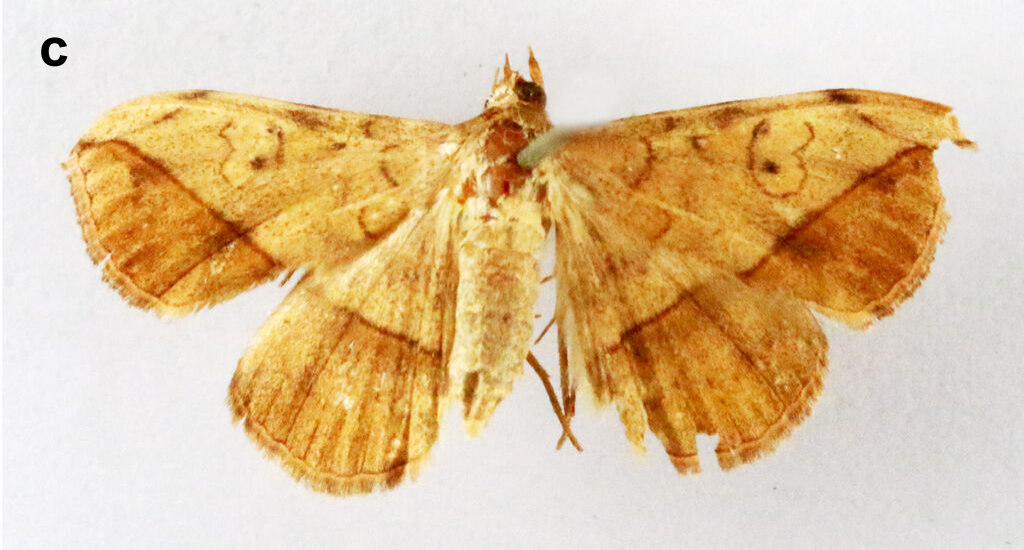
Anticarsiairrorata

**Figure 17d.**
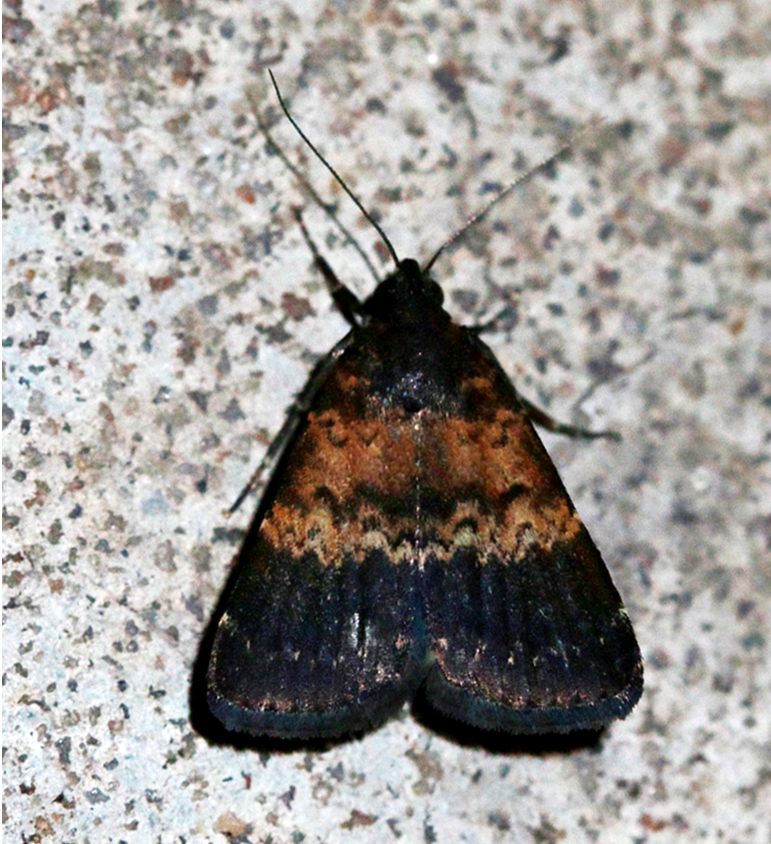
Hydrillodeslentalis

**Figure 17e.**
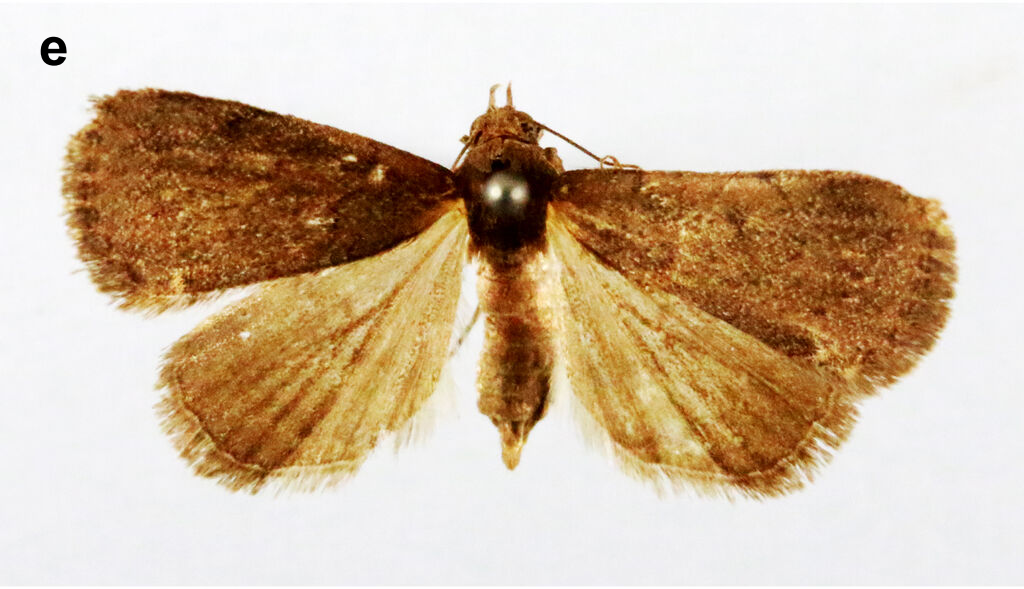
Nodariacingala

**Figure 17f.**
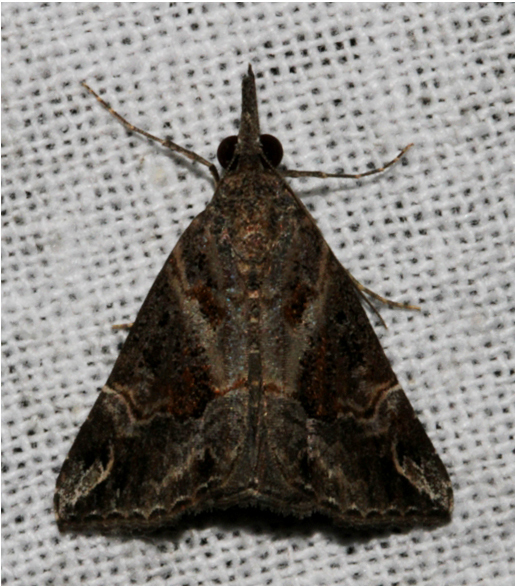
Hypenalaceratalis

**Figure 18a.**
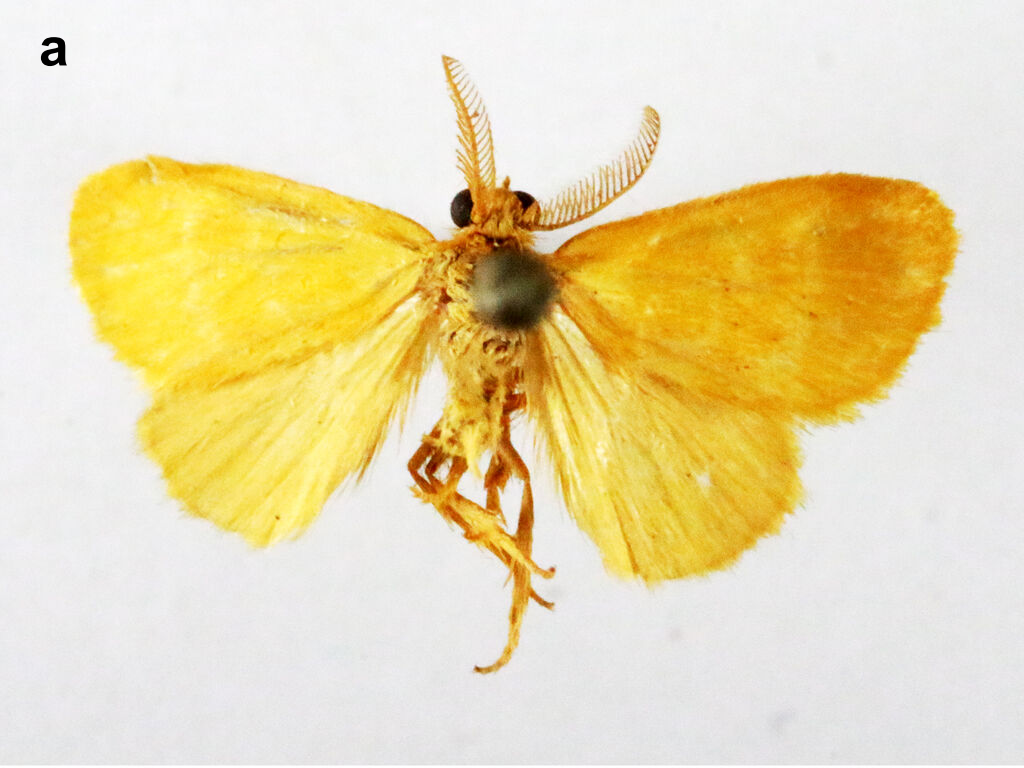
Euproctiscervina

**Figure 18b.**
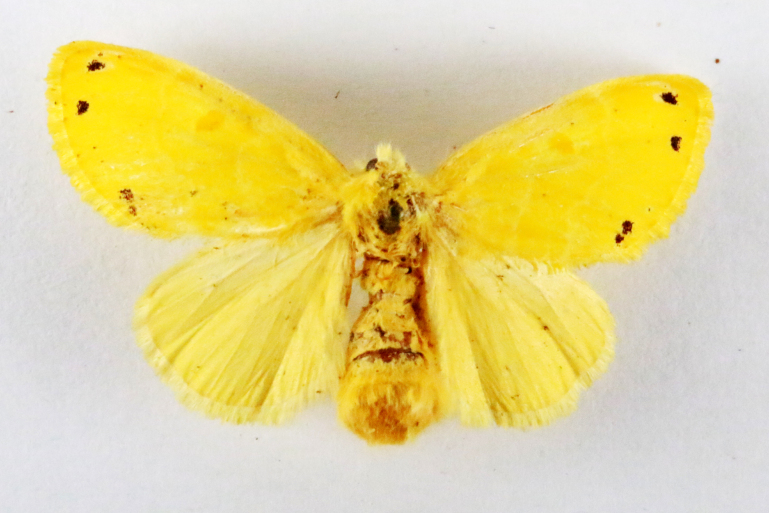
Euproctisfraterna

**Figure 18c.**
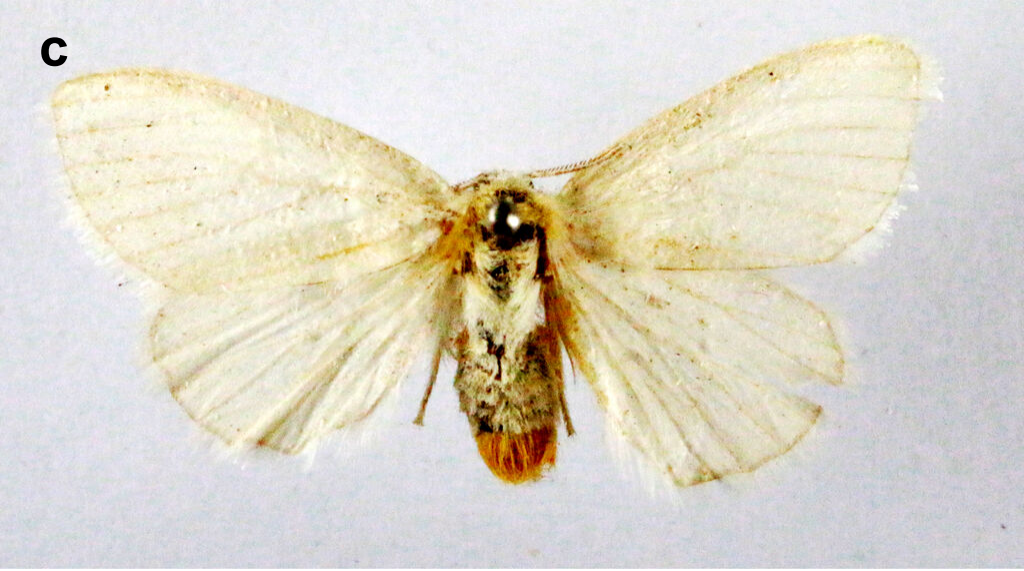
Euproctisxanthorrhoea

**Figure 18d.**
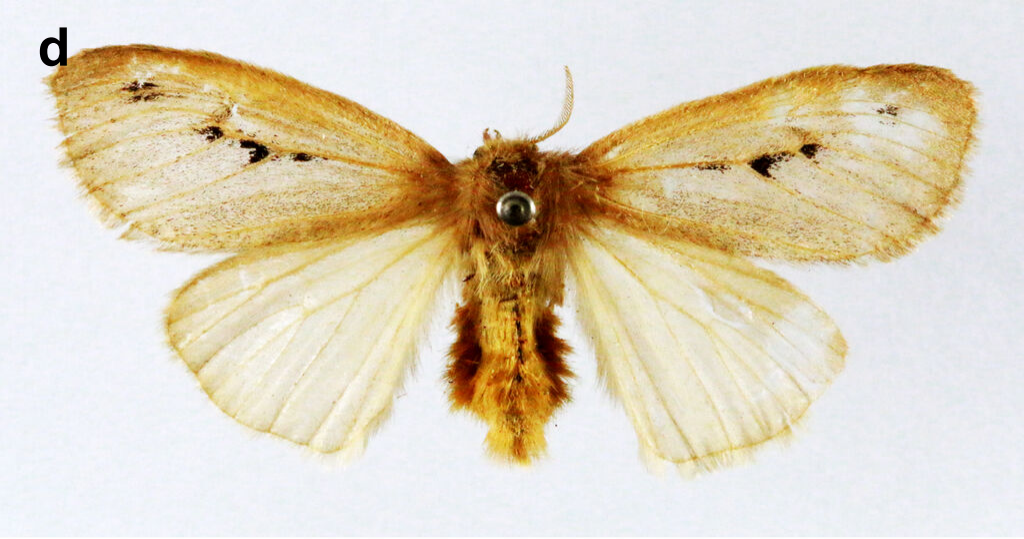
Laeliatestacea

**Figure 18e.**
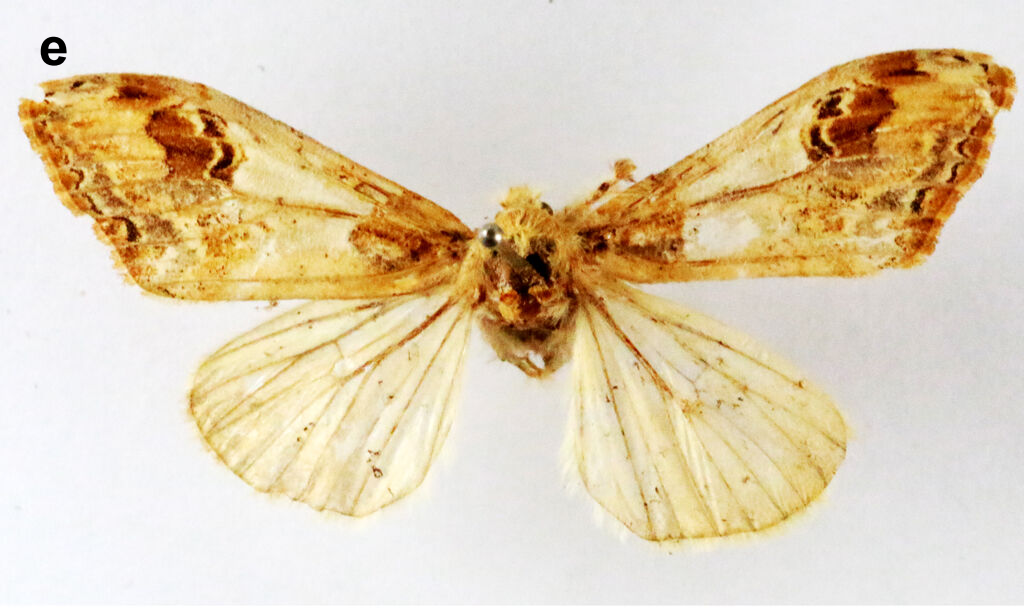
Olenemendosa

**Figure 18f.**
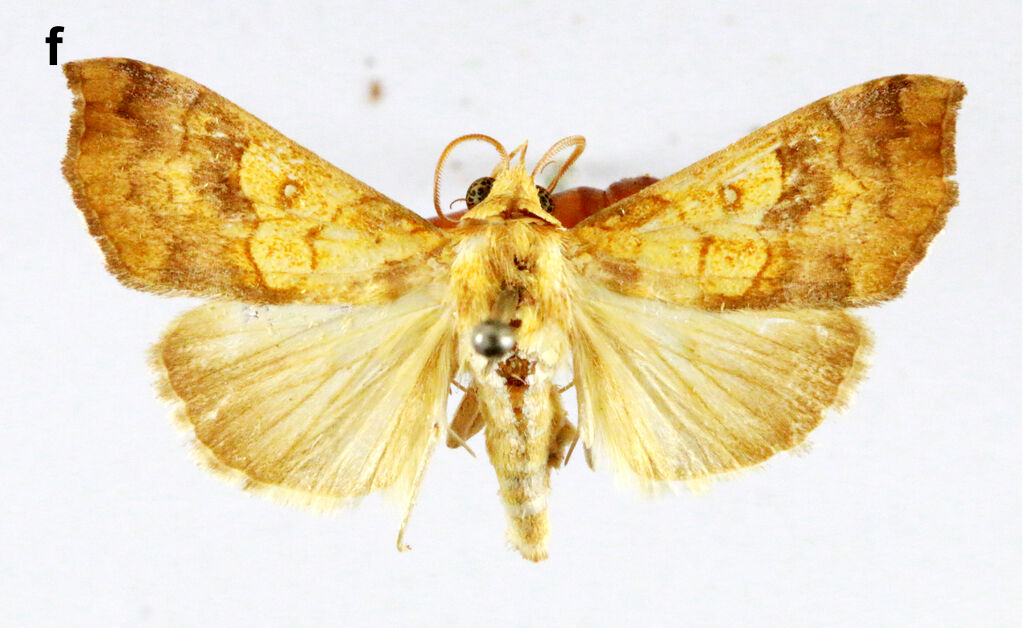
Anomisflava

**Figure 19a.**
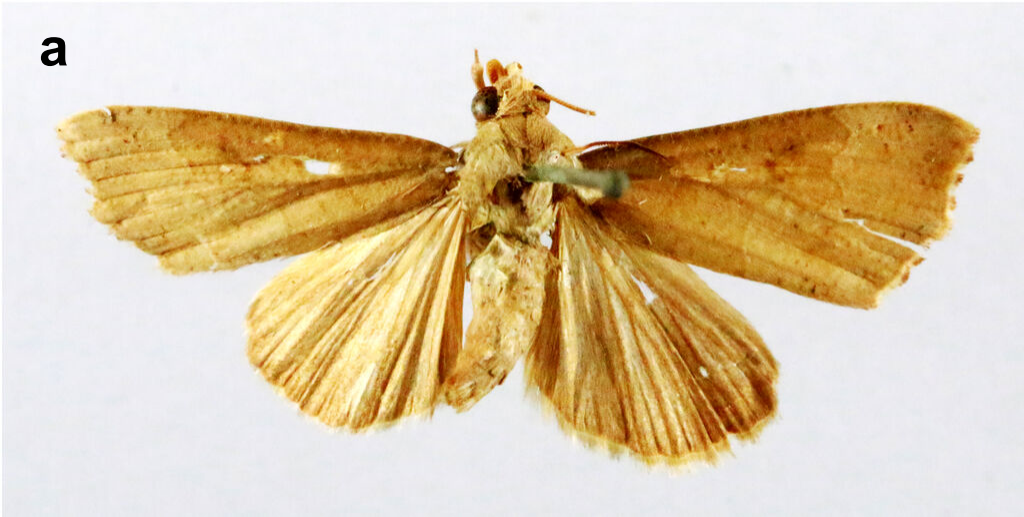
Anomisinvoluta

**Figure 19b.**
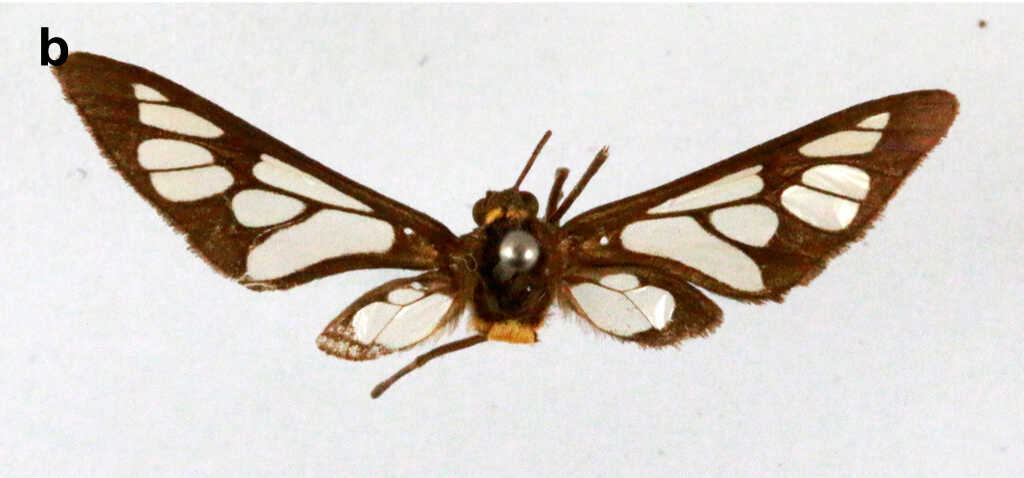
Syntomoidesimaon

**Figure 19c.**
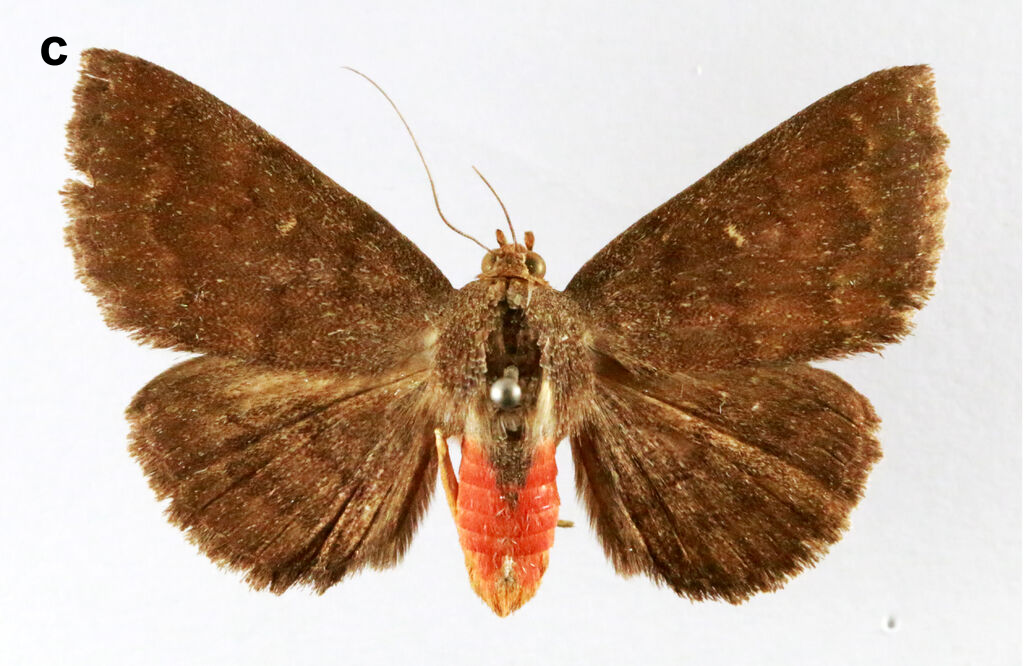
Calesiahaemorrhoa

**Figure 19d.**
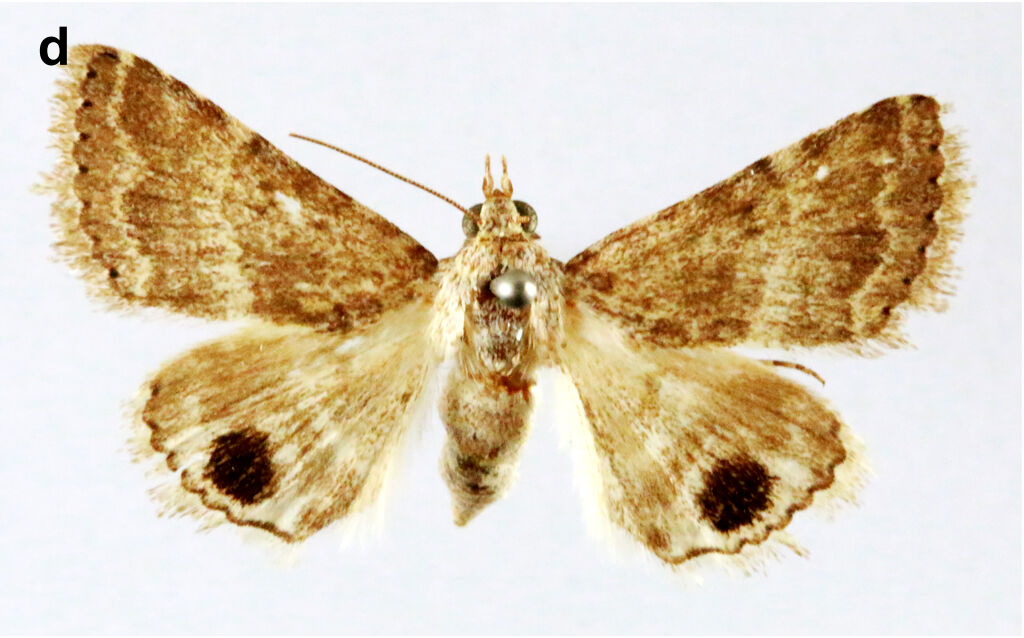
Anumetaatrosignata

**Figure 19e.**
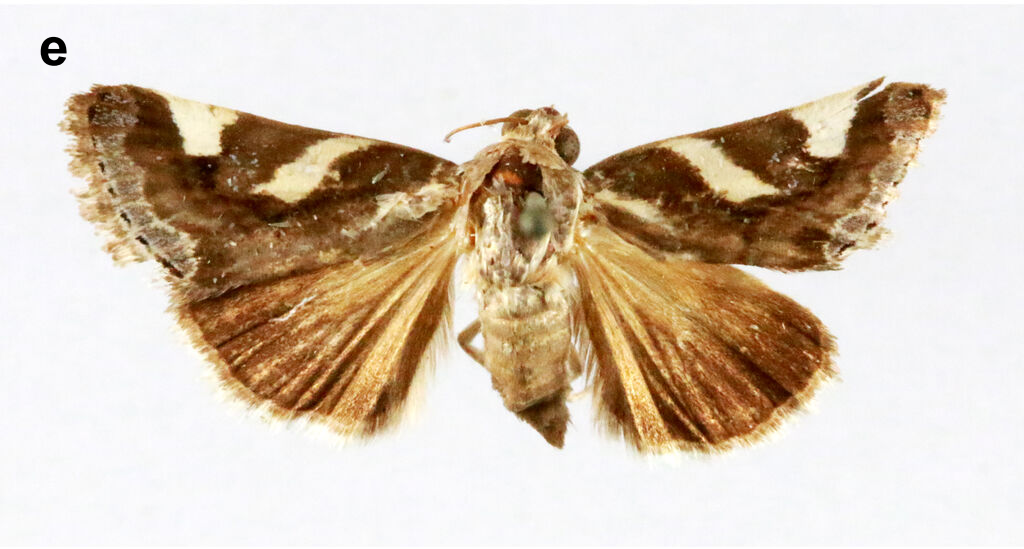
Acontiacatenula

**Figure 19f.**
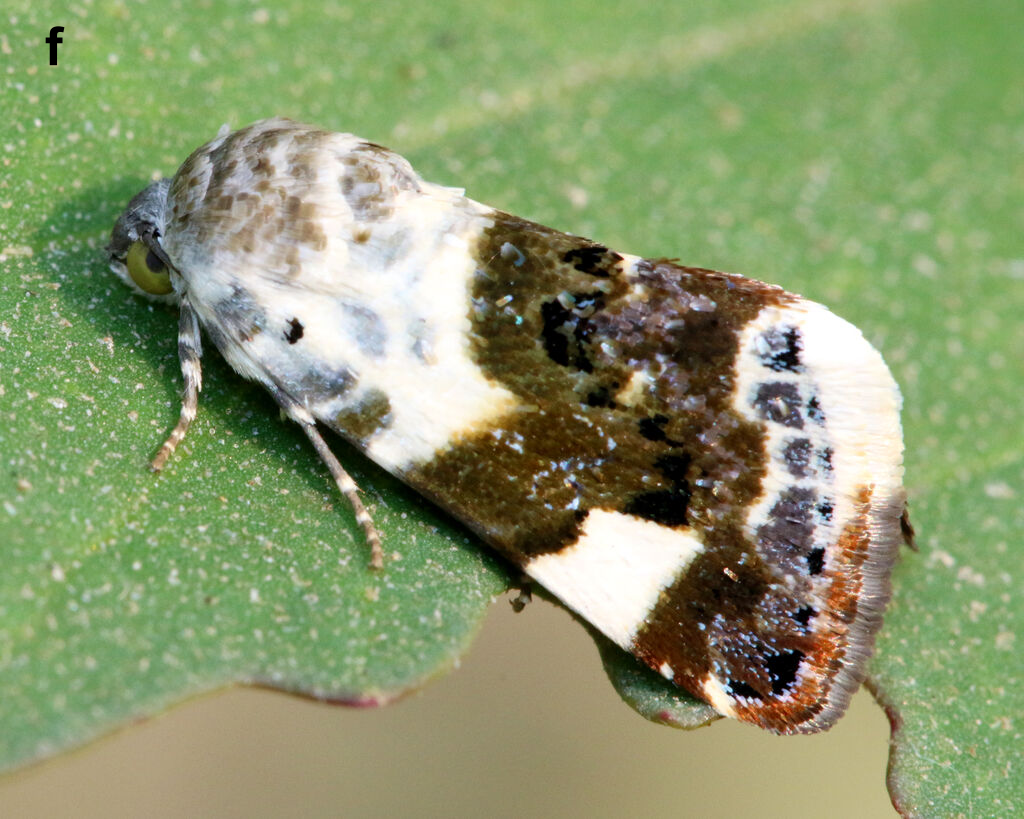
Acontialucida

**Figure 20a.**
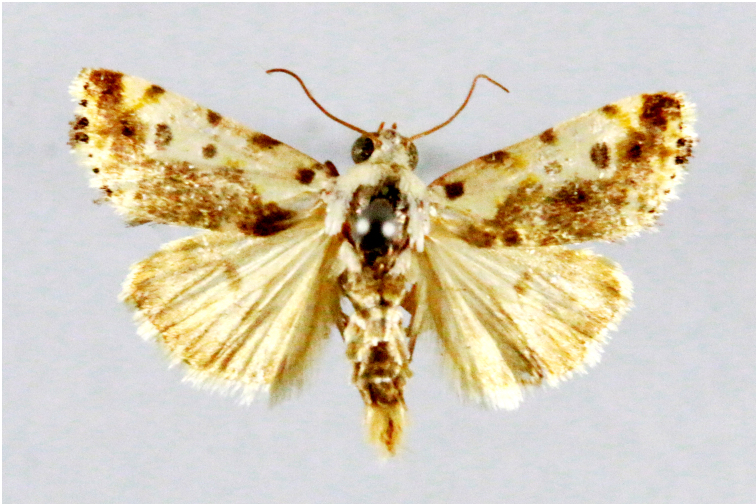
Acontiamarmoralis

**Figure 20b.**
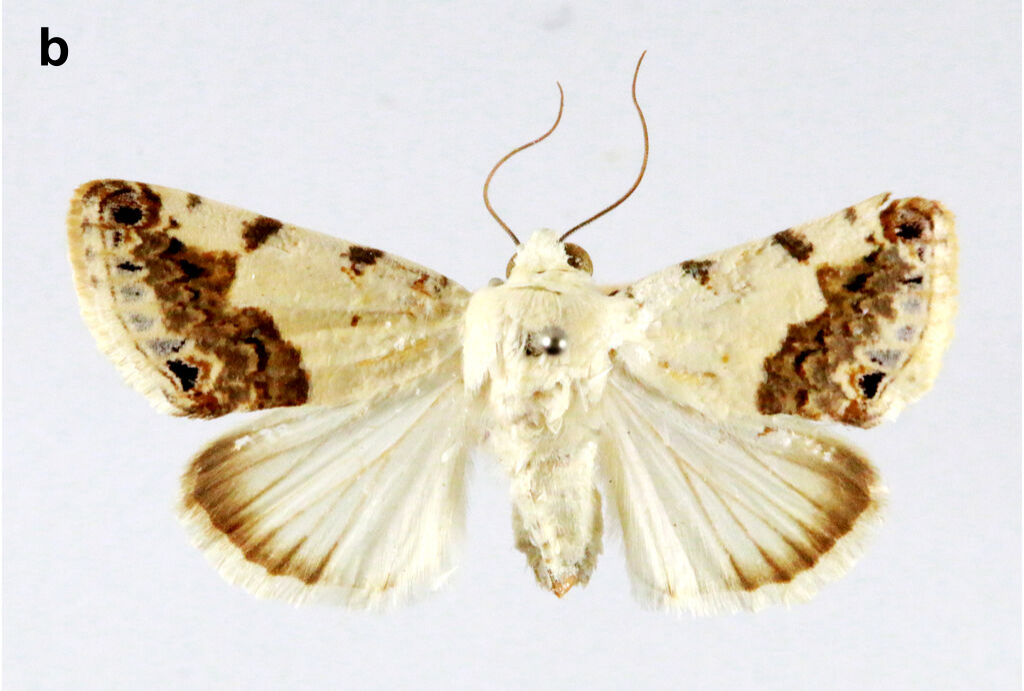
Acontianotabilis

**Figure 20c.**
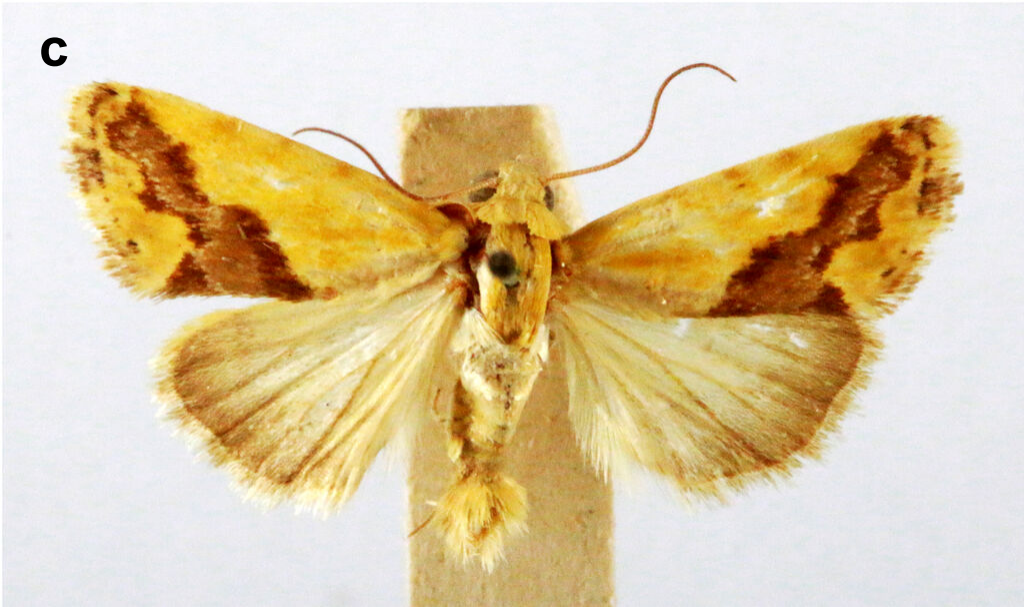
Acontiasexpunctata

**Figure 20d.**
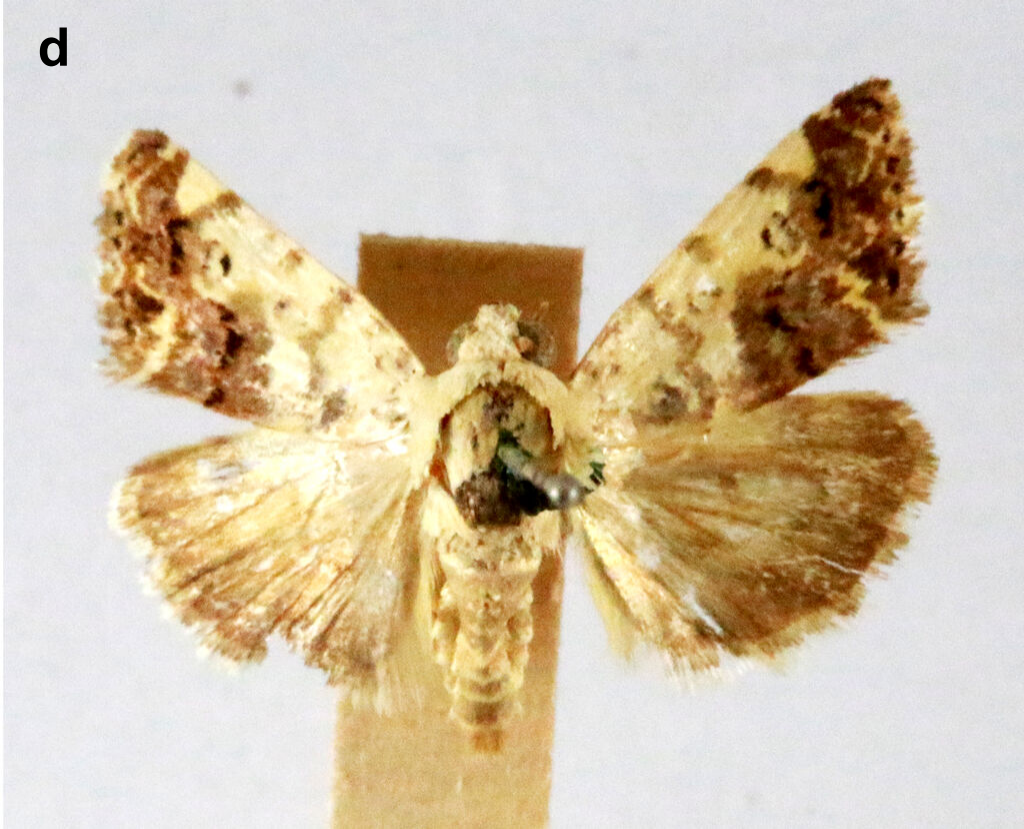
Emmeliasemipallida

**Figure 20e.**
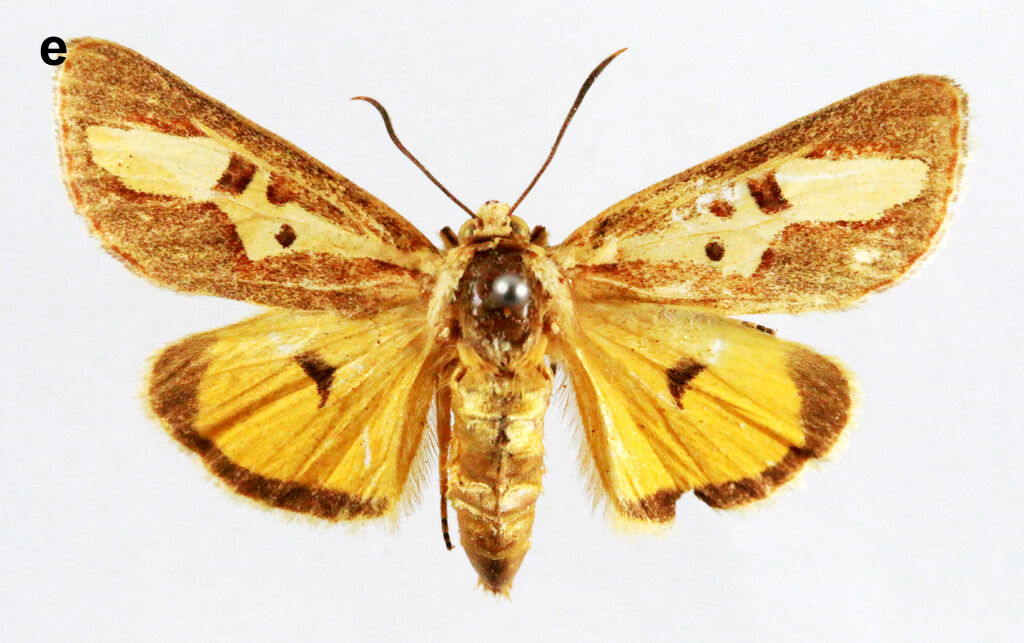
Aegoceravenulia

**Figure 20f.**
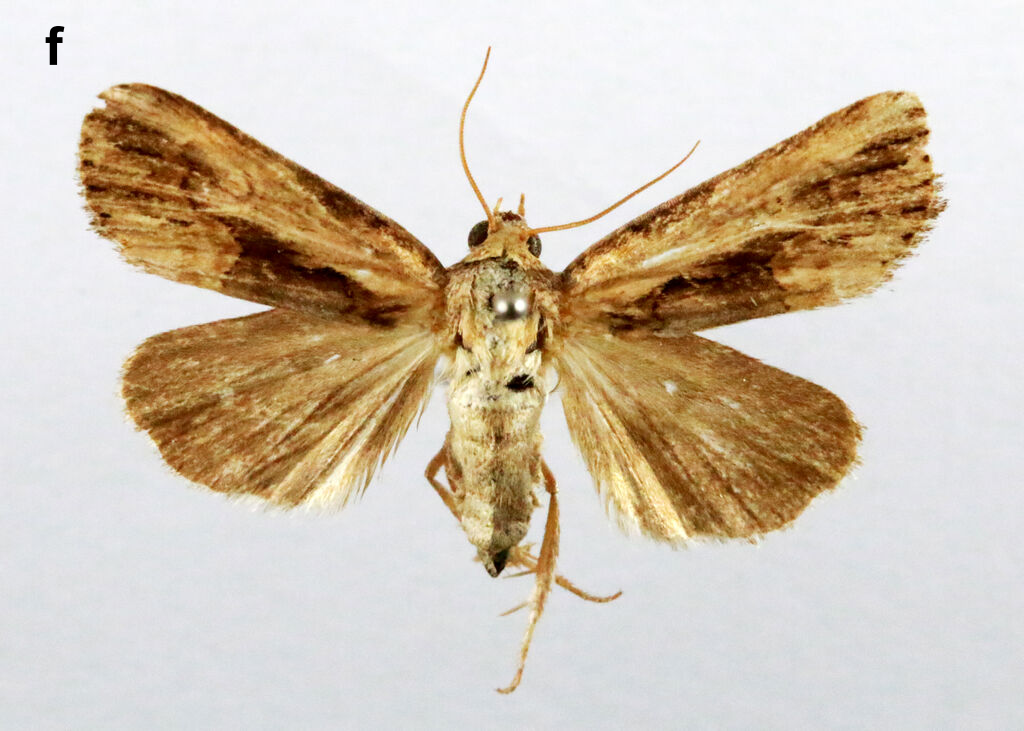
Matoposelecta

**Figure 21a.**
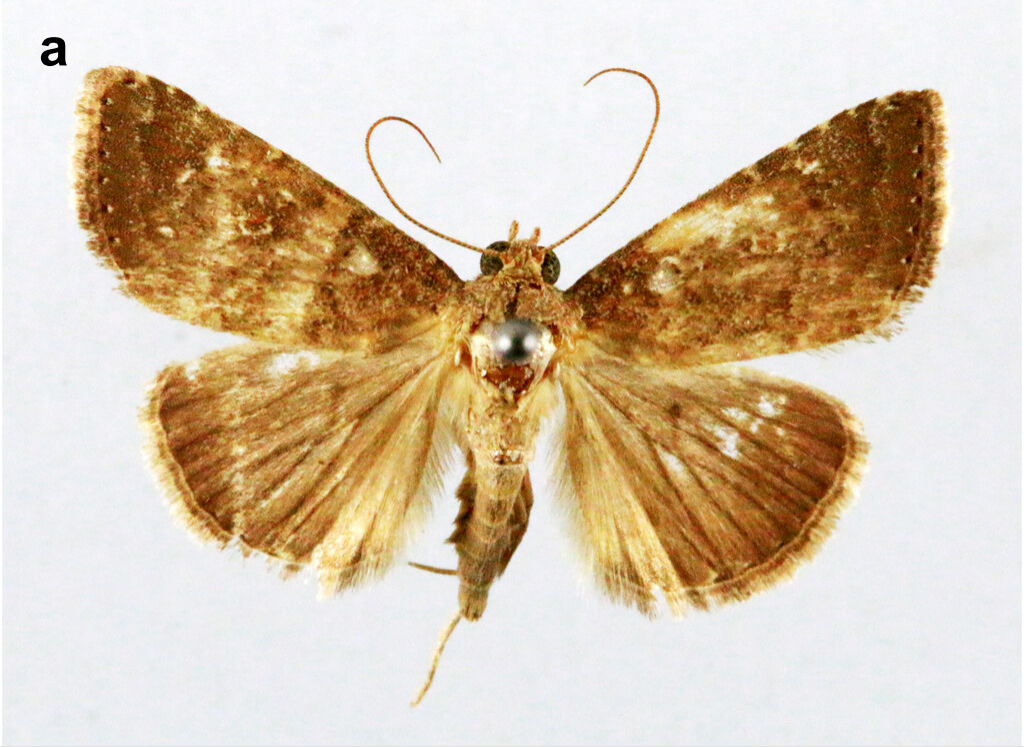
Amynaaxis

**Figure 21b.**
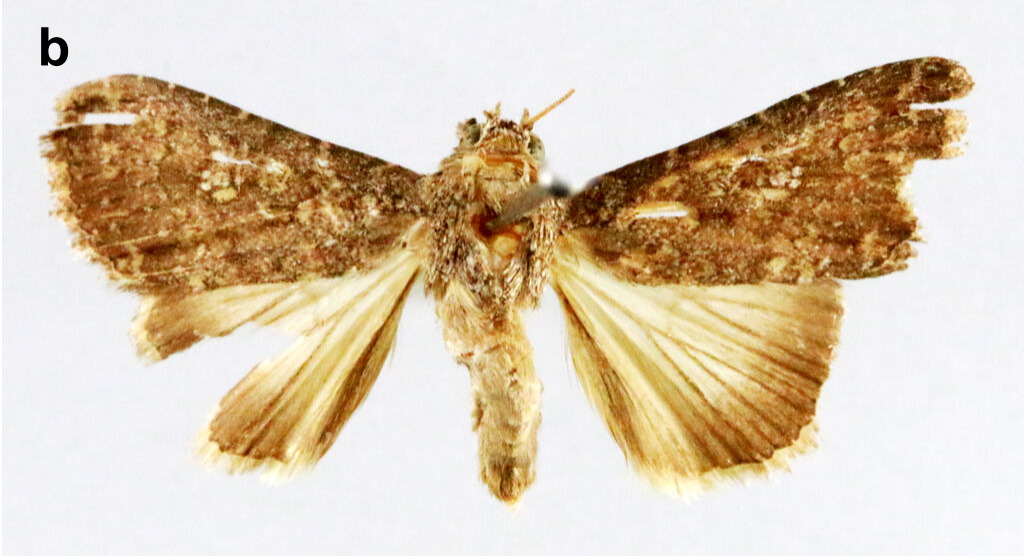
Perigeagalaxia

**Figure 21c.**
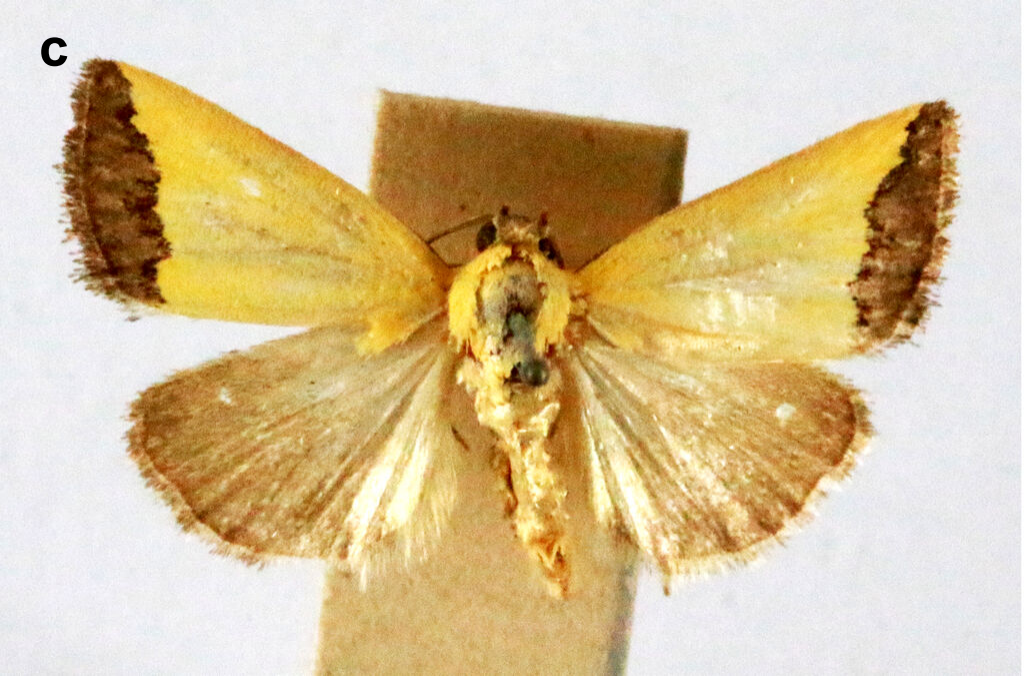
Deltotemarginate

**Figure 21d.**
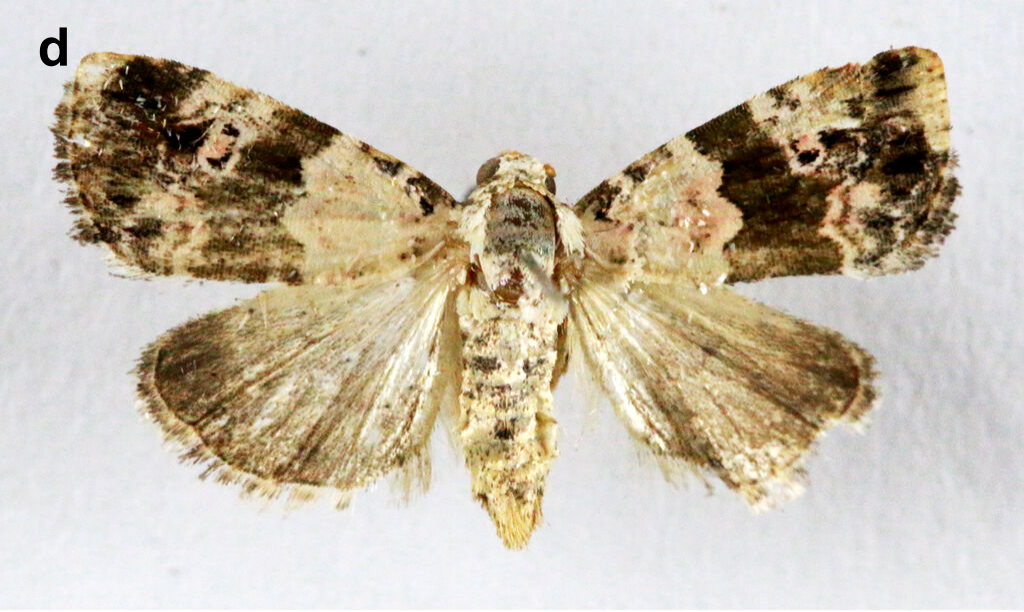
Maliatthasignifera

**Figure 21e.**
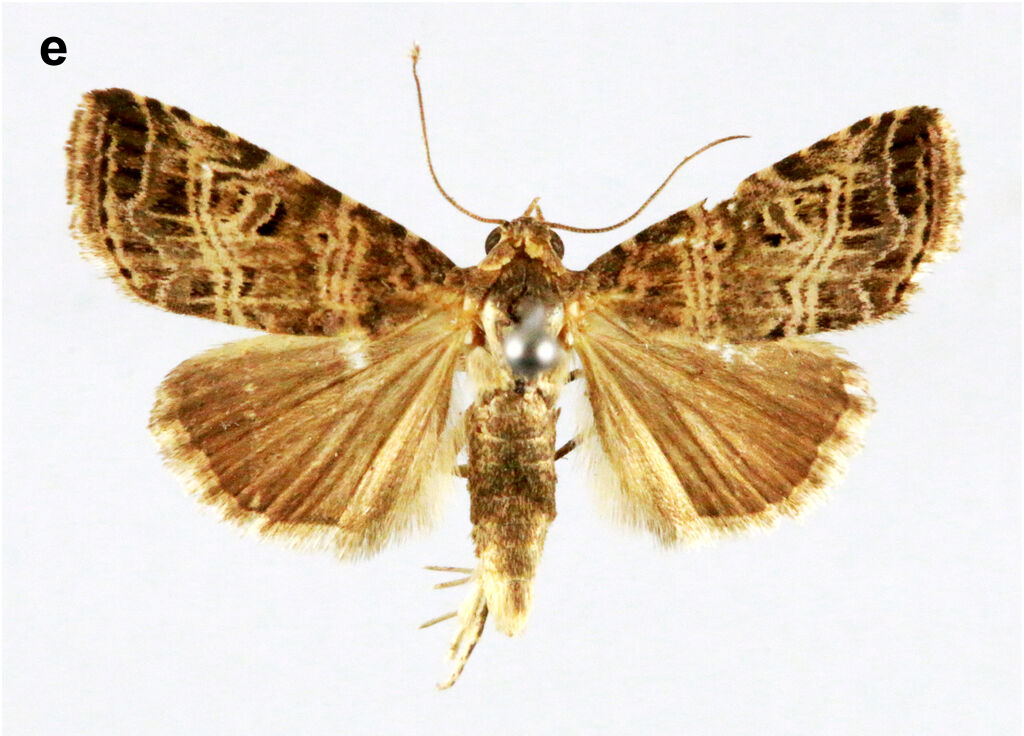
Ozarbamallarba

**Figure 21f.**
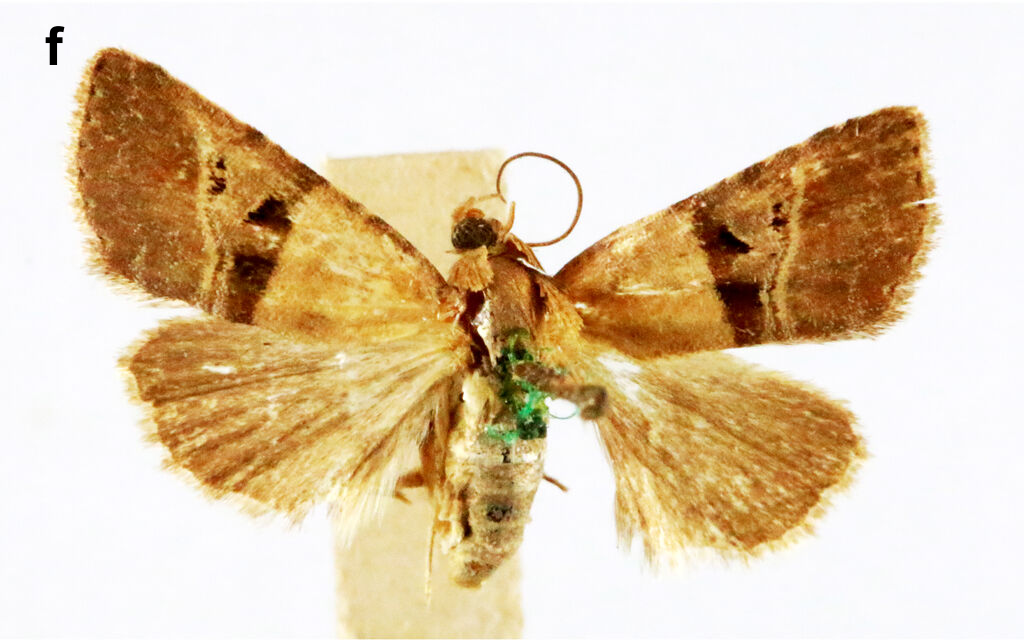
Ozarbapunctigera

**Figure 22a.**
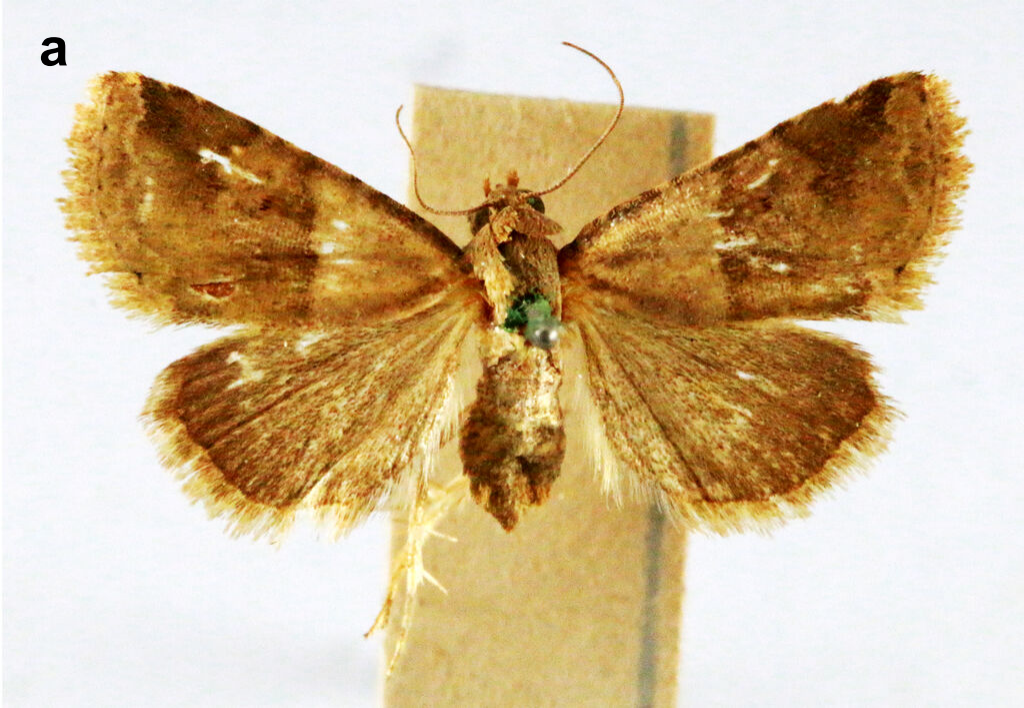
Ozarbarufula

**Figure 22b.**
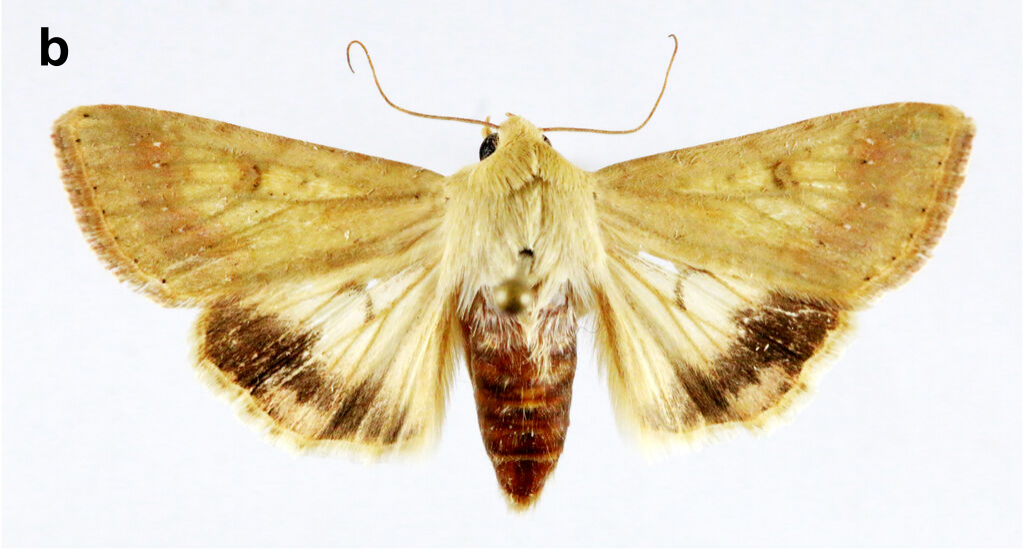
Helicoverpaarmigera

**Figure 22c.**
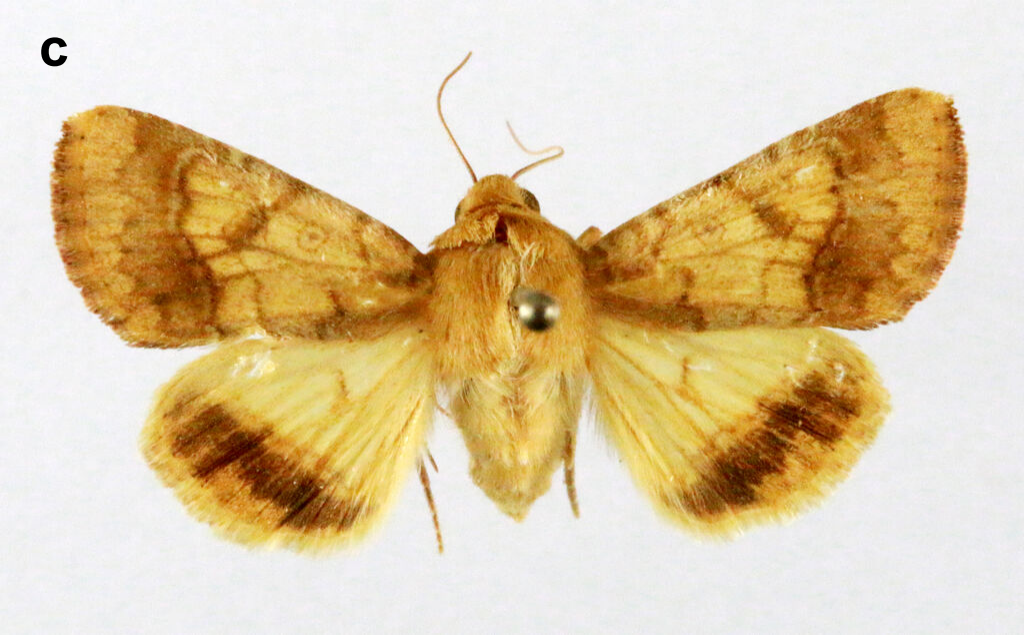
Helicoverpaassulta

**Figure 22d.**
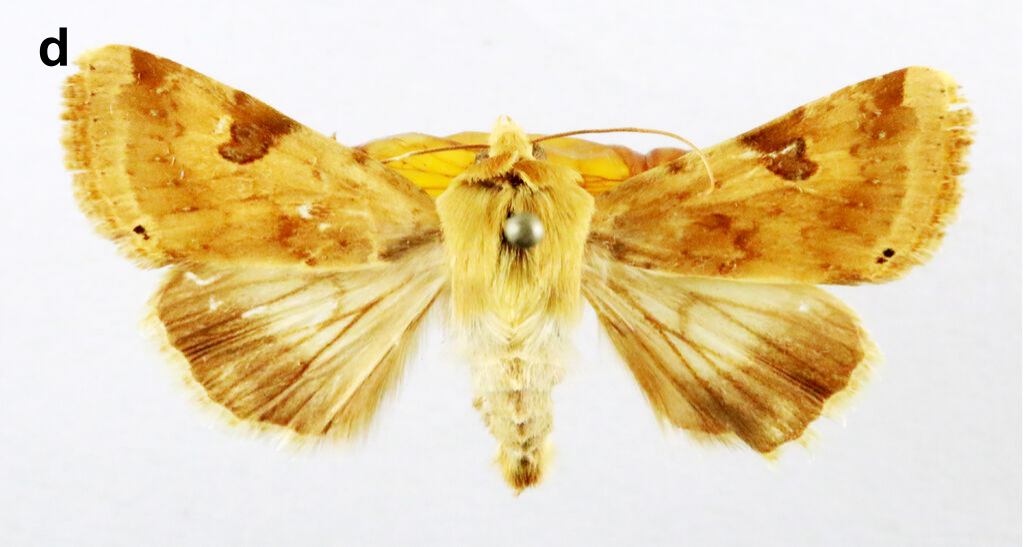
Heliothispeltigera

**Figure 22e.**
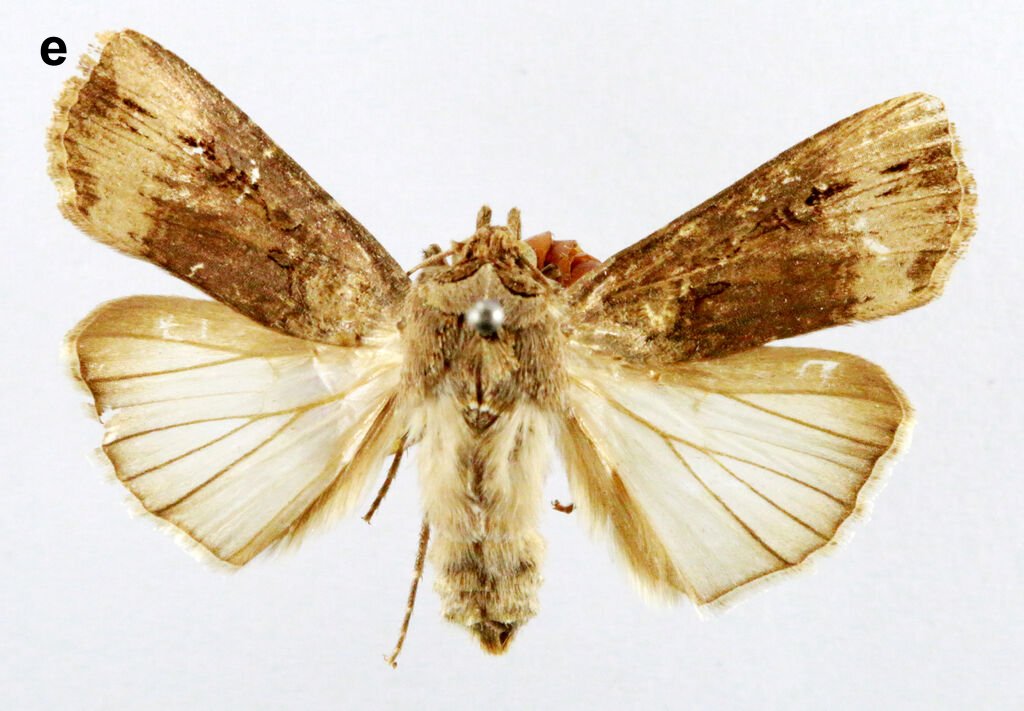
Agrotisipsilon

**Figure 22f.**
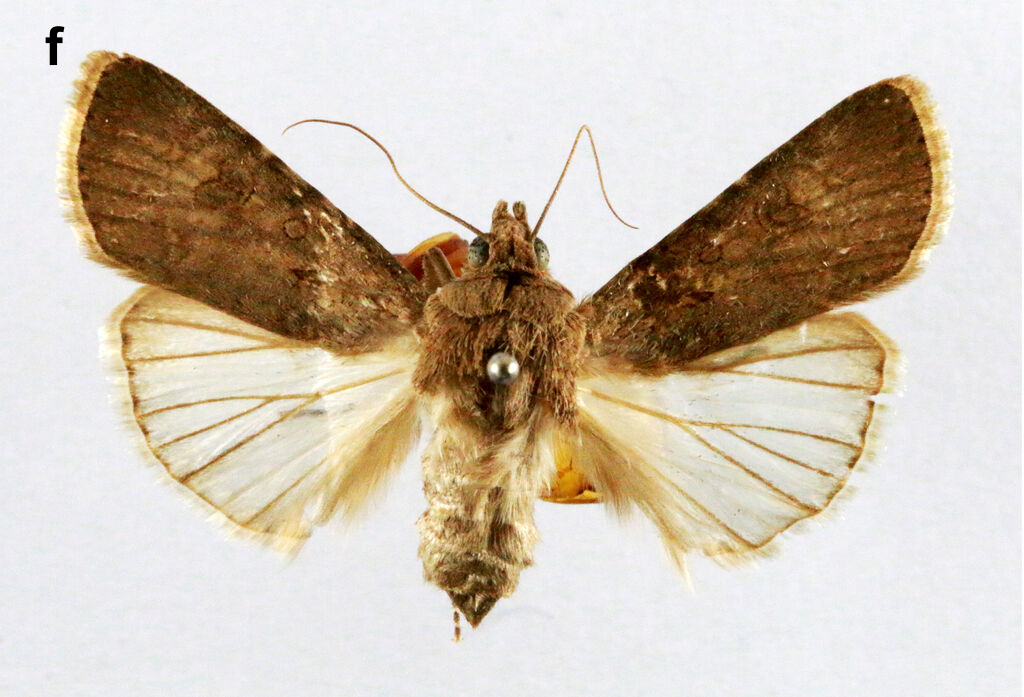
Agrotissegetum

**Figure 23a.**
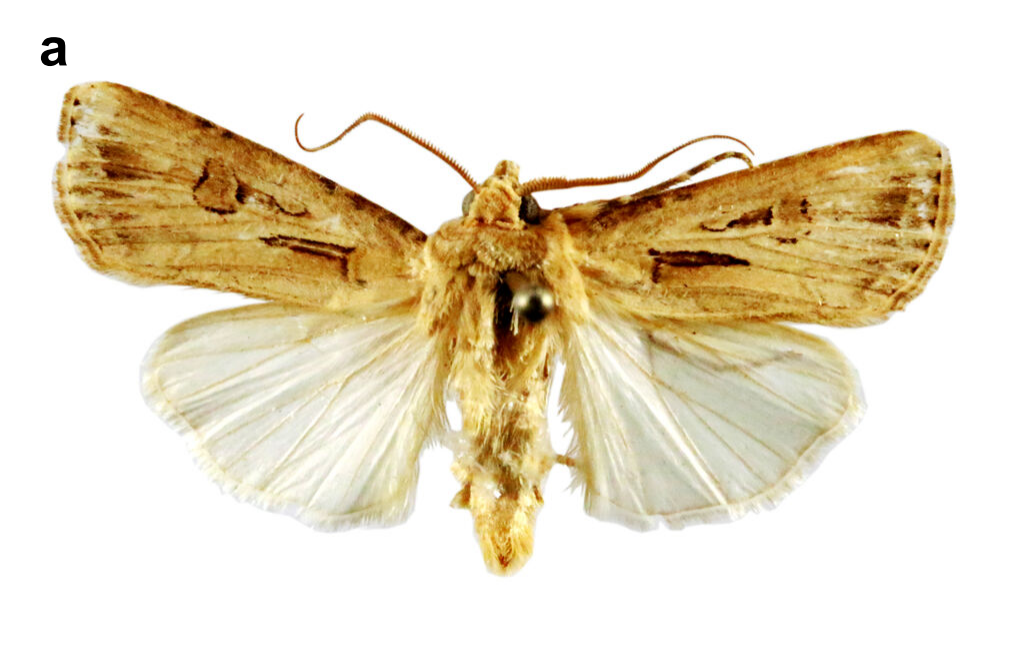
Agrotisspinifera

**Figure 23b.**
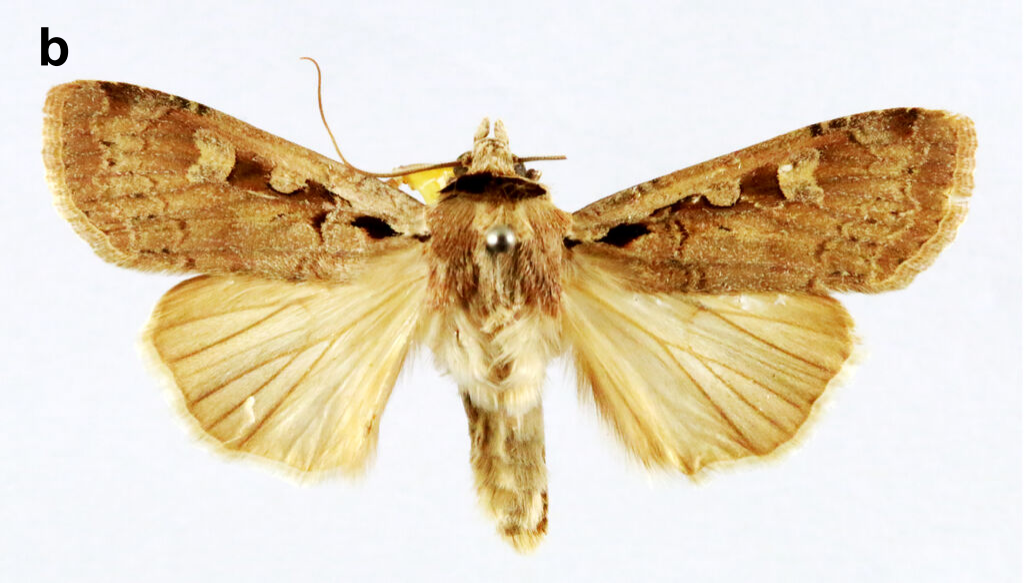
Dichagyrisflammatra

**Figure 23c.**
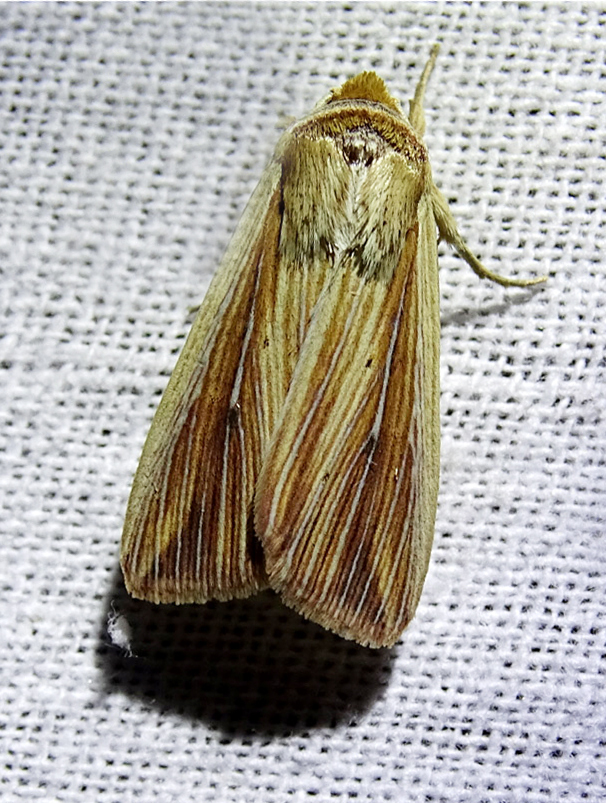
Leucaniacomma

**Figure 23d.**
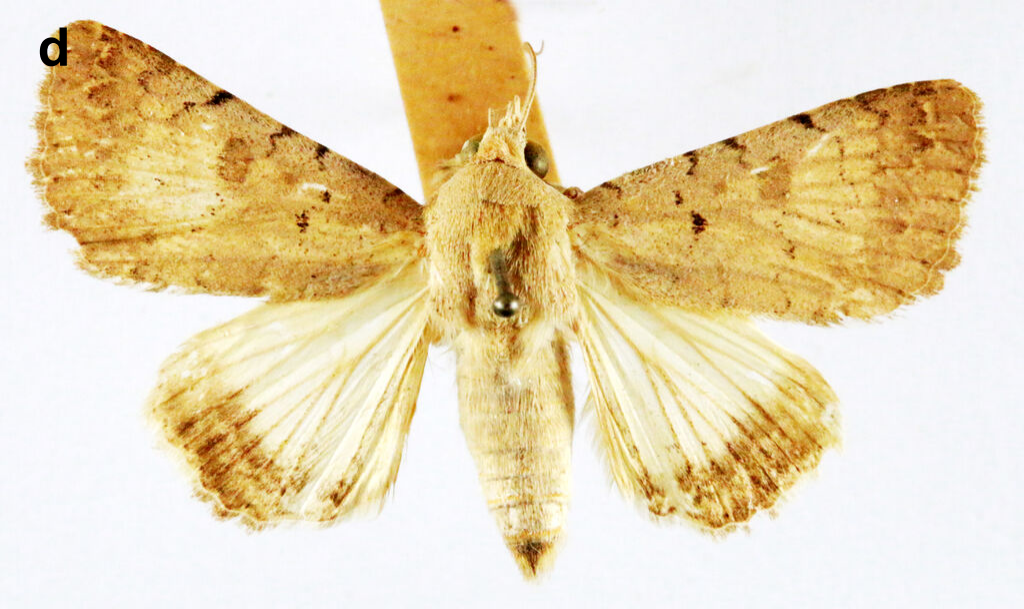
Mudariacornifrons

**Figure 23e.**
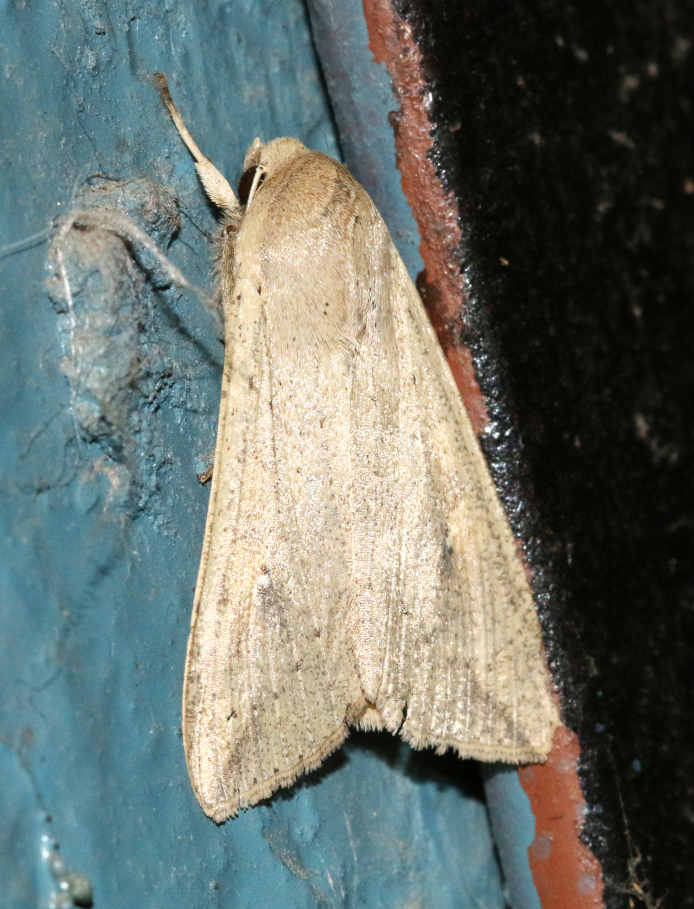
Mythimnaseparata

**Figure 23f.**
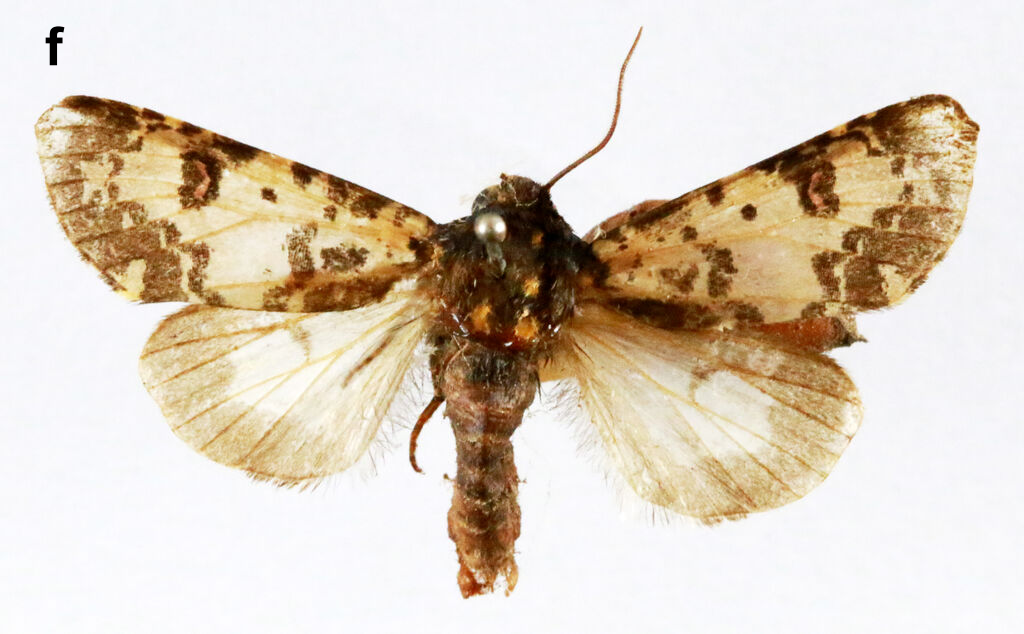
Polytelacliens

**Figure 24a.**
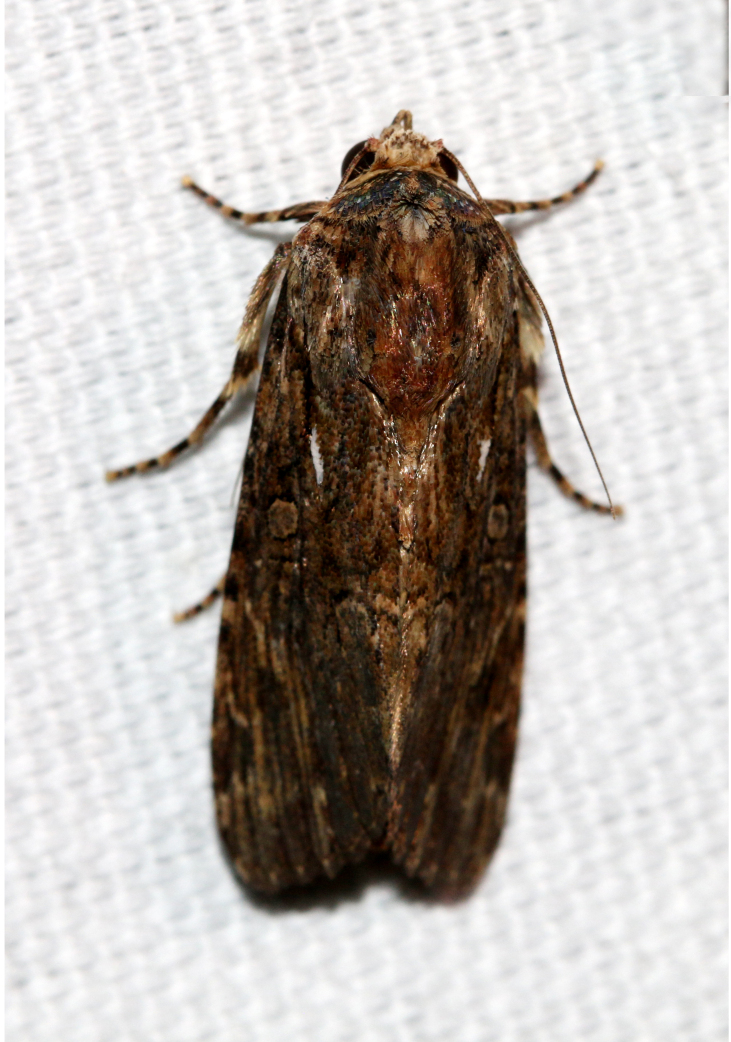
Sasunagatenebrosa

**Figure 24b.**
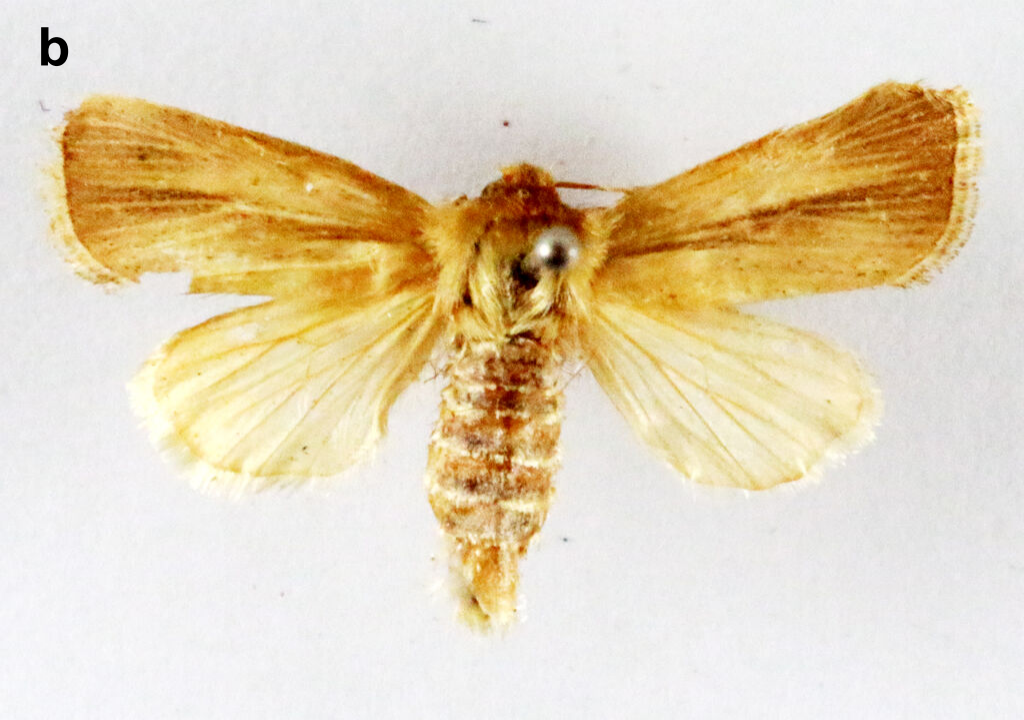
Sesamiauniformis

**Figure 24c.**
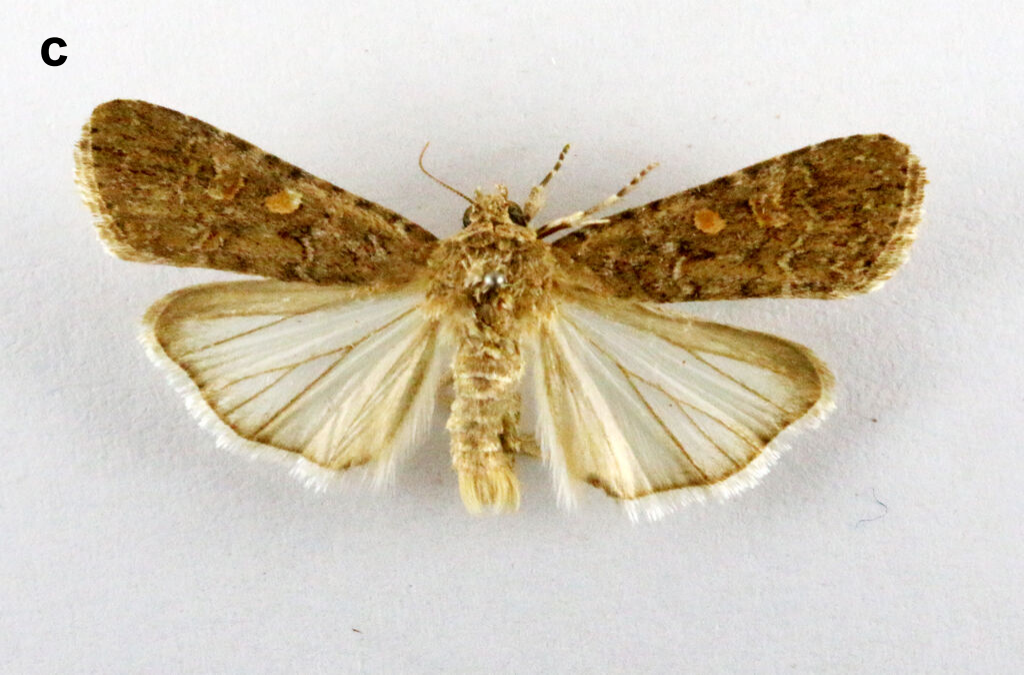
Spodopteraexigua

**Figure 24d.**
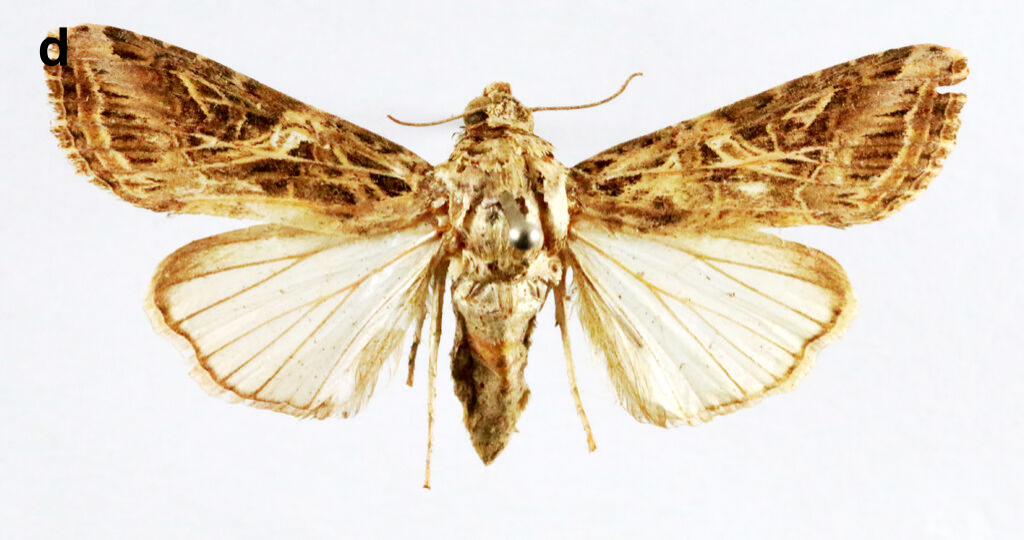
Spodopteralitura

**Figure 24e.**
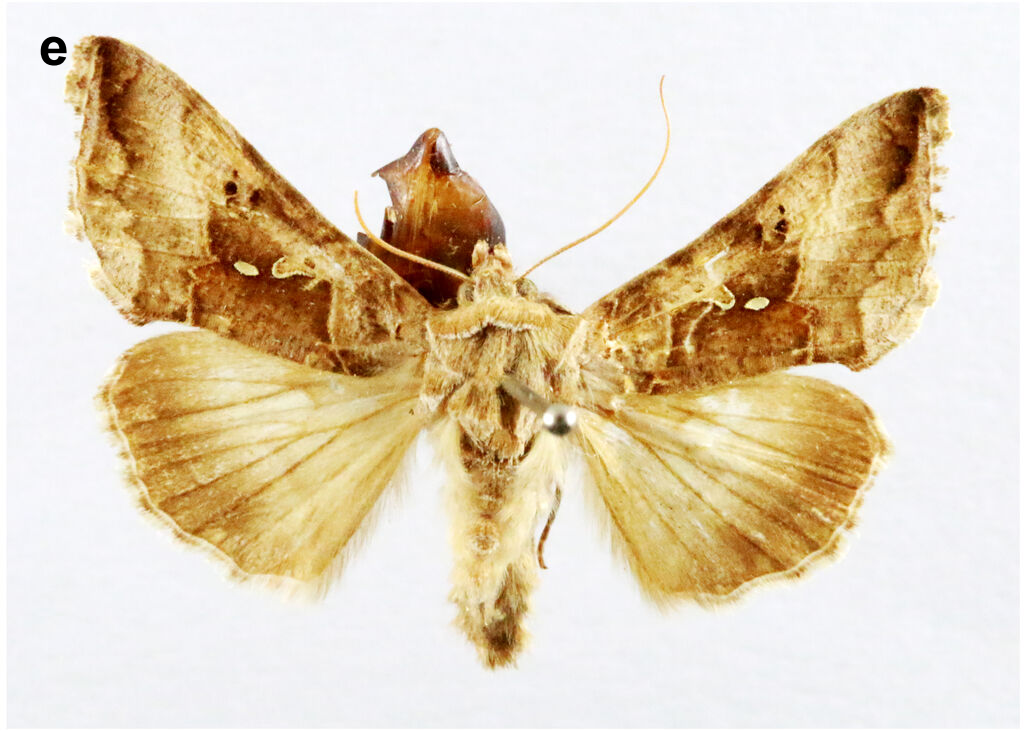
Autographanigrisigna

**Figure 24f.**
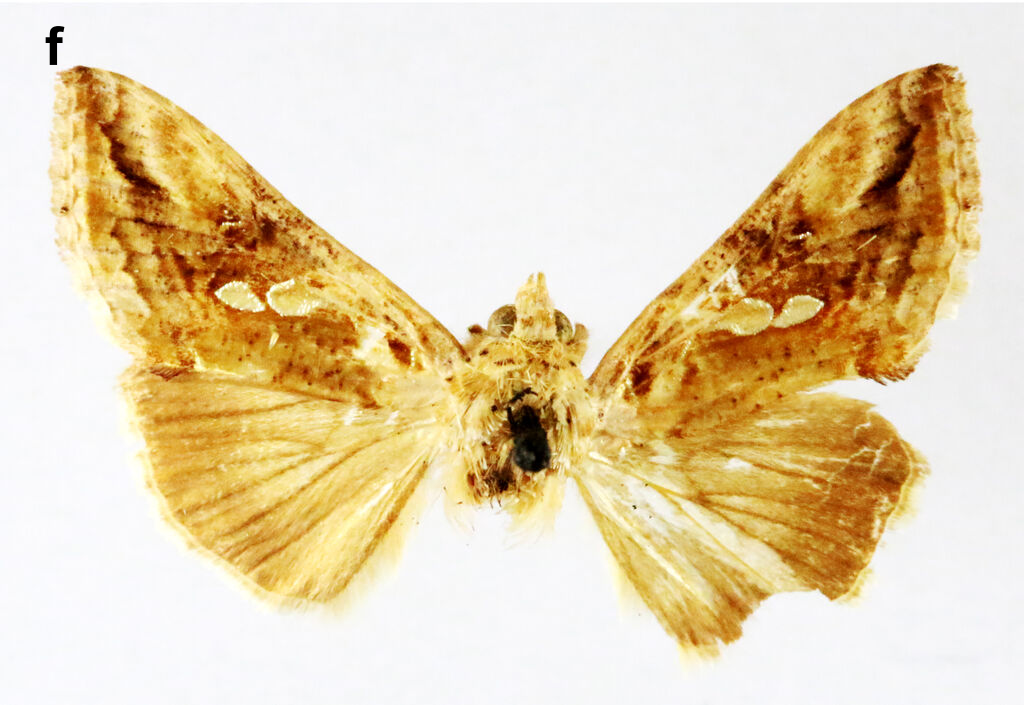
Chrysodeixischalcites

**Figure 25a.**
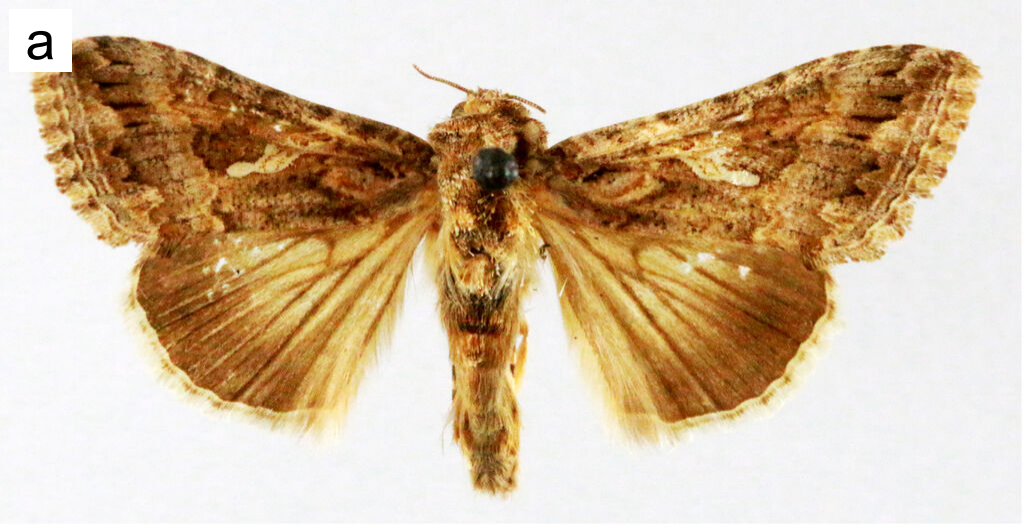
Chrysodeixiseriosoma

**Figure 25b.**
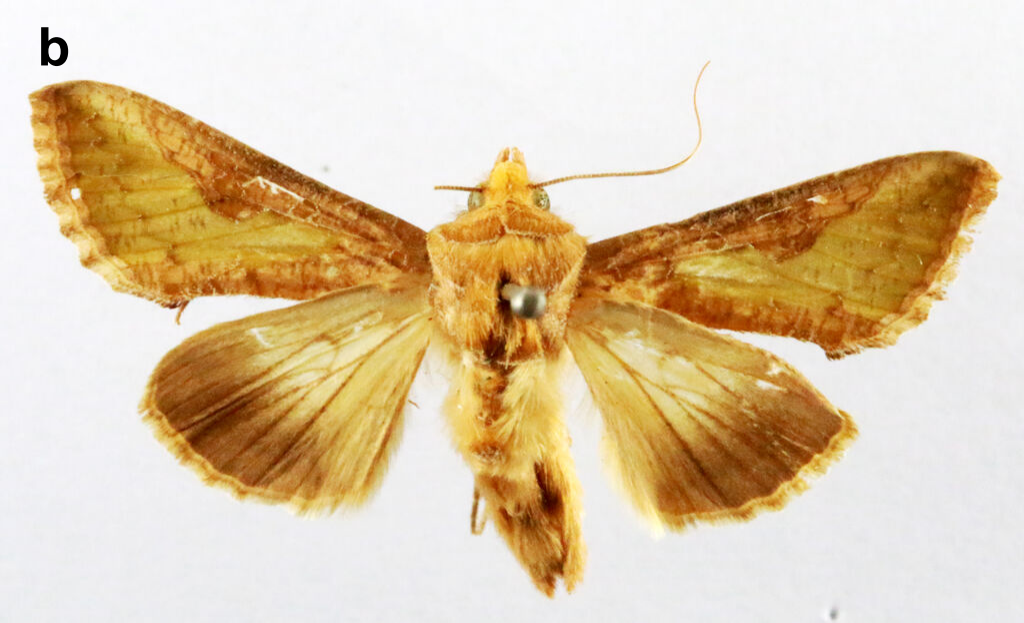
Thysanoplusiaorichalcea

**Figure 25c.**
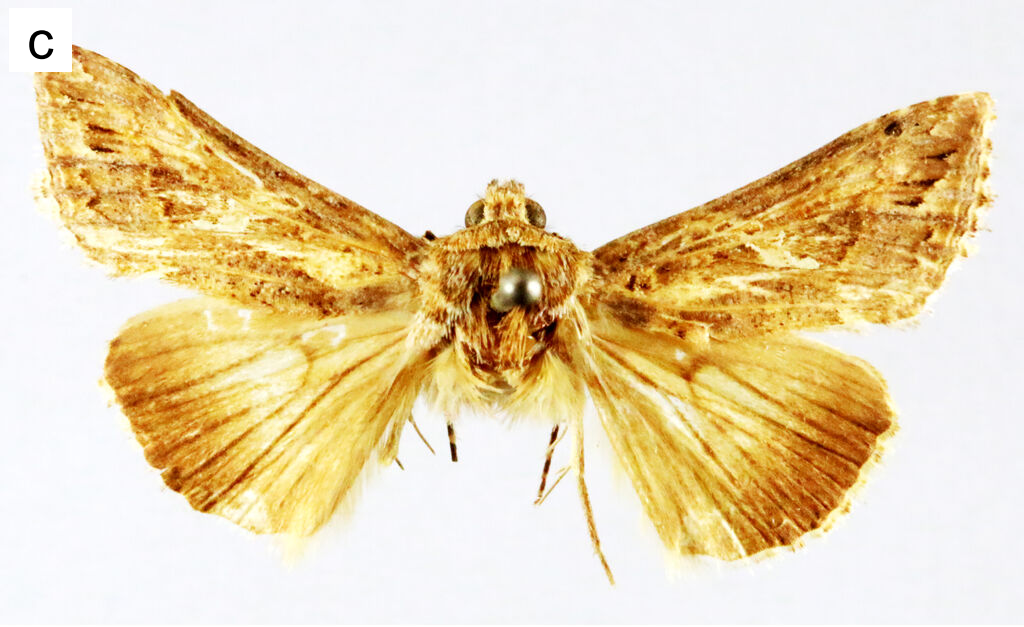
Trichoplusiani

**Figure 25d.**
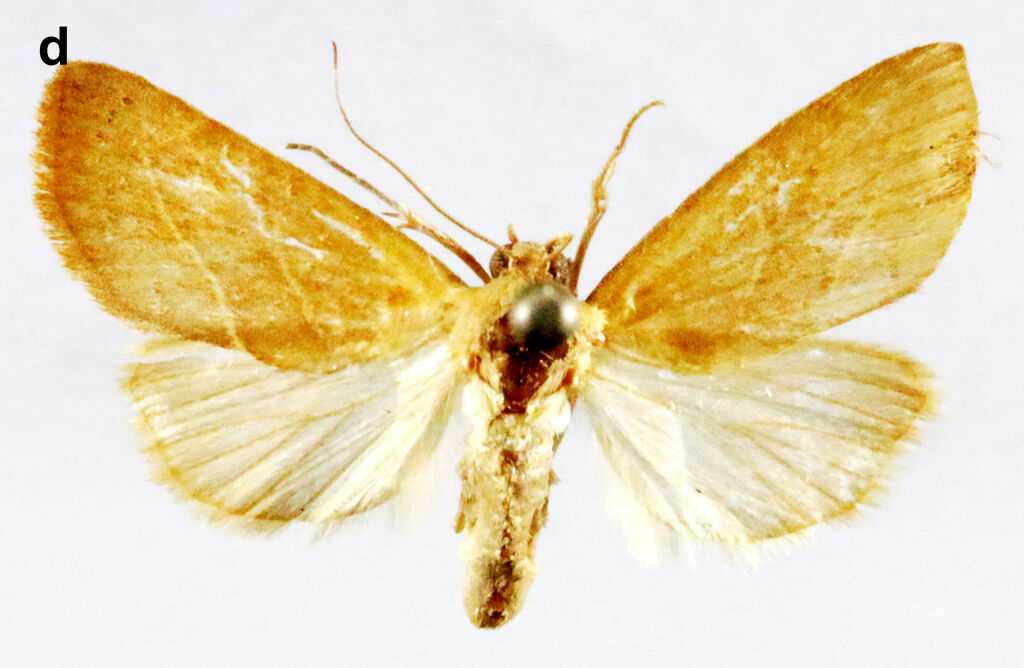
Arcyophoradentula

**Figure 25e.**
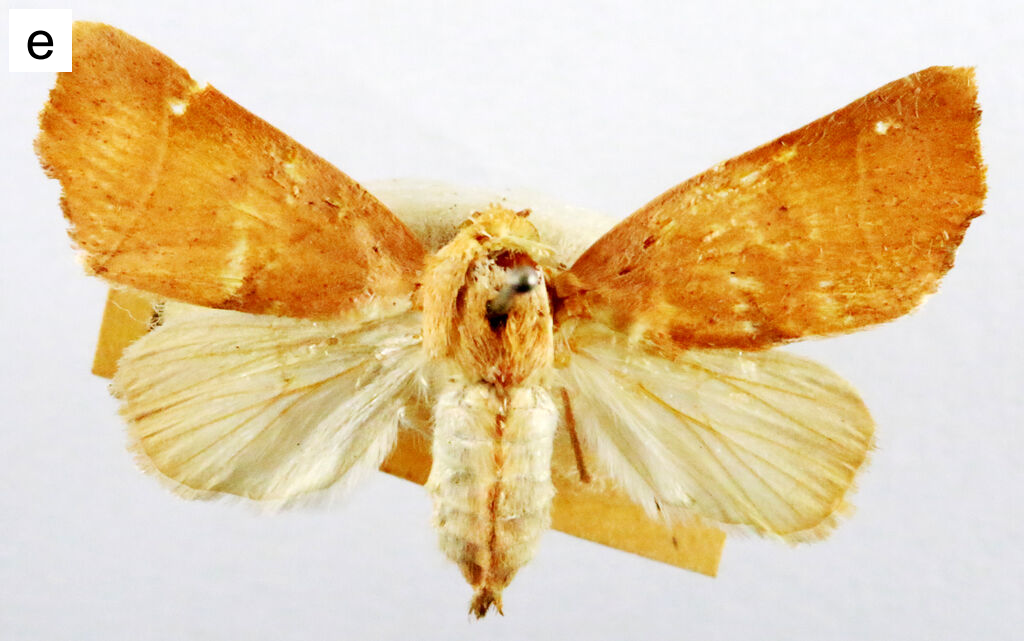
Careaangulata

**Figure 25f.**
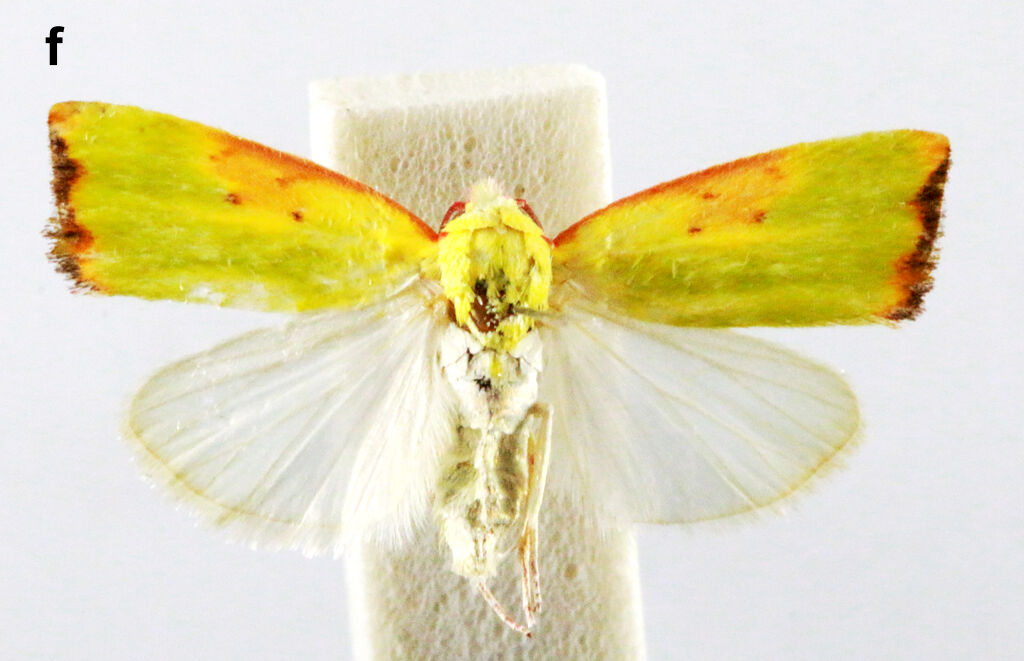
Eariascupreoviridis

**Figure 26a.**
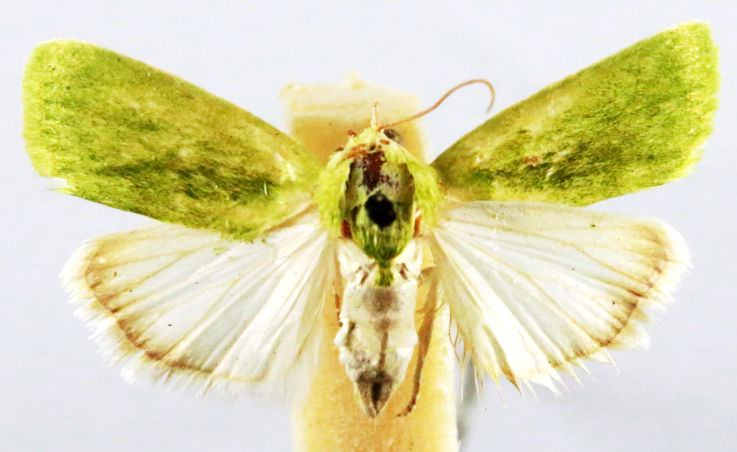
Eariasinsulana

**Figure 26b.**
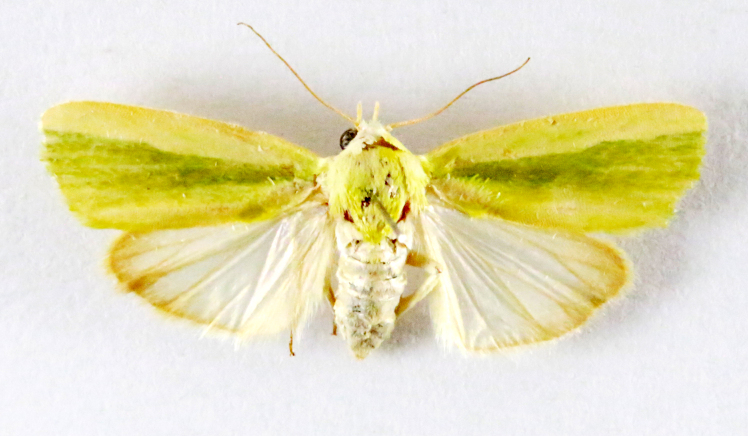
Eariasvittella

**Figure 26c.**
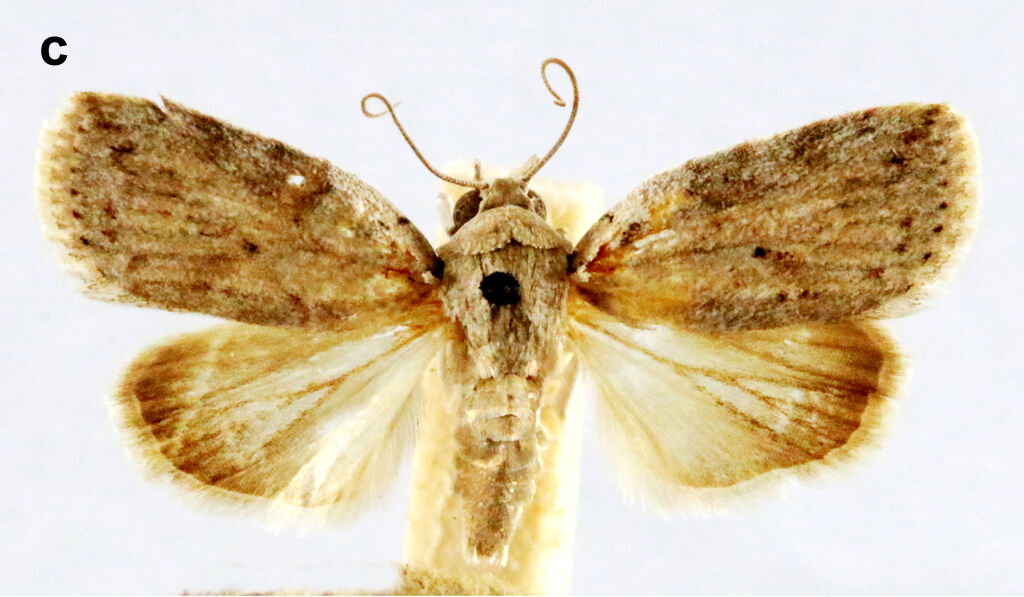
Giaurasceptica

**Figure 26d.**
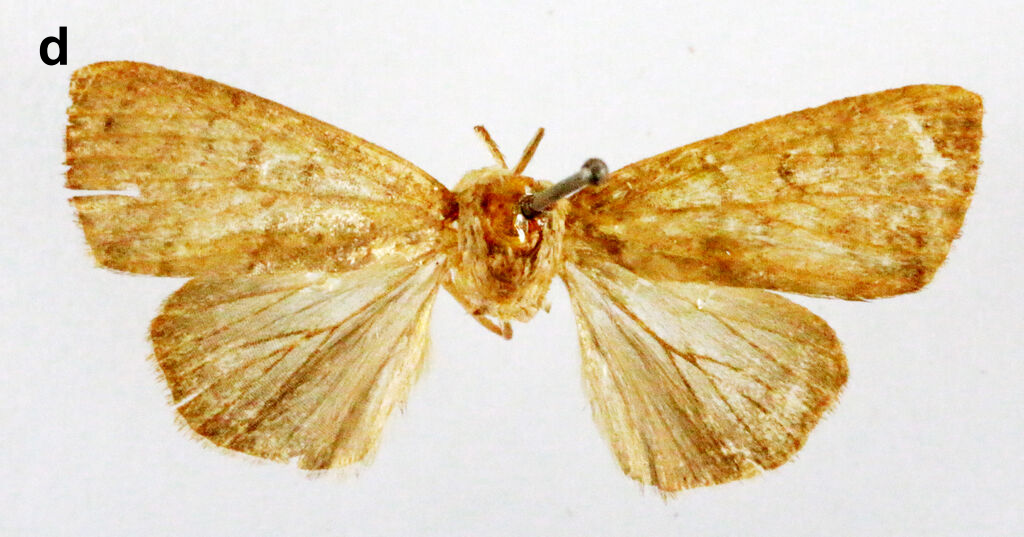
Mauriliaiconica

**Figure 26e.**
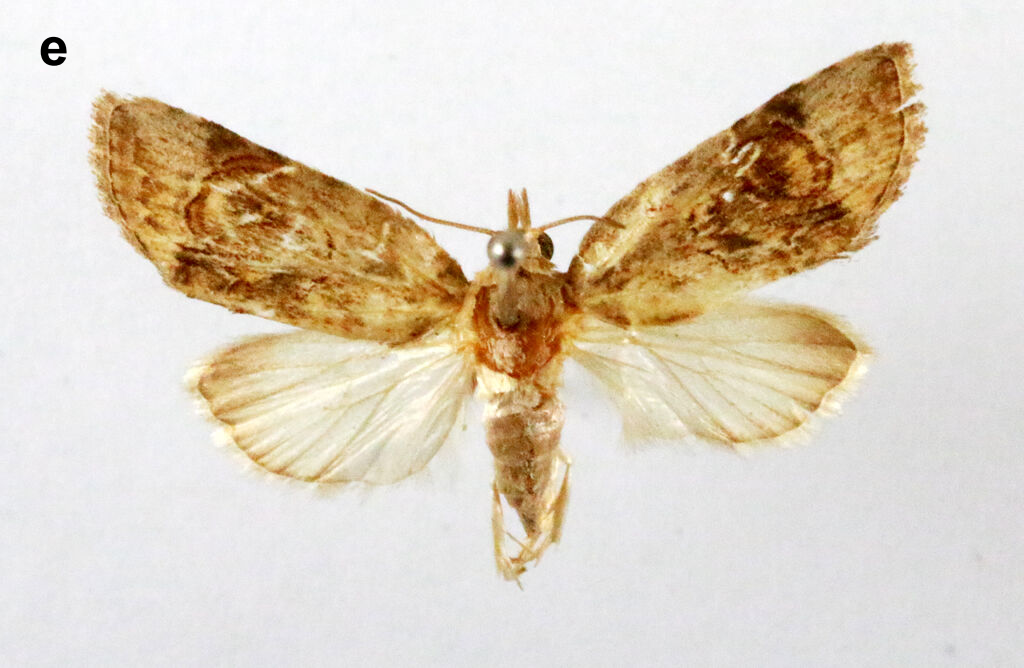
Selepaceltis

**Figure 26f.**
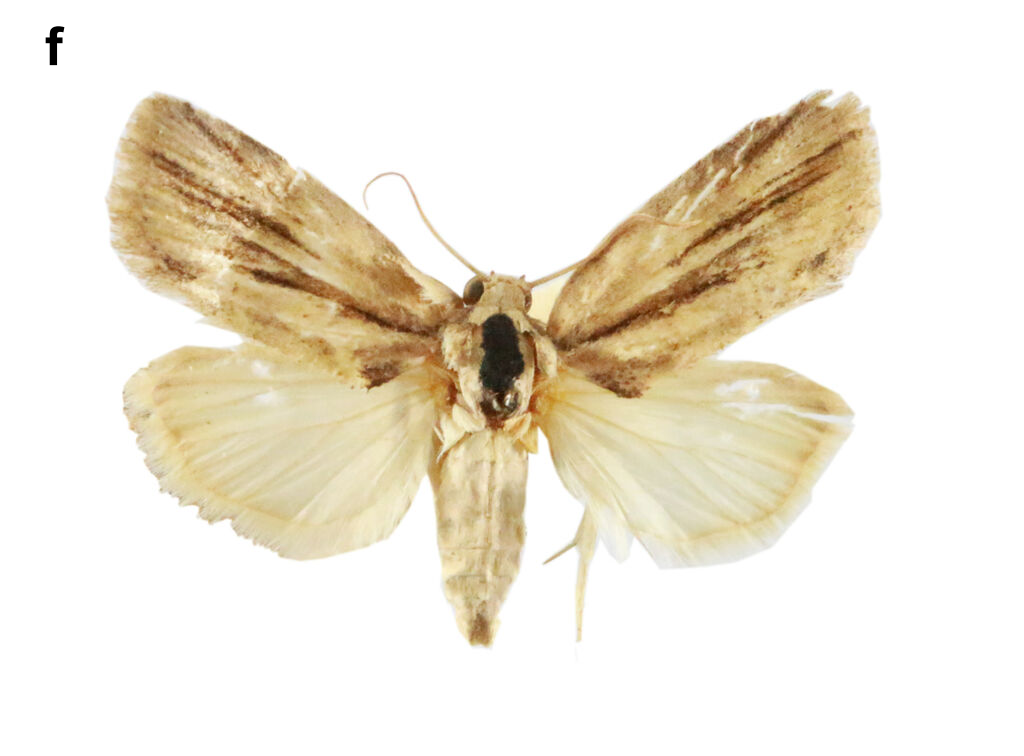
Selepadocilis

**Figure 27a.**
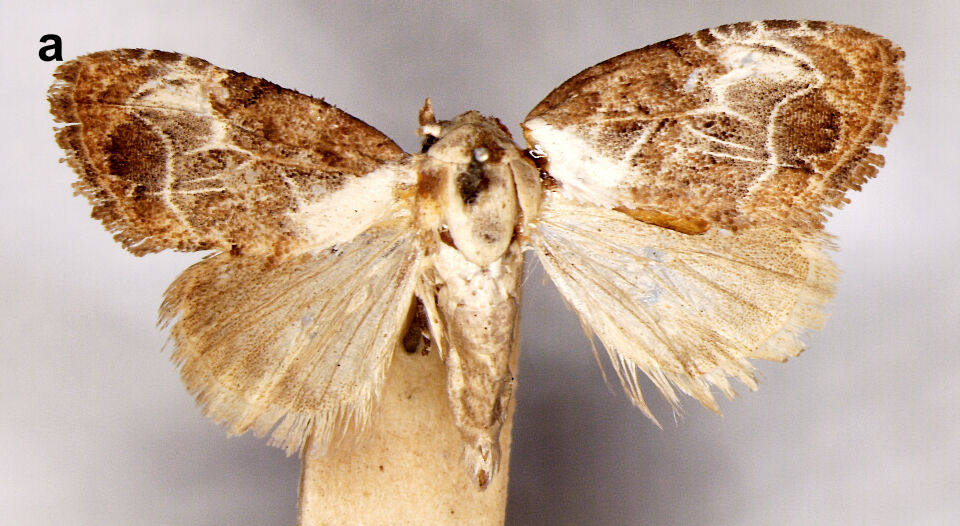
Evonimaplagiola

**Figure 27b.**
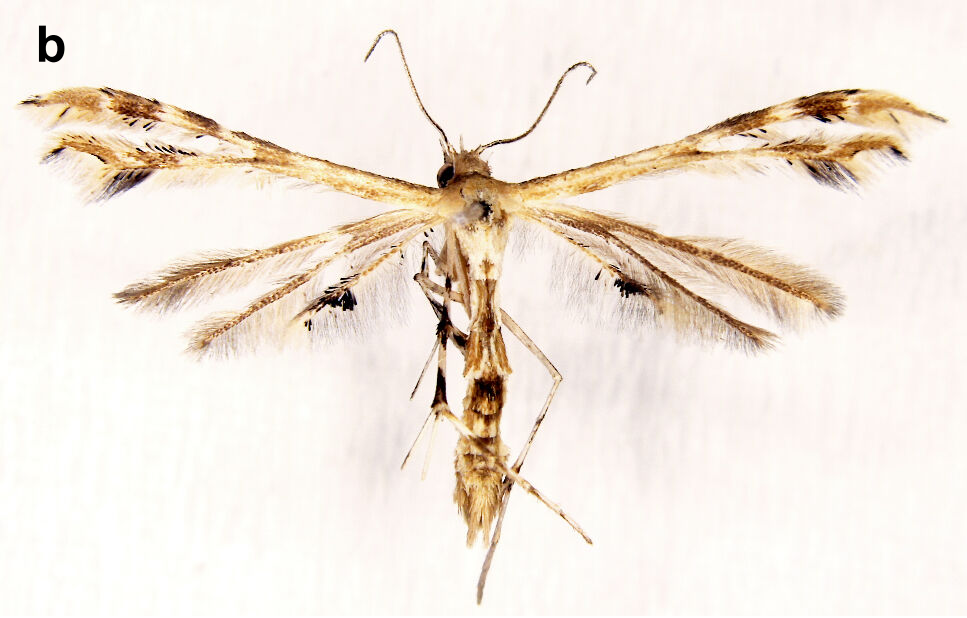
Sphenarchescaffer

**Figure 27c.**
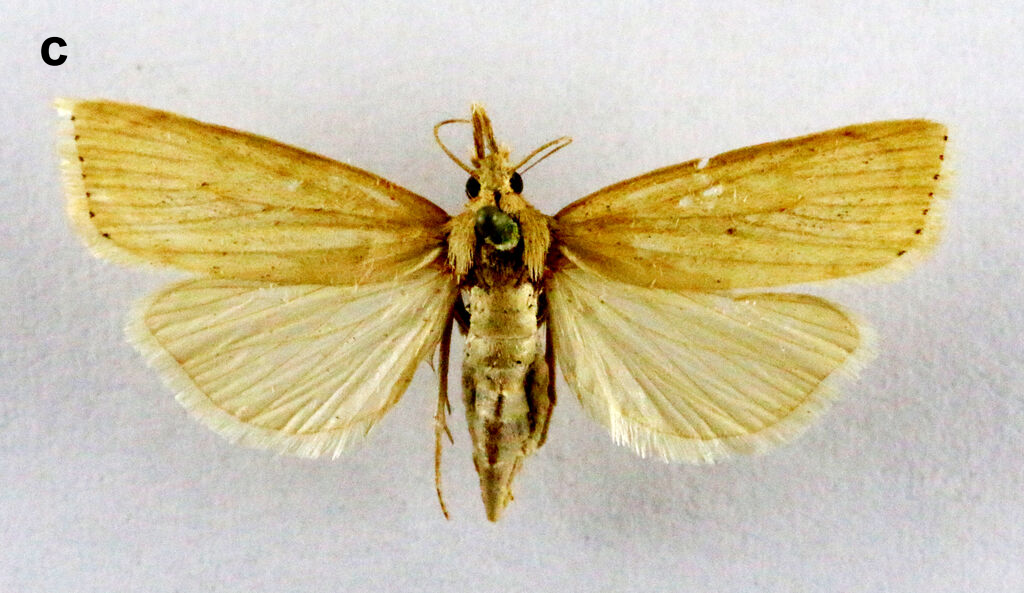
Chilopartellus

**Figure 27d.**
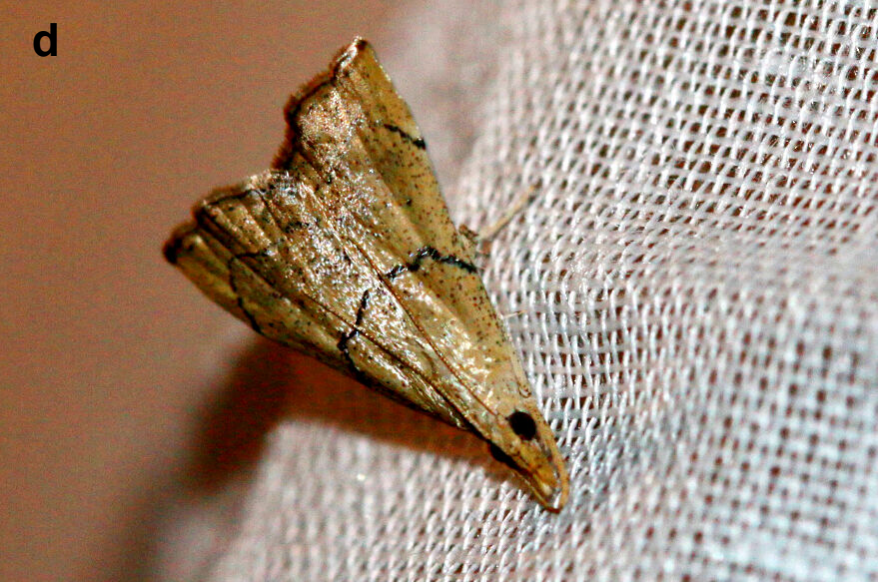
*Ptychopseustis* sp.

**Figure 27e.**
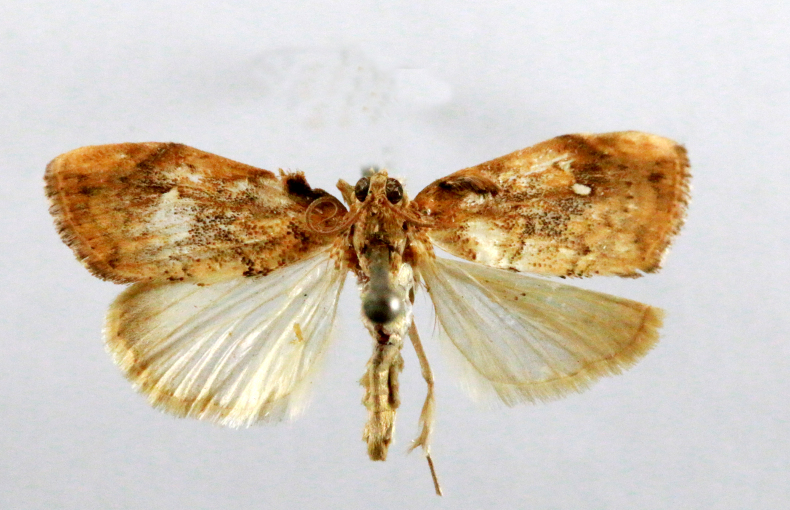
Crocidolomiapavonana

**Figure 27f.**
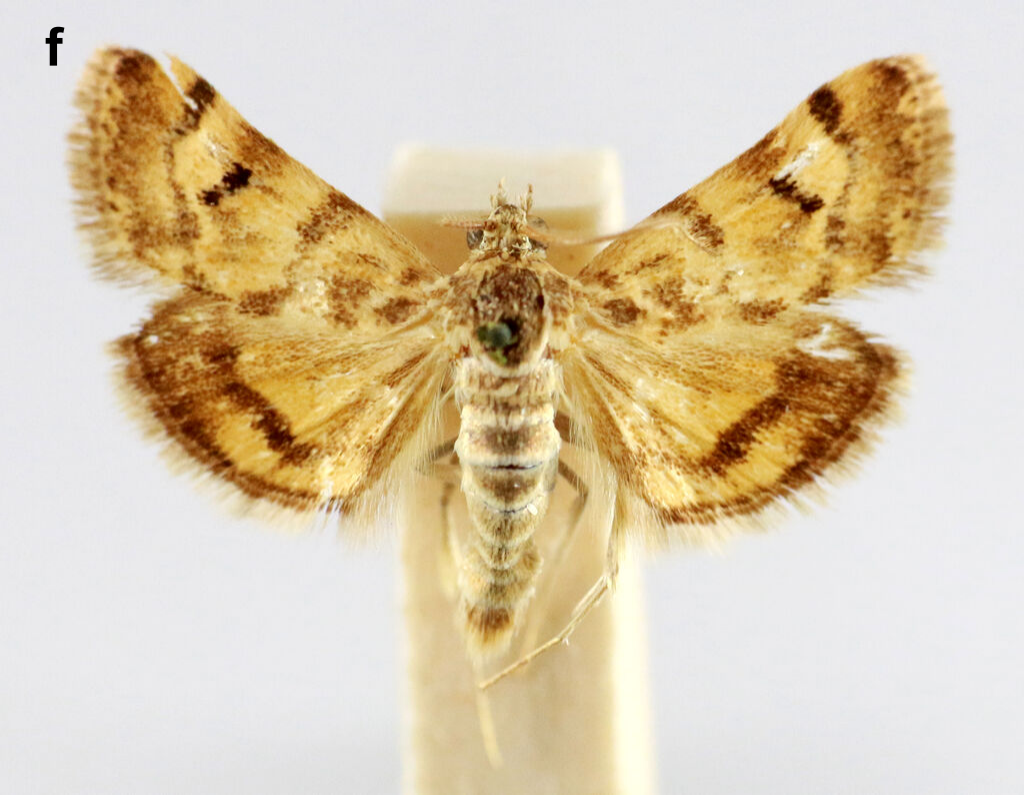
Aporodesfloralis

**Figure 28a.**
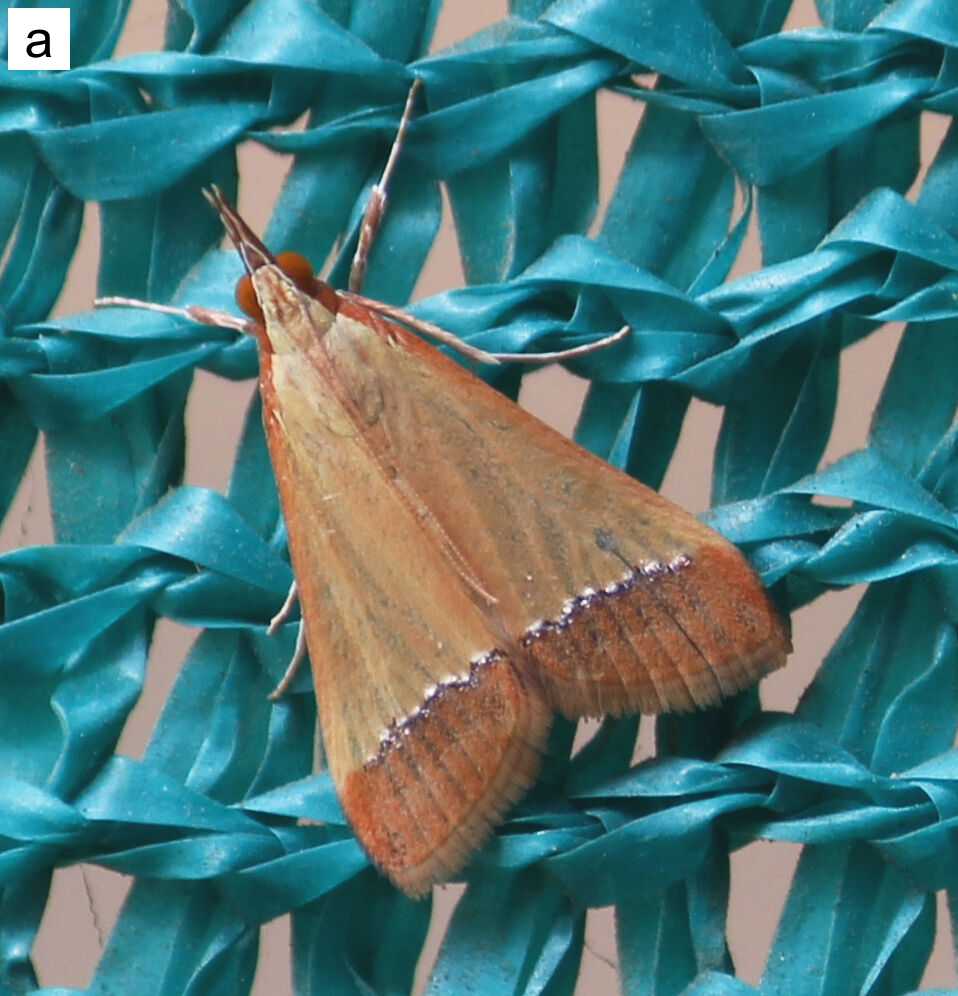
Autocharisfessalis

**Figure 28b.**
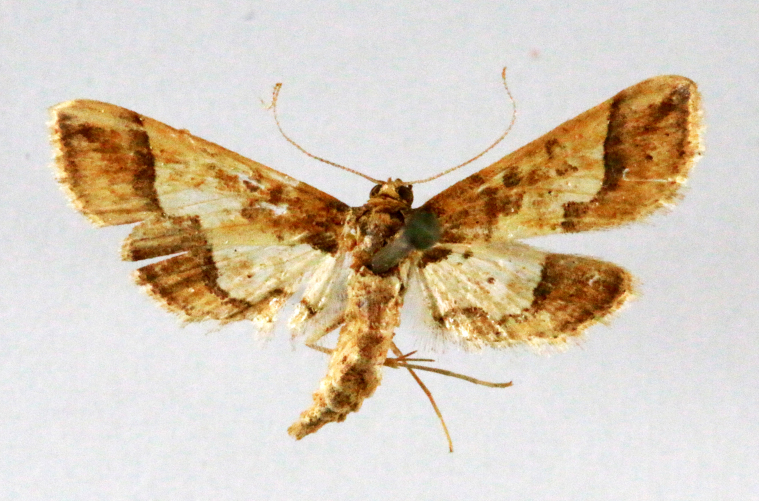
Hydririsornatalis

**Figure 28c.**
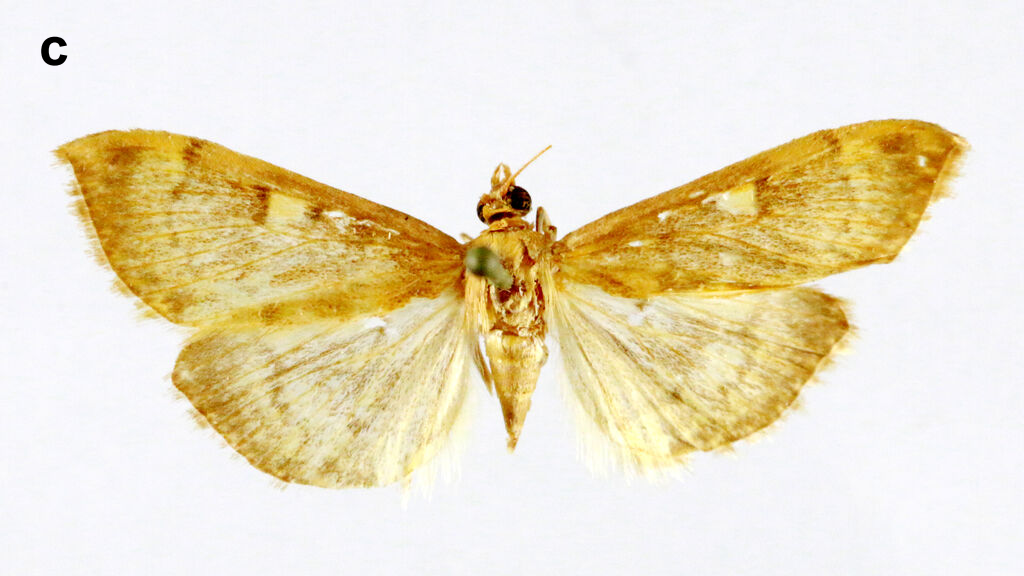
Pyraustaindistans

**Figure 28d.**
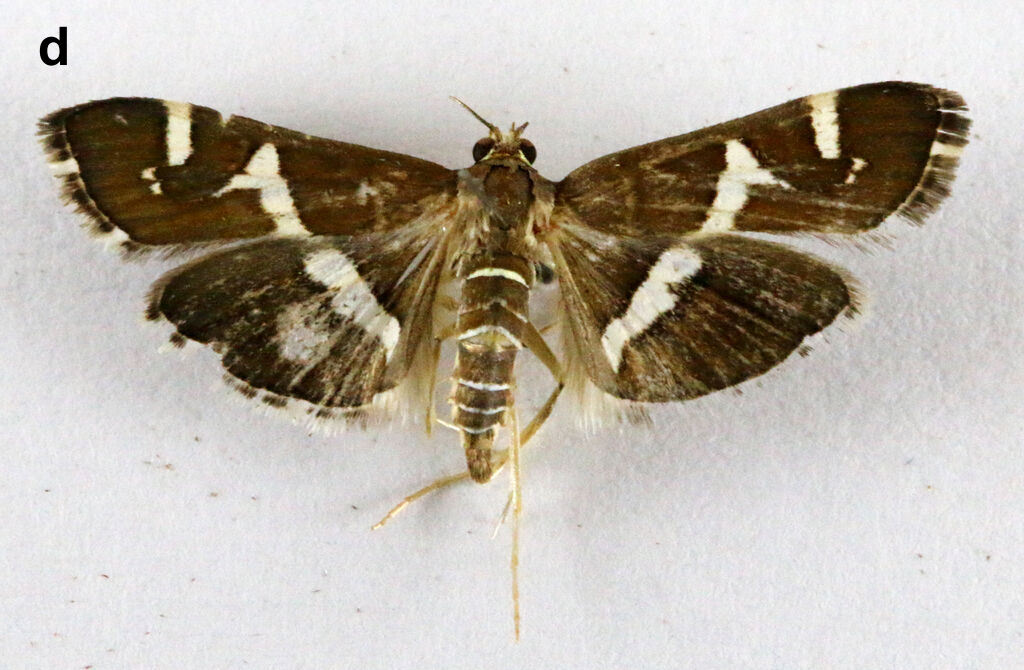
Spoladearecurvalis

**Figure 28e.**
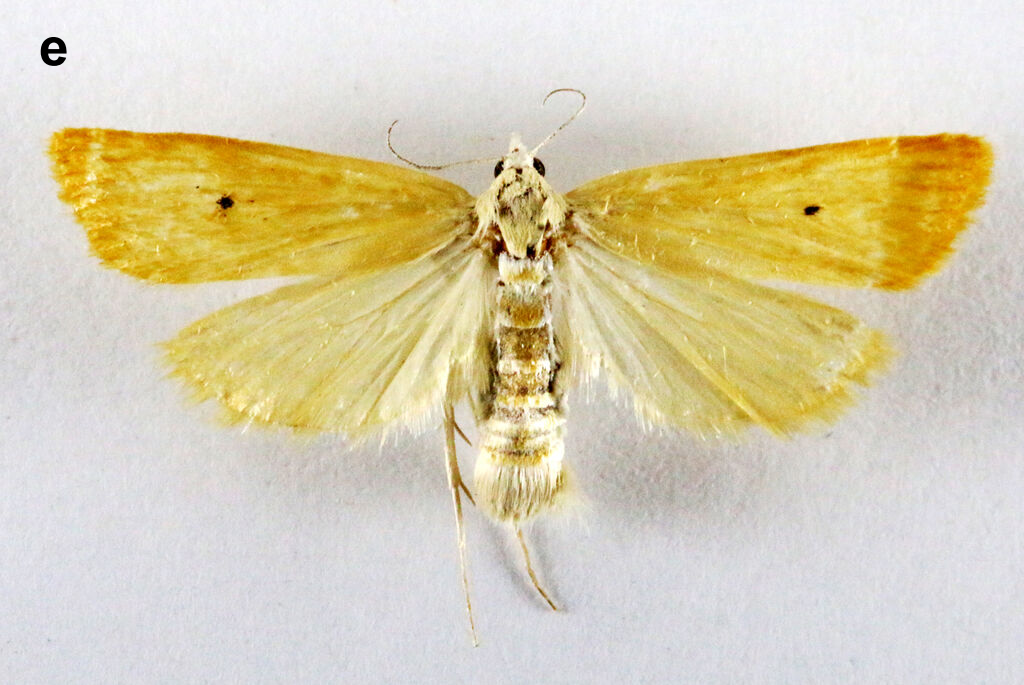
Scirpophagaincertulas

**Figure 28f.**
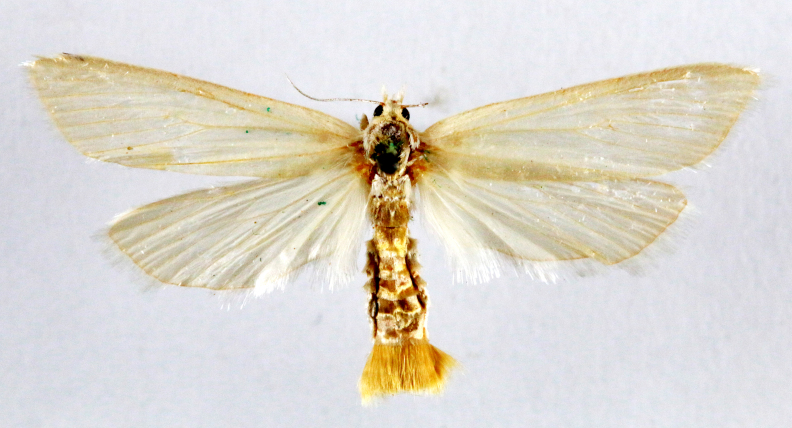
Scirpophaganivella

**Figure 29a.**
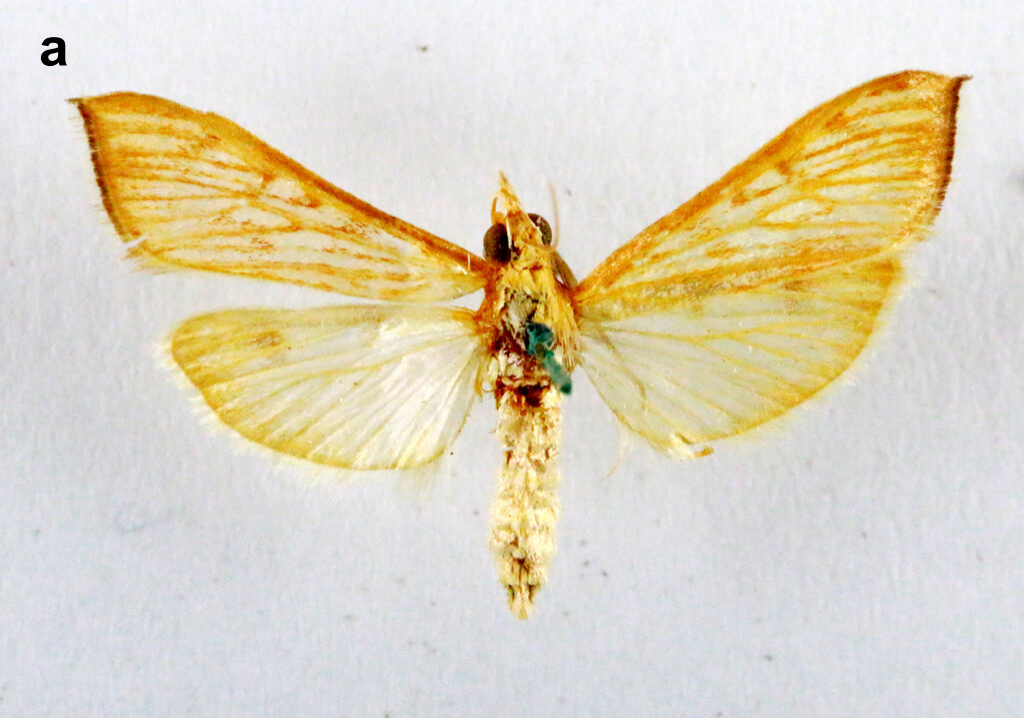
Antigastracatalaunalis

**Figure 29b.**
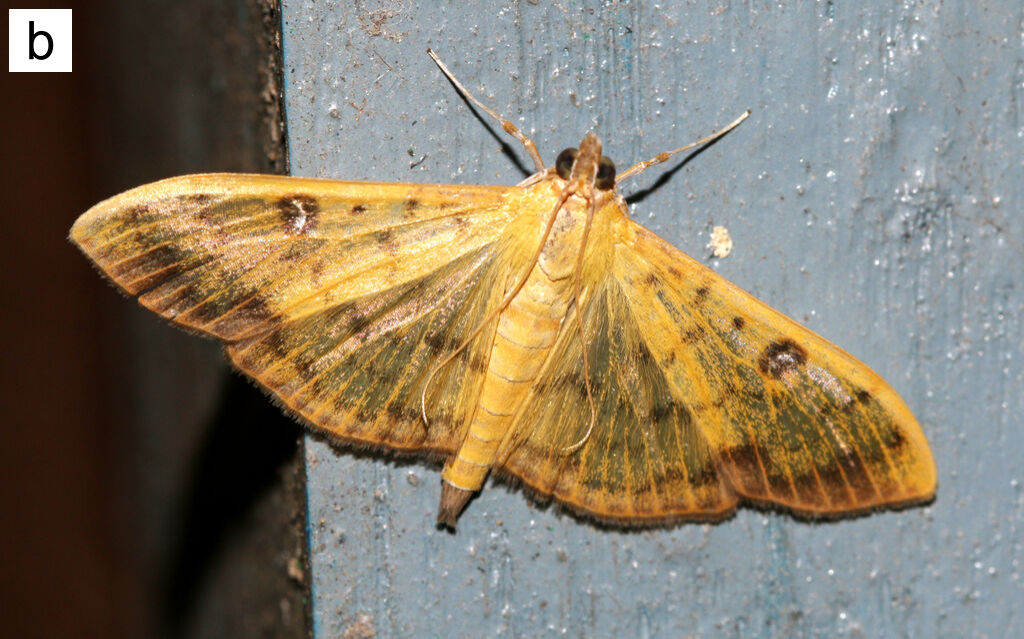
Botyodesdiniasalis

**Figure 29c.**
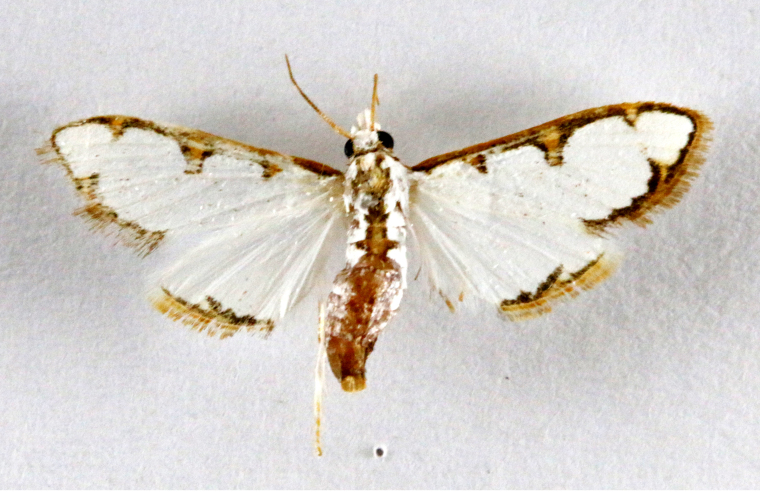
Cirrhochristabrizoalis

**Figure 29d.**
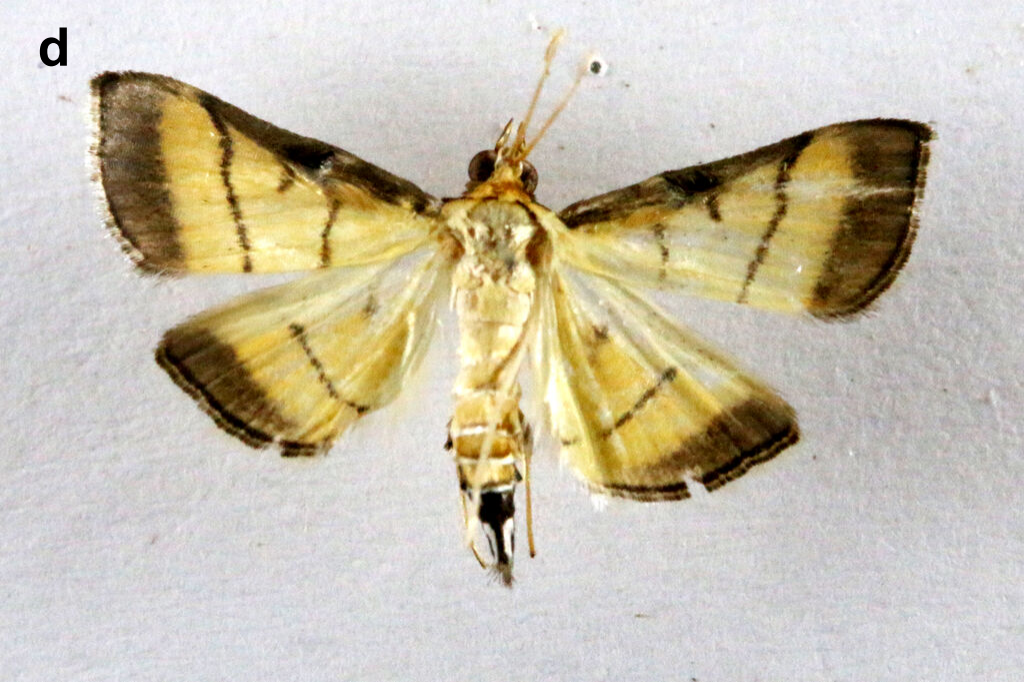
Cnaphalocrocismedinalis

**Figure 29e.**
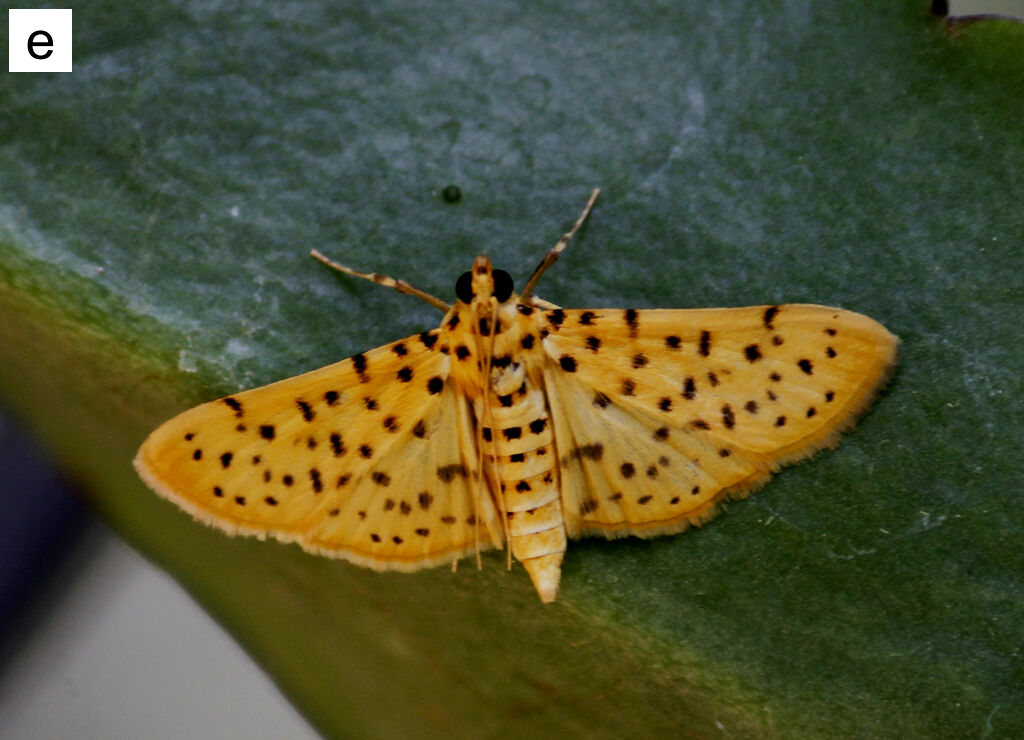
Conogethespunctiferalis

**Figure 29f.**
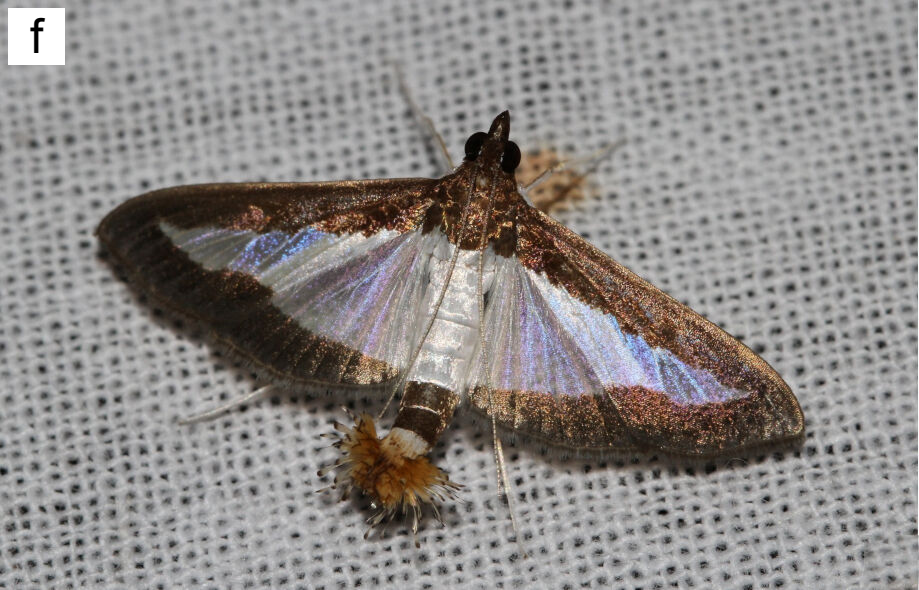
Diaphaniaindica

**Figure 30a.**
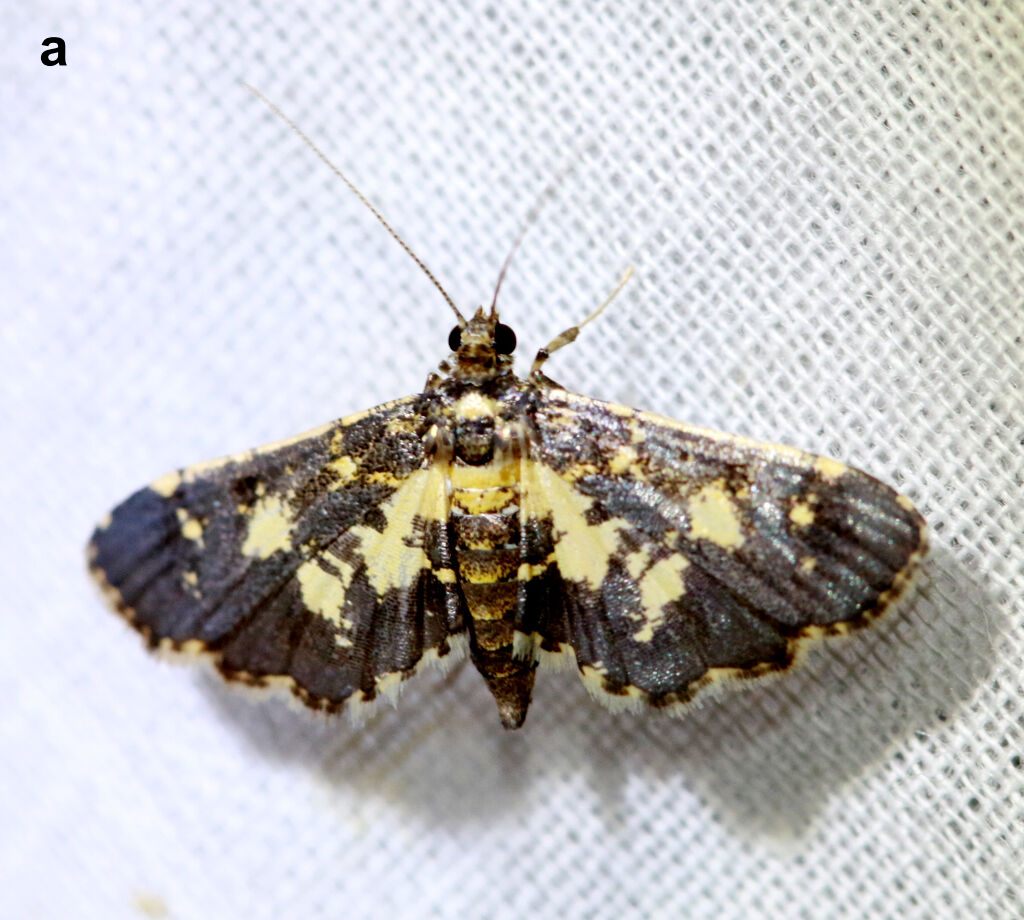
Eurrhyparodesbracteolalis

**Figure 30b.**
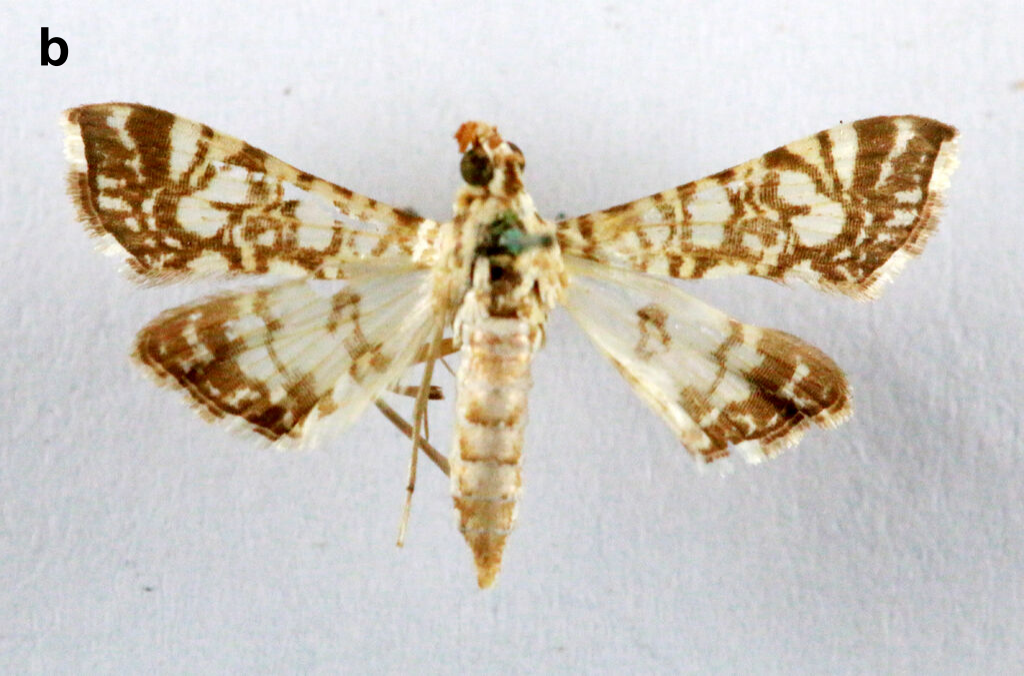
Glyphodesonychinalis

**Figure 30c.**
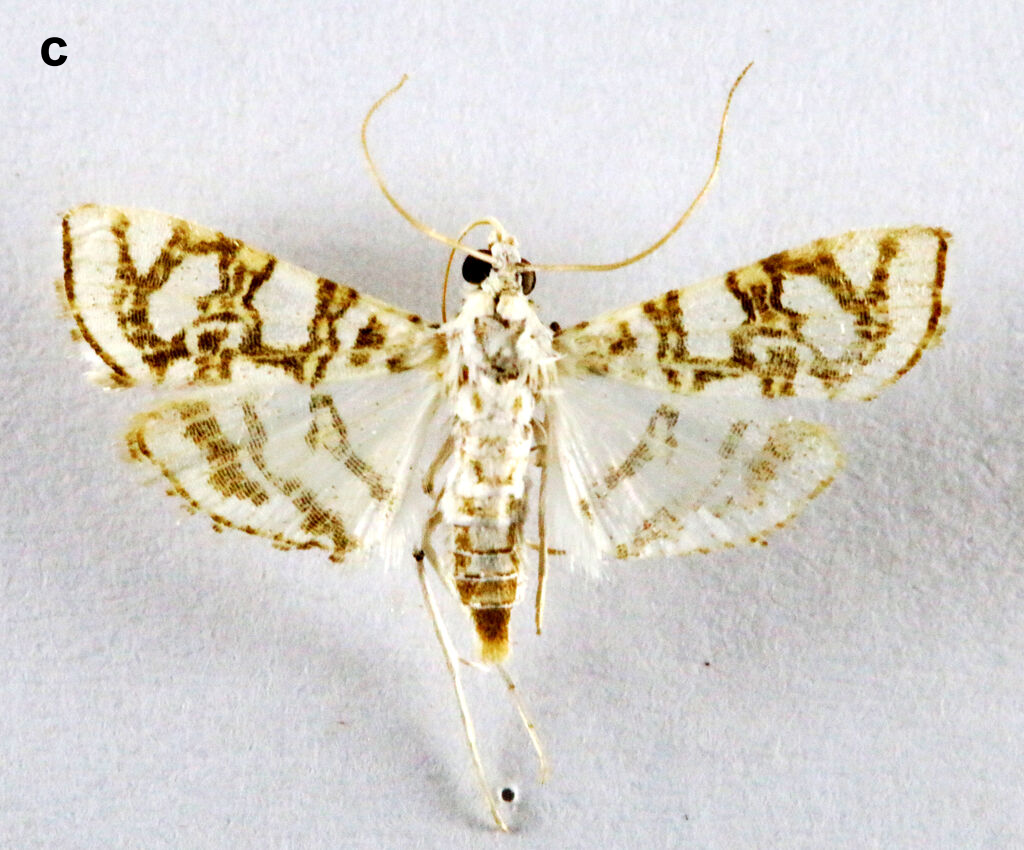
Haritalodesderogata

**Figure 30d.**
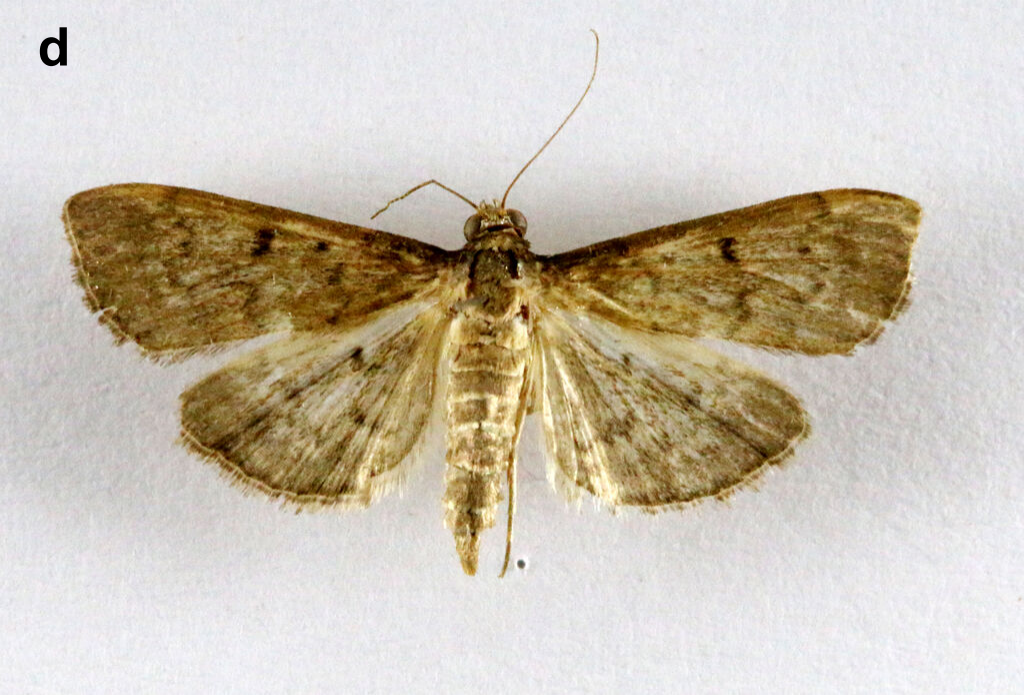
Herpetogrammalicarsisalis

**Figure 30e.**
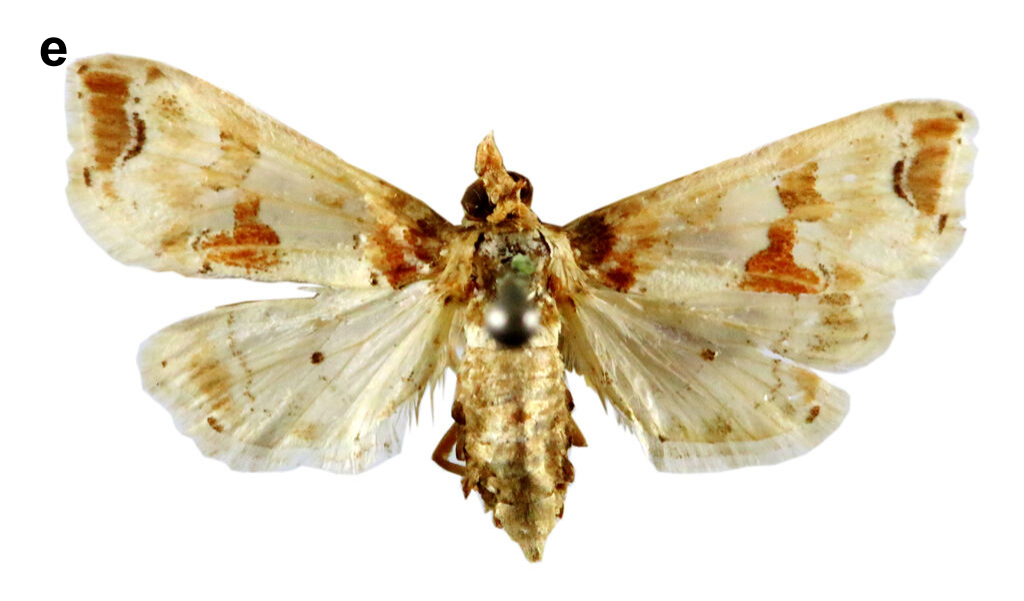
Leucinodesorbonalis

**Figure 30f.**
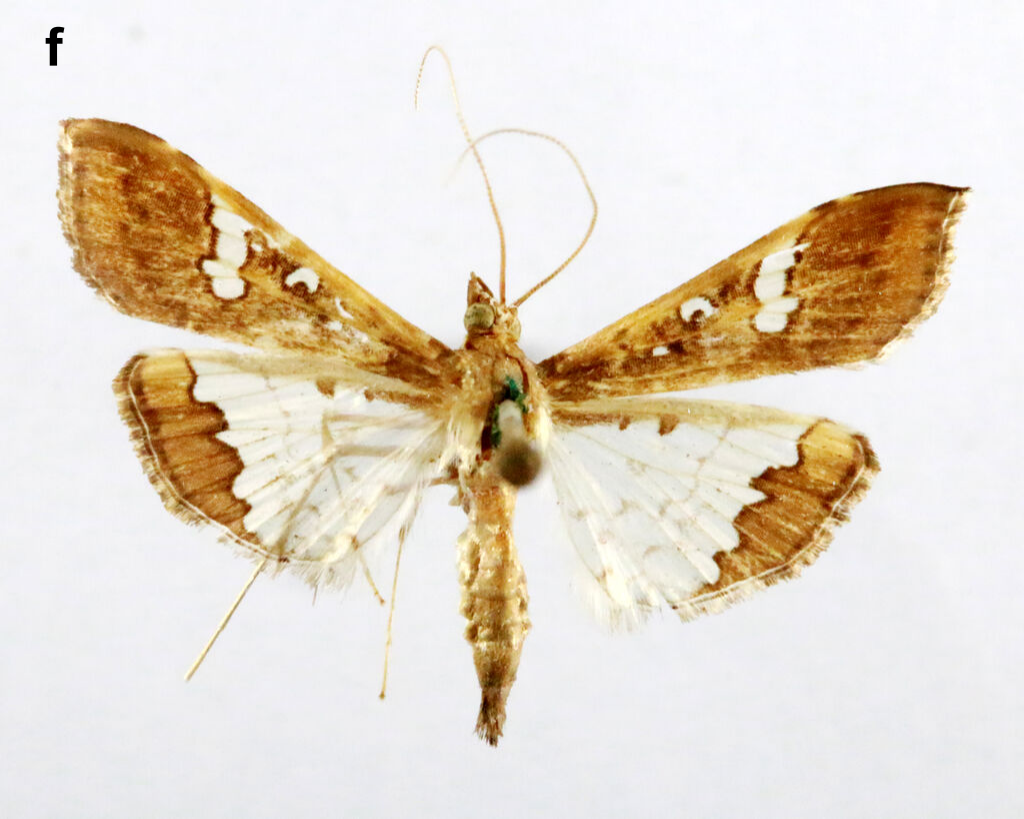
Marucavitrata

**Figure 31a.**
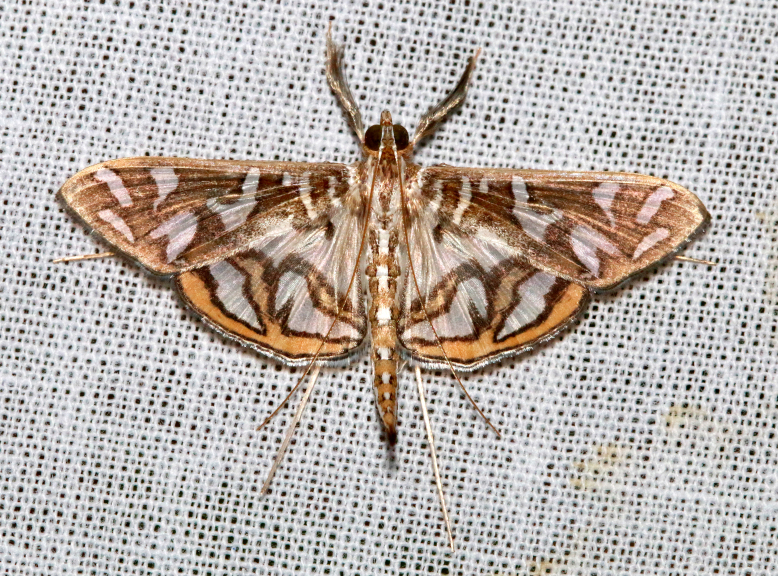
Nausinoeperspectata

**Figure 31b.**
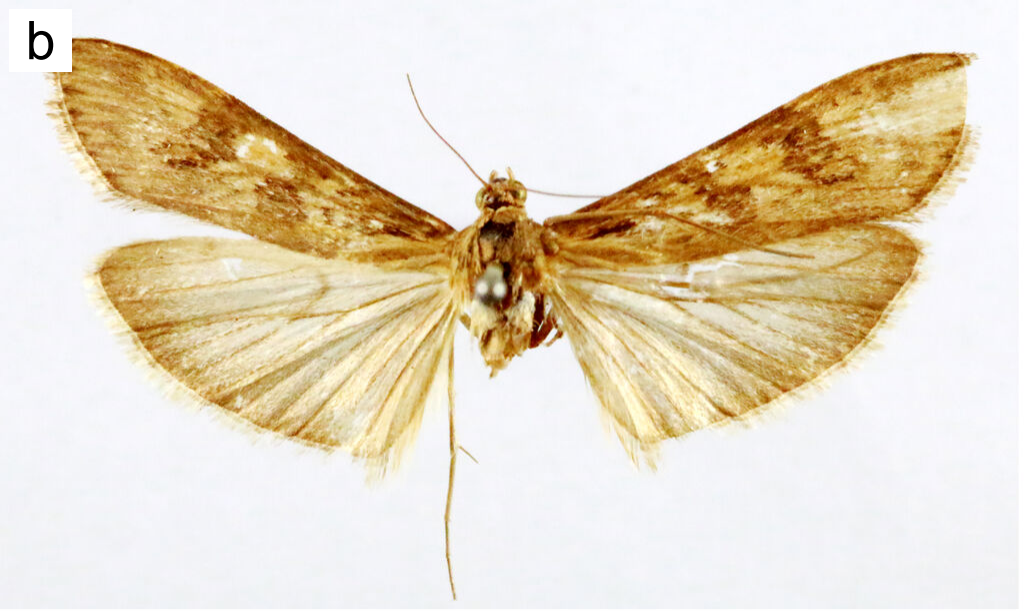
Nomophilanoctuella

**Figure 31c.**
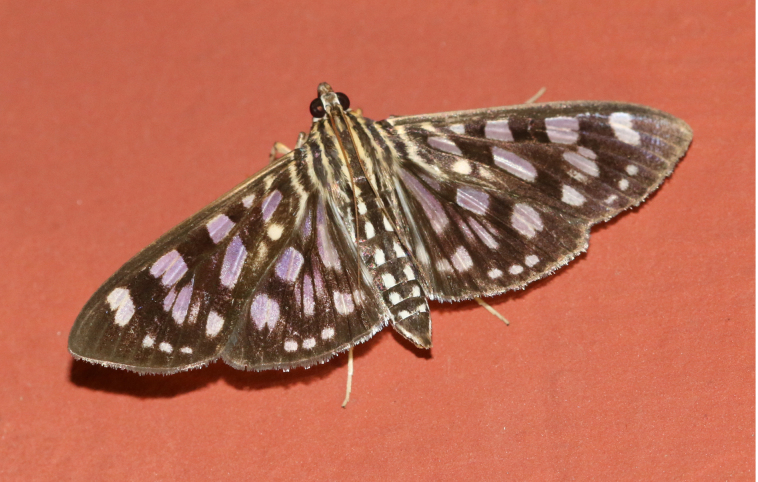
Pygospilatyres

**Figure 31d.**
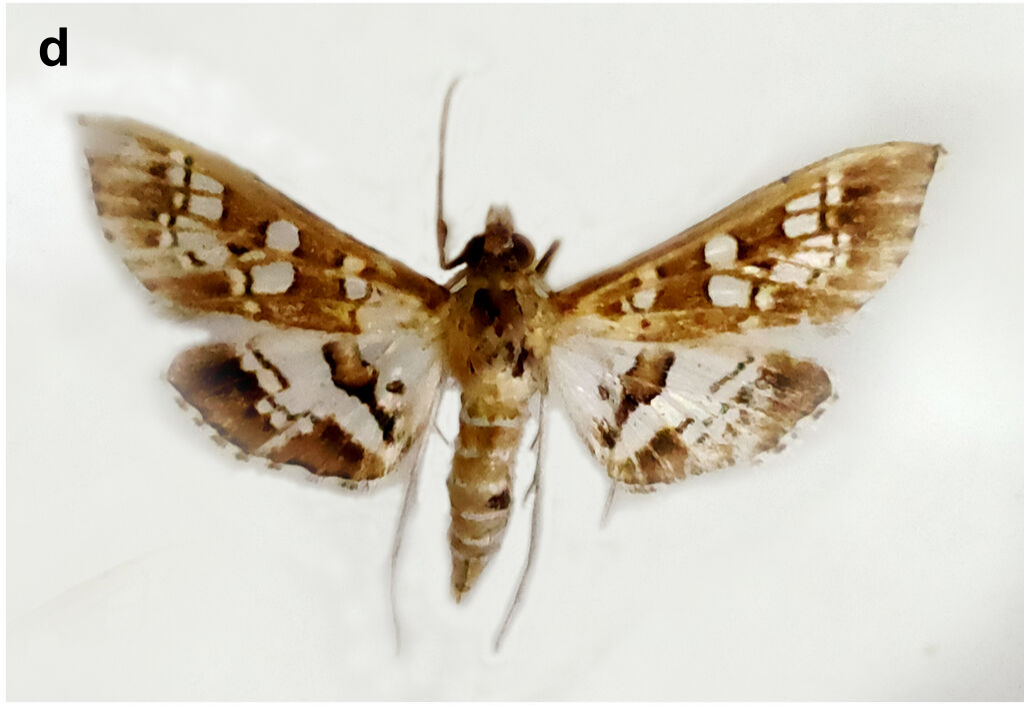
Sameodescancellalis

**Figure 31e.**
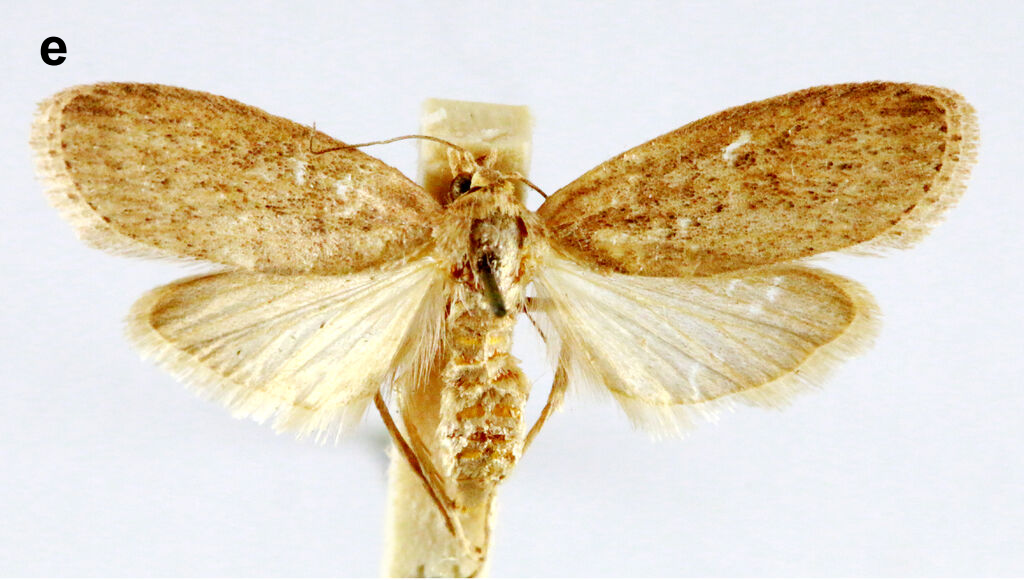
Trachylepidiafructicassiella

**Figure 31f.**
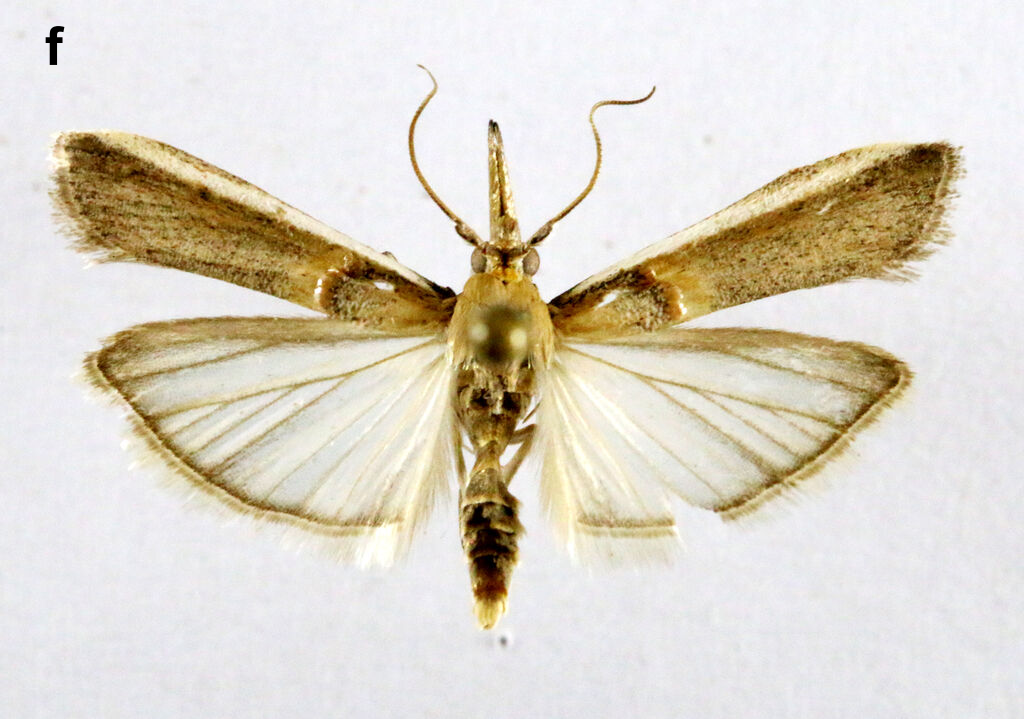
Etiellazinckenella

**Figure 32a.**
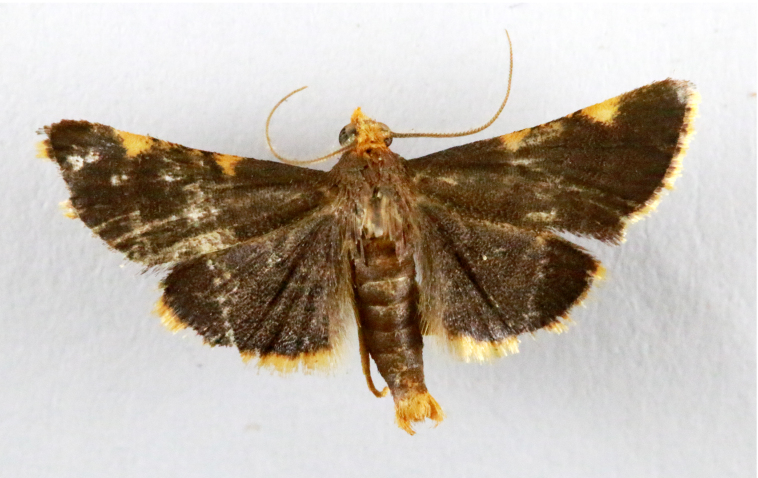
Hypsopygiamauritialis

**Figure 32b.**
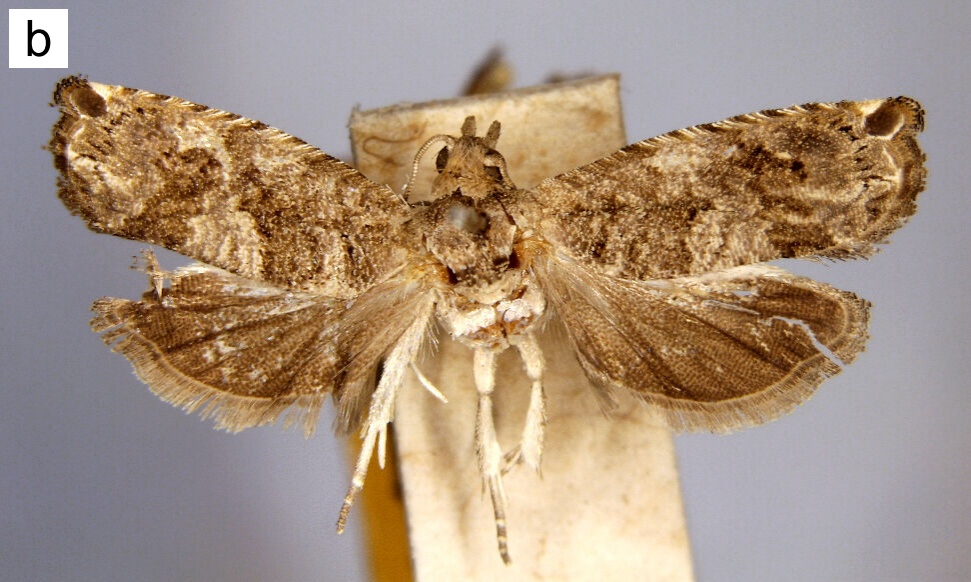
Acanthoclitabalanoptycha

**Figure 32c.**
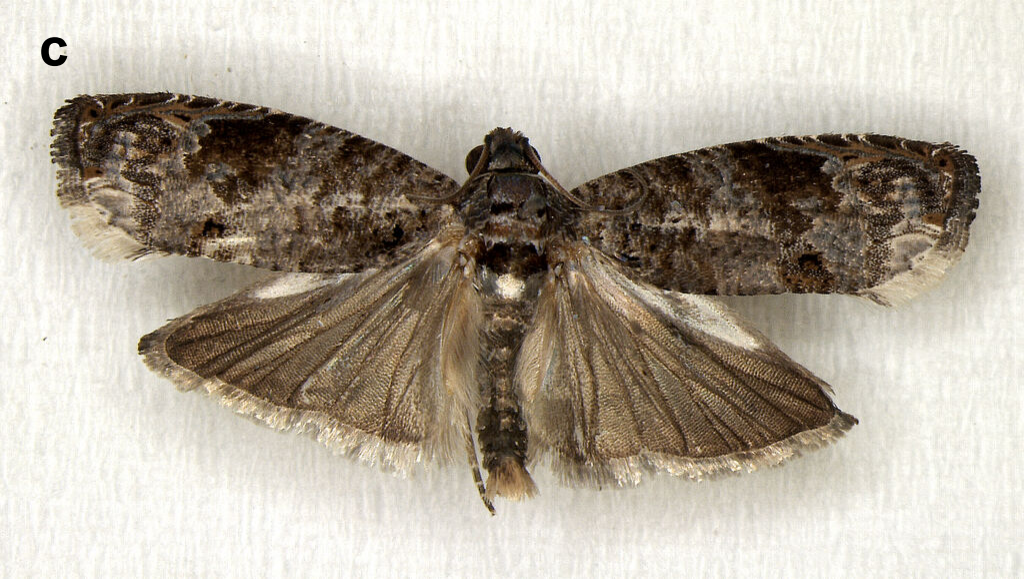
Duduaaprobola

**Figure 32d.**
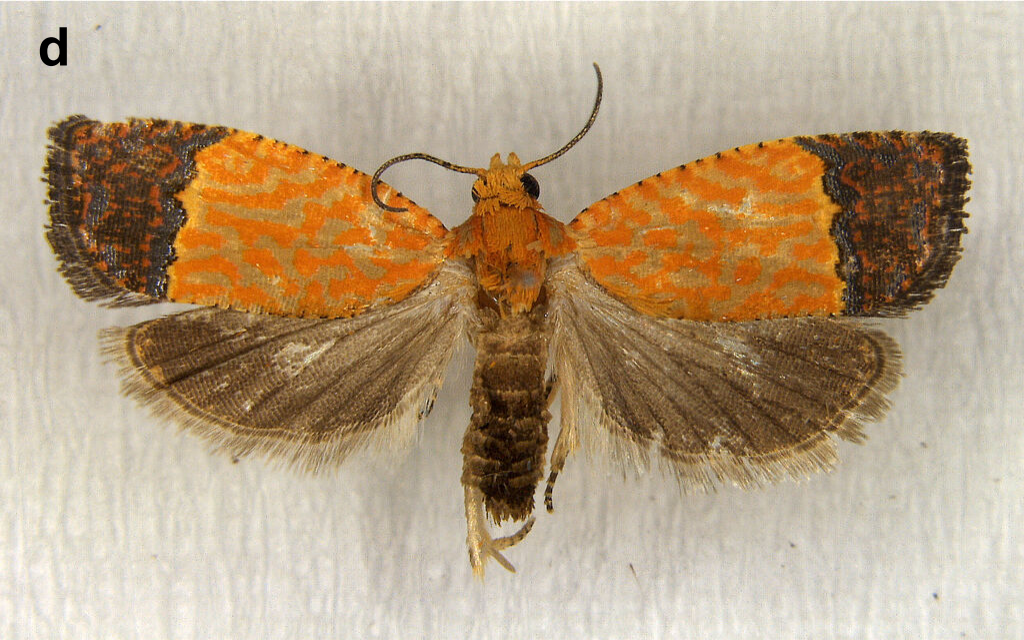
Loboschizakoenigiana

**Figure 32e.**
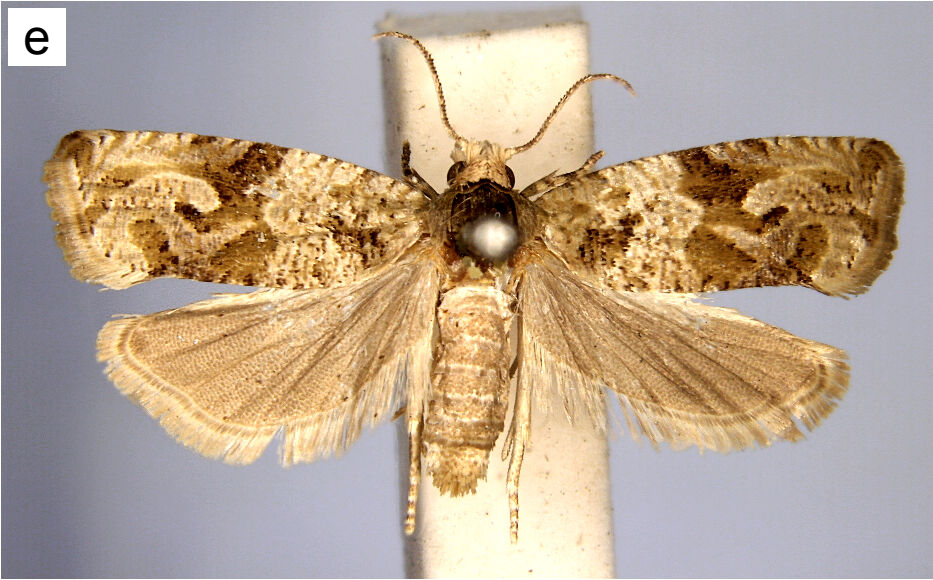
Syntozygaephippias

**Figure 32f.**
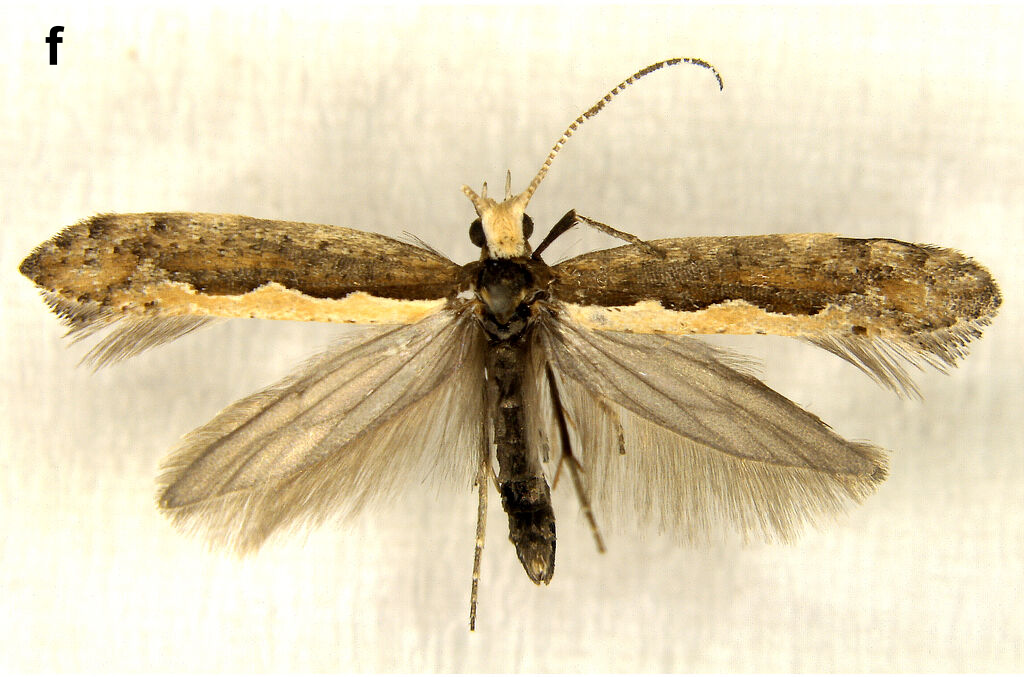
Plutellaxylostella

**Figure 33a.**
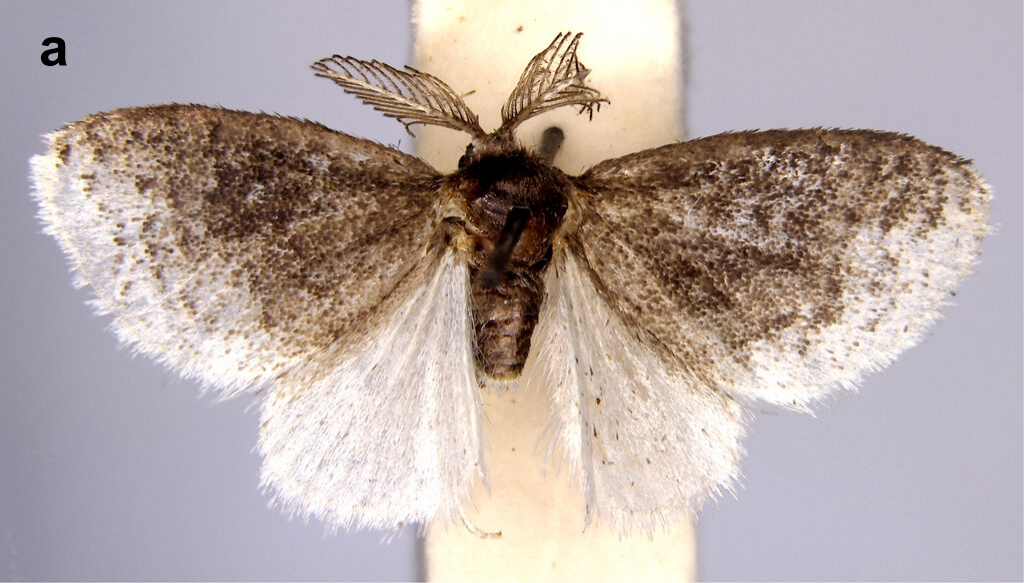
Fulgoraeciamelanoleuca

**Figure 33b.**
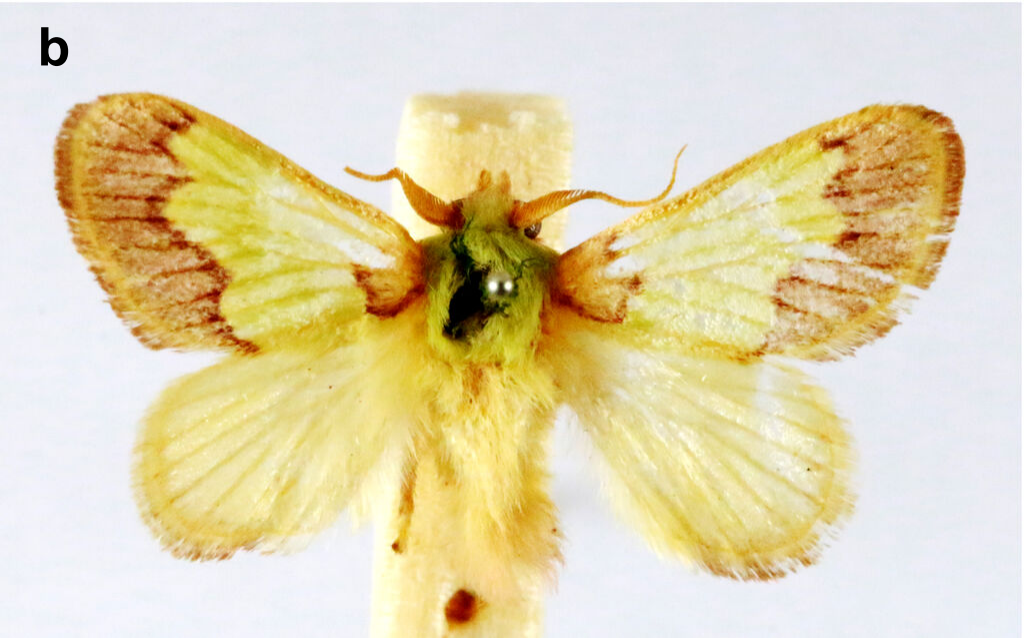
Aerginahilaris

**Figure 33c.**
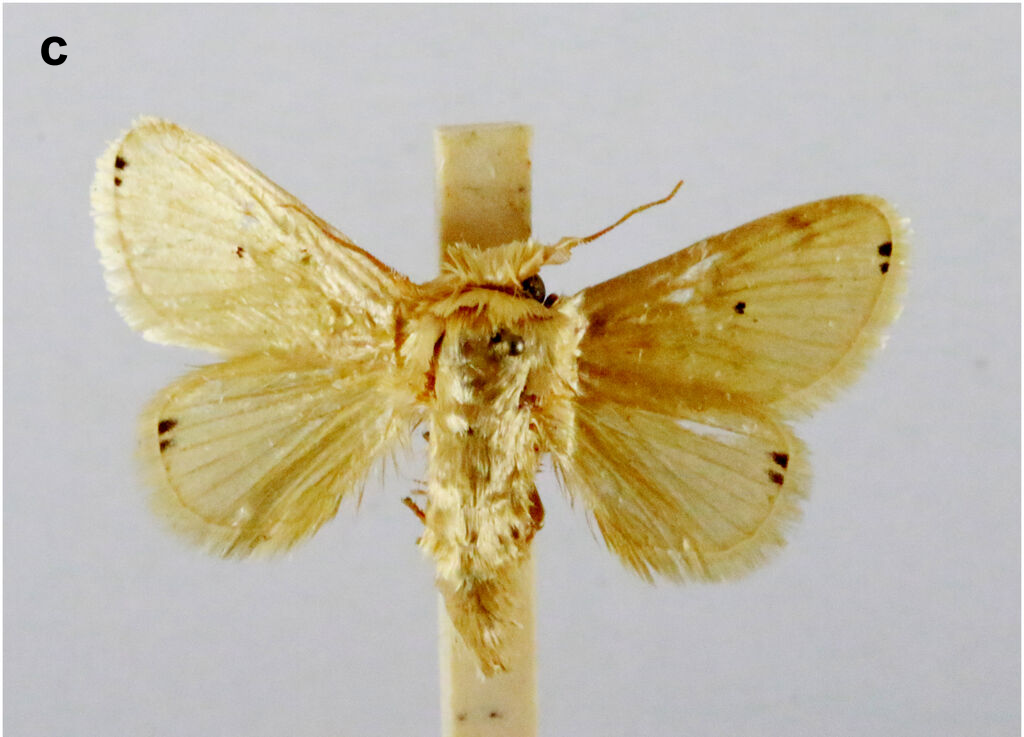
Althanivea

**Table 1.** Number of species under different superfamilies and families in Delhi.

**Superfamily**	**No. of species**	**Family**	**No. of species**
Bombycoidea	24	Bombycidae	2
		Eupterotidae	2
		Sphingidae	20
Cossoidea	1	Brachodidae	1
Gelechioidea	15	Blastobasidae	1
		Coleophoridae	1
		Cosmopterigidae	2
		Elachistidae	3
		Gelechiidae	5
		Oecophoridae	1
		Scythrididae	1
		Stathmopodidae	1
Geometroidea	37	Geometridae	36
		Uraniidae	1
Gracillarioidea	5	Gracillariidae	5
Lasiocampoidea	7	Lasiocampidae	7
Hyblaeoidea	1	Hyblaeidae	1
Noctuoidea	164	Erebidae	95
		Euteliidae	1
		Noctuidae	54
		Nolidae	14
Pterophoroidea	3	Pterophoridae	3
Pyraloidea	69	Crambidae	57
		Pyralidae	12
Tortricoidea	4	Tortricidae	4
Tineoidea	1	Psychidae	1
Yponomeutoidea	3	Lyonetiidae	1
		Plutellidae	1
		Yponomeutidae	1
Zygaenoidea	4	Epipyropidae	1
		Limacodidae	2
		Zygaenidae	1
